# A small molecule exerts selective antiviral activity by targeting the human cytomegalovirus nuclear egress complex

**DOI:** 10.1371/journal.ppat.1011781

**Published:** 2023-11-17

**Authors:** Han Chen, Ming F. Lye, Christoph Gorgulla, Scott B. Ficarro, Gregory D. Cuny, David A. Scott, Fan Wu, Paul W. Rothlauf, Xiaoou Wang, Rosio Fernandez, Jean M. Pesola, Sorin Draga, Jarrod A. Marto, James M. Hogle, Haribabu Arthanari, Donald M. Coen

**Affiliations:** 1 Department of Biological Chemistry and Molecular Pharmacology, Blavatnik Institute, Harvard Medical School, Boston, Massachusetts, United States of America; 2 Department of Cancer Biology, Dana-Farber Cancer Institute, Boston, Massachusetts, United States of America; 3 Department of Physics, Harvard University, Cambridge, Massachusetts, United States of America; 4 Department of Structural Biology, St. Jude’s Children’s Research Hospital, Memphis Tennessee United States of America; 5 Blais Proteomics Center, Dana-Farber Cancer Institute, Boston, Massachusetts, United States of America; 6 Center for Emergent Drug Targets, Dana-Farber Cancer Institute, Boston, Massachusetts, United States of America; 7 Department of Pathology, Harvard Medical School, Boston, Massachusetts, United States of America; 8 Department of Pharmacological and Pharmaceutical Sciences, University of Houston College of Pharmacy, Houston, Texas, United States of America; 9 Medicinal Chemistry Core, Dana-Farber Cancer Institute, Boston, Massachusetts, United States of America; 10 Virtual Discovery, Inc. Chestnut Hill, Massachusetts United States of America; 11 Non-Governmental Research Organization Biologic, Bucharest Romania; Tufts University School of Medicine, UNITED STATES

## Abstract

Human cytomegalovirus (HCMV) is an important pathogen for which new antiviral drugs are needed. HCMV, like other herpesviruses, encodes a nuclear egress complex (NEC) composed of two subunits, UL50 and UL53, whose interaction is crucial for viral replication. To explore whether small molecules can exert selective antiviral activity by inhibiting NEC subunit interactions, we established a homogeneous time-resolved fluorescence (HTRF) assay of these interactions and used it to screen >200,000 compound-containing wells. Two compounds, designated GK1 and GK2, which selectively inhibited this interaction in the HTRF assay with GK1 also active in a co-immunoprecipitation assay, exhibited more potent anti-HCMV activity than cytotoxicity or activity against another herpesvirus. At doses that substantially reduced HCMV plaque formation, GK1 and GK2 had little or no effect on the expression of viral proteins and reduced the co-localization of UL53 with UL50 at the nuclear rim in a subset of cells. GK1 and GK2 contain an acrylamide moiety predicted to covalently interact with cysteines, and an analog without this potential lacked activity. Mass spectrometric analysis showed binding of GK2 to multiple cysteines on UL50 and UL53. Nevertheless, substitution of cysteine 214 of UL53 with serine (C214S) ablated detectable inhibitory activity of GK1 and GK2 in vitro, and the C214S substitution engineered into HCMV conferred resistance to GK1, the more potent of the two inhibitors. Thus, GK1 exerts selective antiviral activity by targeting the NEC. Docking studies suggest that the acrylamide tethers one end of GK1 or GK2 to C214 within a pocket of UL53, permitting the other end of the molecule to sterically hinder UL50 to prevent NEC formation. Our results prove the concept that targeting the NEC with small molecules can selectively block HCMV replication. Such compounds could serve as a foundation for development of anti-HCMV drugs and as chemical tools for studying HCMV.

## Author summary

Human cytomegalovirus (HCMV) causes severe diseases in newborns and in adults with compromised immunity. With no available vaccine for this virus, and with the drawbacks of existing anti-HCMV drugs, there is a pressing need for new effective treatments. HCMV, like other herpesviruses, uses an unusual process called nuclear egress by which viral capsids exit the nucleus. The interaction of two HCMV proteins plays a crucial role in nuclear egress. To investigate the possibility that a small compound can inhibit production of infectious HCMV by specifically targeting this interaction, we screened ~200,000 chemicals and found two related compounds that specifically inhibit the interaction of the two proteins in biochemical assays and exert anti-HCMV activity in cells. Both the inhibition of the interaction and the antiviral activity of at least one of the compounds depend upon the compounds binding to a specific location on one of the viral proteins. Our results provide compelling evidence that targeting these proteins is a valid strategy for developing new anti-HCMV drugs. The compounds that we identified might serve as a starting point for development of such drugs and as tools for studying the virus.

## Introduction

The herpesvirus human cytomegalovirus (HCMV), the prototypic member of the betaherpesvirus subfamily, is a large (>230 kbp) double-stranded DNA virus with ubiquitous, worldwide distribution [1]. Although HCMV infection of immunocompetent individuals is typically asymptomatic, it can cause severe disease in immune-naïve newborns, with congenital HCMV infection being the most common cause of infant hearing loss. HCMV infection is also the leading cause of morbidity and mortality in immunocompromised individuals, including AIDS patients and transplant recipients, where opportunistic HCMV infections cause inflammatory disease in various organs [1].

Current approaches to control HCMV infections primarily focus on preemptive therapy and prophylaxis. HCMV encodes more than 100 viral proteins, with 45 open reading frames essential for viral replication [2,3]. However, few targets have been identified for effective drug treatments. Currently approved drugs interfere with the HCMV DNA polymerase that is crucial for viral DNA synthesis, the terminase complex that packages viral DNA during assembly, or UL97, a viral kinase that affects multiple processes during infection [1,4]. Although these drugs can prevent and/or ameliorate HCMV disease, some patients’ infections do not respond to them, and their effectiveness is often further limited by various toxicities, limited bioavailability, and/or the emergence of drug resistance during long-term therapeutic regimens [1,4]. Therefore, there is a critical need for development of new antiviral inhibitors to combat HCMV infection, particularly those with novel molecular targets, as viruses resistant to current drugs would be much less likely to be cross-resistant. Moreover, new antiviral inhibitors could serve as useful tools for studying virus biology and biochemistry.

One interesting potential drug target is the nuclear egress complex (NEC) of HCMV. Like many eukaryotic DNA viruses, HCMV and other herpesviruses replicate and package their genomes into capsids in the host cell nucleus. Newly synthesized herpesvirus nucleocapsids then transit to the cytoplasm using a fascinating process known as nuclear egress [5]. This process includes disruption of the nuclear lamina and budding through the inner nuclear membrane (INM), both of which are orchestrated by the NEC, followed by fusion of enveloped nucleocapsids with the outer nuclear membrane [6–9].

The HCMV NEC is a virally encoded, two-subunit protein heterodimer, comprised of an INM-anchored subunit (UL50) and a nucleoplasmic subunit (UL53) [10–12], which, based on work from other herpesviruses, forms higher order hexagonal arrays to promote budding [13–15]. Mutations inactivating either NEC subunit results in severely impaired production if not elimination of infectious virus [2,3,16,17]. Although nuclear egress was long thought to be a process unique to herpesviruses, a similar process has been identified for certain ribonucleoprotein particles in *Drosophila* muscle cells [18,19]. Nonetheless, there are important differences between the mechanisms of the *Drosophila* and viral processes (e.g., [20,21]). Similarly, although both HCMV NEC subunits share a structural element known as the Bergerat fold in common with a number of host proteins [22], their overall structures are unique to herpesvirus NECs [14,22–26]. Thus, the HCMV NEC is crucial for viral replication and differs markedly from host proteins, important properties for an antiviral drug target.

UL50 and UL53 heterodimerize by a mechanism in which portions of two alpha helices of UL53 nestle between the UL50 “core” and an alpha-helix of UL50 that appears to function like the moveable arm of a vise [22,25,26]. Single substitutions along this interface can drastically decrease UL50-UL53 interactions in vitro and in infected cells, and eliminate viral propagation; as such, this interface potentially could serve as a target for small molecule inhibitors [12,22,24,26,27]. Peptides corresponding to the UL53 alpha helices have been shown to inhibit UL50-UL53 binding in vitro [24,28,29], but these are not small molecules.

To attempt to identify small molecule inhibitors of NEC subunit interactions, protein fragment complementation and ELISA assays have been developed [24,29]. Neither of these, however, has been shown to be suitable for high throughput screening of large compound libraries. Although a paper, which appeared as we were completing the studies described below, used one of these assays to screen a small library and reported that a ~750 dalton compound, merbromin disrupts NEC interactions in vitro and inhibits HCMV replication, this study [30] and a subsequent paper [31] appear to have had important limitations (see Discussion).

To investigate the hypothesis that targeting the NEC with a small molecule could lead to selective antiviral activity, we developed a homogeneous time-resolved fluorescence (HTRF) assay for UL50-UL53 interactions, and used it to screen ~200,000 compounds. From the screening results, we selected two promising hits with some useful chemical features and further characterized their properties through multiple complementary approaches. From these studies, we conclude that one of these hits selectively acts against HCMV by targeting the NEC, potentially providing tools for studying NEC functions in infected cells and a starting point for development of new anti-HCMV drugs.

## Results

### Establishment of a high throughput assay for inhibitors of NEC subunit interactions

**Fig 1:**
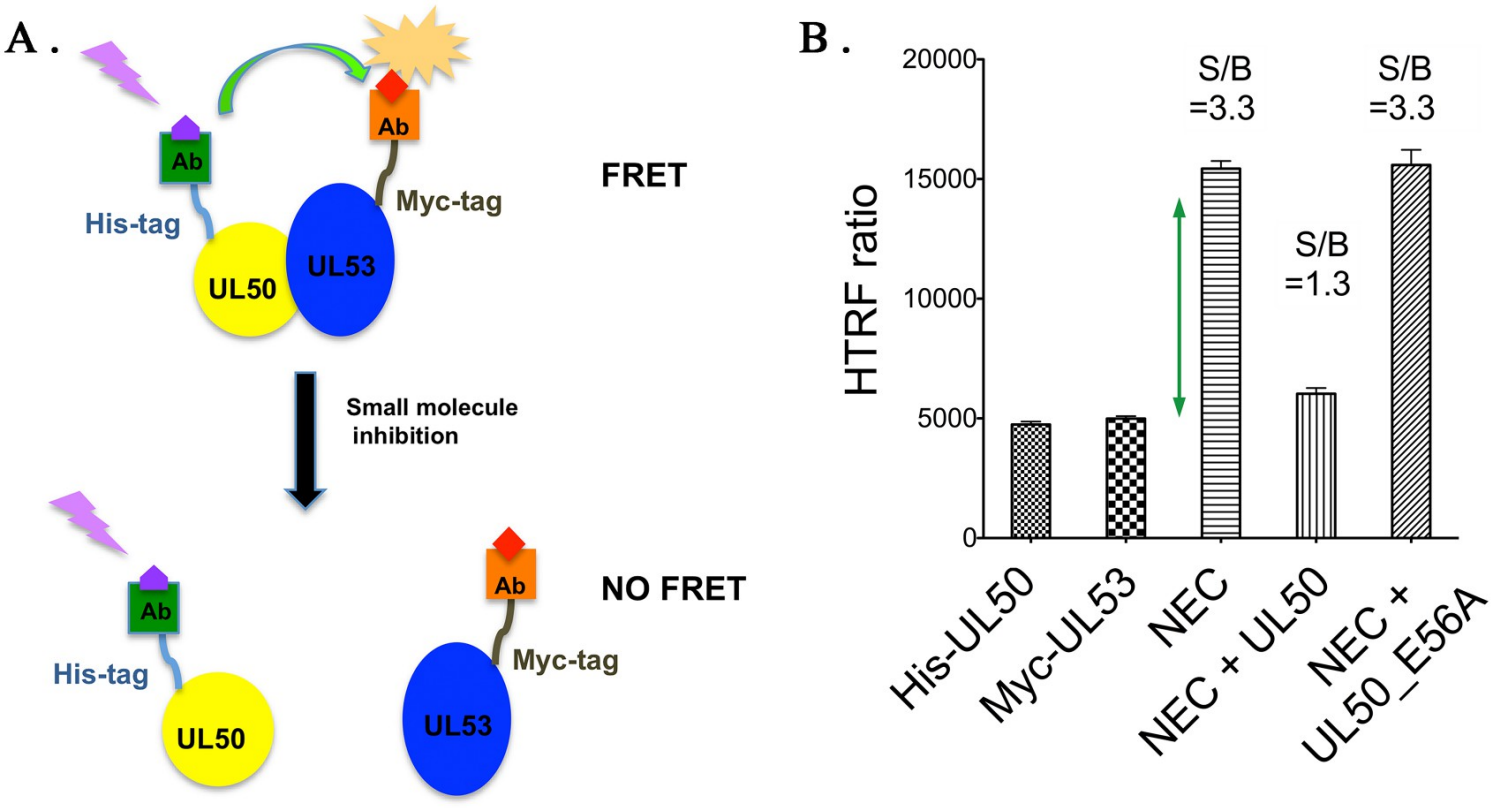
HTRF for high-throughput screening of HCMV NEC inhibitors. (A) HTRF assay working principle. His-tagged UL50 and Myc-tagged UL53 were reacted with antibodies linked to a donor probe and an acceptor probe, respectively. Formation of the NEC brings the two probes in proximity to generate FRET signal. Small compounds that inhibit NEC formation reduce the likelihood that donor probe will be near the acceptor probe, leading to reduced FRET signal. (B) Validation of HTRF assay. The HTRF ratio for the NEC (signal, S) was ~3-fold higher than the background (B) HTRF ratios for His-UL50 or Myc-UL53 alone (S/B ~3). Untagged UL50 decreased S/B to near background levels, serving as a positive control, while UL50_E56A, which is highly defective for UL53 binding [12] did not decrease S/B, serving as a negative control. Error bars are standard deviations of the mean from three replicates.

We investigated whether HTRF technology would be suitable for assaying inhibitors of NEC subunit interactions in a high throughput format. HTRF is a single step assay that uses fluorescence resonance energy transfer (FRET) between interacting partner proteins, one with a donor fluorophore and the other with an acceptor. Both fluorophores have long-lived emissions, allowing for time-resolved measurement that reduces interference from short-lived background fluorescence. The FRET and donor emissions are at different wavelengths, so that the ratio of their signals provides normalization and mitigates assay interference as well as instrument errors that can cause well to well variability [32]. For the HCMV NEC, we used UL50 and UL53 tagged at their N-termini with polyhistidine (His) or a Myc epitope, respectively. The UL53 construct also included a linker of alternating glycine and serine residues between the tag and the protein to facilitate fluorophore-tagged antibody accessibility. The His tag is recognized by an antibody coupled to a caged terbium cryptate fluorescence donor that is resistant to photobleaching and the Myc epitope is recognized by an antibody coupled to a d2 red fluorescence acceptor so that when the two antibodies are in proximity, a FRET signal will be generated (Fig 1A). The UL50 and UL53 proteins were independently purified using affinity chromatography, incubated overnight at a 1:2 molar ratio of UL53 to UL50 at 4°C, and the complex purified using size-exclusion chromatography to ensure active protein (S1 Fig). Various concentrations of donor and acceptor antibodies were tested to identify a combination that produced a high signal to noise ratio while also limiting the amounts of reagents used (due to cost), with 4 nM donor and 40 nM acceptor found optimal. At 0.3 μΜ NEC (the *K*_d_ for the complex [12], which permits exchange between bound and unbound subunits over the time of the assay), the HTRF ratios (acceptor emission/donor emission x 10,000) for the tagged UL50-UL53 complex were ~3-fold higher than the background signals from either tagged UL50 or UL53 alone at those concentrations (Fig 1B). Moreover, when 3 μΜ untagged UL50 was added to the tagged complex to block subunit interactions, the signal was reduced to near background levels. In contrast, an untagged UL50 with a substitution (E56A) that reduces UL53 binding by more than 100-fold [26] failed to inhibit the HTRF signal.

To determine the feasibility of a robust screening experiment, a 384 well plate with 128 replicates each of tagged NEC complex with either untagged UL50 or untagged UL53 as positive control inhibitors, or DMSO as a negative control was set up to determine Z’ scores. Samples with DMSO established the maximal HTRF signal, setting the upper limits of the assay, while untagged UL50 and UL53 provided the signal with an inhibitor, setting the lower limits of the assay. A Z’ score greater than 0.5 indicates an assay with sufficiently wide separation between the minimal and maximal signals as well as sufficiently low variability in the measurements. Untagged UL50 and untagged UL53 generated Z’ scores of 0.725 and 0.723, respectively, indicating the suitability of this system for high throughput screening.

### Two related hit compounds selectively inhibit NEC subunit interactions

**Fig 2:**
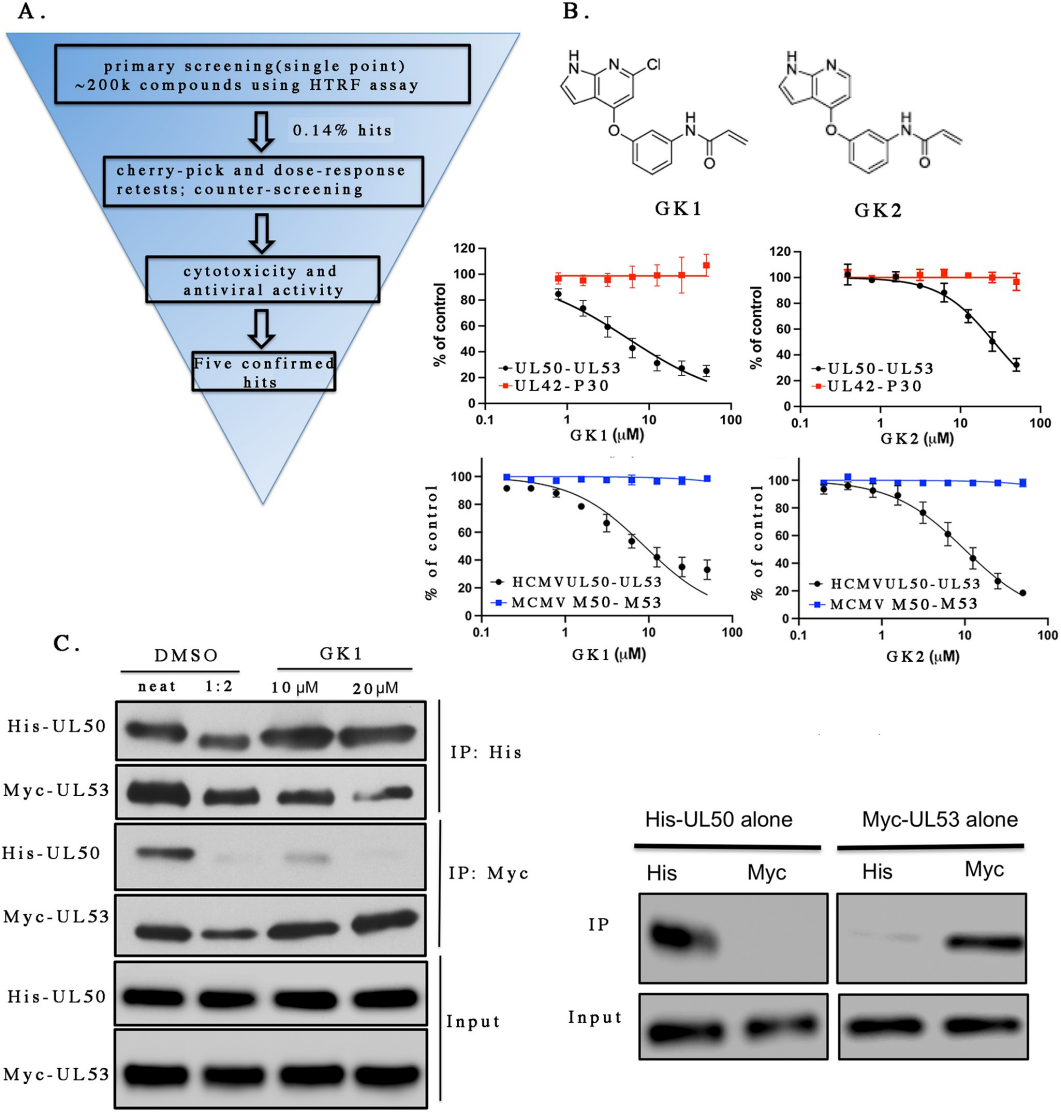
Identification of two hits GK1 and GK2 that selectively disrupt NEC subunit interactions. (A) High-throughput screening flow-chart. About 200,000 compounds were screened at a single concentration using the HTRF assay outlined in Fig 1. The hit rate was 0.14%. Hits were retested first at a single concentration from the libraries (“cherry pick”) and those confirmed positive were then obtained from commercial or academic sources and tested in dose-response HTRF assays of HCMV NEC interactions and, as a counterscreen, HSV-1 P30-UL42 interactions. Compounds that showed selective inhibition of NEC interactions were then tested for antiviral activity and cytotoxicity in cell-based assays. Five confirmed hits were found. (B) The chemical structures of GK1 and GK2 (top) and their inhibitory activity towards HCMV UL50-UL53 interactions (middle and bottom, black), HSV-1 P30-UL42 interactions (middle, red), and MCMV M50-M53 interactions (bottom, blue). The normalized FRET signal at each concentration of inhibitor relative to control without inhibitor was plotted versus the concentration of GK1 or GK2. Curves were drawn using nonlinear regression analysis in GraphPad Prism 9 for MacOS. Error bars represent standard deviations from three independent experiments. Where no error bars are seen, the standard deviations were too small to be visible. (C) Co-immunoprecipitation assay. Left: His-UL50 and Myc-UL53 were mixed either with DMSO vehicle (left two lanes) or the indicated concentrations of GK1 (right two lanes), as indicated to the top of the image. Aliquots were reserved to show the input proteins (bottom two rows) or immunoprecipitated with either anti-His antibody (top two rows) or anti-Myc antibody (middle two rows), as indicated to the right of the image. The second lane is a two-fold dilution of the material in the first lane. The proteins were analyzed by Western blotting using either anti-His antibody to detect His-UL50 or anti-Myc antibody to detect Myc-UL53 as indicated to the left of the image. Elution from anti-His beads by imidazole is more efficient than elution from anti-Myc beads with peptide, so signals for Myc-UL53 in anti-His immunoprecipitates are higher than those for His-UL50 in corresponding anti-Myc immunoprecipitates. Right. Input (bottom two rows) or immunoprecipitation of His-UL50 alone (first two lanes) or Myc-UL53 alone (right two lanes), as indicated at the top of the image, by either anti-His (His) or anti-Myc (Myc) antibodies as indicated just above the image. The blot with the first two lanes was probed with anti-His antibody and the blot with the second two lanes was probed with anti-Myc antibody. A second independent experiment showed similar results.

A flowchart of the pipeline for identifying and testing hit compounds is shown in Fig 2A. We used the HTRF assay first in a pilot screen and then to screen 45 libraries (total of 200,217 chemical-containing wells) containing a total of 192,546 unique compounds (some compounds were found in more than one library) at the ICCB-Longwood facility (S1 Appendix). Compound libraries in stock compound plates were screened on duplicate 384 well sample screening plates, with 16 wells each of untagged UL50 and DMSO on each plate serving as positive and negative controls respectively. The Z’ scores for all the sample plates were consistently between 0.6 to 0.85 throughout the screening.

Compounds were judged to be hits if on average they inhibited the HTRF signal by at least 50 percent. The vast majority of compounds scored negative in the screen, including merbromin (S1 Table; see Discussion); only 0.14% (286) of the wells scored as hits. To confirm the activity of the hits, we resampled these hit compounds from the libraries (“cherry-picked”) and tested them at the same concentration used in the initial screen in two assays—the assay for inhibition of NEC subunit interactions used in the screen and a counterscreen HTRF assay that we developed for the interaction of two unrelated proteins–glutathione-S-transferase (GST) fused to the C-terminal 36 residues of UL30, the catalytic subunit of herpes simplex virus 1 (HSV-1) DNA polymerase (P30), and UL42, the HSV-1 polymerase processivity subunit (S2 Fig). Ten compounds passed this test. We then obtained these ten compounds from commercial or academic sources and performed HTRF-based dose-response studies. Eight compounds that showed 50% inhibitory concentration (IC_50_) values below 50 μM for the tagged NEC complex and no meaningful activity observed in the counterscreen were selected. None of these compounds were known promiscuous inhibitors (pan-assay interference compounds; PAINS [33]). These compounds were then tested for anti-HCMV activity using a virus expressing green fluorescent protein (GFP) and an automated plaque reduction assay, which uses high content imaging to distinguish single infected cells from clusters of infected cells, and for cytotoxicity (see below). Five compounds showed antiviral activity that was more potent than cytotoxic activity.

We report here on two of these compounds, previously designated as WZ4140 and WZ4141, which were within a library of protein kinase inhibitors established by the laboratory of Nathanael Gray (Gray Kinase Library), that we term GK1 and GK2. GK1 and GK2 each consist of a 7-azaindole linked by an ether at the 4-position to a phenyl group with an acrylamide substituent. The compounds differ only by a chlorine on the 7-azaindole of GK1 that is a hydrogen on GK2 (Fig 2B, top). For use in the experiments below, the compounds were >95% pure (S2 Appendix). As shown in Fig 2B, bottom, GK1 and GK2 inhibited HTRF signal in the NEC subunit interaction assay (50% inhibitory concentrations (IC_50_s) of 5.3 ± 1.1 μM and 21 ± 1 μM, respectively), but did not show meaningful inhibition in the HSV-1 P30-UL42 counterscreen assay at all concentrations tested (up to 50 μM). Moreover, GK1 and GK2 also did not show inhibition in an HTRF assay for interactions of mouse cytomegalovirus NEC subunits (Fig 2B, bottom and S2A Fig), further suggesting selective inhibition. GK1 also showed little or no effect on HTRF signal in the absence of protein (interference; S3 Fig).

In principle, the HTRF assay could have been detecting a large conformational change in the NEC that moves the tags apart rather than disruption of the UL50-UL53 interaction. We therefore further tested the ability of GK1, the more potent of the two compounds, to inhibit UL50-UL53 interactions using a different assay, co-immunoprecipitation. When His-tagged UL50 and Myc-tagged UL53 were mixed together at 0.3 μM (the *K*_d_ for NEC formation [12] so bound and unbound subunits can exchange over the time of the assay), and incubated with anti-His antibody or anti-Myc antibody in the absence of GK1 (DMSO control), in each case the NEC subunit lacking the immunoreactive tag was efficiently co-immunoprecipitated (Fig 2C, left, left two lanes). In the presence of 10 or 20 μM GK1, the compound had no evident effect on IP of His-UL50 by anti-His antibody, as expected, but decreased co-immunoprecipitation of Myc-UL53 by >2-fold or even more, indicating loss of UL50-UL53 complexes (Fig 2C, left; compare right two lanes with dilutions in left two lanes). Similarly, when the two NEC subunits were mixed and immunoprecipitated with anti-Myc antibody, these concentrations of GK1 had no evident effect on immunoprecipitation of Myc-UL53 as expected, but decreased co-immunoprecipitation of His-UL50 (Fig 2C, left), again indicating loss of UL50-UL53 complexes. Anti-Myc antibody did not immunoprecipitate His-UL50 detectably in the absence of Myc-UL53, and anti-His antibody failed to immunoprecipitate Myc-UL53 efficiently in the absence of His-UL50 (Fig 2C, right). We conclude that GK1 interferes with the interaction of UL50 and UL53.

### GK1 and GK2 inhibit HCMV replication

**Fig 3:**
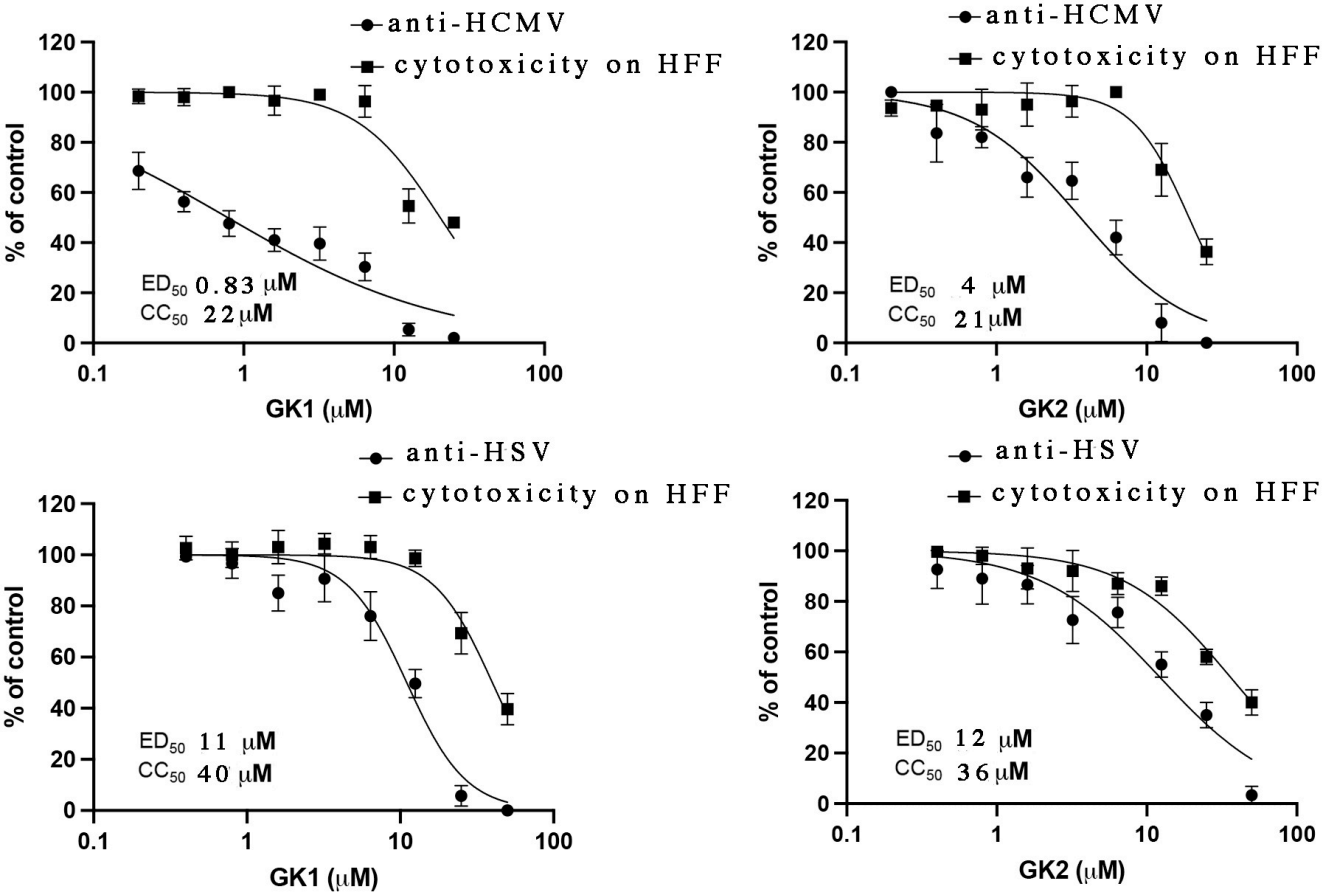
Antiviral activities and cytotoxicity of GK1 and GK2. HFF cells were infected with HCMV expressing GFP (53-F BADGFP; top) or HSV-1 strain KOS (bottom), respectively, and then treated with medium containing DMSO vehicle or the indicated concentrations of GK1 and GK2. The antiviral activities of GK1 and GK2 against HCMV (top panels) were assessed at 6 dpi using an automated plaque reduction assay. The antiviral activities of GK1 and GK2 against HSV (bottom panels) were assessed at 2 dpi using a standard plaque reduction assay. In parallel, uninfected cells were treated with DMSO vehicle or the same concentrations of inhibitors for 6 days (top) or 2 days (bottom), and cytotoxicity was measured using a WST-1 assay. The number of plaques at each concentration of inhibitor relative to the number of plaques with DMSO vehicle was plotted versus the concentrations of the inhibitors, and the ED_50_’s determined using nonlinear regression analysis in GraphPad Prism 9 for MacOS. Absorbances in the WST-1 assay at different concentrations of inhibitors relative to the absorbance with DMSO vehicle were plotted versus the concentrations of the inhibitors, and CC_50_’s determined using nonlinear regression analysis in GraphPad Prism 9 for MacOS. Error bars represent standard deviations from three independent experiments. Where no error bars are seen, the standard deviations were too small to be visible.

We tested whether GK1 and GK2 inhibit HCMV replication in human foreskin fibroblasts (HFFs) using a GFP-expressing HCMV and a high-content imaging, automated plaque reduction assay, and compared their antiviral potencies measured in that assay to their cytotoxicities using a WST-1 assay that measures cell metabolic activity. In the automated assay, GK1 inhibited HCMV plaque formation with a 50% effective dose (ED_50_) of 0.83 μM (Fig 3, top left). A WST-1 assay performed in parallel over the six-day timeframe of the automated plaque reduction assay showed a 50% cytotoxic concentration (CC_50_) of 22 μM (Fig 3, top left), 27-fold higher than the antiviral ED_50_. GK2 inhibited HCMV plaque formation with an ED_50_ of 4 μM, higher than that for GK1, mirroring its less potent inhibition of NEC subunit interactions in vitro (Fig 3, top right). Its CC_50_ in a parallel assay was 21 μM (Fig 3, top right), 5.4-fold higher than its antiviral ED_50_ (Fig 3, top right).

As a second assessment of selectivity, we performed standard plaque reduction assays of HSV-1 in HFFs. GK1 and GK2 both exhibited less potent anti-HSV-1 activity (ED_50_’s of 11 and 12 μM, respectively; Fig 3, bottom) than they did anti-HCMV activity. In WST-1 assays conducted in parallel over the two-day time frame of the HSV-1 plaque reduction assay, cytotoxic potencies of GK1 and GK2 were only 3- to 4-fold higher than their antiviral potencies. The higher CC_50_ values in this WST-1 assay relative to the one accompanying the anti-HCMV assay were likely due to the former’s shorter time frame, resulting in less cytotoxicity.

Although there is a possibility that the differences in the plaque assays used contributed to the higher ED_50_’s for anti-HSV-1 activity than for anti-HCMV activity, the results taken together suggested selective anti-HCMV activity of the two hit compounds GK1 and GK2, especially GK1.

### GK1 and GK2 inhibit late events in the viral replication cycle and modestly affect co-localization of UL50 and UL53

**Fig 4:**
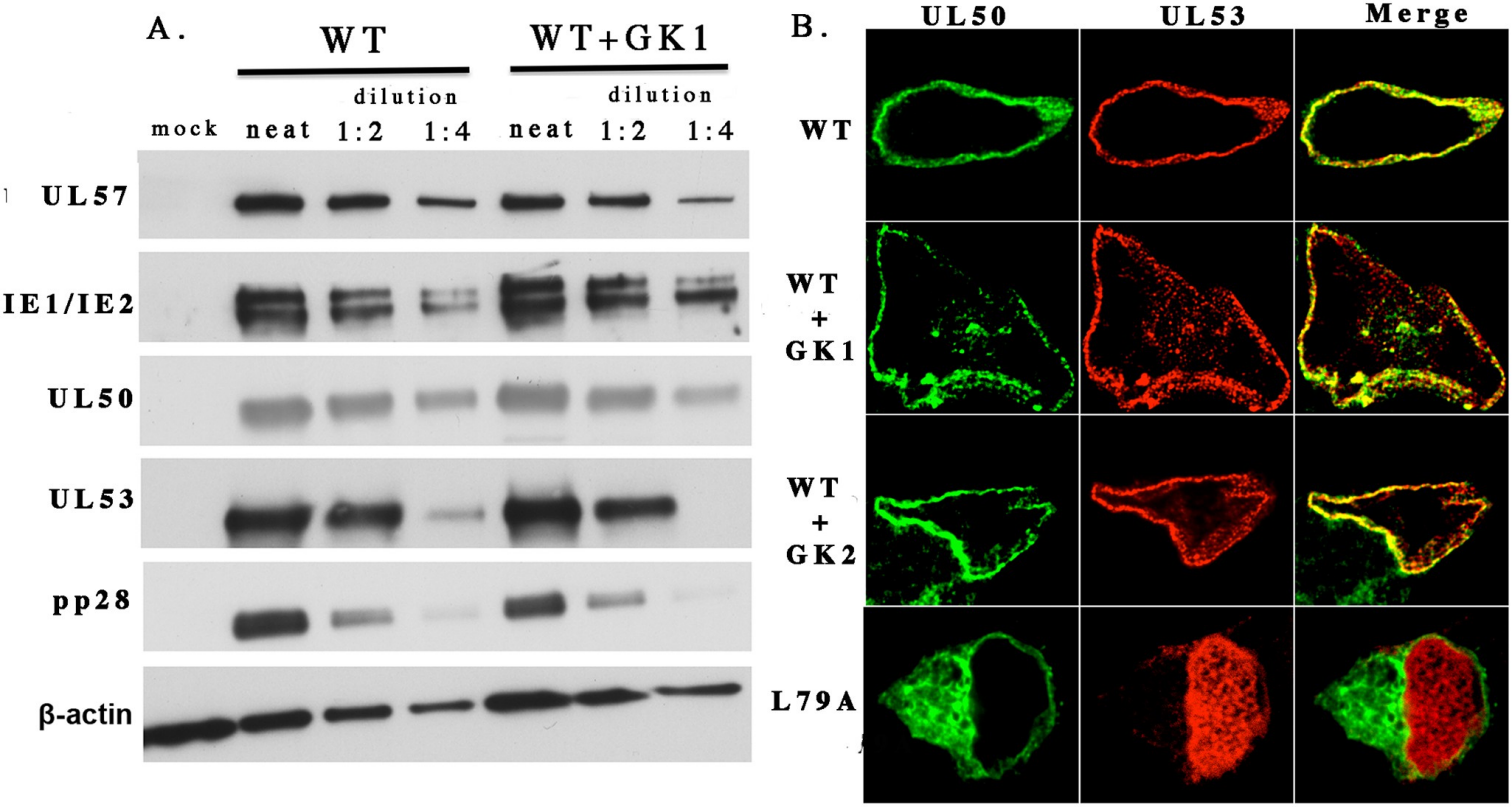
Effects on HCMV protein expression and co-localization of UL50 and UL53 in infected cells. (A) HFF cells were mock infected or infected with 53-F BADGFP HCMV at an MOI of 1. Following absorption, medium containing either DMSO or 10 μM GK1 was added. At 72 hpi, cells were lysed, proteins resolved using SDS-PAGE, and the expression of the viral proteins indicated to the left was assessed by immunoblotting. Similar results were obtained in a second independent experiment. (B) HFF cells were infected with WT HCMV (top three rows) or *UL53* mutant L79A (bottom row) at an MOI of 1. Following absorption, medium containing either DMSO (top and bottom rows), 10 μM GK1 (second row from top) or 10 μM GK2 (third row from top) was added. At 72 hpi, cells were fixed and stained with antibodies to detect the proteins indicated at the top–the rightmost column shows the merged images.

To investigate whether GK1 and GK2 affect HCMV gene expression, we mock-infected or infected cells with 53-F [16], a virus that expresses FLAG-tagged UL53 (FLAG-UL53) and GFP, but is otherwise wild type (WT), at a multiplicity of infection (MOI) of 1 in the presence of 10 μM GK1, a dose that reduces plaque formation by ≥5-fold (Fig 3). At 72 hours post-infection (hpi), we harvested the cells and examined HCMV protein expression by Western blot (Fig 4A). We saw no discernible effect (<2-fold based on dilution series) of GK1 on expression of immediate early proteins IE1 and IE2, the early protein UL57, on the “leaky” late proteins UL50 or UL53, or on the true late protein pp28. Similar results were observed with 10 μM GK2 (S4 Fig), and are consistent with results from a screen in which GK1 or GK2 (at 1 μM) did not score as positive for inhibition of pp28 expression [34]. These results suggest that GK1 and GK2 act subsequent to late gene expression and thus also subsequent to prior steps including DNA synthesis.

We then asked if the two compounds affect co-localization of UL53 with UL50 at the nuclear rim. We infected cells with HCMV 53-F at an MOI of 1 in the absence of compound or in the presence of 10 μM GK1 or GK2, and at 72 hpi stained with antibody against UL50 and FLAG-UL53. As a positive control, we also infected cells in the absence of compound with a similar virus that contains a substitution in UL53 (L79A) that strongly reduces interaction with UL50 [12]. In nearly all untreated WT-infected cells (Fig 4B, top), UL50 and UL53 co-localized at the nuclear rim with little if any UL53 in the nucleoplasm. In nearly all L79A-infected cells, although UL50 remained at the nuclear rim, most UL53 was found in the nucleoplasm (Fig 4B, bottom). In GK1- or GK2-treated WT-infected cells, a subset of cells (~ 20%) exhibited substantial nucleoplasmic UL53 staining, but not as much as in L79A-infected cells (Fig 4B, middle). We also note that the nuclei did not adopt the oval shape seen in L79A-infected cells, but exhibited distortions characteristic of nuclear lamina disruption that are NEC-dependent [16]. These differences may not be surprising as the concentrations of GK1 and GK2 applied to the cells (10 μM) only reduced NEC subunit interactions by ~2.5-fold in the HTRF assay, while the L79A substitution much more drastically affected these interactions (>150-fold in a calorimetric assay) [12], with the caveat that the assays used were different and other explanations are possible (see Discussion). Regardless, although the effects of the compounds were modest, they provide evidence that GK1 and GK2 affect NEC interactions in infected cells.

### Covalent inhibition of NEC subunit interactions via a specific cysteine

**Fig 5:**
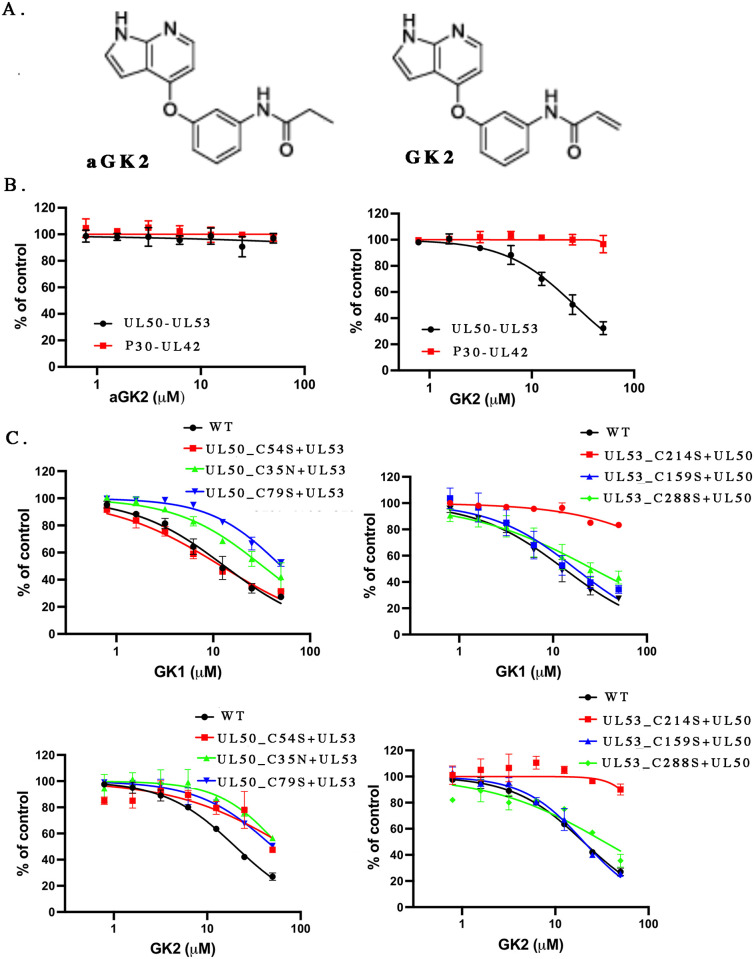
Covalent interaction via UL53 C214. (A) Chemical structures of aGK2 (left) and GK2 (right). (B) Comparison of the inhibitory activities of aGK2 (left) and GK2 against HCMV UL50-UL53 in HTRF assays (black). P30-UL42 served as the counter-screen HTRF assay (red). Error bars represent standard deviations from three independent experiments. (C) The inhibitory activities in the HCMV UL50-UL53 HTRF assay of GK1 (top graphs) and GK2 (bottom graphs) against the interactions of WT (black) or the indicated mutant (red, green, or blue) UL50 proteins with WT UL53 (left graphs) and the interactions of WT (black) or the indicated mutant UL53 (red, green, or blue) proteins with UL50 (right graphs). For B and C, curves were drawn using nonlinear regression analysis in GraphPad Prism 9 for MacOS. Error bars represent standard deviations from three independent experiments. Where no error bars are seen, the standard deviations were too small to be visible.

GK1 and GK2 each contain an acrylamide group that could serve as an electrophilic functional group (“warhead”) for covalent interaction with nucleophilic cysteines on UL50 and/or UL53. To investigate this possibility, we arranged for the synthesis of an analog of GK2, called aGK2 (Fig 5A), in which the acrylamide was replaced with propanamide so that it could no longer covalently interact with cysteines. This analog showed little, if any activity in the HTRF assay for NEC interactions (or in the counterscreen assay) (Fig 5B), consistent with GK2 (and GK1) acting as a covalent inhibitor. We then investigated where GK2 binds to the NEC. Following incubation of the NEC (0.5 nmol) with GK2 (2.5 nmol) for 1h at RT, the sample was analyzed by proteolytic digestion and mass spectrometry. The analysis identified seven cysteines linked to GK2 adducts (Table 1); four from UL50, which has eight cysteines, and three from UL53, which has nine.

**Table 1:** Cysteines labeled with GK2 in UL50 or UL53 identified by Mass Spectrometry.

	Peptide sequence^1^	Labeled cysteine
**UL50**	VTDAGLI**C**K	C35
	NPNYSV**C**DAMLK	C43
	G**C**AVSLCCFVR	C79
	TDTVY**C**VEYLLSYWESR	C54
**UL53**	NFYYGF**C**K	C159
	LHMHVIFENPDVHIP**C**DCITQMLTAAR	C214
	YKELIQEL**C**QSSG	C288

^1^ GK2 labeled cysteines are highlighted in bold

To determine the importance of individual cysteine residues for inhibition of NEC interactions, we engineered and expressed six mutants, each containing a substitution of a cysteine to either serine or asparagine (various substitution mutants of the seventh cysteine, UL50 C43, did not express well). The substitutions had at most slight effects on the affinities of NEC interactions (S2 Table). When tested for susceptibility to inhibition by GK1 or GK2, although UL50 substitutions resulted in modest (~2 to 4-fold) changes in IC_50_, one UL53 substitution, C214S, essentially eliminated inhibition, even at concentrations as high as 50 μM (Fig 5C). Interestingly, the MCMV homolog of UL53 has a serine at the corresponding position [22], which may explain why MCMV NEC interactions are not inhibited by GK1 or GK2 (Fig 2B, bottom). The results taken together strongly suggest that covalent interaction of GK1 or GK2 with UL53 C214 is crucial for inhibition of NEC interactions in vitro.

### Substitution of UL53 cysteine 214 confers resistance to GK1

**Fig 6:**
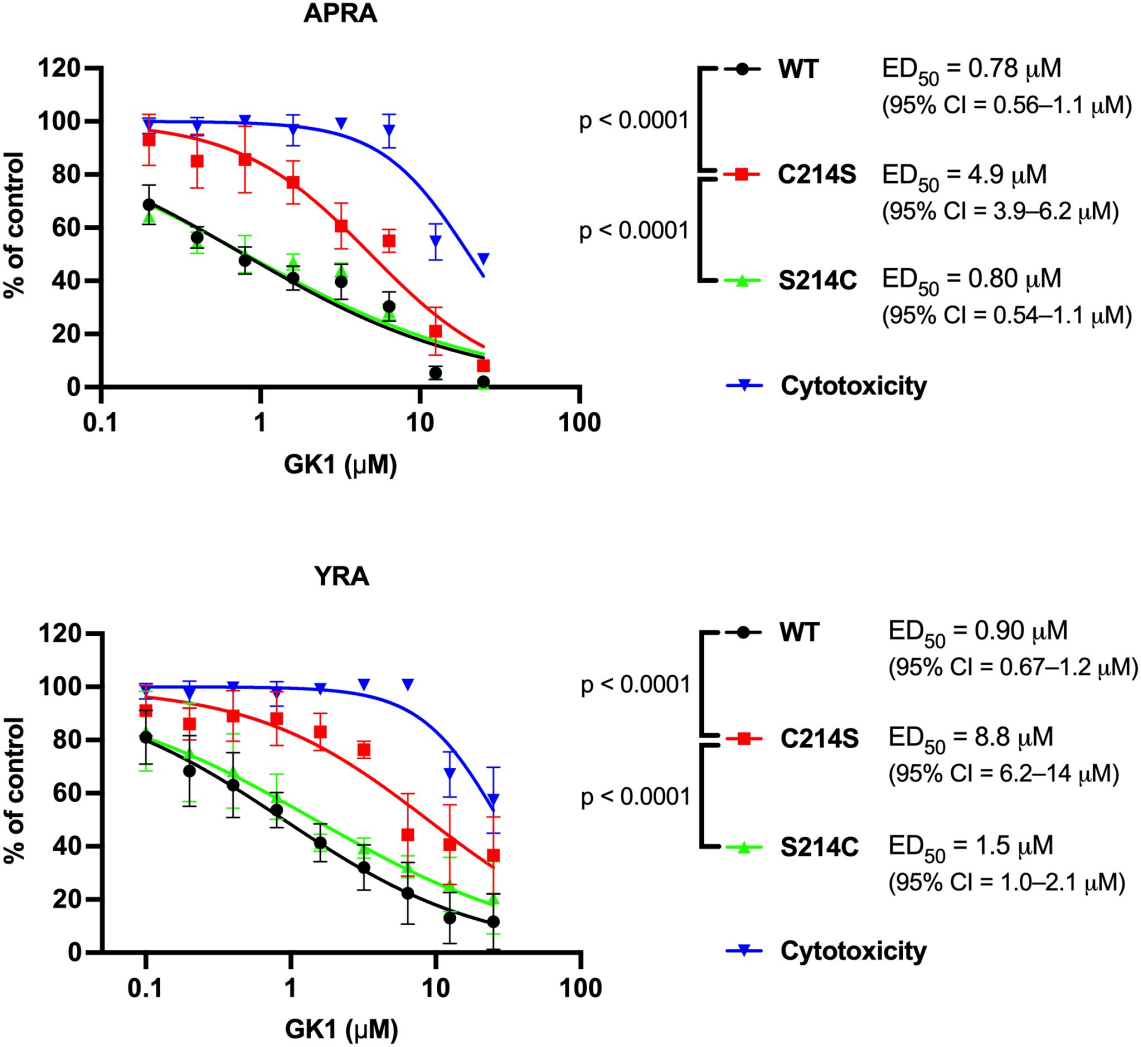
The UL53 C214S substitution confers resistance to GK1. HFF cells were infected at an MOI of 0.02 (top panel, 384-well plate) or an MOI of 1 (bottom panel, 24-well plate) with 53-F BADGFP (WT), the UL53 mutant C214S in that background, or the rescued derivative of the C214S mutant (S214C), and incubated with DMSO vehicle or GK1 at the indicated concentrations. Plaque numbers were measured using automated plaque reduction assays (APRA) at 6 dpi (top) or infected cells were harvested at 5 dpi and titrated in standard yield reduction assays (YRA) (bottom). Cytotoxicity of GK1 was assessed in parallel with each assay of antiviral activity using a WST-1 assay with CC_50_ values of 20 μM (95% confidence interval (CI), 17 to 25 μM) and 27 μM (95% CI, 23 to 37 μM) accompanying the APRA and YRA, respectively. Error bars represent standard deviations from three independent experiments. Where no error bars are seen, the standard deviations were too small to be visible. Curves were fit using nonlinear regression, and ED_50_ values, and the 95% CIs and p values for the difference between the ED_50_’s were calculated using GraphPad Prism 9.5.1 for MacOS. The p values obtained were <0.001 for both assays; these values are below the alpha level of 0.0253 used to correct for two comparisons in each assay, thus keeping the family-wise error rate below 0.05.

We then used bacterial artificial chromosome (BAC) methods to engineer an HCMV mutant in which UL53 cysteine 214 is substituted with serine (C214S) and, as a control, a rescued derivative virus in which the serine was reverted back to cysteine (S214C). Using the automated plaque reduction assay, C214S was substantially resistant to GK1 (6-fold increase in ED_50_), while S214C exhibited susceptibility indistinguishable from the parental WT virus (Fig 6, left). We also tested for resistance using a yield reduction assay in which HFFs were infected with viruses at an MOI of 1 in the presence of varying concentrations of GK1, then harvested at five days post-infection and titrated to determine infectious yield. In this assay, C214S was again substantially resistant to GK1 (9-fold increase in ED_50_) while S214C remained sensitive (Fig 6, right). These ED_50_ differences between C214S and WT and between C214S and S214C were highly significant (Fig 6). In cytotoxicity assays conducted in parallel with both antiviral assays, the CC_50_’s for GK1 were just ~4-fold higher than the ED_50_’s for C214S (Fig 6). C214S had a slightly higher ED_50_ than WT for the less potent analog, GK2, but the p value for this difference was 0.1585 (S5 Fig). We also assessed resistance of the HCMV C214S mutant to two analogs of GK1, GKD4 and GKD6 (S6 Fig). These analogs are intermediate in potency to GK1 and GK2 in both the HTRF and antiviral assays, and C214S similarly exhibited resistance that was intermediate between GK1 and GK2 (~2- and ~3-fold, respectively) and significant (S6–S8 Figs). We conclude that GK1 selectively inhibits HCMV replication largely if not entirely by targeting the NEC.

### Docking of GK1 and GK2 suggests a mode of action

**Fig 7:**
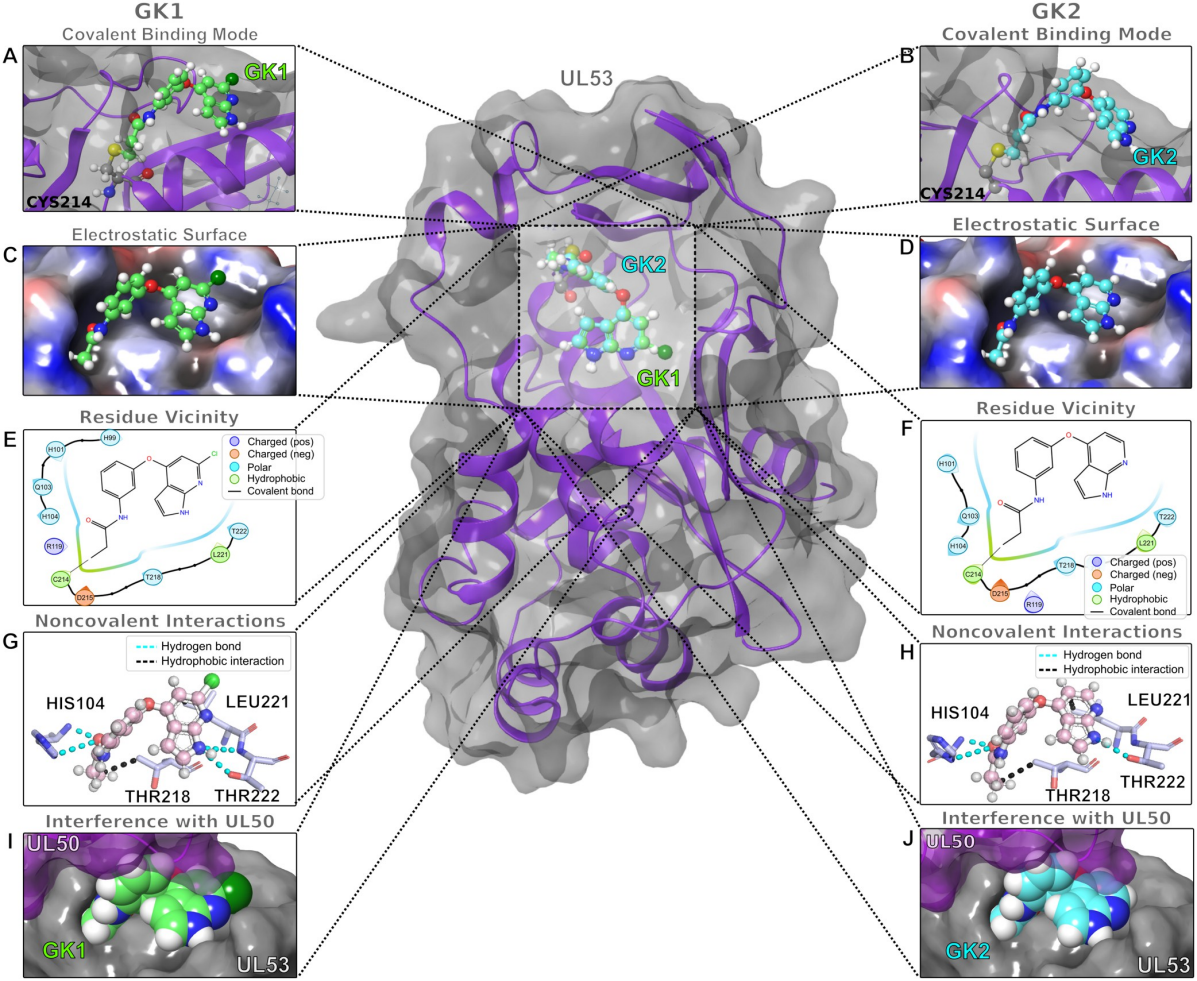
Docking of GK1 and GK2 in the UL53 subunit of the HCMV NEC. The center panel shows the predicted locations (overlaid) of GK1 and GK2 (ball and stick models) on the overall structure of UL53. (A, B) A close-up view of the predicted covalent binding mode of GK1 and GK2 in UL53; (C,D) A close-up view of the electrostatic surfaces predicted to surround GK1 and GK2 in UL53; (E,F) A close-up view of the residues predicted to be close to GK1 and GK2 in UL53. Covalent bonds between C214 and GK1 or GK2 are shown with solid lines. (G,H) A close-up view of the predicted non-covalent interactions between UL53 and GK1 or GK2; GK1 forms six hydrogen bonds with UL53 while GK2 only forms three hydrogen bonds with UL53. (I,H). A close-up view showing the predicted clash between GK1 or GK2 with UL50.

To gain insight into how GK1 and GK2 might block NEC interactions, we prepared the structure of UL53 (PDB ID5DOC; [22]) and these two small molecules for docking, then used Schrödinger’s CovDock [35] to position the molecules following covalent interaction via Michael addition to C214 as described in the Materials and Methods (Fig 7 and S9 Fig, which shows the location of C214 relative to the subunit interfaces on a ribbon diagram of the NEC structure just above the “B” face of UL50, but relatively distant from the “A” face “vise” that interacts with helices of UL53 [22]). GK1 and GK2 are predicted to extend out from the site of covalent attachment within a small pocket lined with a mix of charged, polar, and hydrophobic residues (Fig 7A–7F). Apart from the predicted covalent interaction, Thr218 is predicted to make a hydrophobic interaction with the carbon from the acrylamide of the two compounds that links to the sulfur of C214, while both the side chain and the main chain amide of His104 make hydrogen bonds with the carbonyl from the acrylamide (Fig 7G and 7H). Additionally, both compounds are predicted to make a hydrophobic interaction between the pyridine moiety of the 7-azaindole and the side chain of UL53 Leu221, and hydrogen bond interactions between the NH of the 7-azaindole ring with the main chain amide and side chain of Thr222 (Fig 7G and 7H).

The CovDock docking score of GK1 was -3.97, and the docking score of GK2 was -0.81, predictive of stronger binding of GK1 than GK2, which is consistent with GK1 being more potent than GK2 in the HTRF and antiviral assays. The chlorine of GK1 provides an additional interaction surface with the pocket, particularly Leu221, likely through van der Waals interactions (Fig 7G). Docking scores for GKD4 and GKD6 were similarly predictive of stronger binding to the C214 pocket than GK2, consistent with their greater potencies in the HTRF and/or antiviral assays compared with GK2, and the significant resistance of the viral C214S mutant to these compounds (S6–S8 Figs and S3 Table and S1 Text).

Interestingly, the protrusion of GK1 and GK2 from the pocket would be predicted to interfere with the interaction of UL53 with the “B” face of UL50, and the chlorine of GK1 could contribute to such interference (Fig 7I and 7J). A different docking approach (HADDOCK [36, 37]), despite predicting fewer interactions of GK1 with UL53, nevertheless also predicted that GK1 would clash with UL50 (S10 Fig).

Both docking approaches provide a potential mechanism for how these two compounds disrupt NEC subunit interactions, with CovDock indicating how the chlorine of GK1 may contribute to its increased potency in the HTRF assay and in infected cells.

## Discussion

### A small molecule disruptor of NEC subunit interactions selectively inhibits HCMV

Previous mutational studies have found that single amino acid substitutions in either UL50 or UL53 that strongly disrupt heterodimer formation are lethal for HCMV [12,22,26,38]. Based on these results along with a lack of human homologs of UL50 and UL53, their distinct overall folds, and their unique subunit interface, we hypothesized that a small molecule that could disrupt this interaction would possess selective antiviral activity [12,22,26]. As described here, GK1 and GK2 disrupt HCMV NEC subunit interactions but not those of the MCMV NEC or of the HSV-1 Pol C-terminus and UL42, suggesting biochemical specificity. Moreover, GK1 and GK2 have anti-HCMV activity in HFF cells that appears more potent than their activity against HSV-1 in the same cells, and more potent than their activity in a cytotoxicity assay, suggesting selective antiviral activity, particularly for GK1 whose CC_50_/ED_50_ ratio is 27. However, these kinds of differences, although suggestive, are not sufficient to demonstrate antiviral selectivity, as the WST-1 assay we used, like other cytotoxicity assays, could miss any number of deficiencies in host cell functions. We addressed this issue by identifying an NEC substitution, UL53 C214S, that eliminates detectable inhibition of NEC subunit interactions in vitro by GK1 and confers significant and substantial resistance to GK1 in cell culture when introduced into the HCMV genome. These results imply that GK1’s antiviral potency depends critically on UL53 C214, almost certainly by their covalent interaction, rather than merely depending on inhibition of a host cell function. Thus, GK1, which inhibits UL50-UL53 interactions, acts selectively against HCMV by targeting the NEC, meeting the prediction of the starting hypothesis.

Although the UL53 C214S substitution considerably reduces GK2 inhibition of subunit interactions in vitro, has anti-HCMV activity, and causes localization of UL53 to the nucleoplasm of infected cells, the C214S substitution does not confer significant resistance to GK2. Thus, we have not established that GK2’s anti-HCMV activity is either selective or due to targeting the NEC. There is, nevertheless, a trend towards resistance, particularly at lower concentrations of compound where GK2’s cytotoxicity would be less likely to contribute to an antiviral effect. Thus, it is possible that binding of GK2 to UL53 C214 does contribute to its anti-HCMV activity. However, it is also possible that binding to other cysteines on UL50 or UL53 or on other viral proteins contributes to whatever portion of GK2’s anti-HCMV activity that is due to direct antiviral action rather than due to cytotoxicity.

Although it is clear that most of GK1’s direct antiviral activity requires binding to UL53 C214, it is also possible that some of that activity involves binding to other NEC cysteines. We note that analog GKD9’s activity in the HTRF assay does not depend crucially on UL53 C214 (S6 and S7 Figs), with other cysteines being more important, yet is almost as potent for antiviral activity as GK1, with a higher CC_50_/ED_50_ ratio. Thus, other cysteines on the NEC might serve as useful targets for antiviral activity. It is also highly likely that binding to cysteines of host proteins contributes to cytotoxicity of GK1 and its analogs.

As GK1 and GK2 were originally synthesized as potential protein kinase inhibitors, it is conceivable that the viral UL97 protein kinase or host protein kinases could be targets of their activities. However, two of the analogs (a2GK2 and GKD2) that we synthesized to lack important kinase inhibitor moieties retained antiviral potency but were, if anything more cytotoxic than GK1 or GK2 (S6 Fig and S3 Table and S1 Text). Regardless, GK1 clearly inhibits HCMV replication by targeting the NEC.

### Comparisons with previous screens and compounds

Two prior publications reported the development of assays for screens of inhibitors of HCMV NEC subunit interactions [29,30], but no details were provided regarding their robustness and reproducibility (e.g., Z’ factors). One of these studies, describing an ELISA assay-based screen of pools from a small library, and a subsequent paper reported the interesting finding that merbromin (also known as mercurochrome, a topical antiseptic that is no longer marketed due to toxicity concerns), is an inhibitor of NEC interactions in vitro and in cells, and is more potent for anti-HCMV activity than for cytotoxicity [30,31].

Merbromin is a fluorescein derivative that interacts through its mercury with cysteines in many proteins, and has been widely used as a fluorochrome and a histological dye to stain biological specimens. Thus, the fluorescent and chromogenic properties of merbromin, particularly when bound to proteins, could cause positive or negative interference with various assays. In our screen, we observed <33% inhibition of FRET (acceptor emission) in four different tests (S1 Table) at 13.3 μM merbromin, a concentration ~200-fold and ~10-fold above the two reported IC_50_’s [30,31]. Interestingly, merbromin suppressed donor emissions more strongly than acceptor emissions in our HTRF assay (S1 Table), consistent with assay interference and perhaps explaining its failure to score as a hit.

In a fluorescence-based neutral red assay, at doses corresponding to those exerting anti-HCMV activity, merbromin substantially increased fluorescent signal above that with vehicle alone [30], raising the possibility that its cytotoxicity might have been underestimated. Additionally, interference may conceivably have influenced horseradish peroxidase-based ELISA and co-immunoprecipitation assays used to measure merbromin effects on NEC interactions, particularly the latter assays where increasing concentrations of merbromin resulted in decreasing detection of UL53 on Western blots [30,31]. That effect might also be due to merbromin adducts resulting in loss of UL53 solubility, consistent with the relatively large, discrete intranuclear UL53-containing dots observed in merbromin-treated infected cells that differ from what we observed in GK1-treated or UL53 L79A-infected cells [30,31].

Among other concerns regarding merbromin’s effects on HCMV and the NEC are possible impurities, which could include free mercury and other mercury containing compounds, and various fluorophores and chromogens. The purity of merbromin was not stated in one paper [30] and in the other study [31], based on the catalog number provided, was only practical grade. Nevertheless, given that GK1 also covalently interacts with cysteines, it seems possible that merbromin could act similarly, which might indicate that the NEC is particularly vulnerable to covalent adducts([30]; also see next paragraph). However, in our screen, numerous warhead-containing compounds, including two with a scaffold similar to GK1’s (S11 Fig and S1 Text), did not score as hits (the smaller of the two showed no inhibition, and the larger only 19% inhibition of HTRF signal). Similarly, several warhead-containing analogs of GK1 that we synthesized had no detectable activity in our HTRF assay (S6 Fig and S3 Table). Thus, it is clear that various chemical features other than the warhead are crucial for NEC disruption activity (also see next paragraph). We note that some of these GK1 analogs showed anti-HCMV activity, but with CC_50_/ED_50_ ratios of only 5 or less (the ratio for GK2 was 5.4), suggesting low if any selectivity (S6 Fig and S3 Table).

While our report was in a near-final draft, a publication appeared reporting on the activities of ~20 warhead-containing compounds in mainly cell-based assays of disruption of NEC subunit interactions and HCMV replication [39]. Among these compounds was the tyrosine kinase inhibitor, ibrutinib [39]. Like GK1 and GK2, ibrutinib and some of the other compounds caused modest localization of UL53 to the nucleoplasm in HCMV-infected cells, but only ibrutinib and two other compounds tested appeared to meaningfully disrupt UL53-UL50 interactions in other cell-based assays. Of these three, only the one with the least effect had a CC_50_/ED_50_ value >5 [39]. Notably, in the only in vitro assay of NEC subunit interactions reported, which was a co-IP assay in lysates of transfected cells, ibrutinib showed little if any activity. This negative result is consistent with ibrutinib not registering as a hit from five different wells in our screen at assay concentrations up to 50 μM (S4 Table). Ibrutinib, which exhibited a CC_50_/ED_50_ value of only 3.8, showed decreased antiviral potency in a qPCR-based assay of genome replication (rather than an assay of infectious virus) under conditions of low expression of UL50 and UL53. This result was interpreted as confirmation of targeting of the NEC [39]. This experiment was creative and used sophisticated methods, but resistance to inhibitors due to changes in target expression usually entails increased rather than decreased target levels. Why ibrutinib would show a different behavior was not addressed. Additionally, the experiment measured supernatant viral genomes rather than infectious virus, and at the two-week time point used, there were evidently roughly only as many genomes in the supernatant as were inoculated. It is difficult to know how many initially infected cells would lyse under these conditions and release genomes into the medium. If so and if ibrutinib affects a step in viral infection after DNA synthesis, then it might be expected to show reduced potency regardless of whether it targets the NEC. Studies using sufficiently pure compounds and resistant HCMV mutants, if they can be isolated, may determine whether merbromin and ibrutinib specifically target the NEC for selective anti-HCMV activity.

### Mechanism of GK1 inhibition

We had anticipated that our screen might yield a hit that directly blocked the interaction of UL53 helices between the fixed and moveable jaws of the UL50 “vise” [12,22,26]. Although GK2 bound to a UL50 residue (C54) on the fixed jaw of the vise, its substitution had little effect on inhibition of NEC subunit interactions. Instead, UL53 C214, rather distant from the site of interaction, turned out to be crucial for both inhibition of NEC subunit interactions by GK1 and GK2 and the antiviral activity of GK1. Docking studies suggest that GK1 bound to C214 would extend through a pocket where its 7-azaindole could sterically hinder the UL50-UL53 interaction. A parsimonious interpretation of our results is that GK1’s antiviral activity entails this mechanism.

Consistent with that interpretation, we found that at doses that reduce viral plaque formation by ≥5-fold, GK1 induces localization of UL53 to the nucleoplasm. However, that effect was rather modest, especially compared to the effect of the UL53 L79A substitution. As mentioned in the Results, a simple explanation for this observation is that at the concentration used (10 μM), GK1 is much less efficacious than the L79A substitution for disrupting subunit interactions. A potentially related observation is that GK1’s antiviral ED_50_ is substantially (~7-fold) lower than its IC_50_ in the HTRF assay. Although comparing potencies in such different assays may be naïve, an intriguing possibility that would explain both observations is that GK1 needs only to disrupt a small fraction of NECs to exert antiviral activity. This would be akin to how certain ligands can elicit half-maximal physiological responses at doses less than their affinities for their receptors when there are substantially more receptors present than needed for the response (“spare receptors”). One of many possible scenarios for this would be GK1 exerting antiviral effects by preventing the association of only newly synthesized NEC subunits.

A second possible interpretation of our results is that although GK1 disrupts subunit interactions important for heterodimerization in vitro, in infected cells other interactions such as ones that stabilize oligomerization of heterodimers into hexamers, hexamer-hexamer interactions at the inner nuclear membrane, and/or interactions with components of the inner nuclear membrane might retain most UL53 at that location despite disruption of heterodimerization by GK1. We note that, with the caveats mentioned above among others, merbromin has been reported to disrupt oligomerization of the NEC and cause more thorough localization of UL53 to the nucleoplasm [31].

A third possible explanation is that GK1 binding to UL53 C214 has effects aside from disruption of NEC heterodimerization. This could explain the ~7-fold more potent activity in the antiviral assay relative to the HTRF assay mentioned above. We also note that although GK1 clearly inhibits NEC subunit interactions in a co-immunoprecipitation assay, it appeared to show slightly less inhibition than in the HTRF assay at the concentrations tested. A variety of technical issues may account for these apparent discrepancies, but it is possible that binding of GK1 to C214 alters UL53 so that it reduces NEC function(s) independent of its effects on subunit interactions. The location of the GK1 binding site is not near where UL53 makes contacts with other heterodimers in crystals containing the HCMV NEC with hexameric packing [25], but binding to that site could conceivably affect those or hexamer-hexamer interactions allosterically, affect hexamer to pentamer transitions during budding [8,13,40], affect interactions with NEC binding partners, and/or inhibit other functions. At least at 10 μM, GK1 did not have a major effect on the NEC-dependent distortion of nuclear shape that also requires the NEC binding partner, UL97 [16,41], although that may be more a reflection of limited efficacy at that dose than something more specific. Further investigation is needed to distinguish among these and other mechanistic possibilities.

### Prospects for development of NEC inhibitors into antiviral drugs

GK1 is a <500 molecular weight, cell-permeable molecule with fewer than five hydrogen bond donors and fewer than ten hydrogen bond acceptors. It shows clear anti-HCMV selectivity with high nanomolar potency. Its properties as a covalent inhibitor permitted the identification of UL53 C214 as being crucial for its activity in vitro and the subsequent construction of a resistant viral mutant for confirmation of that target in infected cells and demonstration of its selectivity. However, its potency is not high by modern standards, its selectivity is not as great as that of approved anti-HCMV drugs or enough to avoid toxicities at concentrations required to achieve sufficient antiviral efficacy [4], and, as a covalent inhibitor that labels multiple cysteines in the HCMV NEC, its biochemical specificity is suspect.

To see if we could improve upon the potency and selectivity of GK1, we synthesized a small collection of analogs of GK1 (S6 Fig), which we tested for inhibition of NEC subunit interactions in vitro using the HTRF assay, and for antiviral activity and cytotoxicity (S1 Text and S3 Table and S7 and S8 Figs). We identified three compounds that showed reasonable activities in both in vitro and cell-based assays, and the C214S mutant was modestly but significantly resistant to two of them (GKD4 and GKD6; the third GKD9 showed reduced cytotoxicity but was only modestly affected by the UL53 C214S substitution in the HTRF assay (S3 Table and S7 and S8 Figs)). Although these compounds provide some information regarding structure-activity relationships, none was more potent than GK1 (see S1 Text). Regardless, there remain numerous modifications of GK1 to explore.

In summary, GK1 is a selective antiviral compound with an interesting mechanism of inhibition of NEC subunit interactions. Studies using this compound could shed further light on NEC biochemistry and, together with the C214S mutant and rescued derivative, it may be useful for studying aspects of NEC function in infected cells. GK1 has several properties that recommend it as a starting point for developing a useful anti-HCMV drug. Despite the substantial amount of work that lies ahead, insights gained from the analogs synthesized thus far, coupled with the potential for design informed by docking studies or future structural investigations, hold considerable promise for advancing towards that objective.

## Material and methods

### Protein engineering and preparation

Primers used for engineering new plasmid constructs are listed in S5 Table.

The engineering of plasmid pET15bUL50^1-169^ for expression of N-terminal His-tagged HCMV UL50 (residues 1–169) and plasmid pGEX6P- UL53^50-292^ has been previously described [22]. To engineer Myc-tagged HCMV UL53, plasmid pGEX6P-UL53^50-292^ was modified using the QuikChange Site-Directed Mutagenesis kit (Stratagene) to insert a Myc tag followed by a 13-residue glycine-serine (GGGGSGGGGSGGG) linker following the GST sequence on pGEX6P-UL53^50-292^.

Plasmids were transformed into E.Coli BL21 (DE3) RIPL cells (Stratagene) and expressed following induction using 0.3 mM isopropyl-beta-D-thiogalactopyranoside (IPTG; Sigma-Aldrich) as described [22]. The His-tagged UL50 and GST-Myc-tagged UL53 constructs were purified using nickel Sepharose and a step gradient of imidazole (Sigma-Aldrich), and glutathione-agarose (GE Healthcare Life Sciences) with on-column digestion to remove GST using PreScission protease (Genway Biotech), respectively, followed by size exclusion chromatography as described [22]. Complete cleavage and protein purity were confirmed using SDS-polyacrylamide gels. To form the HCMV NEC, Myc-tagged UL53 was incubated with His-tagged UL50 overnight at 4°C, and the complexed proteins purified using size-exclusion chromatography on a Superdex S200 10/300 GL column (GE) as described [22].

An E56A substitution was introduced into the pET15bUL50^1-169^ plasmid using the QuikChange Site-Directed Mutagenesis kit (Stratagene) and the protein was expressed in E. coli as previously described for His-tagged UL50 [22]. To generate untagged UL50 and untagged UL50 E56A, nickel Sepharose-eluted His-tagged UL50 and His-tagged UL50 E56A proteins were each treated with thrombin (500 units/mL; BioPharm Laboratories). SDS-polyacrylamide gel analysis confirmed complete His-tag cleavage and protein purity, and the untagged proteins were further purified using size-exclusion chromatography. To generate untagged UL53, the pGEX6P-UL53^50-292^ plasmid was transformed into E.Coli BL21 (DE3) RIPL cells (Stratagene) and expressed following induction using 0.3 mM IPTG (Sigma-Aldrich) as described [22]. The protein was purified using glutathione-agarose (GE Healthcare Life Sciences) and the GST-tag removed with on-column digestion using PreScission protease (Genway Biotech), followed by size exclusion chromatography as described [22].

Three His-UL50 mutants (C54S, C35N and C79S) and three Myc-UL53 mutants (C214S, C159S and C288S) were engineered using the Q5 site-directed mutagenesis kit (New England BioLabs Inc.) by amplification of the pET15b-UL50^1-169^ plasmid or pGEX6P-Myc-UL53^50-292^ plasmid, respectively. The mutant proteins were prepared using the same methods as those for His-UL50 or Myc-UL53.

For the Pol peptide (P30) construct for the counterscreen, Pol C-terminal peptide sequences (residues 1200–1235) from pGEX-PD3 [42] were cloned using the InFusion Advantage PCR Cloning Kit (Clontech) into the modified pGEX6p plasmid containing the Myc tag described above, and expressed and purified as described for Myc-UL53. For the UL42 construct for the counterscreen, a plasmid (kindly provided by Purba Mukherjee of the Hogle and Coen laboratories) containing UL42 residues 1–340 [43], N-terminally tagged with ten residues of histidine fused to SUMO by cloning into pTG257 [44] (kindly provided by Joseph Loparo); was expressed in *E*.*coli* strain pLysS BL21 grown in the presence of 100 μg/mL ampicillin and 17 μg/mL chloramphenicol. IPTG (Sigma-Aldrich) was added at a concentration of 0.5 mM for overnight induction at 20°C. The proteins were purified and the complex (Myc-P30:His-SUMO-UL42) formed and purified as described for the HCMV NEC proteins.

To generate His-tagged MCMV M50, M50 residues (1–168) were cloned into a pET15 expression vector using the NdeI and EcoRI restriction sites. For MCMV M53, M53 residues (103 to 333) were cloned into the pGEX6p plasmid modified to include a Myc tag and glycine-serine linker described above using the InFusion Advantage PCR Cloning Kit (Clontech). The proteins were expressed, purified, and the complex (Myc-M53:His-M50) formed as for the NEC as described above. Untagged M50 protein was produced by purifying His-tagged M50 using nickel Sepharose and a step gradient of imidazole (Sigma-Aldrich), followed by cleavage with thrombin (500 units/mL; BioPharm Laboratories) and analyzed for complete cleavage and purity using SDS-polyacrylamide gels.

Proteins were quantified using either by measuring absorbance at 280 nm with a Nanodrop (ThermoFisher) or using a Bradford assay (BIO-RAD).

### Chemical compounds

Chemical libraries for screening were made available by the ICCB-Longwood (S1 Appendix). GK1 was purchased from WuXi AppTec as were all other related compounds except GK2 and aGK2, which were purchased from Hoayuan ChemExpress Co, Ltd., and a2GK2, which was prepared by Frederic Feru at the DFCI Medicinal Chemistry Core. All compounds were at least 95% pure (quality control information in S2 Appendix).

### Homogeneous time resolved fluorescence (HTRF) assay and high throughput screening

All HTRF assays were conducted at the ICCB-Longwood screening facility at Harvard Medical School. To determine and establish the specificity of the NEC subunit interactions in the HTRF assays, signal from the NEC was compared to that treated with either untagged UL50 (positive control) or untagged UL50 E56A (negative control). Ten μL of 0.6 μM HCMV NEC protein in assay buffer (50 mM Hepes (pH 7.5; Sigma), 200 mM NaCl (Sigma) and 1mM DTT (Sigma)), or 5 μL of 1.2 μM NEC to which either 5 μL of 12 μM untagged UL50 or 12 μM untagged UL50 E56A were added to each well of low-volume, white 384-well plates (Corning) using a Multidrop Combi nL Reagent Dispenser (ThermoFisher Scientific). Ten μL of a stock of anti-His antibody coupled to the donor terbium cryptate fluorophore (Cisbio) and anti-Myc antibody coupled to the acceptor fluorophore d2 (Cisbio), at a concentration of 8 nM and 80 nM respectively were then added. Plates were centrifuged using a Heraeus Multifuge X3 FR centrifuge (ThermoFisher) at 1,000 RPM for 30 seconds, and incubated overnight at 4°C, then for one hour at room temperature. Fluorescence signals were measured following excitation at 340 nm using an EnVision plate reader (PerkinElmer) and HTRF values were calculated using the ratiometric method (ratio of acceptor to donor emission, 665 and 620 nm, respectively, x 10,000) according to the manufacturer’s guidance (Cisbio). The signal to background ratios of the HTRF from the NEC alone and from the NEC incubated with either UL50 or UL50 E56A were calculated.

To determine the robustness of the HTRF assays, 128 replicates each of tagged NEC with DMSO (to a final concentration of 0.5%), or NEC with either untagged UL50 or untagged UL53 were dispensed into a 384 well plate. The volumes and concentrations of the NEC, the NEC and untagged proteins, and the HTRF reagent solutions were as described above. A stock solution of combined donor and acceptor was then added to each well and the plates centrifuged, followed by incubation and fluorescence measurements as described above. The HTRF ratios and the Z’ factor were calculated.

A pilot screen was then performed using Biomol 4 compound plate of 640 compounds with the NEC and a row of 16 wells each of NEC with untagged UL50 as the positive control, and NEC with DMSO (to a final concentration of 0.5%) as the negative control. The volumes and concentration used were as described above. The plates were centrifuged at 1,000 rpm for 30 seconds as described above. Pin transfer of 100 nL of each compound in DMSO was performed using a 384 well pin array and a compound transfer robot (Epson) and the plates were again centrifuged as described above. The plates were then incubated at room temperature for 1 hr after which the HTRF reagent solutions were dispensed into the plates as described above. After an additional centrifugation, the plates were incubated overnight at 4°C, followed by one hour at room temperature, then the fluorescence measured and HTRF ratios calculated as described above. The percent inhibition for each sample well and a Z’ factor of 0.67 and 0.65 for each plate were calculated.

To screen compound libraries, the addition of 10 μL of NEC for the sample wells, and 5 μL of NEC with 5 μL of untagged UL50 for the positive control was performed as described above. 100 nL of each compound in DMSO was delivered into corresponding wells of two replicate plates using a 384 well pin array and a compound transfer robot (Epson). Compound libraries are shown in S1 Appendix. Stocks of compounds were 5 mg/ml or 10 mM for commercial compounds; 2 mg/ml or 0.4–10 mM for known bioactive compounds; 0.08–10 mM for academic collections; and 15 mg/ml for natural product extracts. As an example, delivery of 100 nL of a 10 mM compound resulted in a 50 μM final concentration after addition of antibodies. Addition of DMSO to the negative controls was performed simultaneously during the compound pin transfer as each compound plate included a row of wells filled with DMSO as controls. After addition of controls or test substances, the plates were centrifuged as described above and incubated at room temperature (RT) for 1h. Anti-6His-Tb cryptate and anti-cmyc-d2 were then added, and the plates incubated and read as above.

For the counterscreen HTRF assays with the HSV DNA polymerase subunit UL42 and the C-terminus of HSV UL30 (P30) or with the MCMV NEC, the same protocol as that for HCMV NEC was used except 1μL 30 μM MBP-UL42Δ340 [43] (gift from Sandrine Boissel, Harvard Medical School) or 1μL 30 μM untagged M50 was added as the positive control for UL42-UL30 or MCMV NEC, respectively.

For dose-response tests with cherry-picked hits or GK compounds, 100 nL compound at various concentrations were dispensed using a HP D300e digital dispenser (Hewlett Packard). The ratio of the FRET signal to the donor emission signal at each concentration of the compound was normalized to DMSO controls. The 50% inhibitory concentration (IC_50_) for each compound was calculated from these dose-response curves by nonlinear regression using GraphPad Prism 9 for MacOS. *K*_d_ values for the interactions of wild type and mutant NEC subunits were calculated using the method of Newton et al. [45].

### Co-immunoprecipitation assay

0.3 μM purified HCMV NEC in 50 mM Hepes (pH 7.5) and 200 mM NaCl were incubated with DMSO or with 10 μM or 20 μM GK1 at RT for 30 min followed by incubation at 4°C overnight. For immunoprecipitation of His-UL50, each sample was incubated with 25 μL Dynabeads His-Tag magnetic beads (Invitrogen) on a roller for 10 min at RT, followed by four washes and then elution from the beads using the manufacturer’s guidance. For immunoprecipitation of Myc-UL53, each sample was incubated with 50 μL Pierce anti-c-Myc magnetic beads (ThermoFisher Scientific) on a roller for 30 min at RT. Then the beads were washed three times and the proteins were eluted from the beads with 50 μL 0.5mg/mL Pierce c-Myc peptide (ThermoFisher Scientific) following the manufacturer’s guidance. To test the binding specificity of the beads, 0.3 μM purified HCMV His-UL50 alone or HCMV Myc-UL53 alone was incubated with His-tag magnetic beads or Myc-tag magnetic beads, and the corresponding immunoprecipitation was performed with the same protocols described above. Aliquots of the input samples (2.5% of what was incubated with each type of magnetic beads) as well as proteins eluted from the beads (20% of each eluate) were subjected to SDS-PAGE, followed by Western blot analysis using a mouse anti-His antibody (1:1,000, Invitrogen) and a mouse anti-Myc antibody (1:1,000, Invitrogen). Horseradish peroxide (HRP) conjugated anti-mouse antibody (1:1,000, Southern Biotech) was used as the second antibody. Protein bands were detected using chemiluminescence solution (Pierce).

### Cells and viruses

Human foreskin fibroblast (HFF) cells (Hs27; American Type Culture Collection) were maintained as previously described [46]. HCMV 53-F BADGFP, which expresses GFP under the control of the major HCMV immediate early promoter and FLAG-tagged UL53 [16], served as the WT virus. The HCMV mutant with an L79A substitution in UL53 was generated by introducing the L79A substitution into the 53-F pBADGFP bacmid using previously described primers and methods [22], then electroporating the resulting bacmid into complementing UL53-expressing cells, harvesting and concentrating virus, and titrating on the complementing cells as described [22,47]. Virus carrying *UL53* mutation C214S was generated by introducing the mutation into the bacmid 53-F BADGFP [16] as described previously by using the PCR primers listed in S3 Table and the two-step red recombination method [48,49]. To construct the rescued derivative BAC-S214C, wild type sequences were restored to the mutant BAC using the PCR primers listed in S3 Table and the same methodology. Virus was titrated using standard plaque assays [26].

### Antiviral activity and cytotoxicity assays

Antiviral activity was assessed using three methods—automated plaque reduction assays, standard plaque reduction assays, and yield reduction assays. For automated plaque reduction assays, 2400 HFF cells HFF (ATCC, clone Hs27) in 20 μL were seeded into each well of a 384-well plate (Corning). Then ~50 PFU in 10 μL of 53-F BADGFP [16] or the GFP-expressing viruses constructed in this study were dispensed into each well, followed by centrifugation at 1000 rpm for 10 min using a Heraeus X3 FR centrifuge (ThermoFisher) to promote infection. The plate was kept at 37°C for 4h and 30 μL of various concentrations of compounds (0.2 ~ 50 μM) in Dulbecco’s Modified Eagle Medium (DMEM, Gibco) containing 1% DMSO or a DMSO control (1% in DMEM) were added, with 32 replicate wells for each concentration. At 6 dpi, the plates were imaged using a high content instrument (Acumen, STP Labtech), which can distinguish clusters (plaques) of GFP-expressing (infected) cells from singly infected cells by size. Plaque numbers at each concentration of the compound were normalized to those in DMSO control wells, and the 50% inhibitory dose (ED_50_) for each compound was calculated using nonlinear regression in GraphPad Prism 9 or 9.5 for MacOS.

Standard plaque reduction assays used to assess the antiviral activity of GK1 and GK2 against HSV in HFF cells were performed as described [50] with minor modifications. Briefly, 1x10^5^ HFF cells in each well of a 24-well plate were infected with ~ 100 PFU/well of HSV-1 strain KOS. After 2h, the inocula was removed and DMEM containing methylcellulose, 1% DMSO, and various concentrations of GK1 or GK2 was added. After two days, plaques were stained with crystal violet and counted. ED_50_’s for GK1 or GK2 were calculated as described above.

Yield reduction assays, used to assess the antiviral activity of GK1 against WT HCMV, HCMV-UL53 C214S and HCMV UL53 S214C, were performed as described [51] with modifications: 1x10^5^ HFF cells in each well of a 24-well plate were infected with virus (WT HCMV, HCMV UL53 C214S or HCMV UL53 S214C) at an MOI of 2.5. After 2h infection at 37°C, the virus was removed and the cells were incubated with 1 mL of fresh DMEM containing 1% DMSO, and either no GK1 or GK1 at various concentrations. The supernatants were harvested at 5 dpi and the titers were determined using standard plaque assays. ED_50_’s for GK1 were calculated as described above.

Cytotoxicities of various compounds were determined using a WST-1 assay that measures metabolic activity (Roche), and CC_50_’s were calculated as described previously [52].

### Western blot analysis of protein expression in infected cells

HFF cells were mock infected or infected with 53-F BADGFP at an MOI of 1 and then incubated in medium containing 1% DMSO and either no drug, 10 μM GK1, or 10 μM GK2 for 3 days. Then the cells were washed with PBS and lysed in 2x Laemmli buffer (Bio-Rad) containing 5% (v/v) 2-mercaptoethanol. Total protein was applied to SDS-PAGE and transferred to a PVDF membrane (Millipore). The membranes were blocked in 5% milk for 1h at RT, followed by the addition of specific primary antibodies overnight at 4°C. Primary antibodies included mouse anti-IE1/IE2 monoclonal antibody (1:1,000, Abcam), mouse anti-UL57 monoclonal antibody (1:1,000, Virusys), rabbit anti-UL50 antibody (1:500, [16]), mouse anti-FLAG M2 (1:1,000, Sigma), mouse anti-pp28 antibody (1:1,000, Virusys), and mouse anti-beta actin antibody (1:5,000, Sigma). Horseradish peroxide (HRP) conjugated anti-rabbit antibody (1:1,000, Southern Biotech) and HRP conjugated anti-mouse antibody (1:1,000, Southern Biotech) were used as the secondary antibodies. Protein bands were detected using chemiluminescence solution (Pierce).

### Immunofluorescence

Immunofluorescence was performed as described previously with some changes [16]. Briefly, 1x10^5^ HFF cells seeded on glass coverslips in a 24-well plate were infected with HCMV 53F BADGFP [16] or HCMV 53F UL53 L79A at MOI 1. After 4h absorption, the inoculum was removed. 1mL of DMEM containing 1% DMSO, and either no compound or 10 μM GK1 or GK2 was added to infected cells. After three days of incubation, cells were washed with PBS twice and fixed with 4% formaldehyde in PBS for 15 min at RT. The fixed cells were permeabilized with 0.1% Triton X-100 in PBS for 10 min followed by two washes with PBS and then blocking with 1% bovine serum albumin in PBS overnight at 4 °C. Rabbit anti-UL50 [16], mouse anti-FLAG M2 (Sigma) or mouse anti-pp28 (Virusys) were applied as primary antibodies and incubated at 4°C overnight. After removing primary antibodies, the cells were washed with PBS and incubated with secondary antibodies Alex Fluor 647 chicken anti-mouse (ThermoFisher) and Alex Fluor 568 donkey anti-rabbit (ThermoFisher). Cell nuclei were labeled with DAPI (ThermoFisher). Coverslips were mounted with ProLong Glass Antifade Mountant (ThermoFisher) on microscope slides. Images were recorded with a Nikon Ti inverted fluorescence microscope equipped with a Hamamatsu ORCA-R2 cooled CCD camera (confocal: 6.45 μm^2^ photodiode; widefield: 6.45 μm^2^ photodiode) at the Nikon Imaging Center (Harvard Medical School). Three laser lines (488 nm, 561nm and 642 nm) were selected; selection of laser lines was controlled by AOTF. The images were acquired with MetaMorph image acquisition software and processed with ImageJ.

### Mass spectrometry

UL50 or UL53 protein (10 μg) was treated with DMSO or a 5 fold molar excess of GK2 for 1 hr at room temperature. After reactions, proteins were denatured with Rapigest (0.1% final concentration, Waters Corp), reduced (10 mM DTT, 56°C for 30 minutes), alkylated (22.5 mM iodoacetamide, 30 minutes, room temperature, protected from light), and digested with trypsin overnight at 37°C. Peptides were desalted by SP3 [53] using an equimolar mixture of hydrophilic and hydrophobic Sera-mag beads and eluted with 25% acetonitrile and dried by vacuum centrifugation. Peptides were reconstituted in 50% acetonitrile/1% formic acid with 100 mM ammonium acetate and analyzed by CE-MS using a ZipChip CE-MS instrument coupled to an Orbitrap mass spectrometer (QExactive HF, San Jose, CA). Peptides were loaded for 30 seconds and separation performed at 500 V/cm for 10 minutes using an HR chip (22 cm separation channel) with a background electrolyte composed of 1% formic acid in 50% acetonitrile. Pressure assist was utilized and started at 1 minute. The mass spectrometer was operated in data dependent mode and subjected the 5 most abundant ions in each MS scan (60k resolution, 3E6 target) to MS/MS (15k resolution, 1E5 target, 100 ms max inject time). Dynamic exclusion was enabled with an exclusion time of 5 seconds. MS/MS data were extracted to.mgf using mulitplierz scripts [54] and searched against a custom database containing target protein sequences using Mascot version 2.6. Search parameters specified fixed carbamidomethylation of cysteine, and variable oxidation (methionine) and GK2 modification (cysteine). Precursor mass tolerance was set to 10 ppm and product ion tolerance was 25 mmu. Labeled peptide MS/MS spectra were validated with mzStudio software [55].

### Docking

For the molecular docking to HCMV UL53 shown in Fig 7, the structure of UL53 that was used [22] was obtained from the Protein Data Bank, PDB ID 5DOC. The protein structure was prepared for docking using Schrödinger’s Maestro [56, 57]. Missing hydrogens were added, bond orders and metal coordination bonds assigned, and the protein protonated at physiological pH. The ligands were prepared by computing the minimum potential energy conformation with Schrödinger’s LigPrep [58], by assigning bond orders and protonation states at physiological pH. Schrödinger’s CovDock [35] was used to dock the compounds covalently to residue C214 of UL53 via a Michael addition reaction. The centroid of residue C214 was used as the center of the docking box. The docking box size was set to the option "Dock ligands similar in size to workspace ligand", and CovDock docking scores were calculated. The PLIP (Protein-Ligand Interaction Profiler) was used to analyze the noncovalent interactions between the ligands and UL53 [59, 60]. The binding mode of UL50 in Fig 7I and 7J was obtained by aligning the UL53 structure of the UL50-UL53 complex structure of PDB ID 5DOB [22] with the UL53-ligand complex structures obtained via molecular docking.

For the docking of GK1 to UL53 shown in S9 Fig, we utilized the HADDOCK docking software [36,37,61], which integrates experimental data as restraints along with traditional energetics and shape complementarity. Docking experiments were carried out using the HADDOCK2.2 webserver with the guru interface. The crystal structure of the human cytomegalovirus UL53 subunit of the NEC (PDB code: 5DOC), specifically chain B, was used as the starting point. Before docking, the structure was prepared using PDB Tools. For the covalent bond, distance restraints were set to 1.8Å ± 0.1Å, representing the average length of a C-S bond. Additional constraints were applied to the Zn^2+^ ion to ensure proper ion coordination. The loop adjacent to the covalently bound cysteine residue (amino acids EDNRILP) was allowed full flexibility. The HADDOCK docking protocol involved three main stages: (1) randomization of orientations and rigid-body minimization, (2) semi-flexible simulated annealing in torsion angle space, and (3) refinement in Cartesian space with explicit TIP3P solvent. The models were subjected to a short molecular dynamics simulation at 300°K during the refinement stage. Finally, the models were automatically clustered based on a similarity measure, namely RMSD. Results were visualized with PyMOL Open Source (The PyMOL Molecular Graphics System, Version 2.3.0 Schrödinger, LLC.)

## Supporting information

S1 Fig Purification of HCMV NEC containing His-UL50 and Myc-UL53 with size-exclusion chromatography. Top: HCMV His-UL50 and Myc-UL53 were purified individually using affinity chromatography and then chromatographed on a size-exclusion column at a ratio of 1 Myc-UL53 to 2 His-UL50 (green trace). A trace of His-UL50 alone is shown for comparison (blue trace). Bottom: Aliquots of fractions corresponding to the faster eluting peak following chromatography of the protein mixture were resolved using SDS-PAGE and the indicated fractions were pooled for use in HTRF assays.
(PDF)

S2 Fig HTRF assays for inhibition of HSV-1 UL30 peptide-UL42 and MCMV M50-M53 interactions. C-terminal 36 residues of HSV-1 UL30 (P30) fused to Myc-tagged GST was mixed with His-tagged HSV-1 UL42 (left graph) or His-tagged MCMV M50 was mixed with Myc-tagged MCMV M53 (right graph) and assayed using the same reagents and methods as used for the HTRF assay for HCMV NEC interactions. Untagged P30 or UL42 (left graph) or untagged M50 (right graph) substantially reduced HTRF signal in their respective assays.
(PDF)

S3 Fig GK1 does not interfere with the HTRF assay. The HTRF assay was performed at different concentrations of GK1 either with NEC as a target (red lines) or without protein targets (blue lines). Under conditions where GK1 strongly inhibited HTRF (left graph) or FRET signal at 665 nm from the NEC (right graph), it had little or no effect on HTRF in the absence of protein target.
(PDF)

S4 Fig Effect of GK2 on HCMV protein expression in infected cells. HFF cells were mock infected or infected with WT HCMV at an MOI of 1. Following absorption, medium containing either DMSO or 10 μM GK2 was added. At 72 hpi, cells were lysed, proteins resolved using SDS-PAGE, and the expression of the viral proteins indicated to the left was assessed by immunoblotting. Similar results were obtained in a second independent experiment.
(PDF)

S5 Fig Antiviral activities of GK2 against WT HCMV and mutant HCMV C214S. HFF cells infected with each virus indicated were incubated with GK2 at different concentrations. Automated plaque reduction assays were performed at 6 dpi. Cytotoxicity assays of GK2 were performed in parallel. Error bars represent standard deviations from three independent experiments. Where no error bars are seen, the standard deviations were too small to be visible. Curves were fit using nonlinear regression and ED_50_ values and the 95% confidence intervals (CI) and p value for the difference between the ED_50_’s were calculated using GraphPad Prism 9.5.1 for MacOS.
(PDF)

S6 Fig The chemical structures of analogs of GK1 and GK2. (PDF)

S7 Fig Activities of analogs GKD4, GKD6 and GKD9. (A, left graphs) Dose-response of the inhibitory activities of GKD4, GKD6 and GKD9 against interactions of HCMV UL50-UL53 (black) or the UL30 peptide (P30)-UL42 (counter screen, red) in HTRF assays. (A, right graphs). The antiviral activity of each compound against WT HCMV was assessed at 6 dpi with an automated plaque reduction assay (filled circles). Cytotoxicity of each compound was tested in parallel (filled squares). For both left and right graphs, error bars represent standard deviations from three independent experiments. Where no error bars are seen, the standard deviations were too small to be visible. (B) HTRF assays of GKD4, GKD6, and GKD9 for inhibition of interactions of WT UL53 with either WT or the indicated substitution mutants of UL50 (left graphs) or WT UL50 with either WT or the indicated substitution mutants of UL53 (right graphs). For both left and right graphs, error bars represent standard deviations from three independent experiments. Where no error bars are seen, the standard deviations were too small to be visible.
(PDF)

S8 Fig The antiviral activities of GKD4 and GKD6 against WT HCMV and mutant HCMV C214S. HFF cells infected with each virus indicated were incubated with GKD4 (left) or GKD6 (right) at different concentrations. Antiviral activities were assessed at 6 dpi using automated plaque reduction assays. The cytotoxicity of each compound was tested in parallel. Error bars represent standard deviations from three independent experiments. Where no error bars are seen, the standard deviations were too small to be visible. Curves were fit using nonlinear regression, and ED_50_ values and 95% confidence intervals (CI), and the p value for the difference between the ED_50_’s were calculated using GraphPad Prism 9.5.1 for MacOS.
(PDF)

S9 Fig Location of C214 relative to the subunit interfaces of the HCMV NEC. UL53 is shown in blue and UL50 in red with UL53 C214 shown in a space filling model just above the “B” interface of the NEC. The “A” interface involving segments of UL53 within the “vise” of UL50 is below and to the right of UL53 C214.
(PDF)

S10 Fig Docking using HADDOCK predicts a clash between GK1 and UL50. The top panel shows interactions predicted by HADDOCK of GK1 with UL53 (PDB ID: 5DOC), with GK1 covalently binding to UL53 Cys214 (purple bond between the sulfur of Cys 214 (yellow) and C13 of the GK1 acrylamide (black)) and making hydrophobic interactions (red lines) with Arg119, His104, and Ile120. The bottom panel shows a stick model of GK1 (green) bound to Cys214 (orange) in a space filling model of UL53 (PDB ID: 5DOCB, chain B; secondary structure elements shown in yellow), with the chlorine containing pyrrole ring clashing with UL50 in a space filling model of the NEC (PDB ID: 5D5N; blue with a transparent surface representation).
(PDF)

S11 Fig Structures of two compounds containing a scaffold similar to GK1’s that did not score as hits. (PDF)

S1 Table HTRF ratio and FRET emissions for merbromin-treated HCMV NEC. Of the libraries screened, two included the compound merbromin. Screening of the NEC was performed in duplicate (i.e. Plates 1A and 1B) for each library and positive and negative controls were included on each plate. FRET emissions from the acceptor were measured at 665 nm (Channel 1) and donor emissions at 620 nm (Channel 2). The HTRF ratio was calculated as follows: [(Abs_665nm/Abs_620nm)*10,000].
(PDF)

S2 Table *K*_d_ values for the interactions of UL50 mutants with UL53 and UL53 mutants with UL50. (PDF)

S3 Table Summary of the inhibitory activities of the analogs of GK1 and GK2 in HTRF assays and their antiviral activities and cytotoxicity. (PDF)

S4 Table HTRF ratio and FRET emissions for ibrutinib-treated HCMV NEC. Of the libraries screened, two, LINCS2 and Selleck, contained ibrutinib. Each plate was screened in duplicate (i.e., Plates 3A and 3B, and plates 4A and 4B) and well number (i.e., 1, 2, 3, 4). The concentration of ibrutinib was 10 mM, 2 mM, 400 μM and 80 μM in LINCS2 wells 1 to 4 respectively, so the concentrations in the assay were 50, 10, 2, and 0.4 μM, respectively, and 10 mM in the Sellecks well for an assay concentration of 50 μM. Positive and negative controls were included on each plate. FRET emissions from the acceptor were measured at 665 nm (Channel 1) and donor emissions at 620 nm (Channel 2). The HTRF ratio was calculated as follows: [(Abs_665nm/Abs_620nm)*10,000]. The Selleck plate showed a narrower difference between the positive and negative controls than the LINCS plate, but the Z’ score was 0.6, with 14% and 32% inhibition observed. Essentially no inhibition was observed from any of the LINCS wells.
(PDF)

S5 Table List of primers used in this study. (PDF)

S1 Text Design and testing of GK1 analogs. (PDF)

S1 Appendix List of libraries screened. (PDF)

S2 Appendix Compound characterization data. (PDF)

S3 Appendix Data for Figs 1, S2 and S3. (XLSX)

S4 Appendix Data for Figs 2, 3, 5, 6, S5, S7 and S8 and S2 Table. (XLSX)

## References

[1] Goodrum F, Britt W, Mocarski ES. Cytomegalovirus. In: Howley PM, Knipe DM, editors. Fields Virology: DNA Viruses. 7th ed: Wolters Kluwer; 2020. pp. 389–445.

[2] Yu D, Silva MC, Shenk T. Functional map of human cytomegalovirus AD169 defined by global mutational analysis. Proc Natl Acad Sci USA. 2003;100: 12396–12401. doi: 10.1073/pnas.1635160100 14519856 PMC218769

[3] Dunn W, Chou C, Li H, Hai R, Patterson D, Stolc V, et al. Functional profiling of a human cytomegalovirus genome. Proc Natl Acad Sci USA. 2003;100: 14223–14228. doi: 10.1073/pnas.2334032100 14623981 PMC283573

[4] Coen DM, Namchuk MN, Kuritzkes DR. Antiviral Agents. In: Howley PM, Knipe DM, editors. Field Virology: Emerging Virus. 7th ed: Wolters Kluwer. pp. 353–397.

[5] Mettenleiter TC. Herpesvirus assembly and egress. J Virol. 2002;76: 1537–1547. doi: 10.1128/jvi.76.4.1537-1547.2002 11799148 PMC135924

[6] Lye MF, Wilkie AR, Filman DJ, Hogle JM, Coen DM. Getting to and through the inner nuclear membrane during herpesvirus nuclear egress. Curr Opin Cell Biol. 2017;46: 9–16. doi: 10.1016/j.ceb.2016.12.007 28086162 PMC5503802

[7] Sanchez V, Britt W. Human cytomegalovirus egress: overcoming barriers and co-opting cellular functions. Viruses. 2022;14: 15. 10.3390/v14010015 PMC8778548 35062219

[8] Bigalke JM, Heldwein EE. Nuclear exodus: herpesviruses lead the way. Annu Rev Virol. 2016;3: 387–409. doi: 10.1146/annurev-virology-110615-042215 27482898 PMC5065387

[9] Roller RJ, Johnson DC. Herpesvirus nuclear egress across the outer nuclear membrane. Viruses. 2021;13: 2356. doi: 10.3390/v13122356 34960625 PMC8706699

[10] Camozzi D, Pignatelli S, Valvo C, Lattanzi G, Capanni C, Monte PD, et al. Remodelling of the nuclear lamina during human cytomegalovirus infection: role of the viral proteins pUL50 and pUL53. J Gen Virol. 2008;89: 731–740. doi: 10.1099/vir.0.83377-0 18272765

[11] Milbradt J, Auerochs S, Marschall M. Cytomegaloviral proteins pUL50 and pUL53 are associated with the nuclear lamina and interact with cellular protein kinase C. J Gen Virol. 2007;88: 2642–2650. doi: 10.1099/vir.0.82924-0 17872514

[12] Sam MD, Evans BT, Coen DM, Hogle JM. Biochemical, biophysical, and mutational analyses of subunit interactions of the human cytomegalovirus nuclear egress complex. J Virol. 2009;83: 2996–3006. doi: 10.1128/JVI.02441-08 19153235 PMC2655548

[13] Bigalke JM, Heuser T, Nicastro D, Heldwein EE. Membrane deformation and scission by the HSV-1 nuclear egress complex. Nat Commun. 2014;5: 4131. doi: 10.1038/ncomms5131 24916797 PMC4105210

[14] Bigalke JM, Heldwein EE. Structural basis of membrane budding by the nuclear egress complex of herpesviruses. EMBO J. 2015;34: 2921–2936. doi: 10.15252/embj.201592359 26511020 PMC4687684

[15] Hagen C, Dent KC, Zeev-Ben-Mordehai T, Grange M, Bosse JB, Whittle C, et al. Structural basis of vesicle formation at the inner nuclear membrane. Cell. 2015;163: 1692–1701. doi: 10.1016/j.cell.2015.11.029 26687357 PMC4701712

[16] Sharma M, Kamil JP, Coughlin M, Reim NI, Coen DM. Human cytomegalovirus UL50 and UL53 recruit viral protein kinase UL97, not protein kinase C, for disruption of nuclear lamina and nuclear egress in infected cells. J Virol. 2014;88: 249–262. doi: 10.1128/JVI.02358-13 24155370 PMC3911691

[17] Häge S, Büscher N, Pakulska V, Hahn F, Adrait A, Krauter S, et al. The complex regulatory role of cytomegalovirus nuclear egress protein pUL50 in the production of infectious virus. Cells. 2021;10: 3119. doi: 10.3390/cells10113119 34831342 PMC8625744

[18] Speed SD, Ashley J, Jokhi V, Nunnari J, Barria R, Li Y, et al. Nuclear envelope budding enables large ribonucleoprotein particle export during synaptic Wnt signaling. Cell. 2012;149: 832–846. doi: 10.1016/j.cell.2012.03.032 22579286 PMC3371233

[19] Jokni V, Ashley J, Nunnari J, Noma A, Ito N, Wakabayashi-Ito N, et al. Torsin mediates primary envelopment of large ribonucleoprotein granules at the nuclear envelope. Cell Rep. 2013;3: 988–995. doi: 10.1016/j.celrep.2013.03.015 23583177 PMC3683601

[20] Sharma M, Bender BJ, Kamil JP, Lye MF, Pesola JM, Reim NI, et al. Human cytomegalovirus UL97 phosphorylates the viral nuclear egress complex. J Virol. 2015;89: 523–534. doi: 10.1128/JVI.02426-14 25339763 PMC4301116

[21] Mou F, Wills EG, Park P, Baines JD. Effects of lamin A/C, lamin B1, and viral US3 kinase activity on viral infectivity, virion egress, and the targeting of herpes simplex virus U(L)34-encoded protein to the inner nuclear membrane. J Virol. 2008;82: 8094–8104. doi: 10.1128/JVI.00874-08 18524819 PMC2519571

[22] Lye MF, Sharma M, Omari KE, Filman DJ, Schuermann JP, Hogle JM, et al. Unexpected features and mechanism of heterodimer formation of a herpesvirus nuclear egress complex. EMBO J. 2015;34: 2937–2952. doi: 10.15252/embj.201592651 26511021 PMC4687685

[23] Zeev-Ben-Mordehai T, Weberruß M, Lorenz M, Cheleski J, Hellberg T, Whittle C, et al. Crystal structure of the herpesvirus nuclear egress complex provides insights into inner nuclear membrane remodeling. Cell Rep. 2015;13: 2645–2652. doi: 10.1016/j.celrep.2015.11.008 26711332 PMC4700048

[24] Muller YA, Häge S, Alkhashrom S, Höllriegl T, Weigert S, Dolles S, et al. High-resolution crystal structures of two prototypical ß- and γ-herpesviral nuclear egress complexes unravel the determinants of subfamily specificity. J Bio Chem. 2020;295: 3189–3201. 31980459 10.1074/jbc.RA119.011546 PMC7062154

[25] Walzer SA, Egerer-Sieber C, Sticht H, Sevvana M, Hohl K, Milbradt J, et al. Crystal structure of the human cytomegalovirus pUL50-pUL53 core nuclear egress complex provides insight into a unique assembly scaffold for virus-host protein interactions. J Bio Chem. 2015;290: 27452–27458. doi: 10.1074/jbc.C115.686527 26432641 PMC4645997

[26] Leigh KE, Sharma M, Sam M, Boeszoermenyi A, Filman DJ, Hogle JM, et al. Structure of a herpesvirus nuclear egress complex subunit reveals an interaction groove that is essential for viral replication. Proc Natl Acad Sci USA. 2015;112: 9010–9015. doi: 10.1073/pnas.1511140112 26150520 PMC4517201

[27] Milbradt J, Auerochs S, Sevvana M, Muller YA, Sticht H, Marschall M. Specific residues of a conserved domain in the N terminus of the human cytomegalovirus pUL50 protein determine its intranuclear interaction with pUL53. J Bio Chem. 2012;287: 24004–24016. 22589554 10.1074/jbc.M111.331207 PMC3390675

[28] Alkhashrom S, Kicuntod J, Stillger K, Lützenburg T, Anzenhofer C, Neundorf I, et al. A peptide inhibitor of the human cytomegalovirus core nuclear egress complex. Pharmaceuticals. 2022;15: 1040. doi: 10.3390/ph15091040 36145260 PMC9505826

[29] Schnee M, Wagner F, Koszinowski U, Ruzsics Z. A cell free protein fragment complementation assay for monitoring core interaction of the human cytomegalovirus nuclear egress complex. Antivir Res. 2012;95: 12–18. 22580129 10.1016/j.antiviral.2012.04.009

[30] Alkhashrom S, Kicuntod J, Häge S, Schweininger J, Muller YA, Lischka P, et al. Exploring the human cytomegalovirus core nuclear egress complex as a novel antiviral target: a new type of small molecule inhibitors. Viruses. 2021;13: 471. doi: 10.3390/v13030471 33809234 PMC7998563

[31] Kicuntod J, Alkhashrom S, Häge S, Diewald B, Müller R, Hahn F, et al. Properties of oligomeric interaction of the cytomegalovirus core nuclear egress complex (NEC) and its sensitivity to an NEC inhibitory small molecule. Viruses. 2021;13: 462. doi: 10.3390/v13030462 33799898 PMC8002134

[32] Degorce F, Card A, Soh S, Trinquet E, Knapik GP, Xie B. HTRF: a technology tailored for drug discovery—a review of theoretical aspects and recent applications. Curr Chem Genomics. 2009;3: 22–32. doi: 10.2174/1875397300903010022 20161833 PMC2802762

[33] Baell JB, Holloway GA. New substructure filters for removal of pan assay interference compounds (PAINS) from screening libraries and for their exclusion in bioassays. J Med Chem. 2010;53: 2719–2740. doi: 10.1021/jm901137j 20131845

[34] Khan AS, Murray MJ, Ho CM, Zuercher WJ, Reeves MB, Strang BL. High-throughput screening of a GlaxoSmithKline protein kinase inhibitor set identifies an inhibitor of human cytomegalovirus replication that prevents CREB and histone H3 post-translational modification. J Gen Virol. 2017;98: 754–768. doi: 10.1099/jgv.0.000713 28100301 PMC5817216

[35] Zhu K, Borrelli KW, Greenwood JR, Day T, Abel R, Farid RS, et al. Docking covalent inhibitors: a parameter free approach to pose prediction and scoring. J Chem Inf Model. 2014;54: 1932–1940. doi: 10.1021/ci500118s 24916536

[36] Dominguez C, Boelens R, Bonvin AMJJ. HADDOCK: a protein-protein docking approach based on biochemical or biophysical information. J Am Chem Soc. 2003;125: 1731–1737. doi: 10.1021/ja026939x 12580598

[37] Zundert GCP, Rodrigues JPGLM, Trellet M, Schmitz C, Kastritis PL, Karaca E, et al. The HADDOCK2.2 web server: user-friendly integrative modeling of biomolecular complexes. J Mol Biol. 2016;428: 720–725. doi: 10.1016/j.jmb.2015.09.014 26410586

[38] Milbradt J, Kraut A, Hutterer C, Sonntag E, Schmeiser C, Ferro M, et al. Proteomic analysis of the multimeric nuclear egress complex of human cytomegalovirus. Mol Cell Proteomics. 2014;13: 2132–2146. doi: 10.1074/mcp.M113.035782 24969177 PMC4125742

[39] Tillmanns J, Häge S, Borst EM, Wardin J, E J., Klebl B, et al. Assessment of covalently binding warhead compounds in the validation of the cytomegalovirus nuclear egress complex as an antiviral target. Cells. 2023;12: 1162. doi: 10.3390/cells12081162 37190072 PMC10137179

[40] Draganova E, Zhang J, Zhou ZH, Heldwein EE. Structural basis for capsid recruitment and coat formation during HSV-1 nuclear egress. eLife. 2020;9: e56627. doi: 10.7554/eLife.56627 32579107 PMC7340501

[41] Hamirally S, Kamil JP, Ndassa-Colday YM, Lin AJ, Jahng WJ, Baek M-C, et al. Viral mimicry of cdc2/cyclin-dependent kinase 1 mediates disruption of nuclear lamina during human cytomegalovirus nuclear egress. PLos Pathog. 2009;5: e1000275. doi: 10.1371/journal.ppat.1000275 19165338 PMC2625439

[42] Zuccola HJ, Filman DJ, Coen DM, Hogle JM. The crystal structure of an unusual processivity factor, herpes simplex virus UL42, bound to the C terminus of its cognate polymerase. Mol Cell. 2000;5: 267–278. doi: 10.1016/s1097-2765(00)80422-0 10882068

[43] Randell JC, Komazin G, Jiang C, Hwang CBC, Coen DM. Effects of substitutions of arginine residues on the basic surface of herpes simplex virus UL42 support a role for DNA binding in processive DNA synthesis. J Virol. 2005;79: 12025–12034. doi: 10.1128/JVI.79.18.12025-12034.2005 16140778 PMC1212618

[44] Graham TGW, Walter JC, Loparo JJ. Two-stage synapsis of DNA ends during non-homologous end joining. Mol Cell. 2016;61: 850–858. doi: 10.1016/j.molcel.2016.02.010 26990988 PMC4799494

[45] Newton P, Harrison P, Clulow S. A novel method for determination of the affinity of protein:protein interactions in homogeneous assays. J Biomol Screen. 2008;13: 674–682. doi: 10.1177/1087057108321086 18626116

[46] Kamil JP, Coen DM. Human cytomegalovirus protein kinase UL97 forms a complex with the tegument phosphoprotein pp65. J Virol. 2007;81: 10659–10668. doi: 10.1128/JVI.00497-07 17634236 PMC2045453

[47] Wilkie AR, Sharma M, Coughlin M, Pesola JM, Ericsson M, Lawler JL, et al. Human cytomegalovirus nuclear egress complex subunit, UL53, associates with capsids and myosin Va, but is not important for capsid localization towards the nuclear periphery. Viruses. 2022;14: 479. doi: 10.3390/v14030479 35336886 PMC8949324

[48] Tischer BK, von Einem J, Kaufer B, Osterrieder N. Two-step red-mediated recombination for versatile high-efficiency markerless DNA manipulation in Escherichia coli. Biotechniques. 2006;40: 191–197. doi: 10.2144/000112096 16526409

[49] Tischer BK, Smith GA, Osterrieder N. En passant mutagenesis: a two step markerless red recombination system. Methods Mol Biol. 2010;634: 421–430. doi: 10.1007/978-1-60761-652-8_30 20677001

[50] Pilger BD, Cui C, Coen DM. Identification of a small molecule that inhibits herpes simplex virus DNA polymerase subunit interactions of the viral polymerase. Chem Biol. 2004;11: 647–654. 15157875 10.1016/j.chembiol.2004.01.018

[51] Loregian A, Coen DM. Selective anti-cytomegalovirus compounds discovered by screening for inhibitors of subunit interactions of the viral polymerase. Chem Biol. 2006;13: 191–200. doi: 10.1016/j.chembiol.2005.12.002 16492567

[52] Chen H, Coseno M, Ficarro SB, Mansueto MS, Komazin-Meredith G, Boissel S, et al. A small covalent allosteric inhibitor of human cytomegalovirus DNA polymerase subunit interactions. ACS Infect Dis. 2017;3: 112–118. doi: 10.1021/acsinfecdis.6b00079 28183184 PMC5480311

[53] Hughes CS, Foehr S, Garfield DA, Furlong EE, Steinmetz LM, Krijgsveld J. Ultrasensitive proteome analysis using paramagnetic bead technology. Mol Syst Biol. 2014;10: 757. doi: 10.15252/msb.20145625 25358341 PMC4299378

[54] Alexander WM, Ficarro SB, Adelmant G, Marto JA. multiplierz v2.0: A python-based ecosystem for shared access and analysis of native mass spectrometry data Proteomics. 2017;17: 1700091. 10.1002/pmic.201700091 28686798

[55] Ficarro SB, Alexander WM, Marto JA. mzStudio: A dynamic digital canvas for user-driven interrogation of mass spectrometry data. Proteomics. 2017;5: 1–8. doi: 10.3390/proteomes5030020 28763045 PMC5620537

[56] Epic. Schrödinger Release 2021–3: Protein Preparation Wizard. New York: Schrödinger LLC.; 2021.

[57] Sastry GM, Adzhigirey M, Day T, Annabhimoju R, Sherman W. Protein and ligand preparation: parameters, protocols, and influence on virtual screening enrichments. J Comput Aid Mol Des. 2013;27: 221–234. doi: 10.1007/s10822-013-9644-8 23579614

[58] Schrödinger release 2021–3: LigPrep. New York: Schrödinger LLC.; 2021.

[59] Adasme MF, Linnemann KL, Bolz SN, Kaiser F, Salentin S, Haupt VJ, et al. PLIP 2021: expanding the scope of the protein-ligand interaction profiler to DNA and RNA. Nucleic Acids Res. 2021;49: W530–W534. doi: 10.1093/nar/gkab294 33950214 PMC8262720

[60] Salentin S, Schreiber S, Haup VJ, Adasme MF, Schroeder M. PLIP: fully automated protein-ligand interaction profiler. Nucl Acids Res. 2015;43: W443–W447. doi: 10.1093/nar/gkv315 25873628 PMC4489249

[61] Rodrigues JPGLM, Teixeira JMC, Trellet M, Bonvin AMJJ. pdb-tools: a swiss army knife for molecular structures. F1000Res. 2018;7: 1961. doi: 10.12688/f1000research.17456.1 30705752 PMC6343223

