# Supplementary material for: A small molecule exerts selective antiviral activity by targeting the human cytomegalovirus nuclear egress complex
Source: PLoS Pathog. 2023 Nov 17;19(11):e1011781. doi: 10.1371/journal.ppat.1011781 (PMC10691697; doi:10.1371/journal.ppat.1011781)
Supplement: S1 Fig — Top: HCMV His-UL50 and Myc-UL53 were purified individually using affinity chromatography and then chromatographed on a size-exclusion column at a ratio of 1 Myc-UL53 to 2 His-UL50 (green trace). A trace of His-UL50 alone is shown for comparison (blue trace). Bottom: Aliquots of fractions corresponding to the faster eluting peak following chromatography of the protein mixture were resolved using SDS-PAGE and the indicated fractions were pooled for use in HTRF assays. (PDF) [file ppat.1011781.s001.pdf]

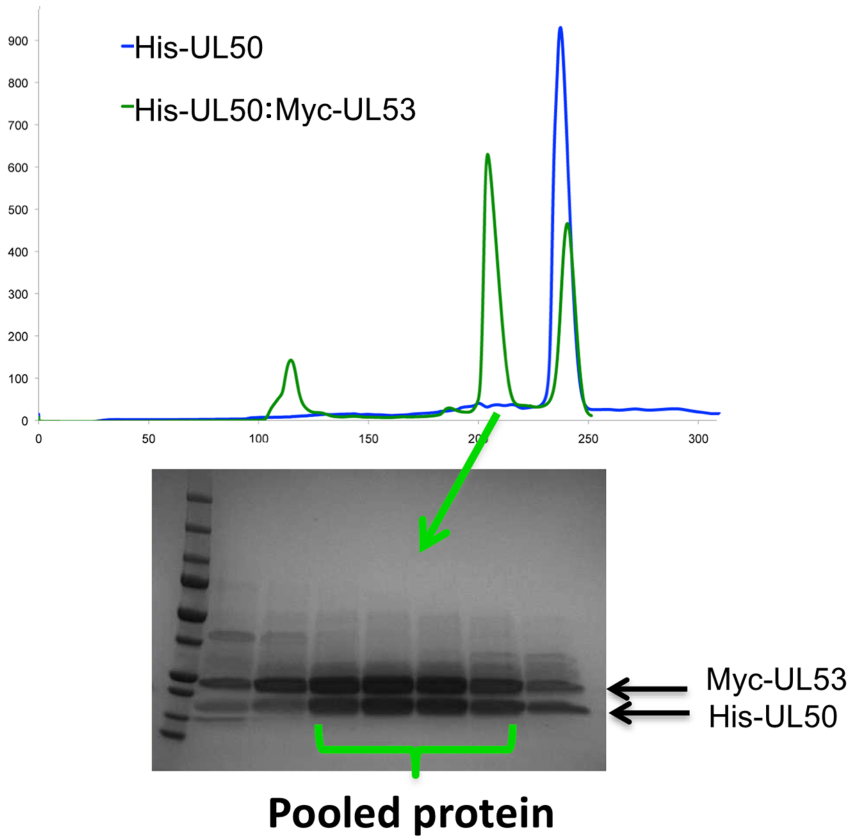

S1 Fig. Purification of HCMV NEC containing His-UL50 and Myc-UL53 using size-exclusion chromatography. Top: HCMV His-UL50 and Myc-UL53 were purified individually using affinity chromatography and then chromatographed on a size-exclusion column at a ratio of 1 Myc-UL53 to 2 His-UL50 (green trace). A trace of His-UL50 alone is shown for comparison (blue trace). Bottom: Aliquots of fractions corresponding to the faster eluting peak following chromatography of the protein mixture were resolved using SDS-PAGE and the indicated fractions were pooled for use in HTRF assays.
