# Supplementary material for: A small molecule exerts selective antiviral activity by targeting the human cytomegalovirus nuclear egress complex
Source: PLoS Pathog. 2023 Nov 17;19(11):e1011781. doi: 10.1371/journal.ppat.1011781 (PMC10691697; doi:10.1371/journal.ppat.1011781)
Supplement: S2 Fig — C-terminal 36 residues of HSV-1 UL30 (P30) fused to Myc-tagged GST was mixed with His-tagged HSV-1 UL42 (left graph) or His-tagged MCMV M50 was mixed with Myc-tagged MCMV M53 (right graph) and assayed using the same reagents and methods as used for the HTRF assay for HCMV NEC interactions. Untagged P30 or UL42 (left graph) or untagged M50 (right graph) substantially reduced HTRF signal in their respective assays. (PDF) [file ppat.1011781.s002.pdf]

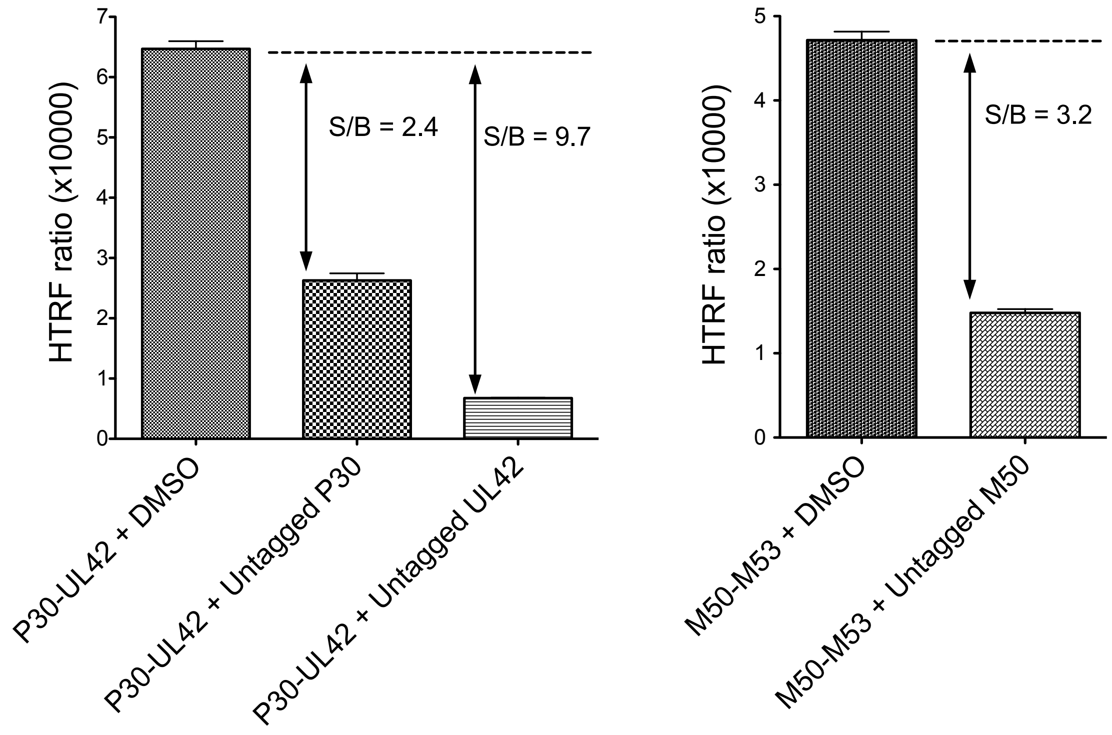

S2 Fig. HTRF assays for inhibition of HSV-1 UL30 peptide-UL42 and MCMV M50-M53 interactions. C-terminal 36 residues of HSV-1 UL30 (P30) fused to Myc-tagged GST was mixed with His-tagged HSV-1 UL42 (left graph) or His-tagged MCMV M50 was mixed with Myc-tagged MCMV M53 (right graph) and assayed using the same reagents and methods as used for the HTRF assay for HCMV NEC interactions. Untagged P30 or UL42 (left graph) or untagged M50 (right graph) substantially reduced HTRF signal in their respective assays.
