# Supplementary material for: A small molecule exerts selective antiviral activity by targeting the human cytomegalovirus nuclear egress complex
Source: PLoS Pathog. 2023 Nov 17;19(11):e1011781. doi: 10.1371/journal.ppat.1011781 (PMC10691697; doi:10.1371/journal.ppat.1011781)
Supplement: S3 Fig — The HTRF assay was performed at different concentrations of GK1 either with NEC as a target (red lines) or without protein targets (blue lines). Under conditions where GK1 strongly inhibited HTRF (left graph) or FRET signal at 665 nm from the NEC (right graph), it had little or no effect on HTRF in the absence of protein target. (PDF) [file ppat.1011781.s003.pdf]

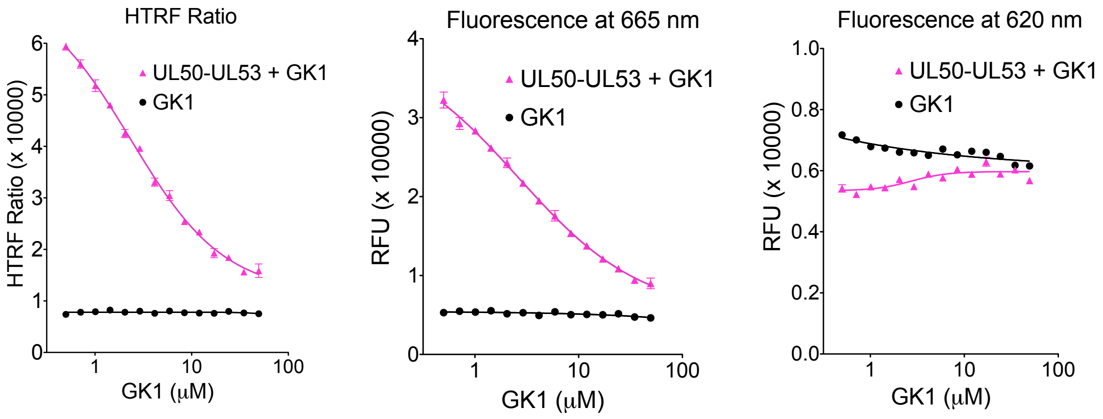

S3 Fig. GK1 does not interfere with the HTRF assay. The HTRF assay was performed at different concentrations of GK1 either with NEC as a target (pink lines) or without protein targets (black lines). Under conditions where GK1 strongly inhibited HTRF (left graph) or FRET signal at 665 nm from the NEC (middle graph), it had little or no effect on HTRF in the absence of protein target (right graph).
