# Supplementary material for: A small molecule exerts selective antiviral activity by targeting the human cytomegalovirus nuclear egress complex
Source: PLoS Pathog. 2023 Nov 17;19(11):e1011781. doi: 10.1371/journal.ppat.1011781 (PMC10691697; doi:10.1371/journal.ppat.1011781)
Supplement: S4 Fig — HFF cells were mock infected or infected with WT HCMV at an MOI of 1. Following absorption, medium containing either DMSO or 10 μM GK2 was added. At 72 hpi, cells were lysed, proteins resolved using SDS-PAGE, and the expression of the viral proteins indicated to the left was assessed by immunoblotting. Similar results were obtained in a second independent experiment. (PDF) [file ppat.1011781.s004.pdf]

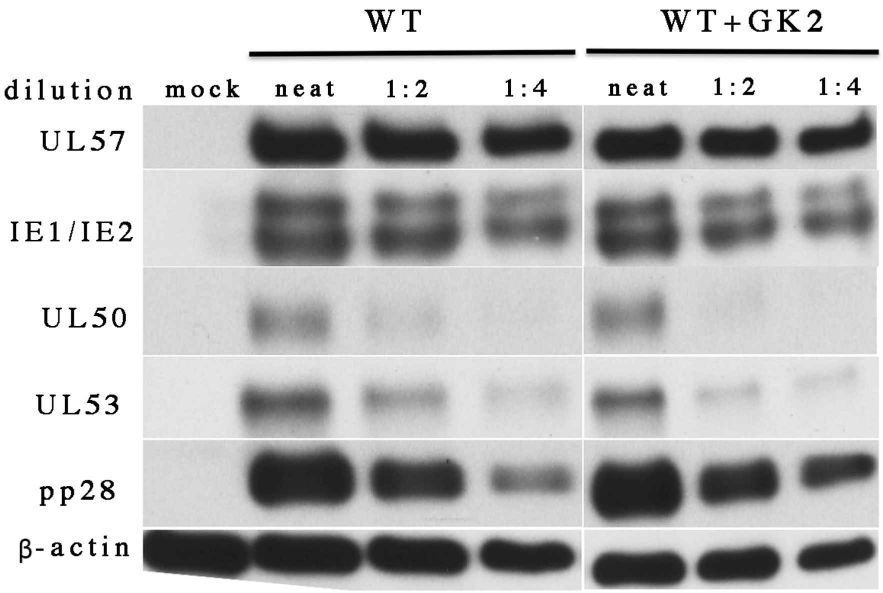

S4 Fig. Effect of GK2 on HCMV protein expression in infected cells. HFF cells were mock infected or infected with WT HCMV at an MOI of 1. Following absorption, medium containing either DMSO or 10 μM GK2 was added. At 72 hpi, cells were lysed, proteins resolved using SDS-PAGE, and the expression of the viral proteins indicated to the left was assessed by immunoblotting. Similar results were obtained in a second independent experiment.
