# Supplementary material for: A small molecule exerts selective antiviral activity by targeting the human cytomegalovirus nuclear egress complex
Source: PLoS Pathog. 2023 Nov 17;19(11):e1011781. doi: 10.1371/journal.ppat.1011781 (PMC10691697; doi:10.1371/journal.ppat.1011781)
Supplement: S5 Fig — HFF cells infected with each virus indicated were incubated with GK2 at different concentrations. Automated plaque reduction assays were performed at 6 dpi. Cytotoxicity assays of GK2 were performed in parallel. Error bars represent standard deviations from three independent experiments. Where no error bars are seen, the standard deviations were too small to be visible. Curves were fit using nonlinear regression and ED50 values and the 95% confidence intervals (CI) and p value for the difference between the ED50’s were calculated using GraphPad Prism 9.5.1 for MacOS. (PDF) [file ppat.1011781.s005.pdf]

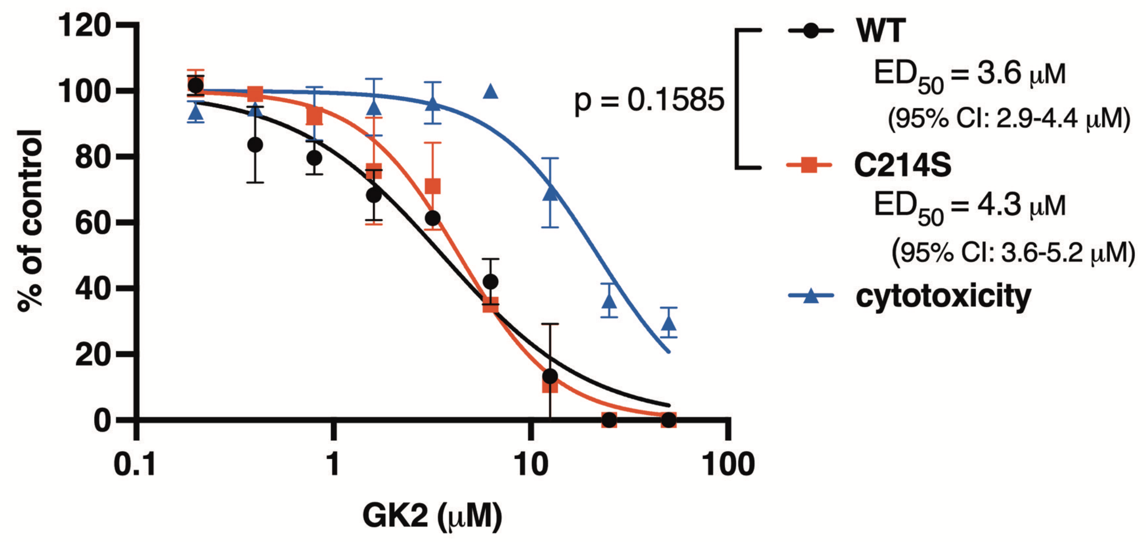

S5 Fig. Antiviral activities of GK2 against WT HCMV and mutant HCMV C214S. HFF cells infected with each virus indicated were incubated with GK2 at different concentrations. Automated plaque reduction assays were performed at 6 dpi. Cytotoxicity assays of GK2 were performed in parallel. Error bars represent standard deviations from three independent experiments. Where no error bars are seen, the standard deviations were too small to be visible. Curves were fit using nonlinear regression, and ED_{50} values and the 95% confidence intervals (CI), and p value for the difference between the ED_{50}'s were calculated using GraphPad Prism 9.5.1 for MacOS.
