# Supplementary figures and images for: A small molecule exerts selective antiviral activity by targeting the human cytomegalovirus nuclear egress complex
Source: PLoS Pathog. 2023 Nov 17;19(11):e1011781. doi: 10.1371/journal.ppat.1011781 (PMC10691697; doi:10.1371/journal.ppat.1011781)

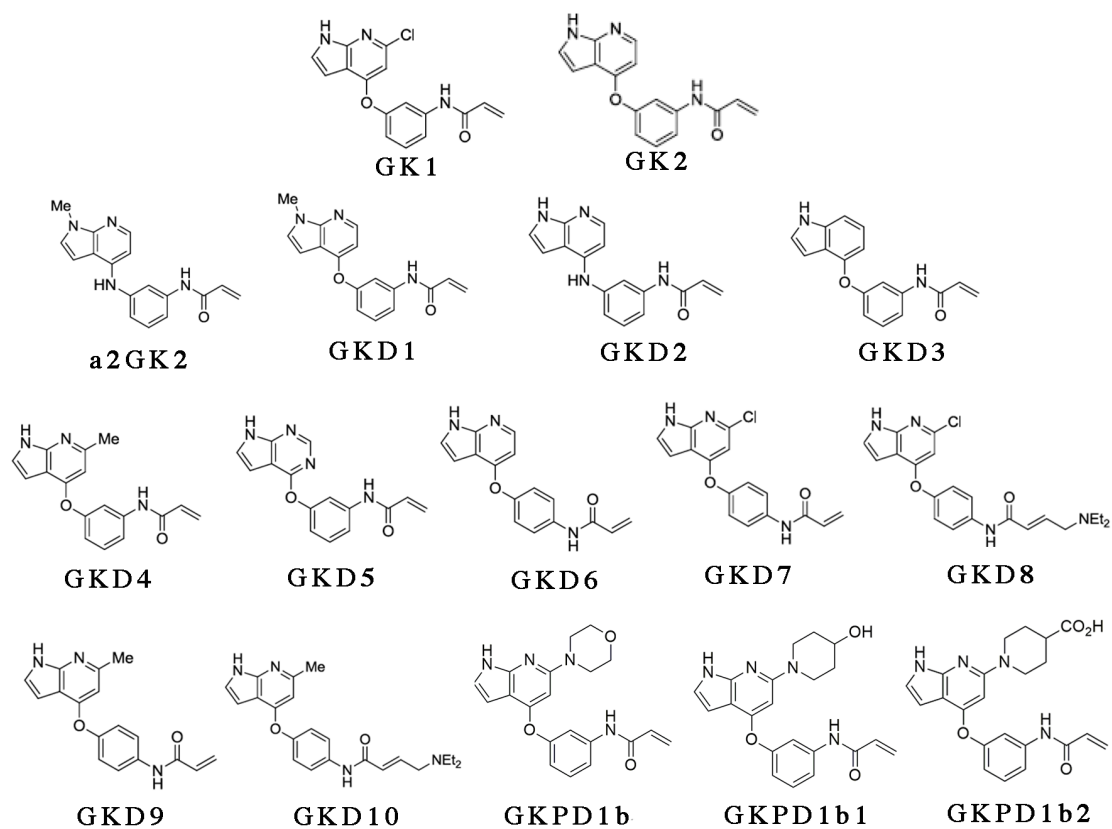

**S6 Fig. The chemical structures of analogs of GK1 and GK2**

Supplement: S6 Fig — (PDF) [file ppat.1011781.s006.pdf]
