# Supplementary material for: A small molecule exerts selective antiviral activity by targeting the human cytomegalovirus nuclear egress complex
Source: PLoS Pathog. 2023 Nov 17;19(11):e1011781. doi: 10.1371/journal.ppat.1011781 (PMC10691697; doi:10.1371/journal.ppat.1011781)
Supplement: S7 Fig — (A, left graphs) Dose-response of the inhibitory activities of GKD4, GKD6 and GKD9 against interactions of HCMV UL50-UL53 (black) or the UL30 peptide (P30)-UL42 (counter screen, red) in HTRF assays. (A, right graphs). The antiviral activity of each compound against WT HCMV was assessed at 6 dpi with an automated plaque reduction assay (filled circles). Cytotoxicity of each compound was tested in parallel (filled squares). For both left and right graphs, error bars represent standard deviations from three independent experiments. Where no error bars are seen, the standard deviations were too small to be visible. (B) HTRF assays of GKD4, GKD6, and GKD9 for inhibition of interactions of WT UL53 with either WT or the indicated substitution mutants of UL50 (left graphs) or WT UL50 with either WT or the indicated substitution mutants of UL53 (right graphs). For both left and right graphs, error bars represent standard deviations from three independent experiments. Where no error bars are seen, the standard deviations were too small to be visible. (PDF) [file ppat.1011781.s007.pdf]

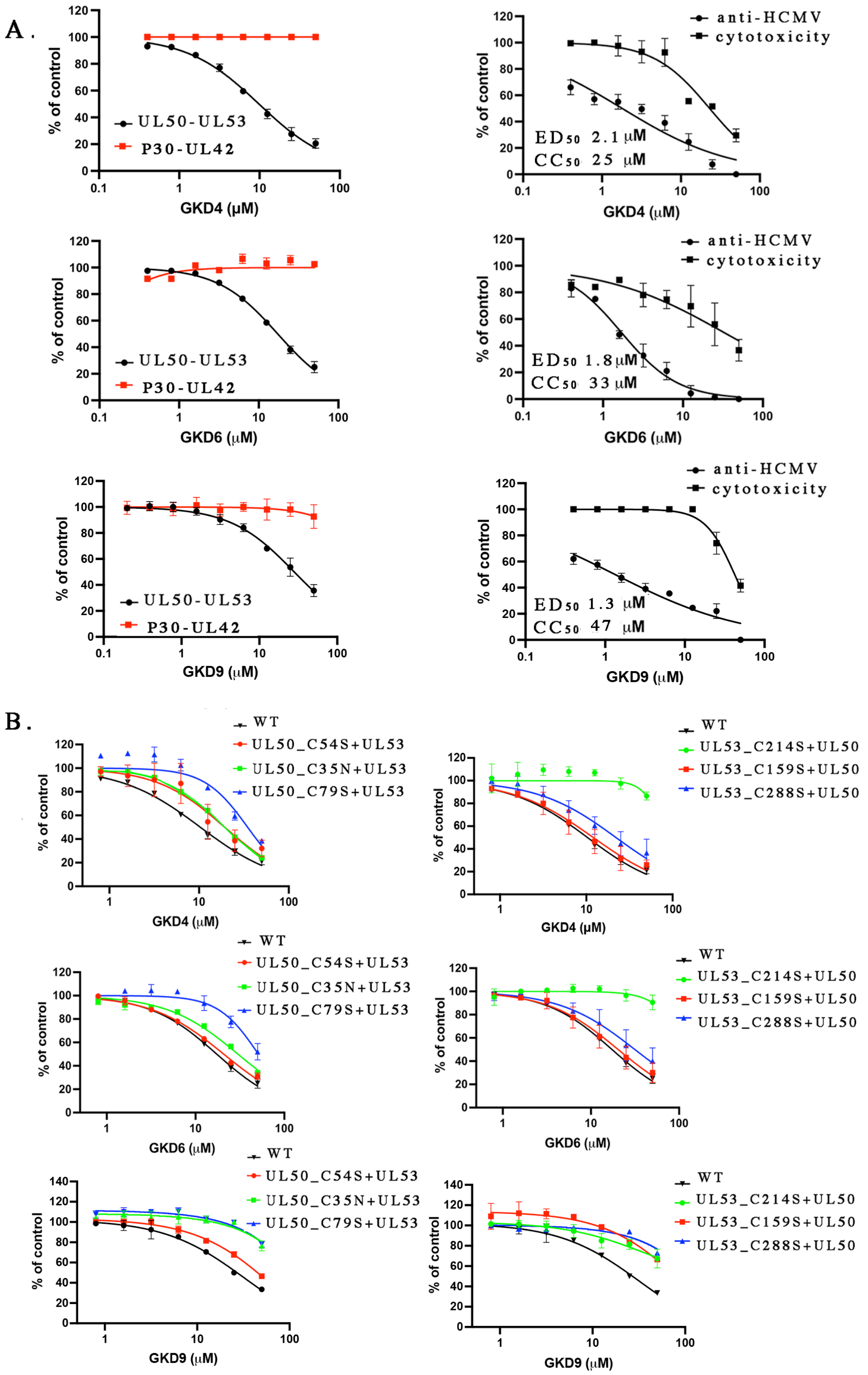
A.
% of control
UL50-UL53
P30-UL42
GKD4 (μM)
anti-HCMV
cytotoxicity
ED50 2.1 μM
CC50 25 μM
GKD6 (μM)
ED50 1.8 μM
CC50 33 μM
GKD9 (μM)
ED50 1.3 μM
CC50 47 μM
B.
WT
UL50_C54S+UL53
UL50_C35N+UL53
UL50_C79S+UL53
UL53_C214S+UL50
UL53_C159S+UL50
UL53_C288S+UL50

S7 Fig. Activities of analogs GKD4, GKD6 and GKD9. (A, left graphs) Dose-response of the inhibitory activities of GKD4, GKD6 and GKD9 against interactions of HCMV UL50-UL53 (black) or the UL30 peptide (P30)-UL42 (counter screen, red) in HTRF assays. (A, right graphs) The antiviral activity of each compound against WT HCMV was assessed at 6 dpi with an automated plaque reduction assay (filled circles). Cytotoxicity of each compound was tested in parallel (filled squares). For both left and right graphs, error bars represent standard deviations from three independent experiments. Where no error bars are seen, the standard deviations were too small to be visible.

(B) HTRF assays of GKD4, GKD6, and GKD9 for inhibition of interactions of WT UL53 with either WT or the indicated substitution mutants of UL50 (left graphs) or WT UL50 with either WT or the indicated substitution mutants of UL53 (right graphs). For both left and right graphs, error bars represent standard deviations from three independent experiments. Where no error bars are seen, the standard deviations were too small to be visible.
