# Supplementary material for: A small molecule exerts selective antiviral activity by targeting the human cytomegalovirus nuclear egress complex
Source: PLoS Pathog. 2023 Nov 17;19(11):e1011781. doi: 10.1371/journal.ppat.1011781 (PMC10691697; doi:10.1371/journal.ppat.1011781)
Supplement: S8 Fig — HFF cells infected with each virus indicated were incubated with GKD4 (left) or GKD6 (right) at different concentrations. Antiviral activities were assessed at 6 dpi using automated plaque reduction assays. The cytotoxicity of each compound was tested in parallel. Error bars represent standard deviations from three independent experiments. Where no error bars are seen, the standard deviations were too small to be visible. Curves were fit using nonlinear regression, and ED50 values and 95% confidence intervals (CI), and the p value for the difference between the ED50’s were calculated using GraphPad Prism 9.5.1 for MacOS. (PDF) [file ppat.1011781.s008.pdf]

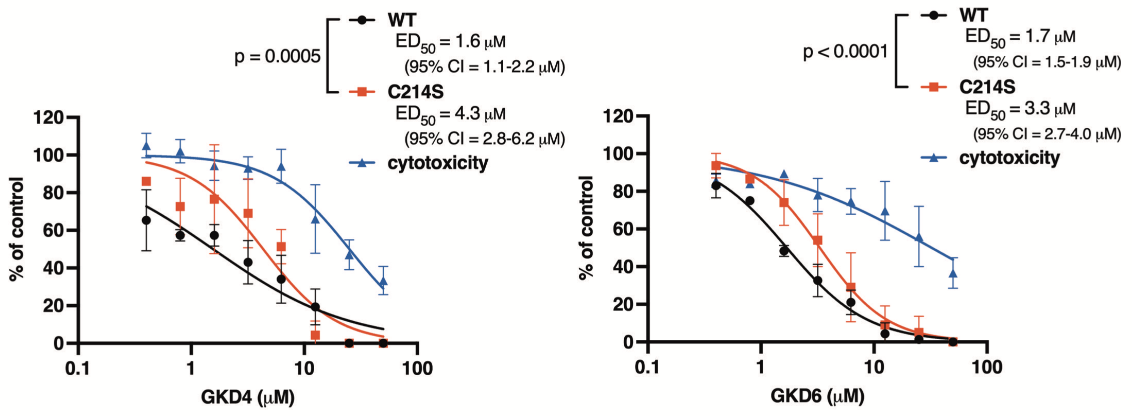

S8 Fig. The antiviral activities of GKD4 and GKD6 against WT HCMV and mutant HCMV C214S. HFF cells infected with each virus indicated were incubated with GKD4 (left) or GKD6 (right) at different concentrations. Antiviral activities were assessed at 6 dpi using automated plaque reduction assays. The cytotoxicity of each compound was tested in parallel. Error bars represent standard deviations from three independent experiments. Where no error bars are seen, the standard deviations were too small to be visible. Curves were fit using nonlinear regression, and ED_{50} values and 95% confidence intervals (CI), and the p value for the difference between the ED_{50}'s were calculated using GraphPad Prism 9.5.1 for MacOS.
