# Supplementary material for: A small molecule exerts selective antiviral activity by targeting the human cytomegalovirus nuclear egress complex
Source: PLoS Pathog. 2023 Nov 17;19(11):e1011781. doi: 10.1371/journal.ppat.1011781 (PMC10691697; doi:10.1371/journal.ppat.1011781)
Supplement: S9 Fig — UL53 is shown in blue and UL50 in red with UL53 C214 shown in a space filling model just above the “B” interface of the NEC. The “A” interface involving segments of UL53 within the “vise” of UL50 is below and to the right of UL53 C214. (PDF) [file ppat.1011781.s009.pdf]

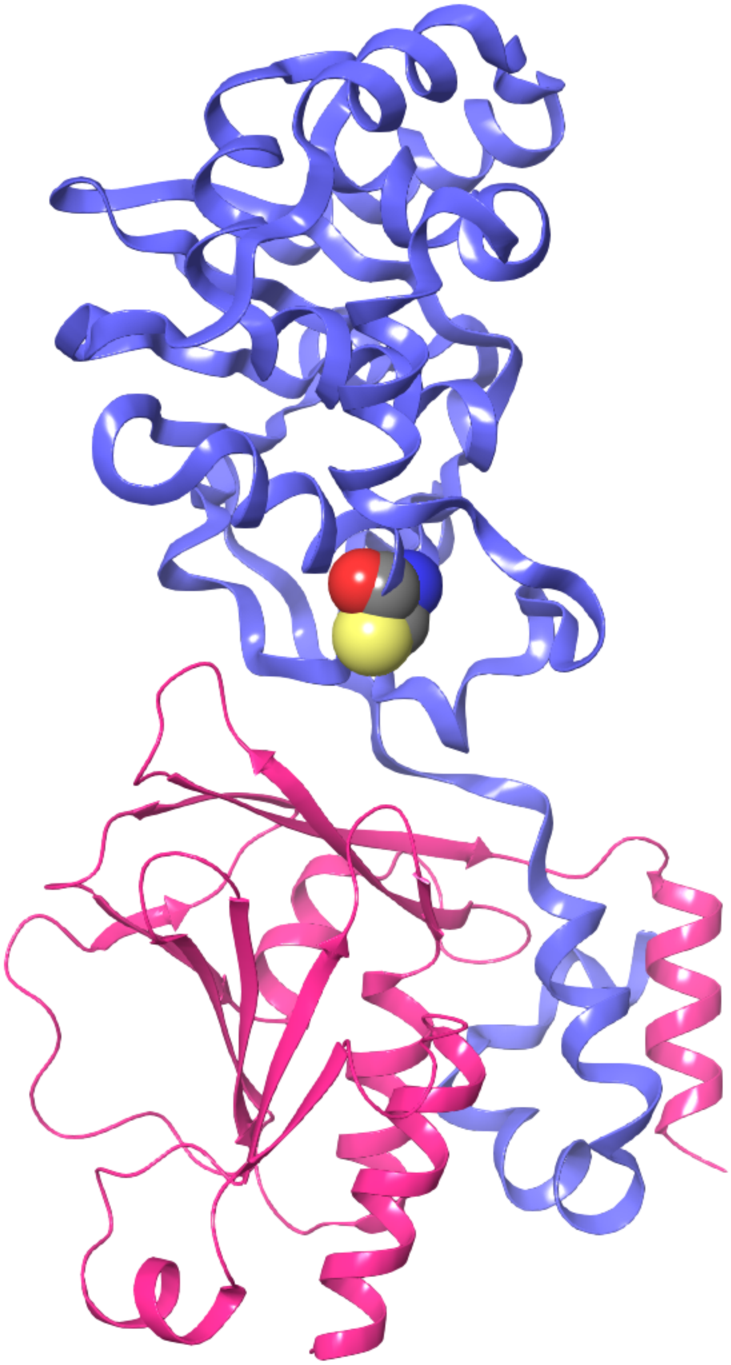

S9 Fig. Location of Cys214 relative to the subunit interfaces of the HCMV NEC. UL53 is shown in blue and UL50 in red with Cys214 shown in a space filling model just above the "B" interface of the NEC. The "A" interface involving segments of UL53 within the "vise" of UL50 is below and to the right of Cys214.
