# Supplementary material for: A small molecule exerts selective antiviral activity by targeting the human cytomegalovirus nuclear egress complex
Source: PLoS Pathog. 2023 Nov 17;19(11):e1011781. doi: 10.1371/journal.ppat.1011781 (PMC10691697; doi:10.1371/journal.ppat.1011781)
Supplement: S10 Fig — The top panel shows interactions predicted by HADDOCK of GK1 with UL53 (PDB ID: 5DOC), with GK1 covalently binding to UL53 Cys214 (purple bond between the sulfur of Cys 214 (yellow) and C13 of the GK1 acrylamide (black)) and making hydrophobic interactions (red lines) with Arg119, His104, and Ile120. The bottom panel shows a stick model of GK1 (green) bound to Cys214 (orange) in a space filling model of UL53 (PDB ID: 5DOCB, chain B; secondary structure elements shown in yellow), with the chlorine containing pyrrole ring clashing with UL50 in a space filling model of the NEC (PDB ID: 5D5N; blue with a transparent surface representation). (PDF) [file ppat.1011781.s010.pdf]

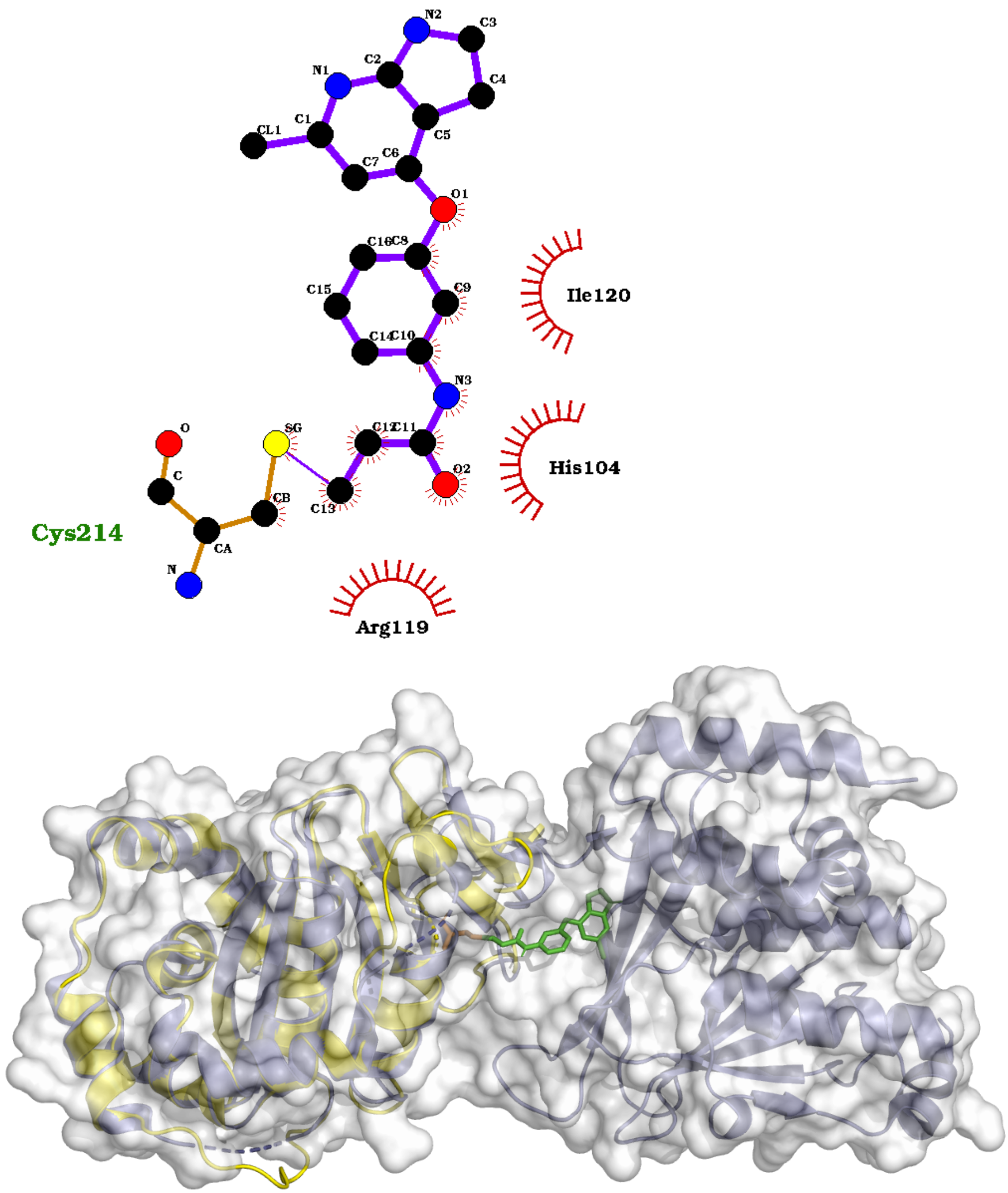

S10 Fig. Docking using HADDOCK predicts a clash between GK1 and UL50. The top panel shows interactions predicted by HADDOCK of GK1 with UL53 (PDB ID: 5DOC), with GK1 covalently binding to UL53 Cys214 (purple bond between the sulfur of Cys 214 (yellow) and C13 of the GK1 acrylamide (black)) and making hydrophobic

interactions (red lines) with Arg119, His04, and Ile120. The bottom panel shows a stick model of GK1 (green) bound to Cys214 (orange) in a space filling model of UL53 (PDB ID: 5DOCB, chain B; secondary structure elements shown in yellow), with the chlorine containing pyrrole ring clashing with UL50 in a space filling model of the NEC (PDB ID: 5D5N; blue with a transparent surface representation).
