# Supplementary material for: A small molecule exerts selective antiviral activity by targeting the human cytomegalovirus nuclear egress complex
Source: PLoS Pathog. 2023 Nov 17;19(11):e1011781. doi: 10.1371/journal.ppat.1011781 (PMC10691697; doi:10.1371/journal.ppat.1011781)
Supplement: S11 Fig — (PDF) [file ppat.1011781.s011.pdf]

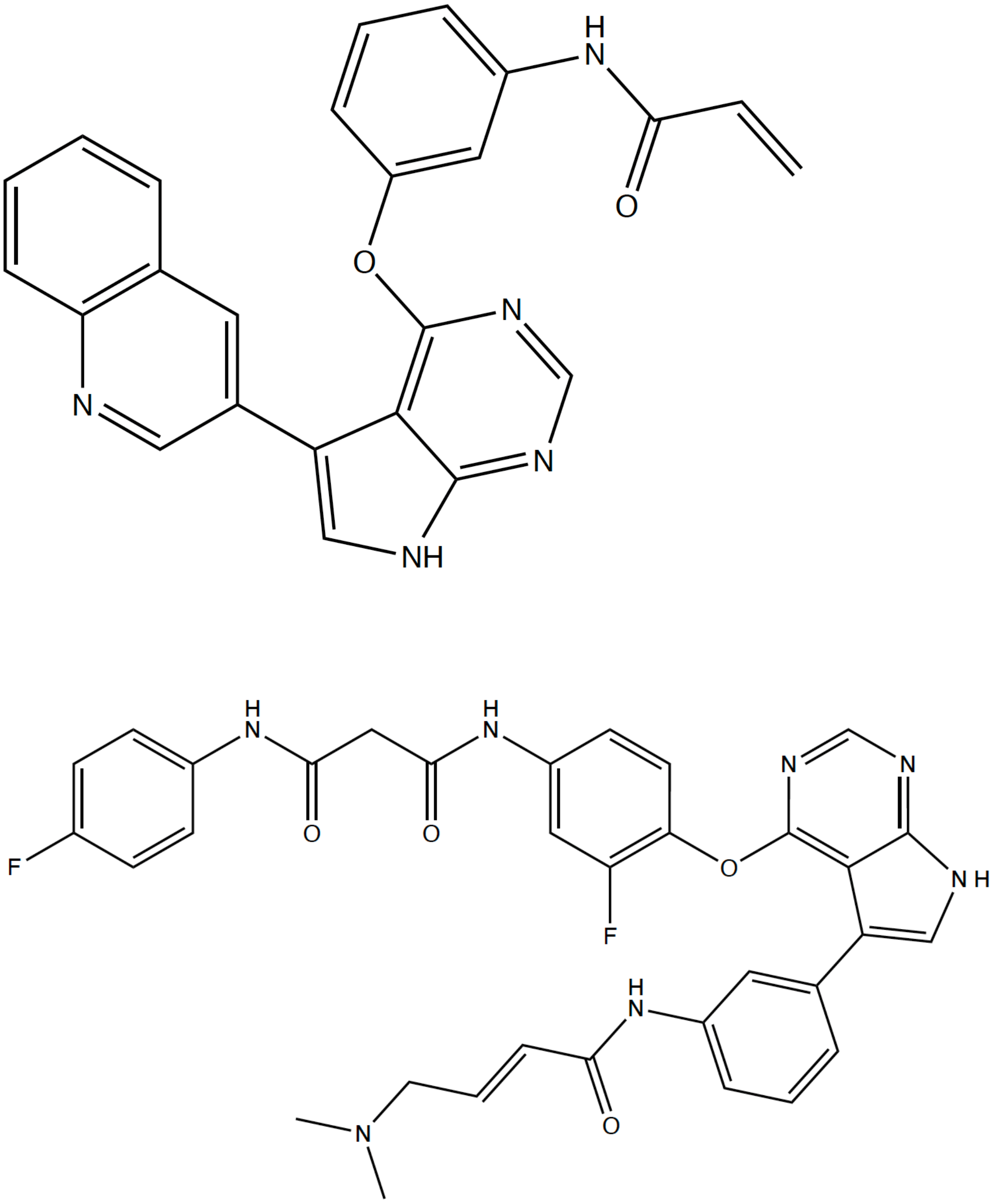

S11 Fig. Structures of two compounds containing a scaffold similar to GK1's that did not score as hits.
