# Supplementary material for: A small molecule exerts selective antiviral activity by targeting the human cytomegalovirus nuclear egress complex
Source: PLoS Pathog. 2023 Nov 17;19(11):e1011781. doi: 10.1371/journal.ppat.1011781 (PMC10691697; doi:10.1371/journal.ppat.1011781)
Supplement: S1 Table — Of the libraries screened, two included the compound merbromin. Screening of the NEC was performed in duplicate (i.e. Plates 1A and 1B) for each library and positive and negative controls were included on each plate. FRET emissions from the acceptor were measured at 665 nm (Channel 1) and donor emissions at 620 nm (Channel 2). The HTRF ratio was calculated as follows: [(Abs_665nm/Abs_620nm)*10,000]. (PDF) [file ppat.1011781.s012.pdf]

S1 Table

Plate	1A	1B	2A	2B
**Experimental**				
Library	Microsource1		Prestwick2	
Reagent Vendor	Microsource		Prestwick	
Compound	Merbromin			
HTRF Channel 1 (665 nm)	14121	14512	5412	5397
HTRF Channel 2 (620 nm)	3705	3793	4560	4809
HTRF Ratio	38113	38260	11868	11223
**Positive Control**				
HTRF Channel 1 (665 nm)	6220	6335	6555	6402
HTRF Channel 2 (620 nm)	10279	10489	11279	11045
HTRF Ratio	6053	6041	5810	5799
**Negative Control**				
HTRF Channel 1 (665 nm)	15982	16932	7941	7684
HTRF Channel 2 (620 nm)	7061	6958	8541	8544
HTRF Ratio	22713	24373	9195	8997

S1 Table. HTRF ratio and FRET emissions for Merbromin-treated HCMV NEC. Of the libraries screened, two included the compound merbromin. Screening of the NEC was performed in duplicate (i.e. Plates 1A and 1B) for each library and positive and negative controls were included on each plate. FRET emissions from the acceptor were measured at 665 nm (Channel 1) and donor emissions at 620 nm (Channel 2). The HTRF ratio was calculated as follows: [(Abs_665nm/Abs_620nm)*10,000].
