# Supplementary material for: A small molecule exerts selective antiviral activity by targeting the human cytomegalovirus nuclear egress complex
Source: PLoS Pathog. 2023 Nov 17;19(11):e1011781. doi: 10.1371/journal.ppat.1011781 (PMC10691697; doi:10.1371/journal.ppat.1011781)
Supplement: S2 Table — (PDF) [file ppat.1011781.s013.pdf]

S2 Table. K_d values for the interactions of UL50 mutants with UL53 and UL53 mutants with UL50.

	K_d (μM)
UL50 - UL53	0.30 ± 0.12[1]
UL50_C54S – UL53	0.24 ± 0.14
UL50_C35N – UL53	0.15 ± 0.06
UL50_C79S – UL53	0.34 ± 0.15
UL50 – UL53_C214S	0.13 ± 0.01
UL50 – UL53_C159S	0.18 ± 0.02
UL50 – UL53_C288S	0.32 ± 0.10

[1] Error bars represent standard deviations from two independent experiments.
