# Supplementary material for: A small molecule exerts selective antiviral activity by targeting the human cytomegalovirus nuclear egress complex
Source: PLoS Pathog. 2023 Nov 17;19(11):e1011781. doi: 10.1371/journal.ppat.1011781 (PMC10691697; doi:10.1371/journal.ppat.1011781)
Supplement: S3 Table — (PDF) [file ppat.1011781.s014.pdf]

S3 Table Summary of the inhibitory activities of the analogs of GK1 and GK2 in HTRF assays and their antiviral activities and cytotoxicity

Compound	HTRF assay	Antiviral activity and cytotoxicity		
	IC_{50} (μM)	ED_{50} (μM)	CC_{50} (μM)	CC_{50}/ED_{50}
GK1	5.3	0.83	22	27
GK2	21	3.9	21	5.4
a2GK2	>50	2.2	9.3	4.2
GKD1	16.6	16	14	0.9
GKD2 (free amine)	>50	5.8	14	2.4
GKD3	9.9	17.5	50	3
GKD4	9.9	2.1	25	12
GKD5	>50	18	45	2.5
GKD6	17	1.8	33	18
GKD7	>50	16	38	2.4
GKD8	>50	2	10	5
GKD9	27	1.4	47	34
GKD10	>50	2	12	6
GKPD1	>50	>50	>50	
GKPD1b1	50	22	>50	
GKPD1b2	50	>50	>50	
