# Supplementary material for: A small molecule exerts selective antiviral activity by targeting the human cytomegalovirus nuclear egress complex
Source: PLoS Pathog. 2023 Nov 17;19(11):e1011781. doi: 10.1371/journal.ppat.1011781 (PMC10691697; doi:10.1371/journal.ppat.1011781)
Supplement: S4 Table — Of the libraries screened, two, LINCS2 and Selleck, contained ibrutinib. Each plate was screened in duplicate (i.e., Plates 3A and 3B, and plates 4A and 4B) and well number (i.e., 1, 2, 3, 4). The concentration of ibrutinib was 10 mM, 2 mM, 400 μM and 80 μM in LINCS2 wells 1 to 4 respectively, so the concentrations in the assay were 50, 10, 2, and 0.4 μM, respectively, and 10 mM in the Sellecks well for an assay concentration of 50 μM. Positive and negative controls were included on each plate. FRET emissions from the acceptor were measured at 665 nm (Channel 1) and donor emissions at 620 nm (Channel 2). The HTRF ratio was calculated as follows: [(Abs_665nm/Abs_620nm)*10,000]. The Selleck plate showed a narrower difference between the positive and negative controls than the LINCS plate, but the Z’ score was 0.6, with 14% and 32% inhibition observed. Essentially no inhibition was observed from any of the LINCS wells. (PDF) [file ppat.1011781.s015.pdf]

Plate _Well	3A_1	3B_1	3A_2	3B_2	3A_3	3B_3	3A_4	3B_4	4A_1	4B_1
**Experimental**										
Library	LINCS2								Selleck	
Compound	Ibrutinib									
HTRF Channel 1 (665 nm)	28311	29201	31038	31679	28019	29138	30006	31815	7529	7468
HTRF Channel 2 (620 nm)	5736	5840	5859	6111	6125	5780	5791	5828	8631	9073
HTRF Ratio	49357	50002	52975	51839	45745	50412	51815	54590	8723	8231
**Positive Control**										
HTRF Channel 1 (665 nm)	7755	7906	7755	7906	7755	7906	7755	7906	6702	6602
HTRF Channel 2 (620 nm)	9029	9081	9029	9081	9029	9081	9029	9081	10035	9897
HTRF Ratio	8594	8707	8594	8707	8594	8707	8594	8707	6787	6776
**Negative Control**										
HTRF Channel 1 (665 nm)	28217	28235	28217	28235	28217	28235	28217	28235	7418	7183
HTRF Channel 2 (620 nm)	5531	5672	5531	5672	5531	5672	5531	5672	8219	8063
HTRF Ratio	51077	49837	51077	49837	51077	49837	51077	49837	9028	8912

S4 Table. HTRF ratio and FRET emissions for ibrutinib-treated HCMV NEC.

Of the libraries screened, two, LINCS2 and Selleck, contained ibrutinib. Each plate was screened in duplicate (i.e., Plates 3A and 3B, and plates 4A and 4B) and well number (i.e., 1, 2, 3, 4). The concentration of ibrutinib was 10 mM, 2 mM, 400 μM and 80 μM in LINCS2 wells 1 to 4 respectively, so the concentrations in the assay were 50, 10, 2, and0.4 μM, respectively, and 10 mM in the Sellecks well for an assay concentration of 50 μM. Positive and negative controls were included on each plate. FRET emissions from the acceptor were measured at 665 nm (Channel 1) and donor emissions at 620 nm (Channel 2). The HTRF ratio was calculated as follows: [(Abs_665nm/Abs_620nm)*10,000]. The Selleck plate showed a narrower difference between the positive and negative controls than the LINCS plate, but the Z' score was

0.6, with 14% and 32% inhibition observed. Essentially no inhibition was observed from any of the LINCS wells.
