# Supplementary material for: A small molecule exerts selective antiviral activity by targeting the human cytomegalovirus nuclear egress complex
Source: PLoS Pathog. 2023 Nov 17;19(11):e1011781. doi: 10.1371/journal.ppat.1011781 (PMC10691697; doi:10.1371/journal.ppat.1011781)
Supplement: S5 Table — (PDF) [file ppat.1011781.s016.pdf]

S5 Table List of primers used in this study

Construct	Forward primers	Reverse Primers
UL50_C35N	5'CGGCCTCATCAATAAAAACC CCAATTACTCGGTGTGC 3'	5' GCATCGGTGACGCGCAGC 3'
UL50_C54S	5'CACGGTCTATAGTGTCGAGT ATC 3'	5' TCTGTCTTGAGCATGGCG
UL50_C79S	5' AAACACTGGCTCCGCCGT CTCCC 3'	5' TTAAAGATAAAACAA GGCACGTGGTCTG 3'
UL53_C214S	5' GCACATCCCCAGCGACTGC ATCACG 3'	5' ACGTCGGGGTTTCGAAG ATGAC 3'
UL53_C159S	5' TTACGGGTTCAGTAAG AGCAGCGAG 3'	5' TAAAAGTTTCTATTTTT CATGATGTTGGACG 3'
UL53_C288S	5' TCAGGAGCTGAGTCAGTC CAGCGGCC 3'	5' ATGAGCTCTTTGTAGC GCTCAAAGGAC 3'
BAC_UL53-C214S	5' CACATGCACGTCATCTTCGA AAACCCCGACGTGCACATCCC CAGCGACTGCATC TAGGGATAACAGGGTAATCGA TTT 3'	5' GTCTTCGCGCGCCGCCG TGAGCATCTGCGTGATGCAG TCGCTGGGGATGTG GCCAGTGTTACAACCAATTA ACC 3'
BAC_UL53-S214C	5'CACATGCACGTCATCTTCGA AAACCCCGACGTGCACATCCC CTGCGACTGCATCTAGGGATA ACAGGGTAATCGATTT 3'	5'GTCTTCGCGCGCCGCCGTGAGC ATCTGCGTGATGCAGTCGCAGGG GATGTGGCCAGTGTTACAAC CAATTAACC 3'
GST-Myc-UL53$^{50\text{-}292}$	Introducing Myc sequence (in two consecutive parts of five residues each) into GST- UL53$^{50\text{-}292}$:	
	Part 1: 5'GGATCCATGGAGCAGAAGTT GATCCCGTCG CCG3'	5' CGGCGACGGGATCAAC TTCTGCTCCATGGATCC 3'
	Part 2: 5'GAAGTTGATCTCGGAGGAGG ACCTGCCGTCGCCG3'	5' CGGCGACGGCAGGTCCT CCTCCGAGATCAACTTC 3'
GST-Myc-G/S linker – UL53$^{50\text{-}292}$	Introducing a 13 glycine-serine linker sequence into GST-Myc-UL53$^{50\text{-}292}$ in three parts:	

	Part 1:<br>5'GAGGAGGACCTGGGAGGAG GAGGCCCGTCGCCG3'	5' CGGCGACGGGCCTCCTCC TCCCAGGTCCTCCTC 3'
	Part 2:<br>5'GGAGGAGGAGGCTCAGGAG GAGGACCGTCGCCG3'	5' CGGCGACGGTCCTCCTCC TGAGCCTCCTCCTCC 3'
	Part 3:<br>5' GGAGGTGGAGGAGGAGGAG GAGGACCGTCGCCG3'	5' CGGCGACGGTCCTCCTCC TCCTCCTCCACCTCC 3'
His- UL50$^{1-169}$ E56A	5' GTCTATTGTGTCGCGTATC TACTCAG 3'	5' CTGAGTAGATACGCGACACA ATAGAC 3'
His- M50$^{1-168}$	Primers for transferring the M50 gene from a previous plasmid to the pET15b vector	
	5'CAGAATGCTGGTCATATGAT GGAGATC 3'	5'CTACGCGTAAGAATTCTCGAC GTC3'
GST-Myc-G/S linker – M53$^{103-333}$	Generating linearized GST-Myc-G/S linker plasmid for InFusion-based cloning	
	5' CCG TCG CCG GCC GAC GCG CGC 3'	5' ACC ACC ACC TGA ACC TCC ACC 3'
	Generating M53 insert for InFusion-based cloning into GST-Myc-G/S linker plasmid	
	5' GGT TCA GGT GGT GGT ATG GCT CCG GAA AG 3'	5' GTC GGC CGG CGA CGG TTA TTA CAG G 3'
GST-Myc-G/S linker – UL30 peptide	Generating UL30 peptide insert for InFusion-based cloning into GST-Myc-G/S linker plasmid	
	5' GGT TCA GGT GGT GGT ATG GAG GAC GTG 3'	5' GTC GGC CGG CGA CGG TTA TTA TGC TAG 3'
