# Supplementary material for: A small molecule exerts selective antiviral activity by targeting the human cytomegalovirus nuclear egress complex
Source: PLoS Pathog. 2023 Nov 17;19(11):e1011781. doi: 10.1371/journal.ppat.1011781 (PMC10691697; doi:10.1371/journal.ppat.1011781)
Supplement: S1 Text — (PDF) [file ppat.1011781.s017.pdf]

# S1 Text

## Design and testing of GK1 analogs

To see if we could improve upon the potency and selectivity of GK1 and gain information about structure-activity relationships, we tested a number of analogs of GK1 and GK2 (S6 Fig.) for inhibition of NEC interactions in vitro and for antiviral activity and cytotoxicity (S3 Table, S7and S8 Figs.).

As GK1 and GK2 came from a kinase inhibitor library and contain certain groups, such as the 7-azaindole NH that makes interactions with the hinge region of protein kinases, we hypothesized that their cytotoxicities might be due at least in part to inhibition of host kinases. Three compounds, a2GK2, GKD1, and GKD2 were synthesized to address this potential off-target issue. a2GK2 installs a methyl group and replaces the ether linkage with an amine, while GKD1 contains just the methyl substitution, and GKD2 just the amine replacement of the ether. However, both compounds where the ether was replaced by an amine (a2GK2 and GKD2) displayed no detectable inhibitory activity against NEC subunit interactions in the HTRF assay, and although they had antiviral potencies similar to that of GK2, they were more cytotoxic than GK2 (S3 Table). Thus, the ether linkage appears to be an important determinant for in vitro activity and for reducing cytotoxicity. GKD1, with just the methyl substituent, retained activity against NEC subunit interactions in the HTRF assay, but was, if anything, less potent for antiviral activity and more cytotoxic.

The remaining compounds were designed to modify different parts of GK1 or GK2 (S6 Fig). Of these, three compounds retained potency in both the HTRF and antiviral assays; GKD4, GKD6, and GKD9.

GKD4, which replaces the Cl or H, respectively, of the GK1 or GK2 7-azaindole with a methyl group, exhibited an inhibitory activity intermediate between the two original compounds against NEC subunit interactions (without affecting P30-UL42 interactions) in the HTRF assay (S7A Fig.) and for inhibition of HCMV in HFF cells (S3 Table, S7A Fig). Using CovDock, it is predicted to interact with the Cys214 pocket similarly to GK1. Its CovDock docking score (-4.095), similar to GK1's was predictive of stronger binding than GK2, consistent with it being more potent than GK2. Its cytotoxicity was not meaningfully different from that of GK1 or GK2. These results suggested some leeway for modifications of the 7-azaindole at this position; however, adding a more bulky substituent in the GK-PD series of analogs (S6 Fig.) reduced both in vitro activity and antiviral potency. A different modification of the 7-azaindole, incorporation of an additional nitrogen in the heterocycle (GKD5), eliminated potency in the in vitro assay and reduced antiviral potency and the $CC_{50}/ED_{50}$ ratio.

Another analog GKD6, in which the acrylamide was changed from the meta- position in GK2 to the para- position, also exhibited potencies intermediate between GK1 and GK2 for disruption of NEC subunit interactions in the HTRF assay and antiviral activity, with reduced cytotoxicity in the cell-based assays, resulting in a 3-fold higher $CC_{50}/ED_{50}$ ratio than that of GK2, but still lower than that of GK1 (S3 Table, S7A Fig.). Using CovDock, it is predicted to interact with the same residues as GK2, but its docking score (-3.77) was predictive of stronger binding, consistent with its greater potencies in the HTRF and antiviral assays compared with GK2. It may be that entropic effects due to the shift in position of the acrylamide account for the predicted stronger binding. GKD6's lack of a

chlorine or methyl in the 7-azaindole ring might contribute to its lower potency than GK1 and GKD4 in the HTRF assay, although that effect is less than 2-fold.

We combined the modifications in GKD4 and GKD6, yielding GKD9. This compound was less potent than GK1 and slightly less potent than GK2 for disruption of NEC subunit interactions in the HTRF assay. Despite that, it exhibited slightly more potent antiviral activity than any of the compounds except GK1, and less cytotoxicity than GK1, GK2, GKD4, and GKD6, resulting in a $CC_{50}/ED_{50}$ ratio of 34, slightly higher than that of GK (S3 Table, S7A Fig.).

We then tested whether these analogs inhibited NEC formation by targeting cysteine 214 of UL53 using HTRF assays. As was the case for GK1 and GK2, the C214S substitution essentially eliminated inhibition of NEC subunit interactions by GKD4 and GKD6 in HTRF assays, with either no or only modest effects of the other substitutions (S7B Fig.), validating the CovDock analysis. To our surprise, however, inhibition of NEC subunit interactions in HTRF assays by GKD9 was not greatly reduced by the C214S substitution; instead every substitution tested resulted in modest decreases in inhibition (S7B Fig.). Perhaps, this difference in how GKD9 interacts with the NEC relative to the other four compounds may be consistent with the disconnect between potencies in the HTRF and antiviral assays. Oddly, GKD7, which contains the Cl moiety of GK1 and the para position of the acrylamide, was much less potent than GK1 in both the in vitro and antiviral assays, and less potent than GK2 also, and a version of GKD9 in which the acrylamide is modified was also not active.

Since UL53 residue C214 was critical for the inhibitory activity of GKD4 and GKD6 in vitro, we further tested whether an HCMV mutant containing the C214S substitution is

resistant to GKD4 and GKD6. Using the automated plaque reduction assay, we found that the $ED_{50}$'s of both compounds for the C214S mutant were significantly higher than WT virus (S8 Fig).

As discussed above, none of the other analogs (S6 Fig.) exhibited either more potent activity than GK1 for inhibition of NEC interactions in the HTRF assays or against HCMV in infected cells (S3 Table). Two compounds in the Gray Kinase library that contain an azaindole-ether-phenyl-acrylamide scaffold (S11 Fig.) but contain bulky modifications did not score as hits, which may provide clues to other modifications that reduce activity.

Although the modifications of GK1/GK2 described here were not sufficient to improve potency and selectivity while maintaining the same UL53 C214-dependent mechanism, this information may provide a starting point for future structure-activity relationship studies.
