# Supplementary material for: A small molecule exerts selective antiviral activity by targeting the human cytomegalovirus nuclear egress complex
Source: PLoS Pathog. 2023 Nov 17;19(11):e1011781. doi: 10.1371/journal.ppat.1011781 (PMC10691697; doi:10.1371/journal.ppat.1011781)
Supplement: S1 Appendix — (PDF) [file ppat.1011781.s018.pdf]

S1 Appendix   List of libraries screened

**Library Name**
ActiMolTimTec1
Asinex1
Biomol4
BiomolICCBL-2012
Bionet2
BU-CMD 2017
Cayman Biolipid 1
CB GPCR
CB IONCore
CB KINACore
CB NHRBCore
ChemBridge3
ChemDiv1
ChemDiv2
ChemDiv3
ChemDiv4
ChemDiv5
ChemDiv6
ChemDiv7
EMD1

eMolecules 2014
Enamine1
Enamine2
Gray1
HME1
IFLab1
IFLab2
LifeChemicals1
LINCS1
LINCS2
LINCS3
LINCS4
LOPAC1
Maybridge3
Maybridge4
Maybridge5
Microsource1
MixCom5
MSDiscovery1
NCC1-2013
NCC2-2013
Prestwick2
Selleck-10mM

SYNthesis2
Tocris2
