# Supplementary material for: A small molecule exerts selective antiviral activity by targeting the human cytomegalovirus nuclear egress complex
Source: PLoS Pathog. 2023 Nov 17;19(11):e1011781. doi: 10.1371/journal.ppat.1011781 (PMC10691697; doi:10.1371/journal.ppat.1011781)
Supplement: S2 Appendix — (PDF) [file ppat.1011781.s019.pdf]

S2 Appendix   Compound characterization data

Confidential. For research only, Not for regulatory filing.

[redacted]

[redacted]

EW17394-9-P1D DMSO Bruker_A_400MHz

[redacted]

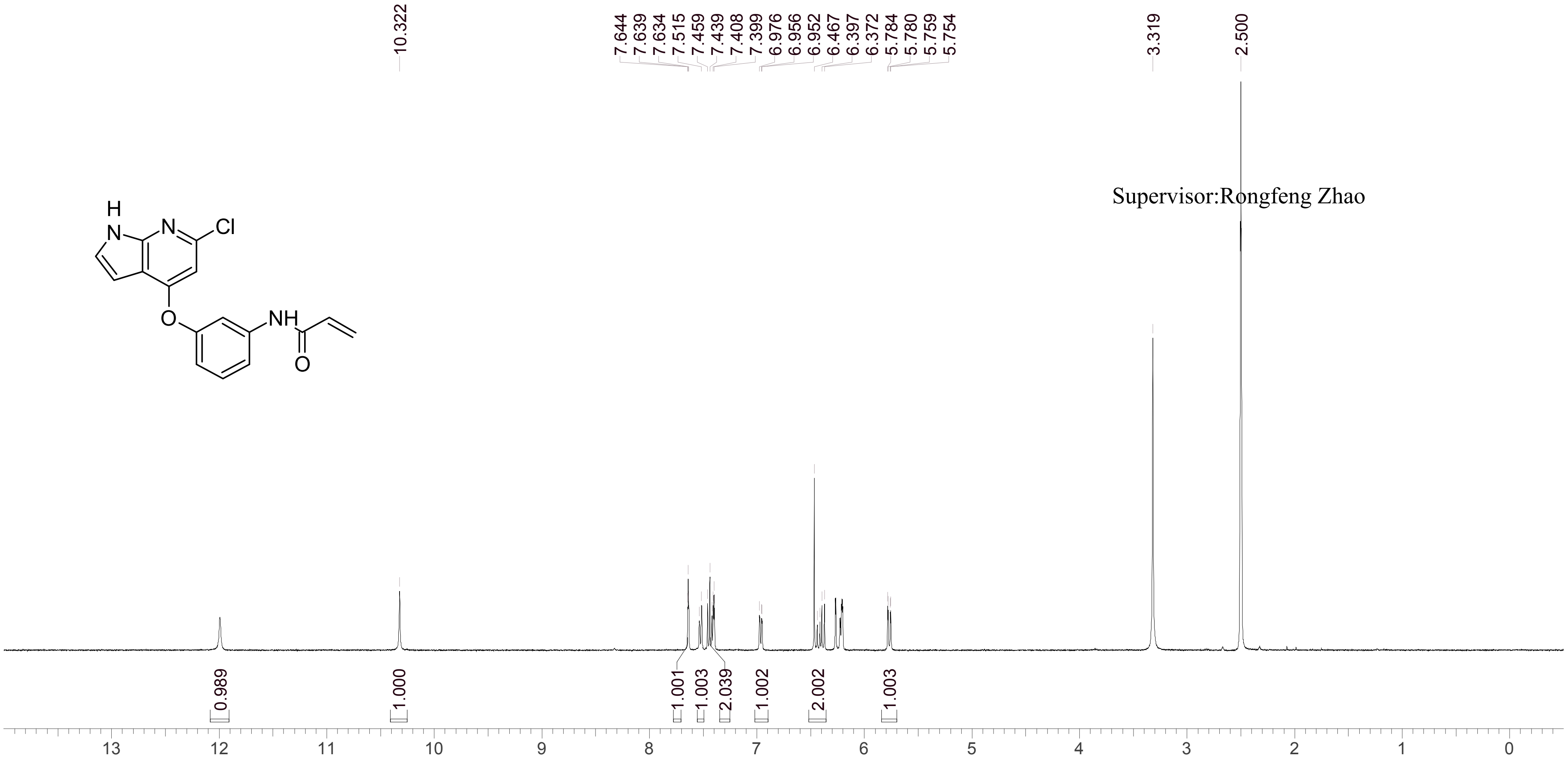

Supervisor:Rongfeng Zhao

Operator:

Date:

[redacted]

Confidential. For research information only

LCMS REPORT

Compound ID : GK1
Sample ID : EW17394-9-P1D1
Injection Vol : 0.5ul
Location : vial55
Acq Method : d:\method\5-95AB_R_220&254.lcm
Org DataFile : D:\DATA\1903\190327\EW17394-9-P1D1.lcd
Injection Date : 3/27/2019 1:59:53 PM
Instrument : LCMS-I

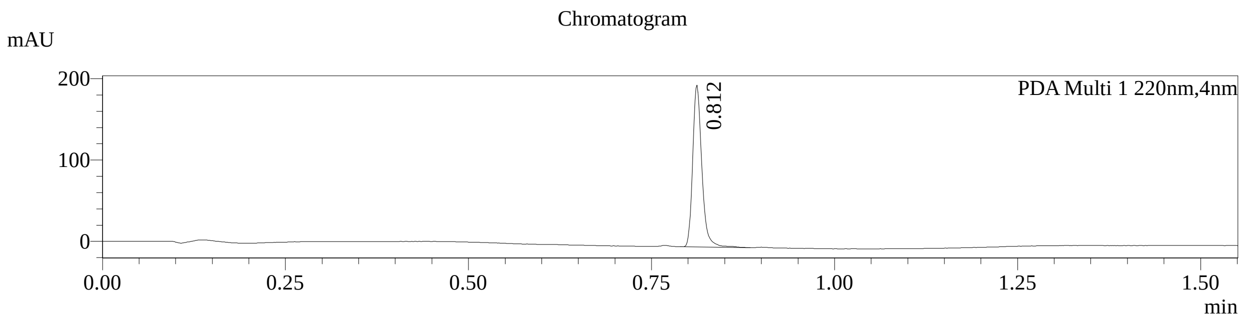

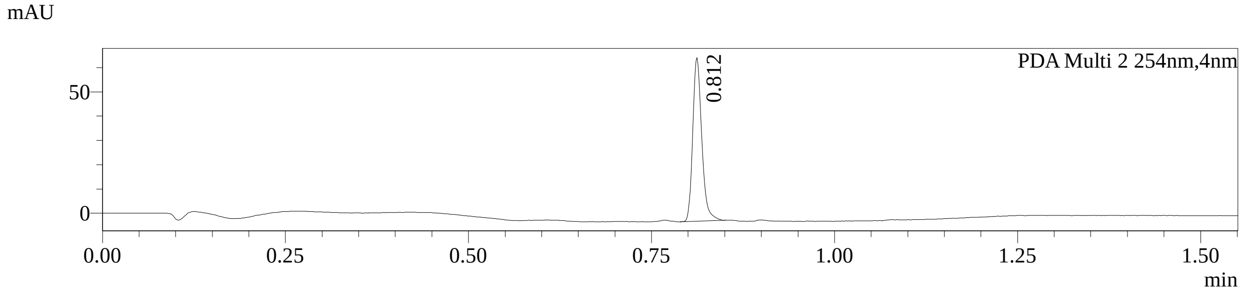

1 PDA Multi 1 / 220nm,4nm
2 PDA Multi 2 / 254nm,4nm

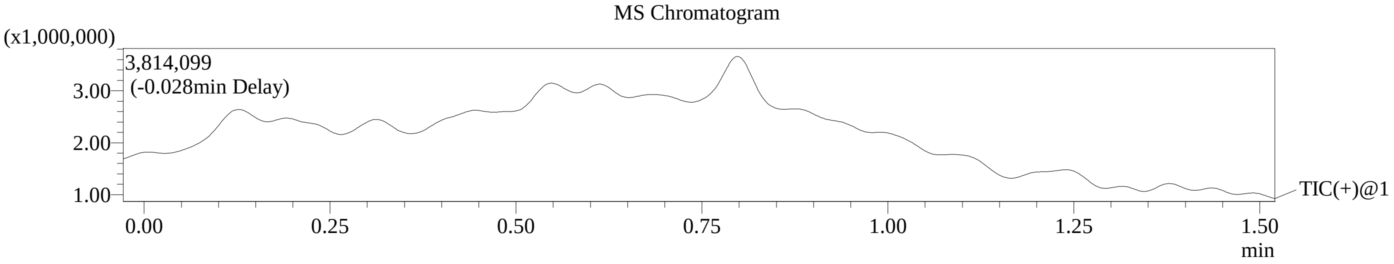

Integration Result

Peak Table

PDA Ch1 220nm

Peak#	Ret. Time	Height	Height%	USP Width	Area	Area%
1	0.812	199017	100.000	0.025	174854	100.000

Peak Table

PDA Ch2 254nm

Peak#	Ret. Time	Height	Height%	USP Width	Area	Area%
1	0.812	67326	100.000	0.025	58278	100.000

Confidential. For research information only

Operator:_______________

Date:___________________

Confidential. For research information only

Mass Spectrum

RetTime: 0.812 DateFile: D:\DATA\1903\190327\EW17394-9-P1D1.lcd

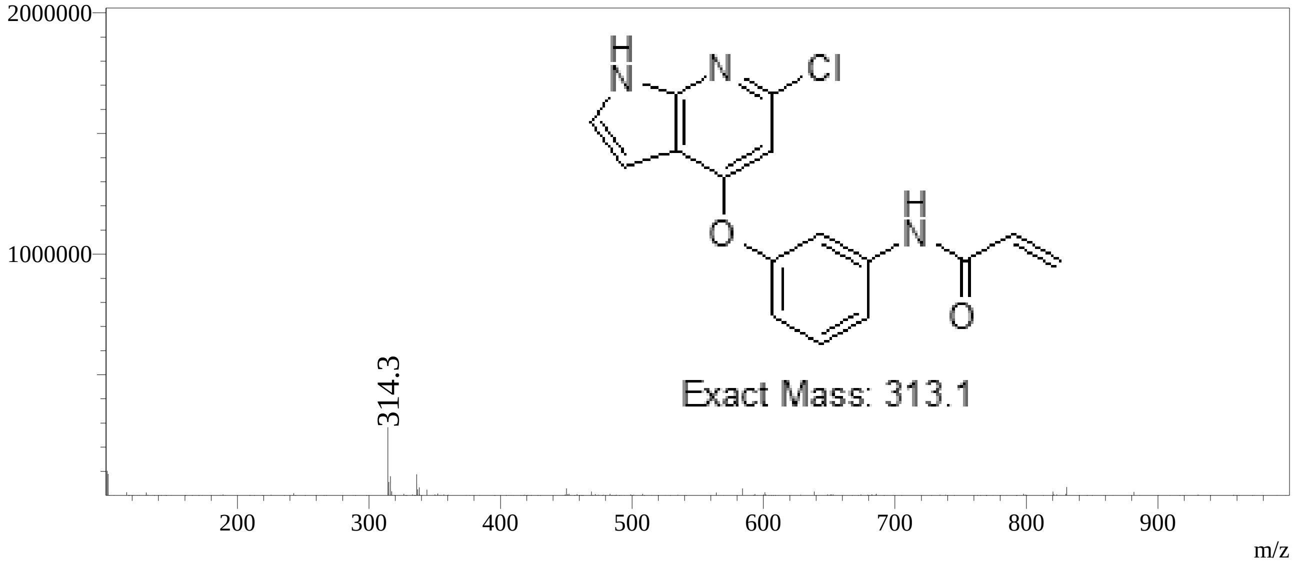

Confidential. For research information only

HPLC REPORT

Compound ID	: GK1
Sample ID	: EW17394-9-P1D
Vial#	: 23
Injection Volumn	: 1
Filename	: D:\DATA\2019\1903\190327\EW17394-9-P1D.lcd
Method Name	: D:\Method\10-80AB_4min.lcm
Instrument	: HPLC-P
Run time	: 3/27/2019 1:53:15 PM

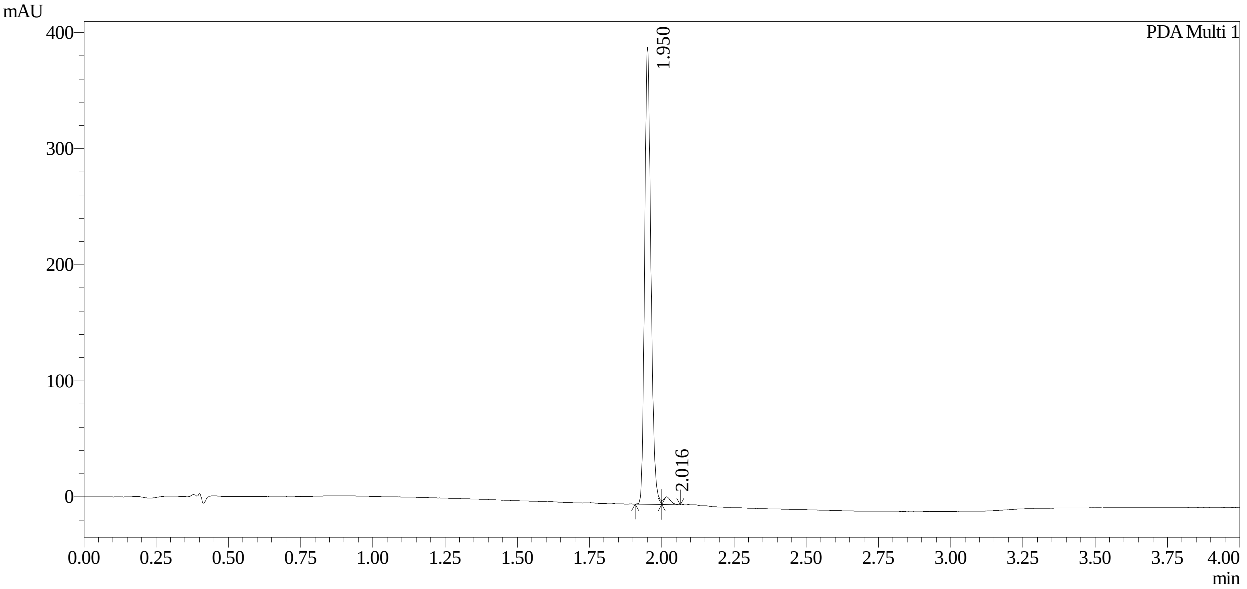

1 PDA Multi 1 / 220nm,4nm

Integration result

PeakTable

PDA Ch1 220nm

Peak#	Ret. Time	USP Width	Resolution	Height	Area	Area %
1	1.950	0.041	0.000	393492	594166	98.161
2	2.016	0.052	1.415	6597	11130	1.839
Total				400090	605296	100.000

Operater : ________________

Date : ________________

Confidential. For research information only

HPLC REPORT

Compound ID : GK1
Sample ID : EW17394-9-P1D
Filename : D:\DATA\2019\1903\190327\EW17394-9-P1D.lcd
Method Name : D:\Method\10-80AB_4min.lcm
Instrument : HPLC-P
Run time : 3/27/2019 1:53:15 PM

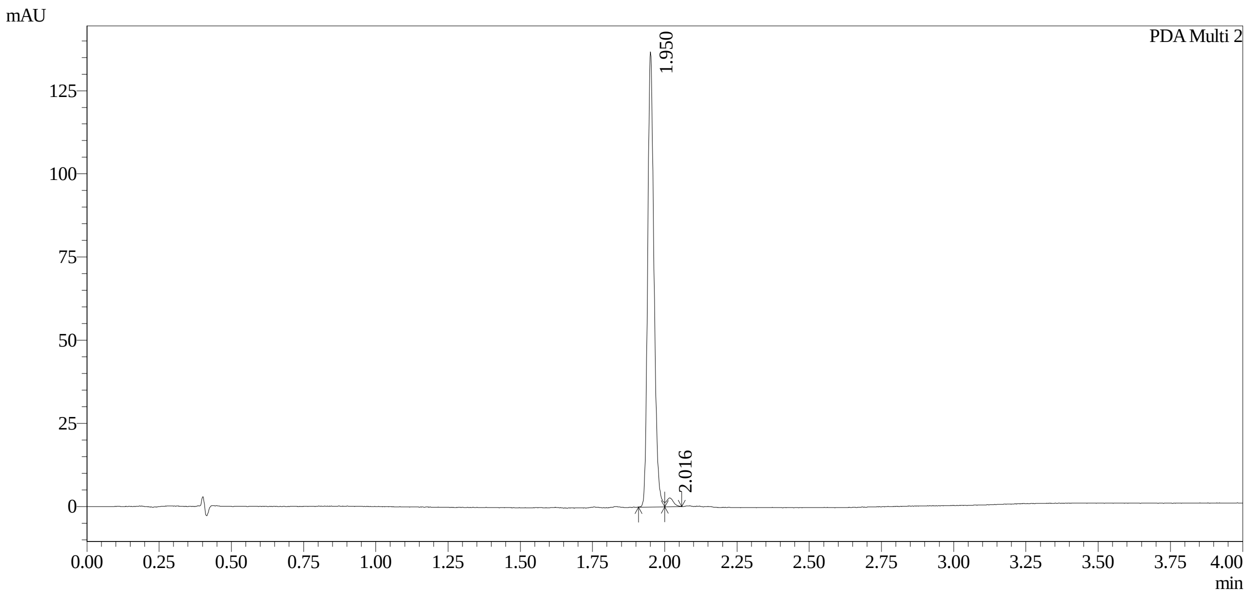

1 PDA Multi 2 / 254nm,4nm

Integration result

PeakTable

PDA Ch2 254nm

Peak#	Ret. Time	USP Width	Resolution	Height	Area	Area %
1	1.950	0.041	0.000	136824	206893	97.880
2	2.016	0.052	1.431	2683	4482	2.120
Total				139507	211374	100.000

Operater : ________________

Date : ________________

Confidential. For research information only

HPLC REPORT

Compound ID : GK1
Sample ID : EW17394-9-P1D
Filename : D:\DATA\2019\1903\190327\EW17394-9-P1D.lcd
Method Name : D:\Method\10-80AB_4min.lcm
Instrument &Column : HPLC-P
Run time : 3/27/2019 1:53:15 PM

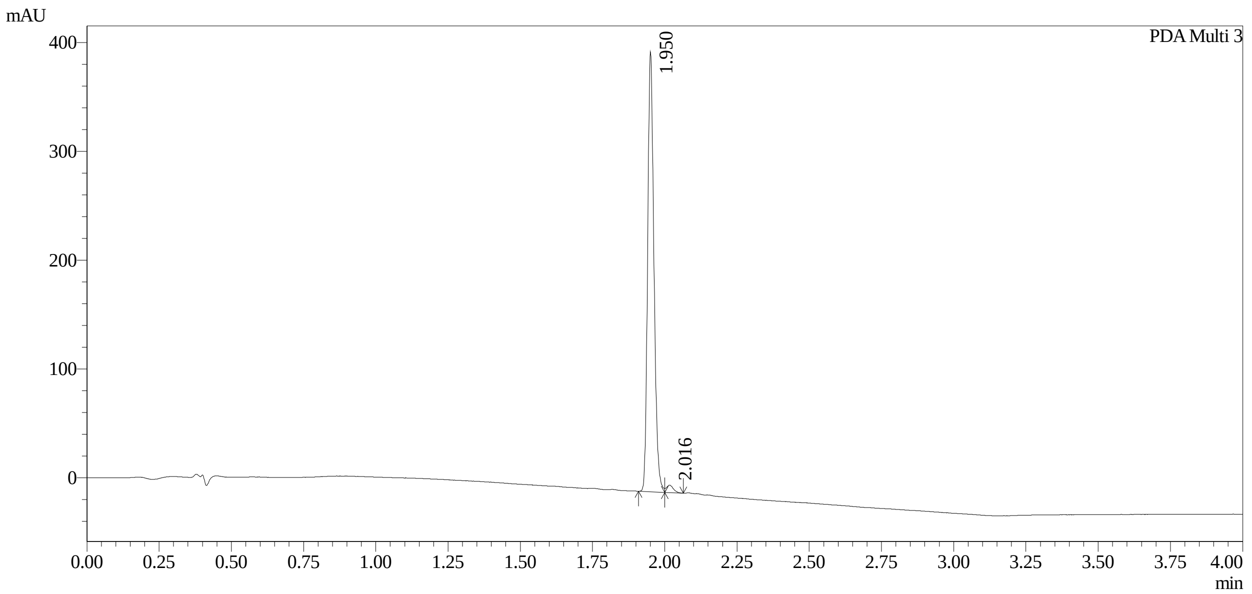

1 PDA Multi 3 / 215nm,4nm

Integration result

PeakTable

PDA Ch3 215nm

Peak#	Ret. Time	USP Width	Resolution	Height	Area	Area %
1	1.950	0.041	0.000	404407	610289	98.139
2	2.016	0.053	1.410	6954	11572	1.861
Total				411362	621862	100.000

Operater : ________________

Date : ________________

[redacted]

[redacted]

Certificate of Analysis

Product Name:	2-Propenamide, N-[3-(1H-pyrrolo[2,3-b]pyridin-4-yloxy)phenyl]-
Chemical Name:	2-Propenamide, N-[3-(1H-pyrrolo[2,3-b]pyridin-4-yloxy)phenyl]-

Cat. No.:	HY-103015	CAS No.:	1222776-76-2
Batch No.:	25811	Formula:	$C_{16}H_{13}N_3O_2$
M.Wt:	279.29	Appearance:	White to off-white (Solid)
Chemical Structure:		Storage:	Storage temp. 2-8°C

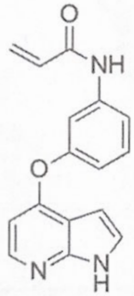

ANALYTICAL DATA

^{1}H NMR Spectrum:	Consistent with structure
LCMS:	Consistent with structure
Purity (LCMS):	98.17%
Conclusion:	The product has been tested and complies with the given specifications.

Caution: Product has not been fully validated for medical applications. For research use only.

[redacted]

Sample ID: CZ4538-015-1, Catalog No: HY-103015 Batch#25811 , DMSO-d6

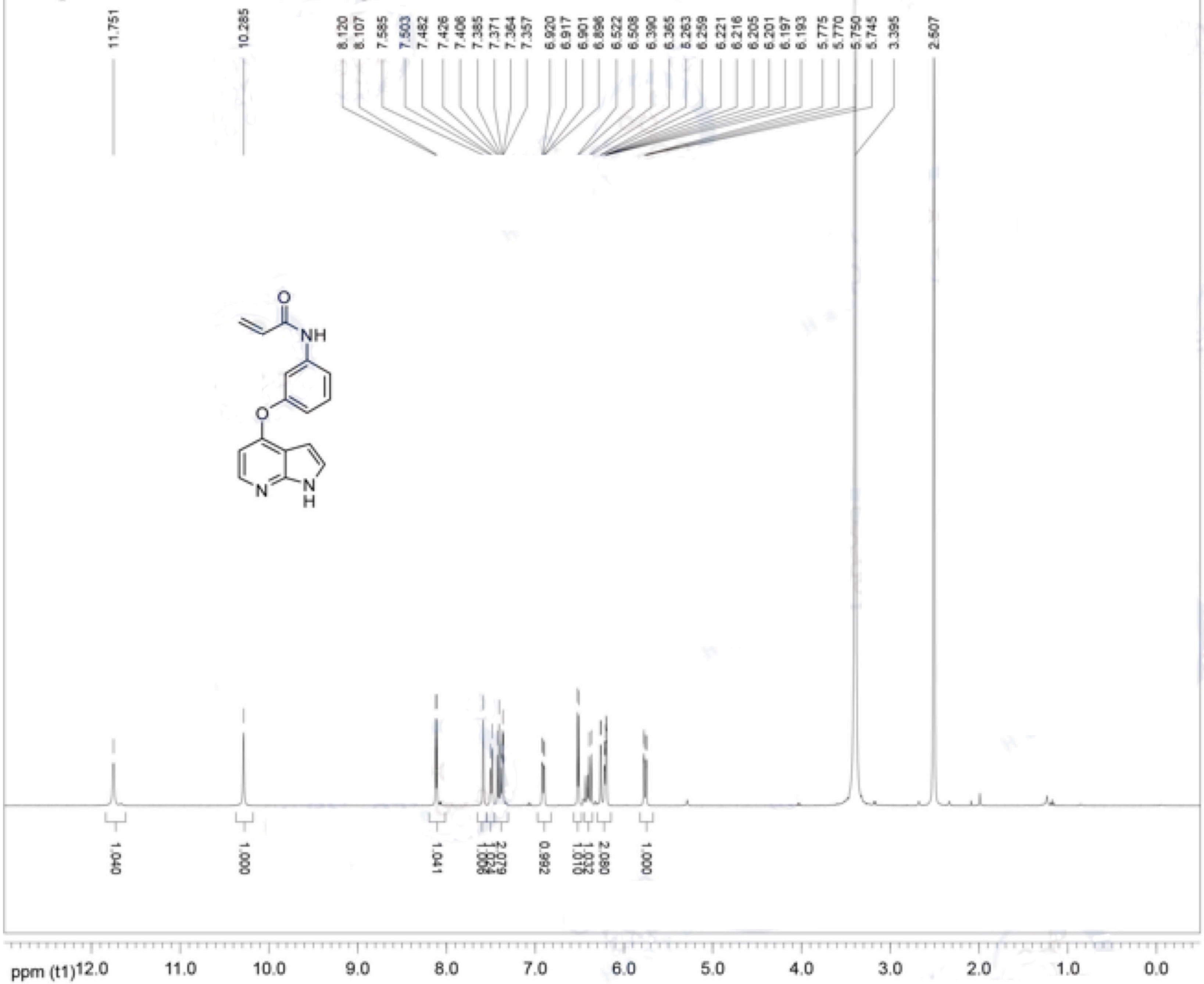

Date:
11 Aug 2017
Document's Title:
Catalog No: HY-103015 Batch#25811

Spectrum Title:
cz4538-015-1-dmso-170811

Frequency (MHz):
(f1) 400.130
Original Points Count:
(f1) 32768
Actual Points Count:
(f1) 32768
Acquisition Time (sec):
(f1) 3.2768
Spectral Width (ppm):
(f1) 24.992
Pulse Program:
Unknown

File ..0809-1 2017-08-09 12-48-50\CZ4538-015-1-1.D Tgt Mass (CHM):
Injection Date : 8 Aug 17 7:03 pm +0800 Seq. Line : 70
Sample Name : CZ4538-015-1_1 Location : P2-C-04
Acq. Operator : GHS_886 Inj : 1
Spec. Reported : MS Integration Inj Volume : 1 ul
Acq. Method : D:\chem32\1\DATA\20170809\20170809-1 2017-08-09 12-48-50\1-POS-3MIN.M
Analysis Method : D:\chem32\1\DATA\20170809\20170809-1 2017-08-09 12-48-50\1-POS-3MIN.M
Catalog No : HY-103015 Batch#25811 ARP-186
Method Info : Mobile Phase: A: water(0.01%TFA) B:ACN(0.01%TFA)
Gradient: 5% to 95%B within 1.3 min
Flow Rate :1.8ml/min
Column :SunFire C18, 4.6*50mm,3.5um A-RP-186
Oven Temperature : 45C

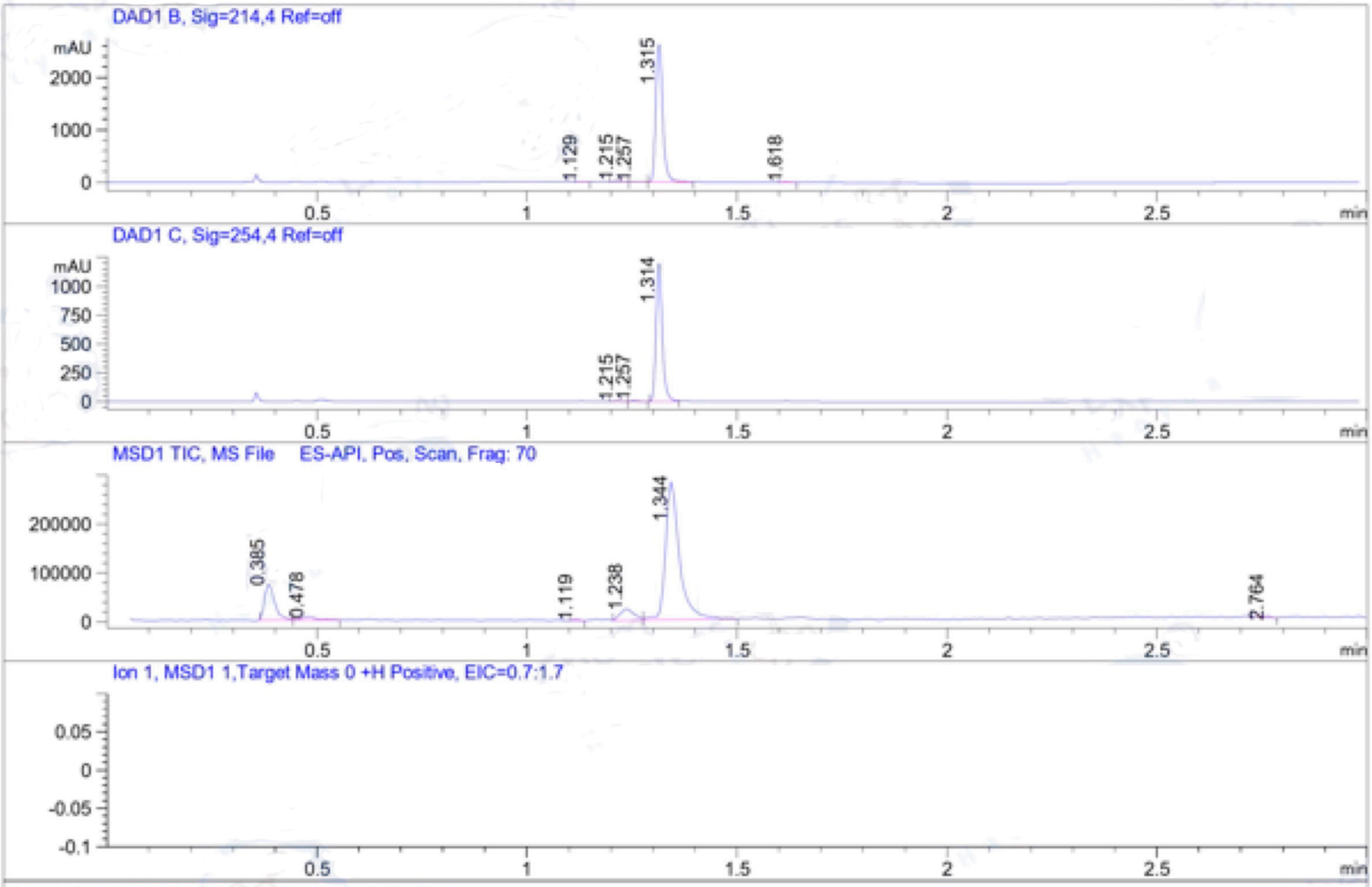

Integration Results for DAD1 B, Sig=214,4 Ref=off

RetTim	Width	Area	Height	Area%	MS(+)
1.13	0.01	5.42	5.91	0.18	ND
1.21	0.01	29.20	29.80	0.95	308
1.26	0.02	12.06	9.90	0.39	226
1.31	0.02	3024.11	2624.48	98.17	280
1.62	0.02	9.74	7.57	0.32	280

Integration Results for DAD1 C, Sig=254,4 Ref=off

RetTim	Width	Area	Height	Area%	MS(+)
1.21	0.02	6.42	6.24	0.52	308
1.26	0.02	11.27	10.26	0.91	226
1.31	0.02	1225.93	1197.16	98.58	280

Integration Results for MSD1 TIC, MS File

RetTim	Width	Area	Height	Area%	MS(+)
0.39	0.03	122125.49	74125.60	14.13	123

Ret. Time: 0.39 <<<< POSITIVE SPECTRA >>>>

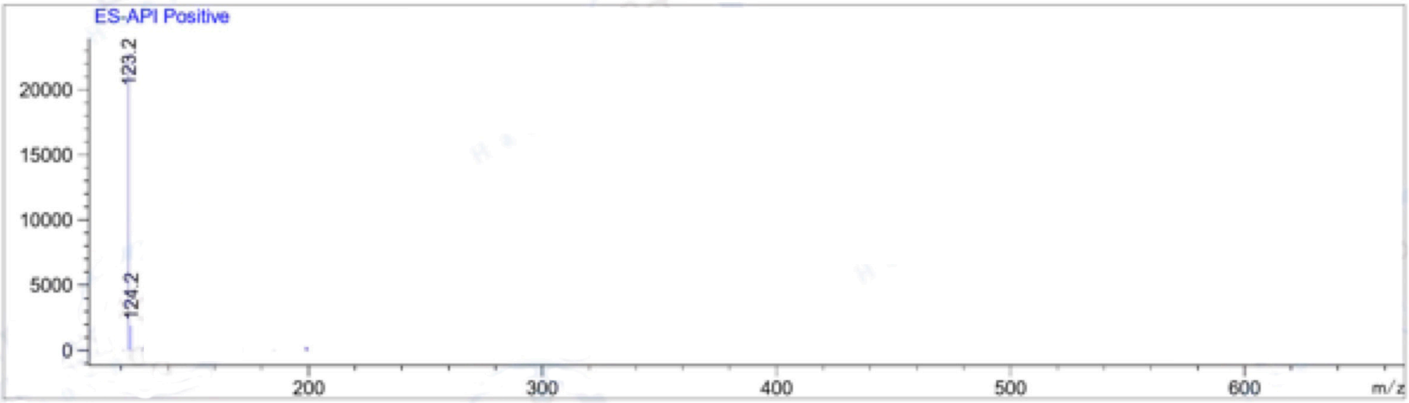

Ret. Time: 0.48

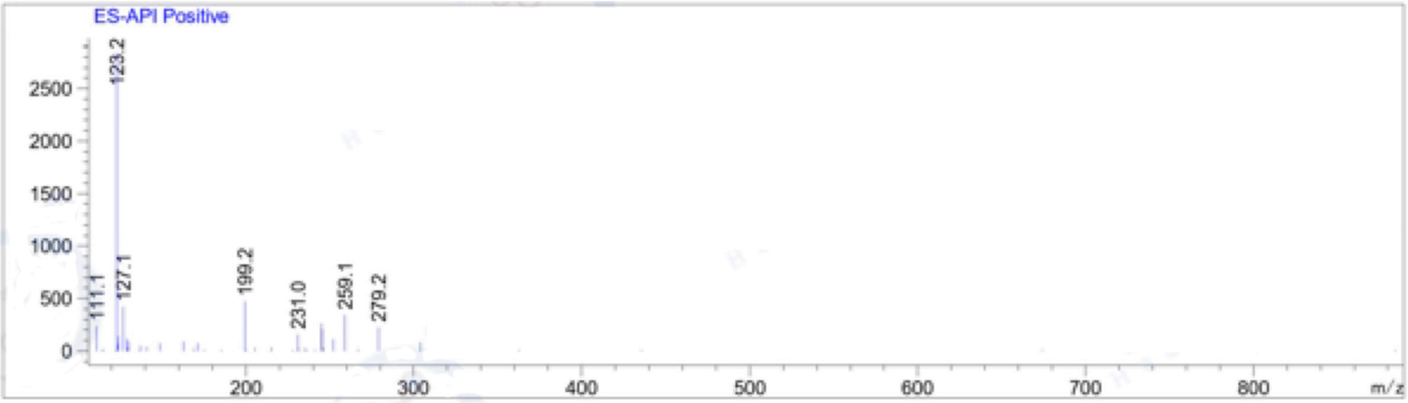

Ret. Time: 1.24

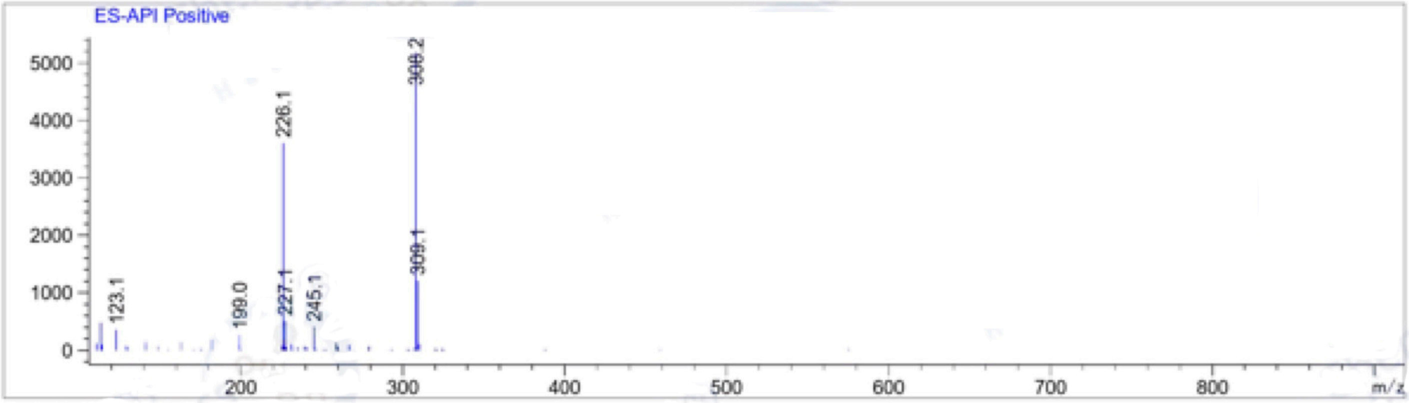

Ret. Time: 1.34

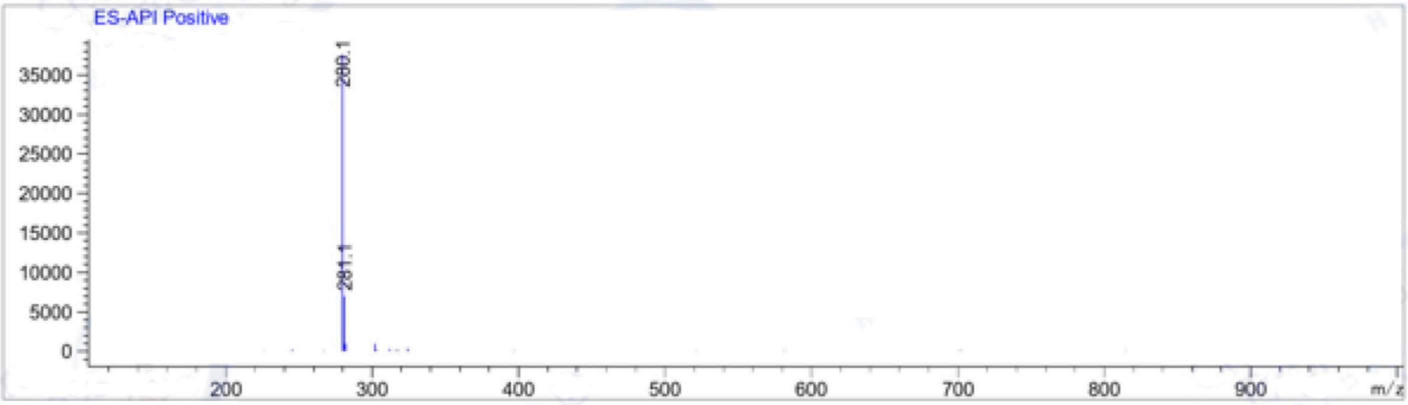

[redacted]

[redacted]

Certificate of Analysis

Product Name:	N-(3-((1H-pyrrolo[2,3-b]pyridin-4-yl)oxy)phenyl)propionamide
Chemical Name:	N-(3-((1H-pyrrolo[2,3-b]pyridin-4-yl)oxy)phenyl)propionamide

Cat. No.:	HY-103016		
Batch No.:	25812	Formula:	$C_{16}H_{15}N_3O_2$
M.Wt:	281.31	Appearance:	White to off-white (Solid)
Chemical Structure:		Storage:	Storage temp. 2-8°C

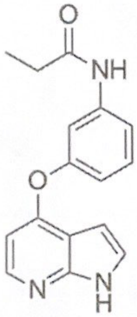

ANALYTICAL DATA

^{1}H NMR Spectrum:	Consistent with structure
LCMS:	Consistent with structure
Purity (LCMS):	98.01%
Conclusion:	The product has been tested and complies with the given specifications.

Caution: Product has not been fully validated for medical applications. For research use only.

[redacted]

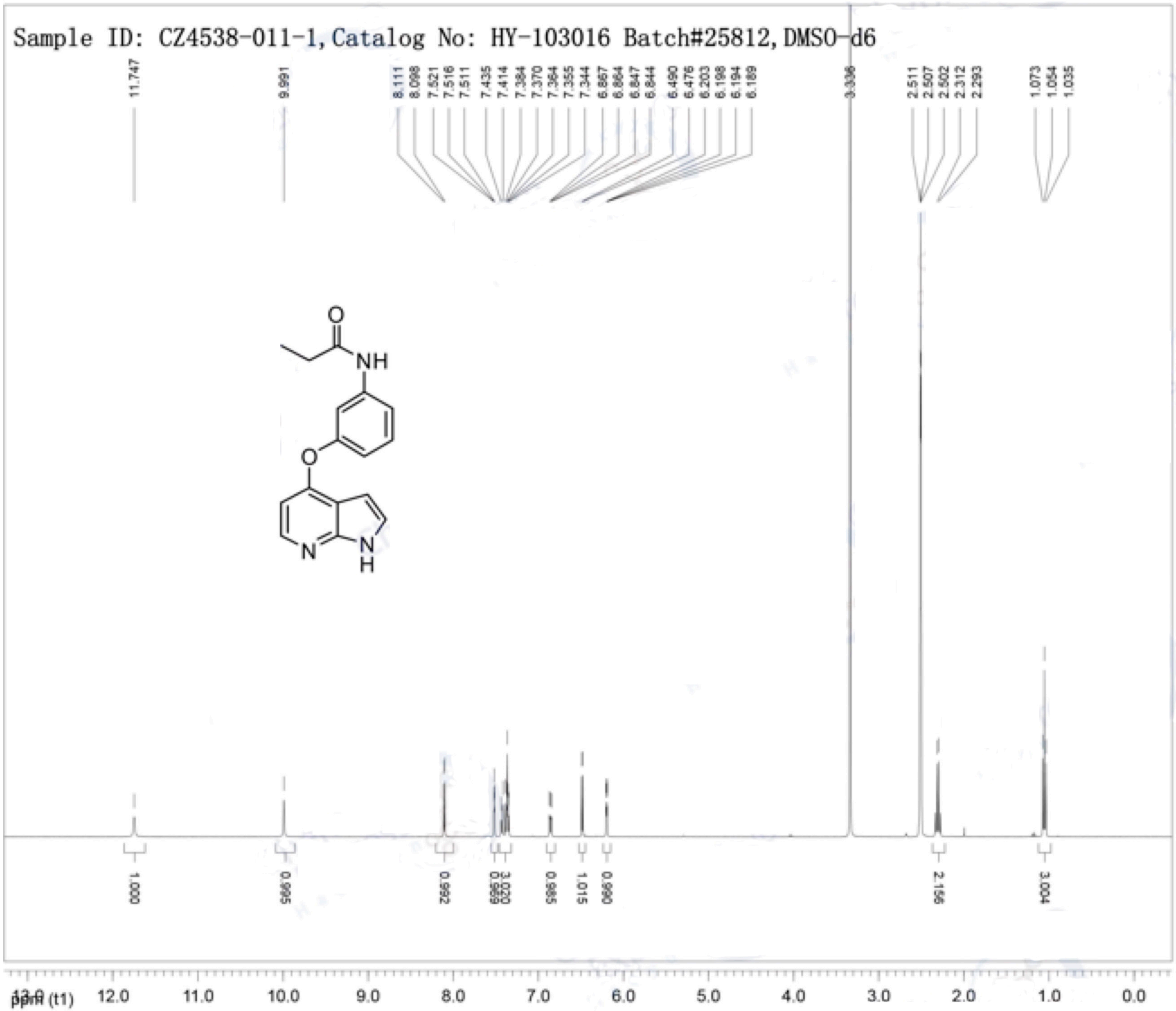

Date:
10 Aug 2017
Document's Title:
Catalog No: HY-103016 Batch#25812

Spectrum Title:
cz4538-011-1-dmso-170809

Frequency (MHz):
(f1) 400.130
Original Points Count:
(f1) 32768
Actual Points Count:
(f1) 32768
Acquisition Time (sec):
(f1) 3.2768
Spectral Width (ppm):
(f1) 24.992
Pulse Program:
Unknown

```
File ..0809-1 2017-08-09 14-16-54\CZ4538-011-1-4.D Tgt Mass (CHM):
Injection Date    :  9 Aug 17   5:58 pm +0800        Seq. Line : 41
Sample Name       : CZ4538-011-1-4              Location  : P2-B-08
Acq. Operator     : GHS_886                              Inj : 1
Spec. Reported    : MS Integration                 Inj Volume : 1 ul
Acq. Method       : D:\chem32\1\DATA\20170809\20170809-1 2017-08-09 14-16-54\1-POS-3MIN.M
Analysis Method   : D:\CHEM32\1\DATA\20170809\20170809-1 2017-08-09 14-16-54\1-POS-3MIN.M
Catalog No  : HY-103016 Batch#25812  ARP-186
Method Info : Mobile Phase: A: water(0.01%TFA) B:ACN(0.01%TFA)
              Gradient: 5% to 95%B within 1.3 min
              Flow Rate :1.8ml/min
              Column :SunFire C18, 4.6*50mm,3.5um A-RP-186
              Oven Temperature : 45C
```

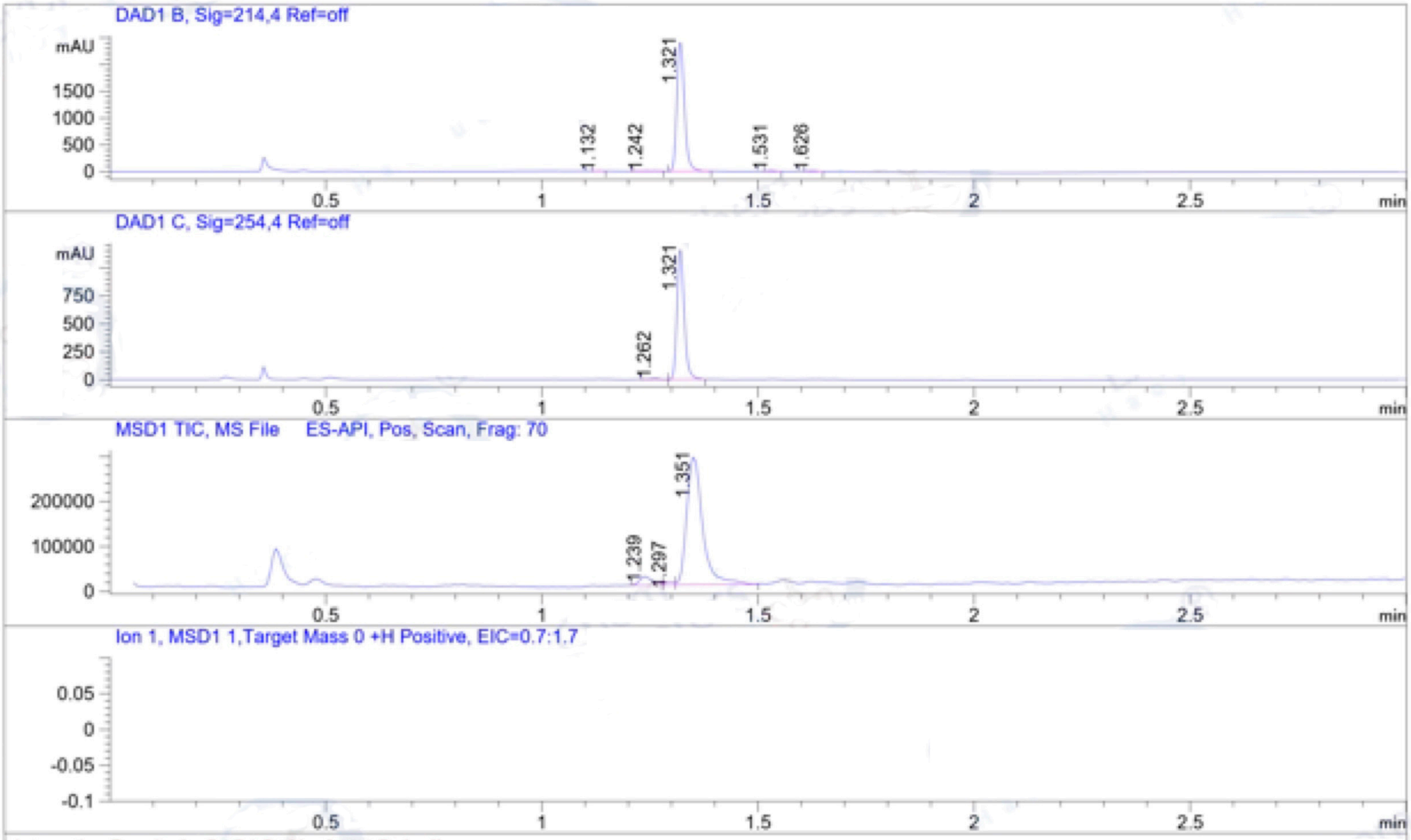

Integration Results for DAD1 B, Sig=214,4 Ref=off

RetTim	Width	Area	Height	Area%	MS(+)
1.13	0.01	4.61	5.36	0.15	149
1.24	0.04	23.58	9.25	0.79	308
1.32	0.02	2930.63	2405.42	98.01	282
1.53	0.02	17.03	14.14	0.57	149
1.63	0.02	14.36	10.60	0.48	149

Integration Results for DAD1 C, Sig=254,4 Ref=off

RetTim	Width	Area	Height	Area%	MS(+)
1.26	0.02	16.04	9.86	1.18	308
1.32	0.02	1343.84	1158.02	98.82	282

Integration Results for MSD1 TIC, MS File

RetTim	Width	Area	Height	Area%	MS(+)
1.24	0.03	41821.64	19873.00	5.43	308
1.30	0.02	9901.61	7198.52	1.29	149

Ret. Time: 1.24 <<<< POSITIVE SPECTRA >>>>

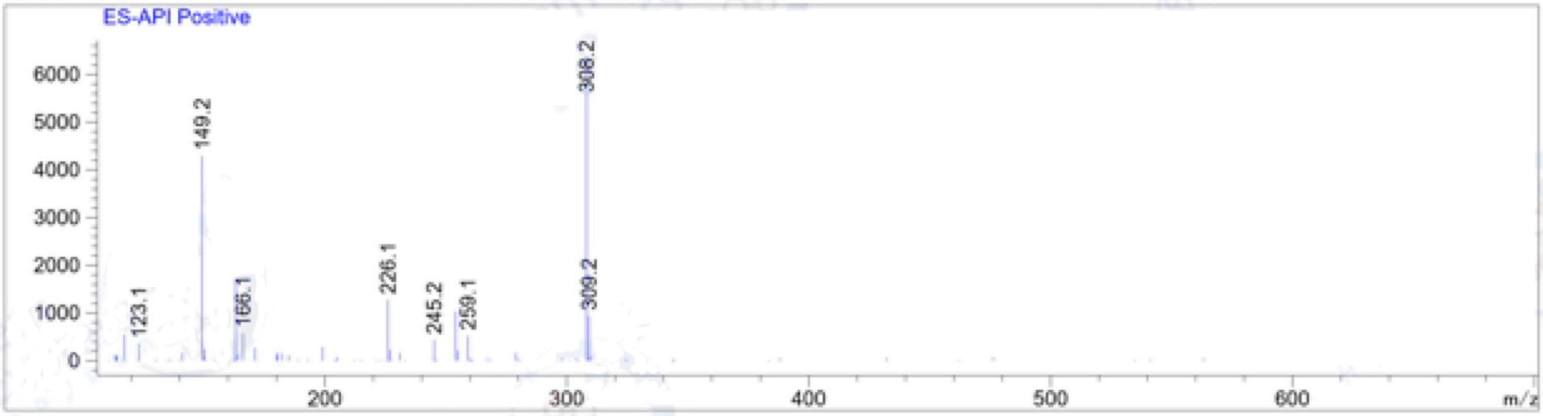

Ret. Time: 1.30

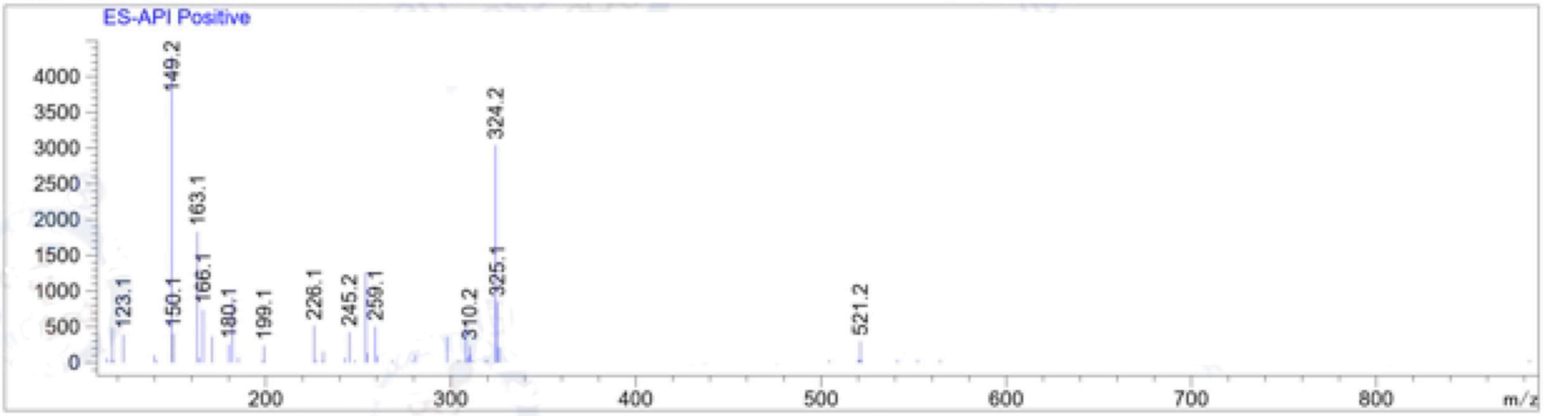

Ret. Time: 1.35

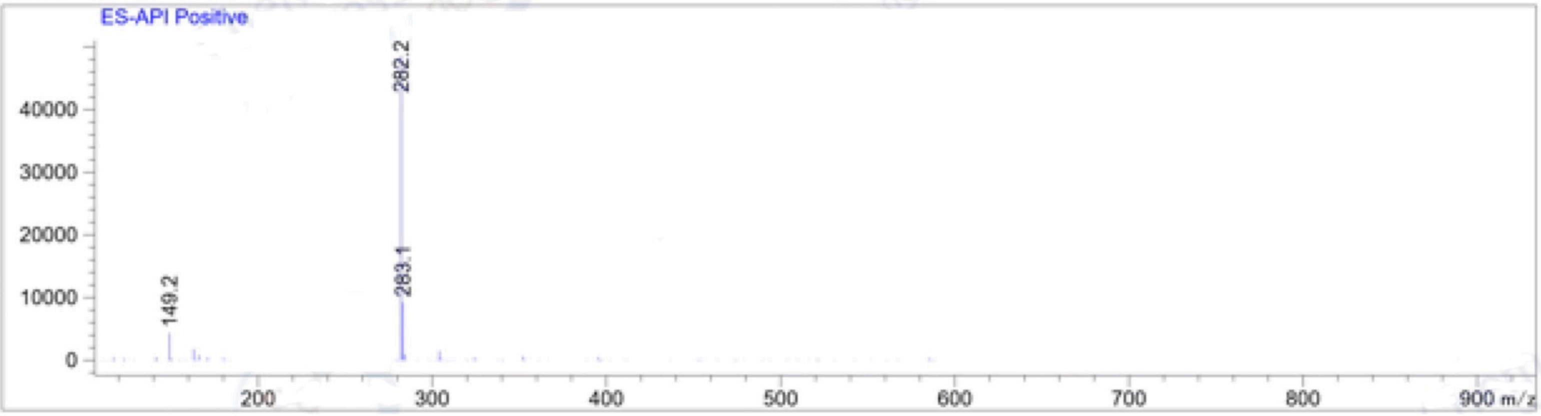

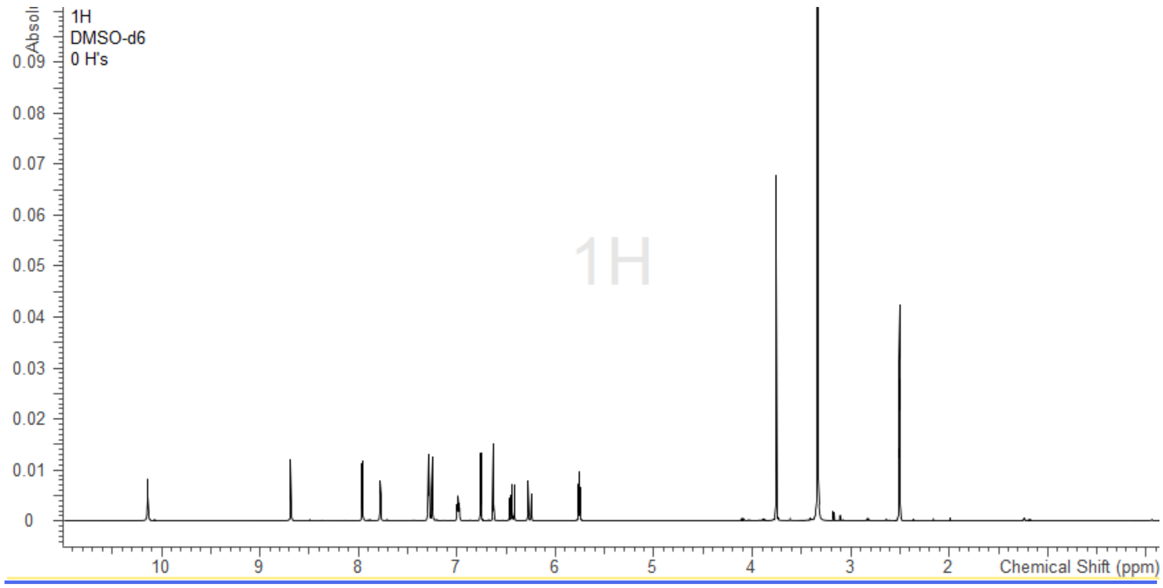

a2GK2 NMR data

Confidential. For research only, Not for regulatory filing.

[redacted]

Compound ID: GK-D1

EW14183-3-P1B CDCl3 Bruker_E_400MHz

Supervisor:Rongfeng Zhao

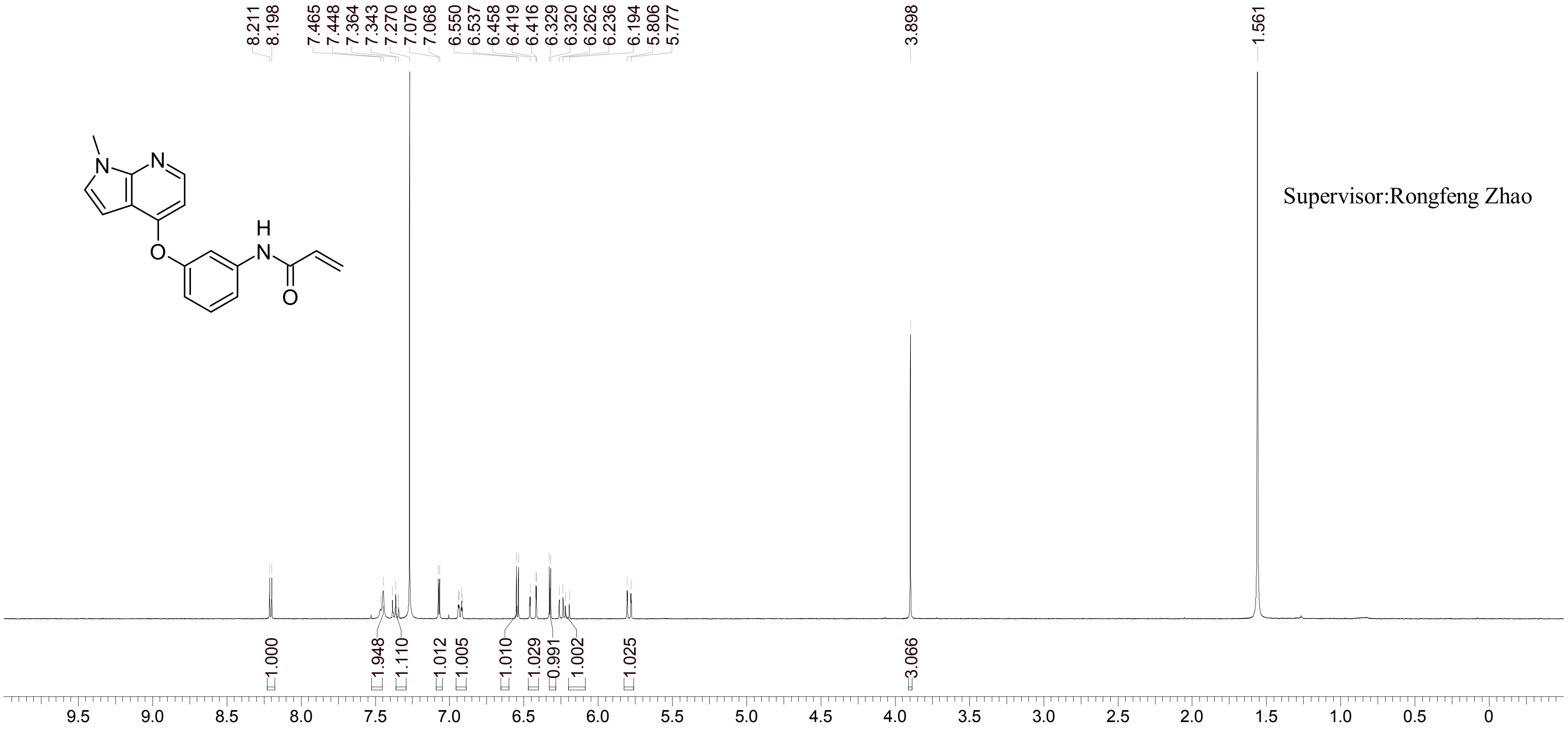

Operator:

Date:

[redacted]

LCMS REPORT

Compound ID : GK-D1
Sample ID : EW14183-3-P1E
Injection Vol : 5ul
Location : vial24
Acq Method : d:\method\5-95AB_R_220&254.lcm
Org DataFile : D:\DATA\1809\180905\EW14183-3-P1E.lcd
Injection Date : 2018-09-05 12:54:03
Instrument :LCMS-N 17-102

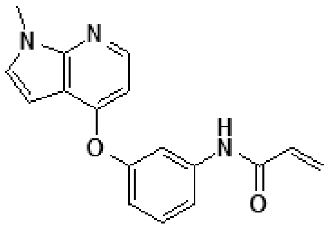

Exact Mass: 293.12

Chromatogram

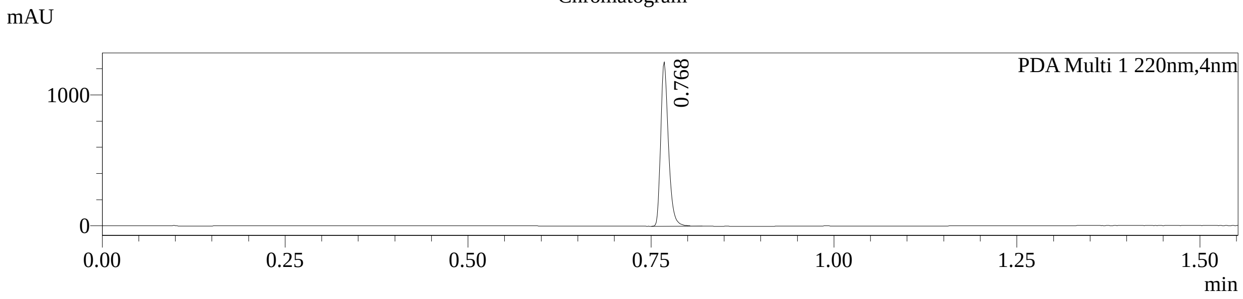

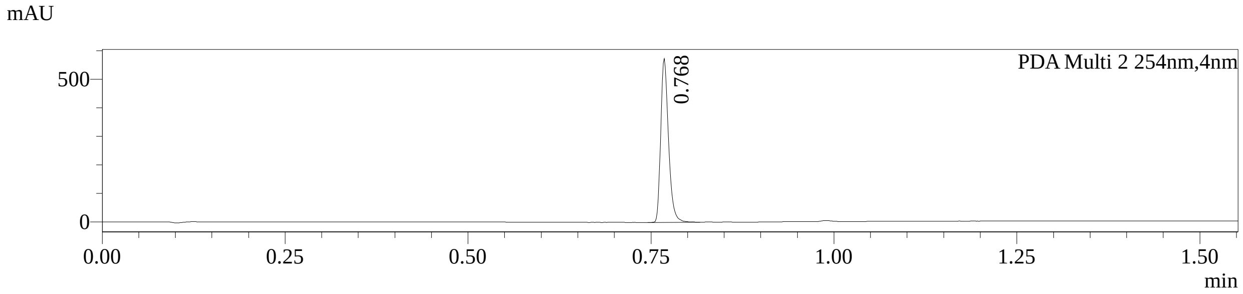

1 PDA Multi 1 / 220nm,4nm
2 PDA Multi 2 / 254nm,4nm

MS Chromatogram

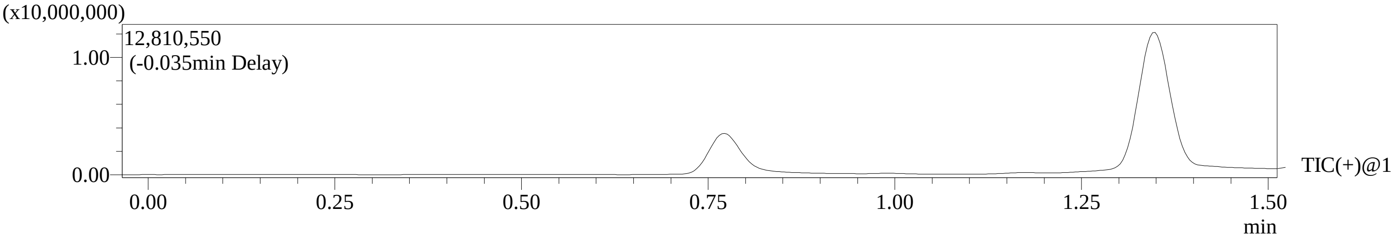

==

Integration Result

==

Peak Table

PDA Ch1 220nm

Peak#	Ret. Time	Height	Height%	USP Width	Area	Area%
1	0.768	1253708	100.000	0.022	920673	100.000

Peak Table

PDA Ch2 254nm

Peak#	Ret. Time	Height	Height%	USP Width	Area	Area%
1	0.768	573711	100.000	0.022	424724	100.000

Operator:_______________

Date:___________________

Confidential, for research only not for regulatory filing

Mass Spectrum
RetTime: 0.768 Datafile: D:\DATA\1809\180905\EW14183-3-P1E.lcd

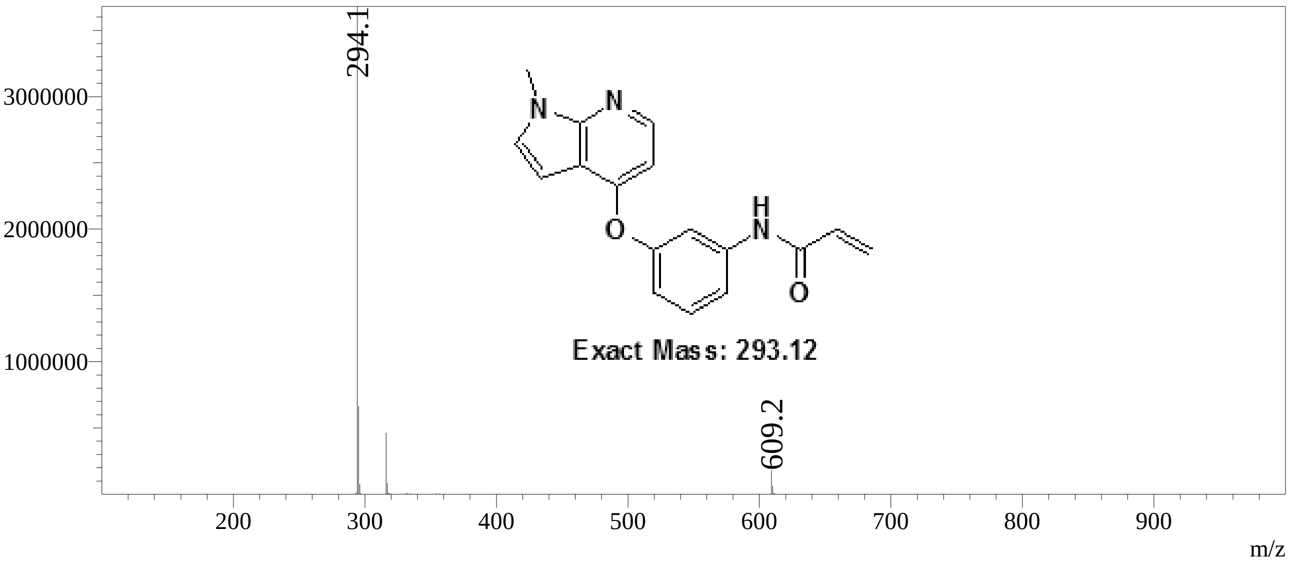

Confidential, for research only not for regulatory filing

HPLC REPORT

Compound ID : GK-D1
Sample ID : EW14183-3-P1B
Vial# : 41
Injection Volumn : 5
Filename : D:\DATA\2018\1809\180905\EW14183-3-P1B.lcd
Method Name : D:\Method\10-80AB_6min.lcm
Instrument : HPLC-N
Run time : 2018-09-05 13:53:48

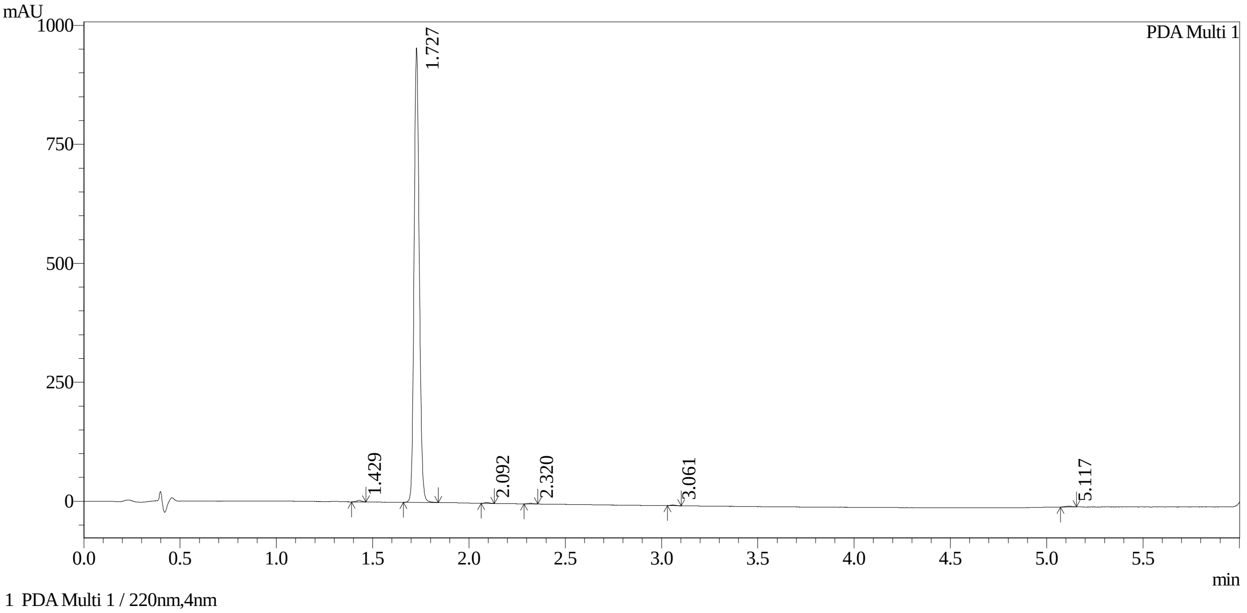

1 PDA Multi 1 / 220nm,4nm

Integration result

PeakTable

PDA Ch1 220nm

Peak#	Ret. Time	USP Width	Resolution	Height	Area	Area %
1	1.429	0.051	0.000	3055	5797	0.320
2	1.727	0.050	5.888	955119	1790025	98.960
3	2.092	0.050	7.285	1815	3367	0.186
4	2.320	0.050	4.541	1350	2406	0.133
5	3.061	0.056	13.970	1520	2953	0.163
6	5.117	0.066	33.658	1777	4282	0.237
Total				964637	1808830	100.000

Operater : ________________

Date : ________________

Confidential, for research only not for regulatory filing

HPLC REPORT

Compound ID : GK-D1
Sample ID : EW14183-3-P1B
Filename : D:\DATA\2018\1809\180905\EW14183-3-P1B.lcd
Method Name : D:\Method\10-80AB_6min.lcm
Instrument &Column : HPLC-N
Run time : 2018-09-05 13:53:48

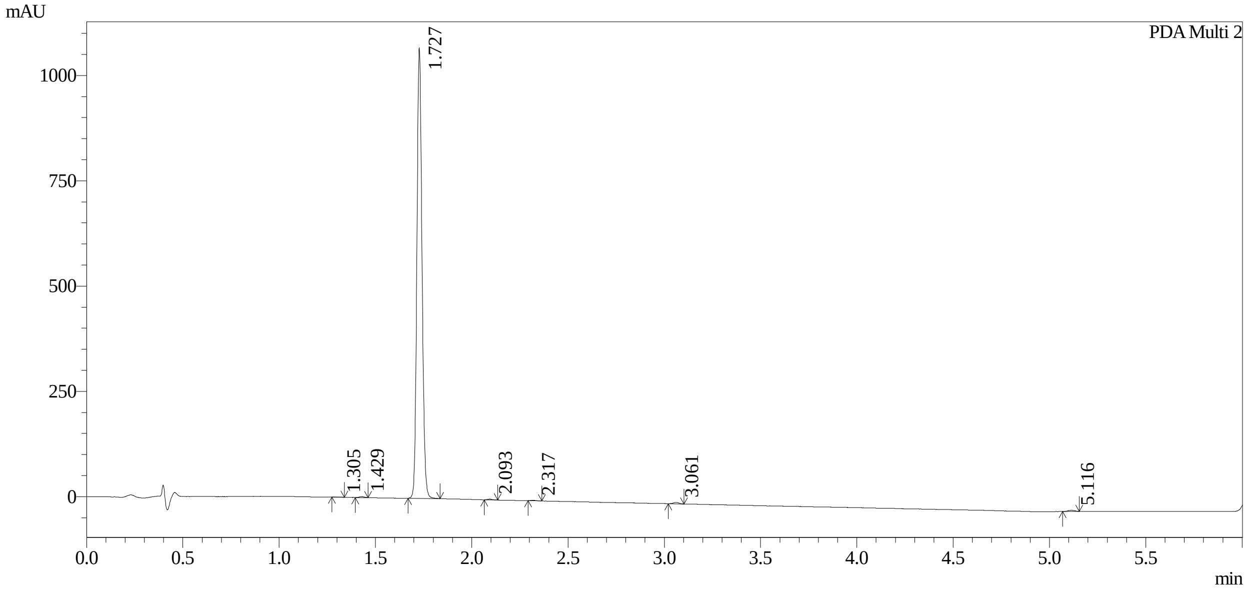

1 PDA Multi 2 / 215nm,4nm

Integration result

PeakTable

PDA Ch2 215nm

Peak#	Ret. Time	USP Width	Resolution	Height	Area	Area %
1	1.305	0.056	0.000	706	1402	0.070
2	1.429	0.050	2.320	2519	4568	0.227
3	1.727	0.050	5.950	1069954	1986426	98.664
4	2.093	0.048	7.460	1929	3447	0.171
5	2.317	0.051	4.507	1290	2463	0.122
6	3.061	0.056	13.947	3489	7144	0.355
7	5.116	0.067	33.584	3067	7873	0.391
Total				1082953	2013323	100.000

Operater : ________________

Date : ________________

Confidential, for research only not for regulatory filing

HPLC REPORT

Compound ID : GK-D1
Sample ID : EW14183-3-P1B
Filename : D:\DATA\2018\1809\180905\EW14183-3-P1B.lcd
Method Name : D:\Method\10-80AB_6min.lcm
Instrument &Column : HPLC-N
Run time : 2018-09-05 13:53:48

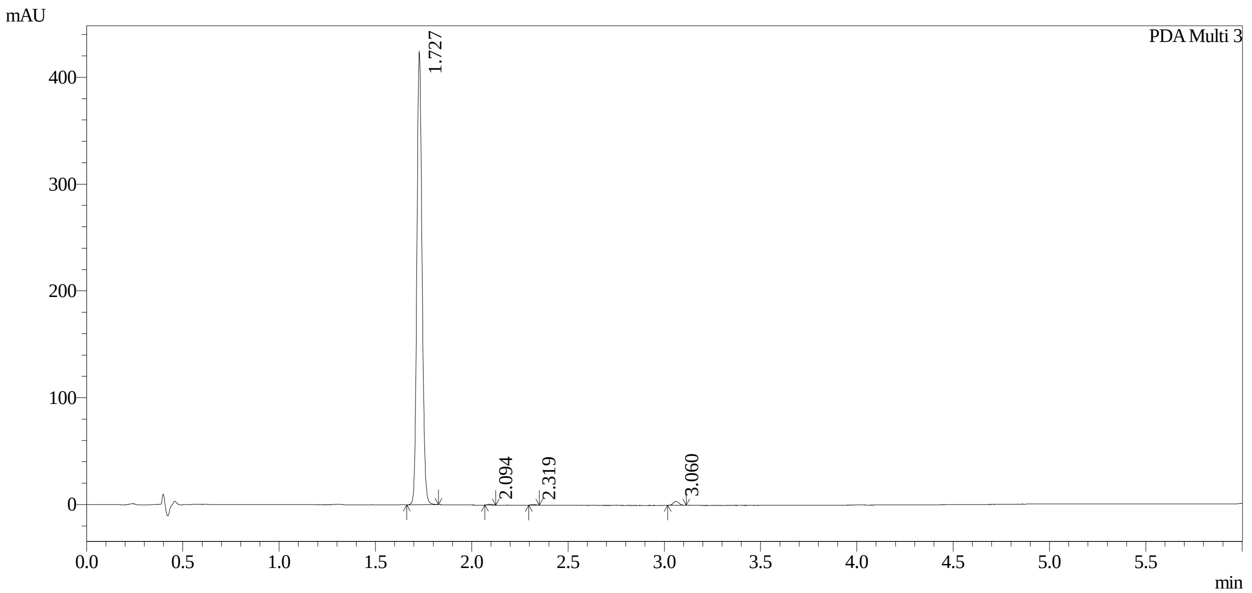

1 PDA Multi 3 / 254nm,4nm

Integration result

PeakTable

PDA Ch3 254nm

Peak#	Ret. Time	USP Width	Resolution	Height	Area	Area %
1	1.727	0.050	0.000	424148	796095	98.765
2	2.094	0.047	7.529	764	1289	0.160
3	2.319	0.047	4.763	587	1041	0.129
4	3.060	0.056	14.286	3684	7625	0.946
Total				429182	806050	100.000

Operater : ________________

Date : ________________

Confidential. For research only, Not for regulatory filing.

[redacted]

[redacted]

EW13860-41-P1B DMSO Bruker_F_400MHz

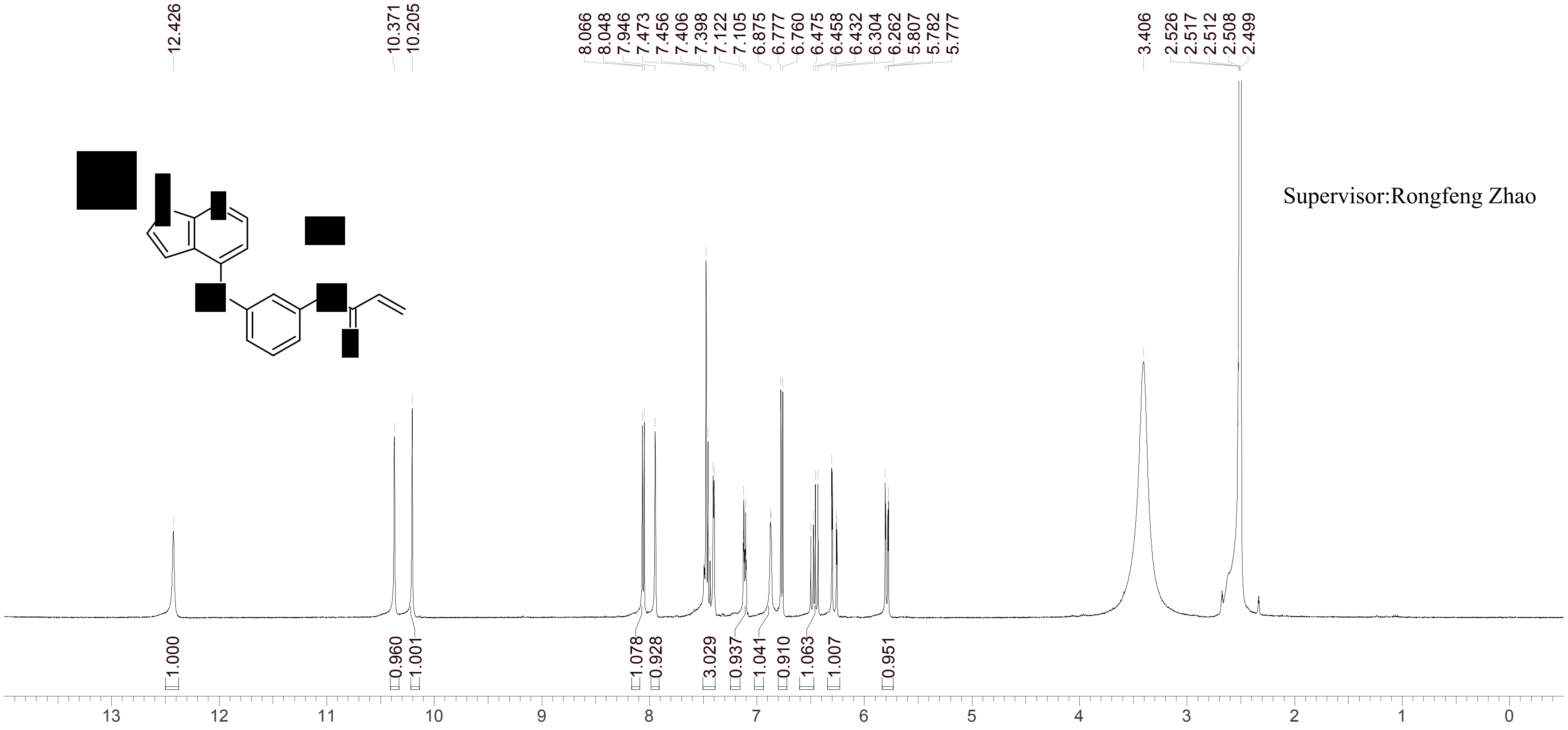

Supervisor:Rongfeng Zhao

Operator:

Date:

If you [redacted]

[redacted]

Confidential. For research only, Not for regulatory filing.

[redacted]

Compound ID: GK-D2

EW13860-41-P1C DMSO Bruker_F_400MHz

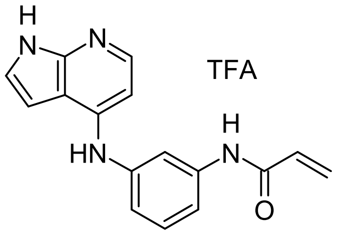

Supervisor:Rongfeng Zhao

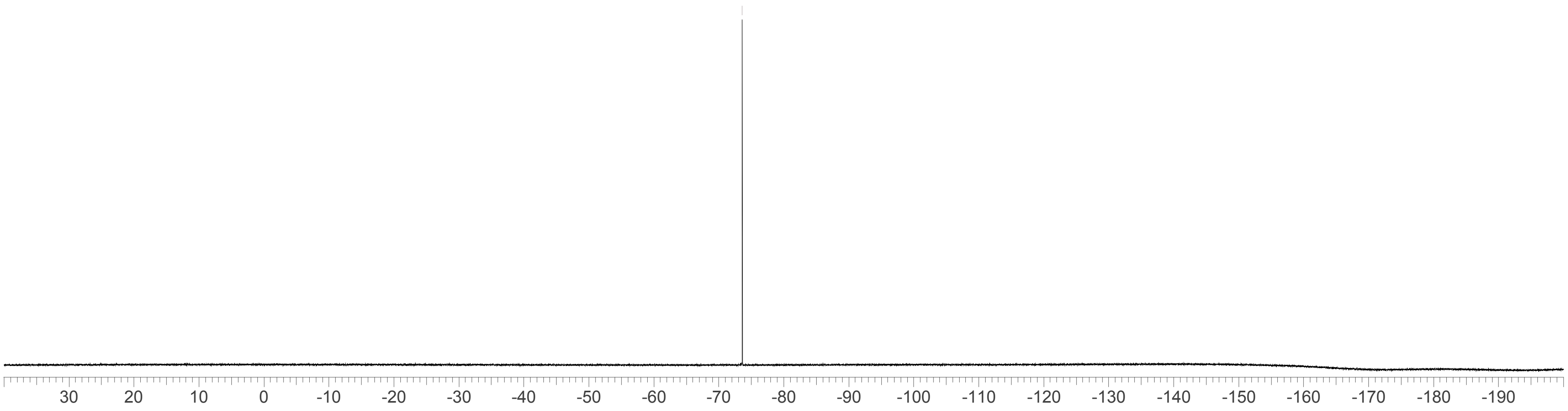

[redacted]

Confidential, for research only not for regulatory filing

LCMS REPORT

Compound ID : GK-D2
Sample ID : EW13860-41-P1F
Injection Vol : 5ul
Location : vial50
Acq Method : d:\method\5-95CD_R_220&254_POS.lcm
Org DataFile : D:\DATA\1809\180907\EW13860-41-P1F.lcd
Injection Date : 2018-09-07 13:21:24
Instrument :LCMS-M 17-102

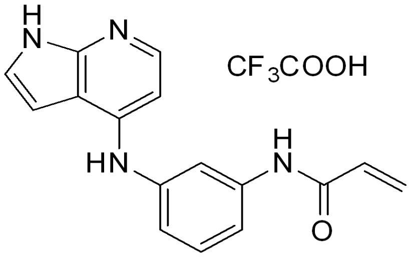

Exact Mass: 392.11

Chromatogram

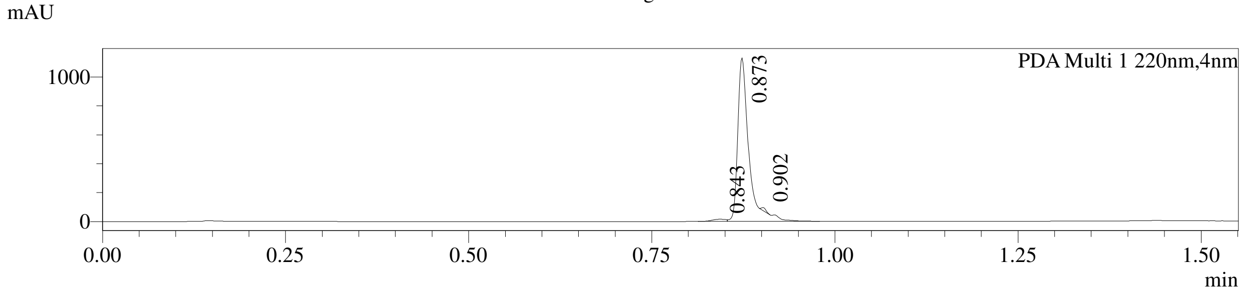

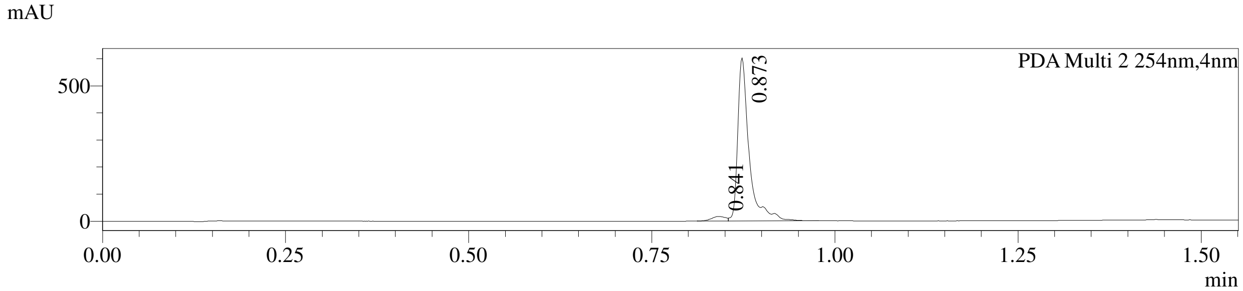

1 PDA Multi 1 / 220nm,4nm
2 PDA Multi 2 / 254nm,4nm

MS Chromatogram

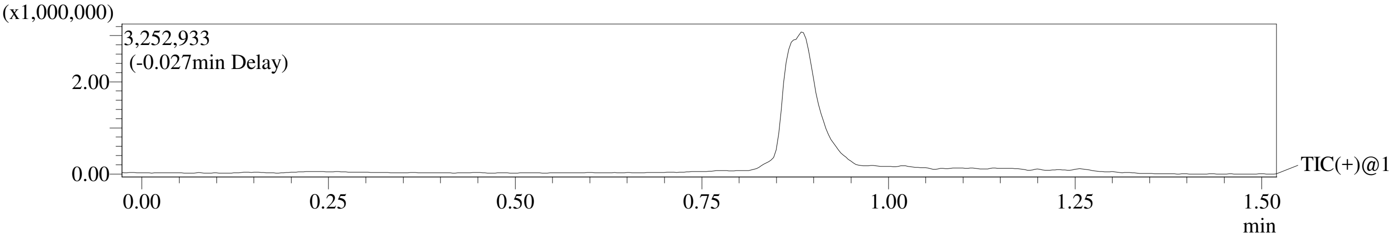

Integration Result

Peak Table

PDA Ch1 220nm

Peak#	Ret. Time	Height	Height%	USP Width	Area	Area%
1	0.843	14523	1.246	0.056	19251	1.569
2	0.873	1130362	96.943	0.027	1199911	97.780
3	0.902	21125	1.812	0.012	7986	0.651

Peak Table

PDA Ch2 254nm

Peak#	Ret. Time	Height	Height%	USP Width	Area	Area%
1	0.841	16298	2.635	0.050	22184	3.282
2	0.873	602249	97.365	0.027	653724	96.718

Confidential, for research only not for regulatory filing

Operator:_______________

Date:___________________

Confidential, for research only not for regulatory filing

Mass Spectrum

RetTime: 0.843 Datafile: D:\DATA\1809\180907\EW13860-41-P1F.lcd

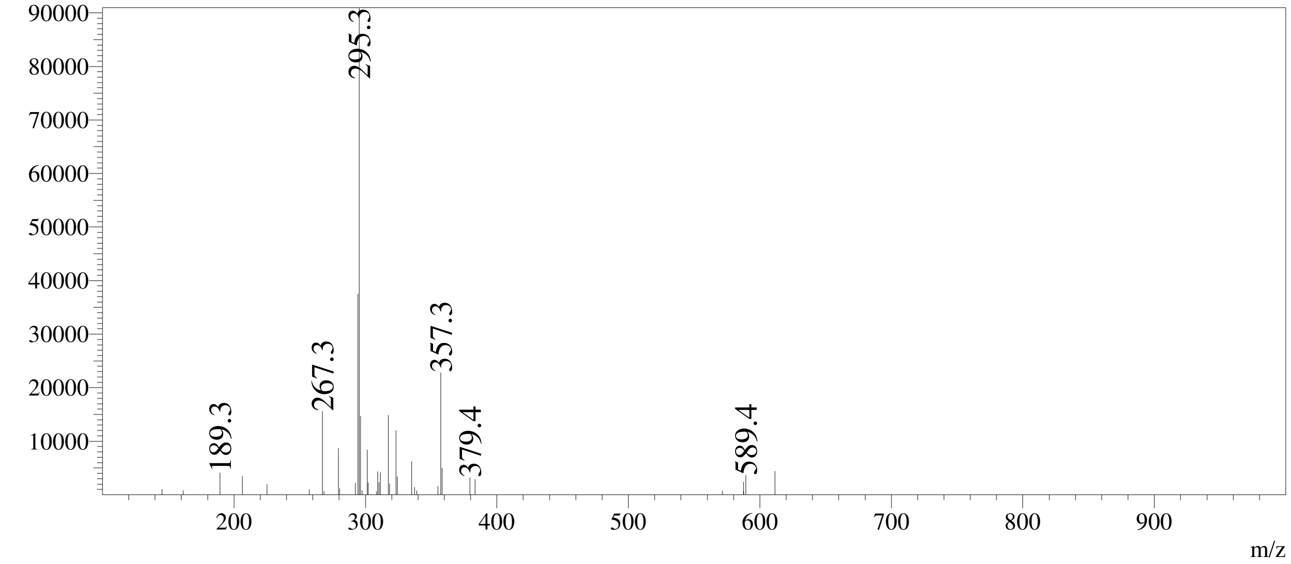

RetTime: 0.873 Datafile: D:\DATA\1809\180907\EW13860-41-P1F.lcd

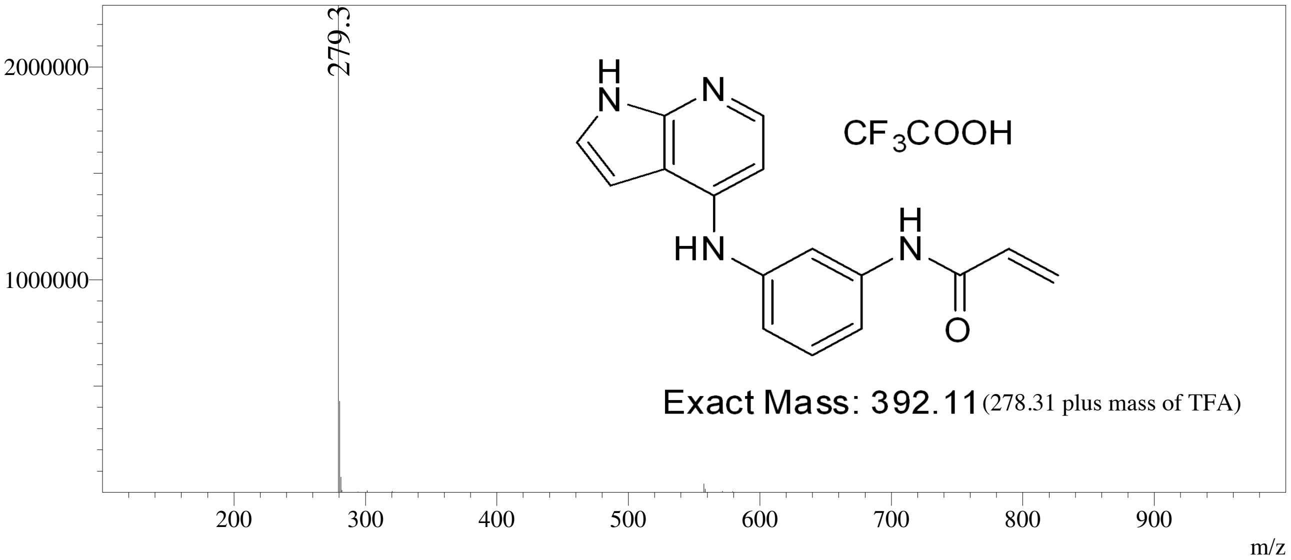

RetTime: 0.903 Datafile: D:\DATA\1809\180907\EW13860-41-P1F.lcd

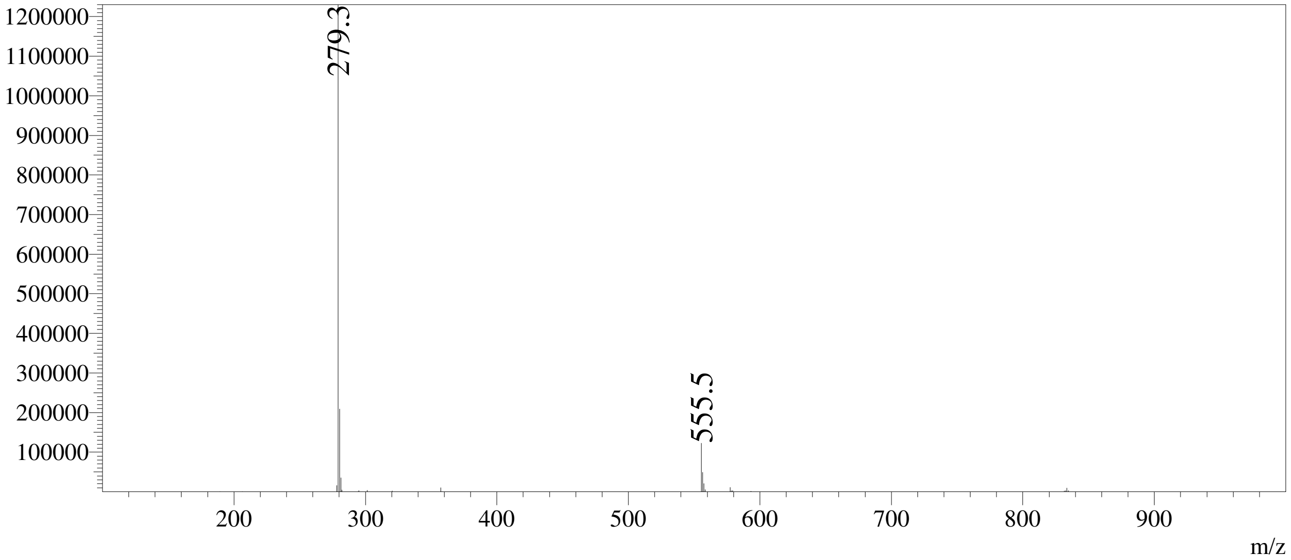

HPLC REPORT

Compound ID : GK-D2
Sample ID : EW13860-41-P1A
Vial# : 87
Injection Volumn : 3
Filename : D:\DATA\2018\1809\180907\EW13860-41-P1A.lcd
Method Name : D:\Method\10-80CD_4min.lcm
Instrument : HPLC-J
Run time : 9/7/2018 11:30:02

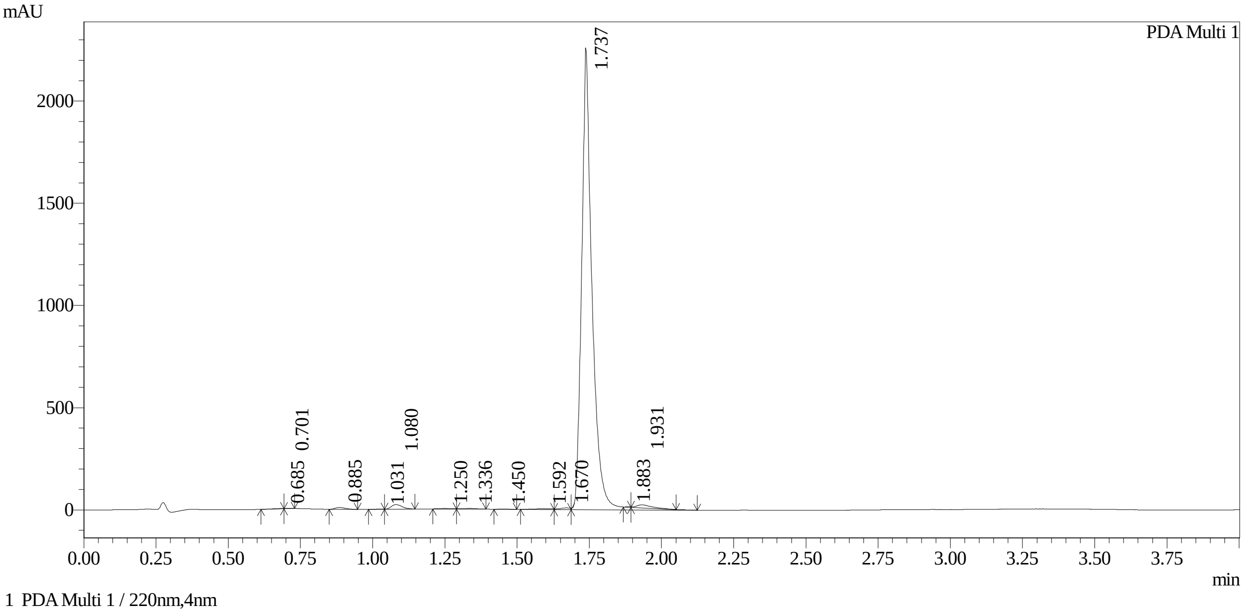

1 PDA Multi 1 / 220nm,4nm

Integration result

PeakTable

PDA Ch1 220nm

Peak#	Ret. Time	USP Width	Resolution	Height	Area	Area %
1	0.685	0.101	0.000	1942	5471	0.093
2	0.701	0.068	0.182	1585	2214	0.038
3	0.885	0.068	2.693	8834	21770	0.369
4	1.031	0.071	2.110	1304	2306	0.039
5	1.080	0.070	0.698	22199	57773	0.980
6	1.250	0.089	2.141	2538	7674	0.130
7	1.336	0.106	0.874	2914	9759	0.165
8	1.450	0.065	1.343	1340	3134	0.053
9	1.592	0.185	1.139	4319	17911	0.304
10	1.670	0.118	0.510	8511	21644	0.367
11	1.737	0.064	0.737	2259744	5689810	96.476
12	1.883	0.000	0.000	1479	1928	0.033
13	1.931	0.098	0.000	15117	56257	0.954
Total				2331828	5897650	100.000

Operater : ________________

PDF created with pdfFactory Pro trial version www.pdffactory.com

Date : ________________

PDF created with pdfFactory Pro trial version www.pdffactory.com

Confidential, for research only not for regulatory filing

HPLC REPORT

Compound ID : GK-D2
Sample ID : EW13860-41-P1A
Filename : D:\DATA\2018\1809\180907\EW13860-41-P1A.lcd
Method Name : D:\Method\10-80CD_4min.lcm
Instrument &Column : HPLC-J
Run time : 9/7/2018 11:30:02

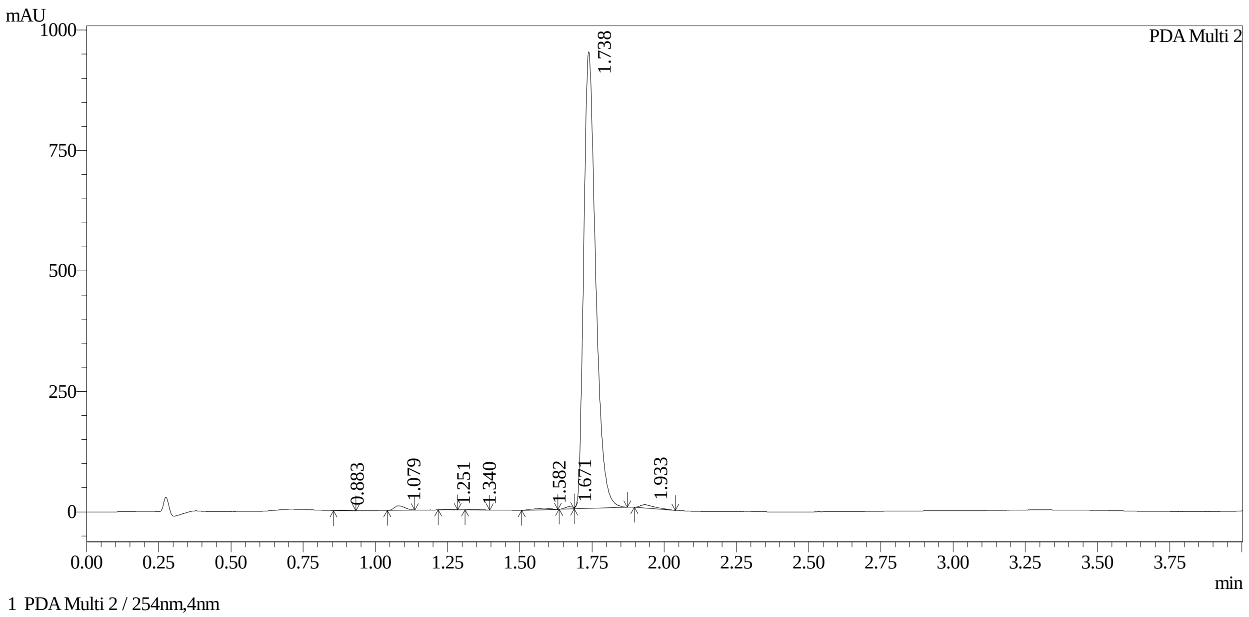

1 PDA Multi 2 / 254nm,4nm

Integration result

PeakTable

PDA Ch2 254nm

Peak#	Ret. Time	USP Width	Resolution	Height	Area	Area %
1	0.883	0.060	0.000	1141	2610	0.093
2	1.079	0.069	3.042	9313	23538	0.836
3	1.251	0.063	2.602	981	2164	0.077
4	1.340	0.065	1.404	1085	2650	0.094
5	1.582	0.120	2.616	2930	11977	0.426
6	1.671	0.067	0.945	4675	8842	0.314
7	1.738	0.076	0.938	947441	2738252	97.293
8	1.933	0.102	2.183	6780	24394	0.867
Total				974348	2814426	100.000

Operater : ________________

Date : ________________

PDF created with pdfFactory Pro trial version www.pdffactory.com

Confidential, for research only not for regulatory filing

HPLC REPORT

Compound ID : GK-D2
Sample ID : EW13860-41-P1A
Filename : D:\DATA\2018\1809\180907\EW13860-41-P1A.lcd
Method Name : D:\Method\10-80CD_4min.lcm
Instrument &Column : HPLC-J
Run time : 9/7/2018 11:30:02

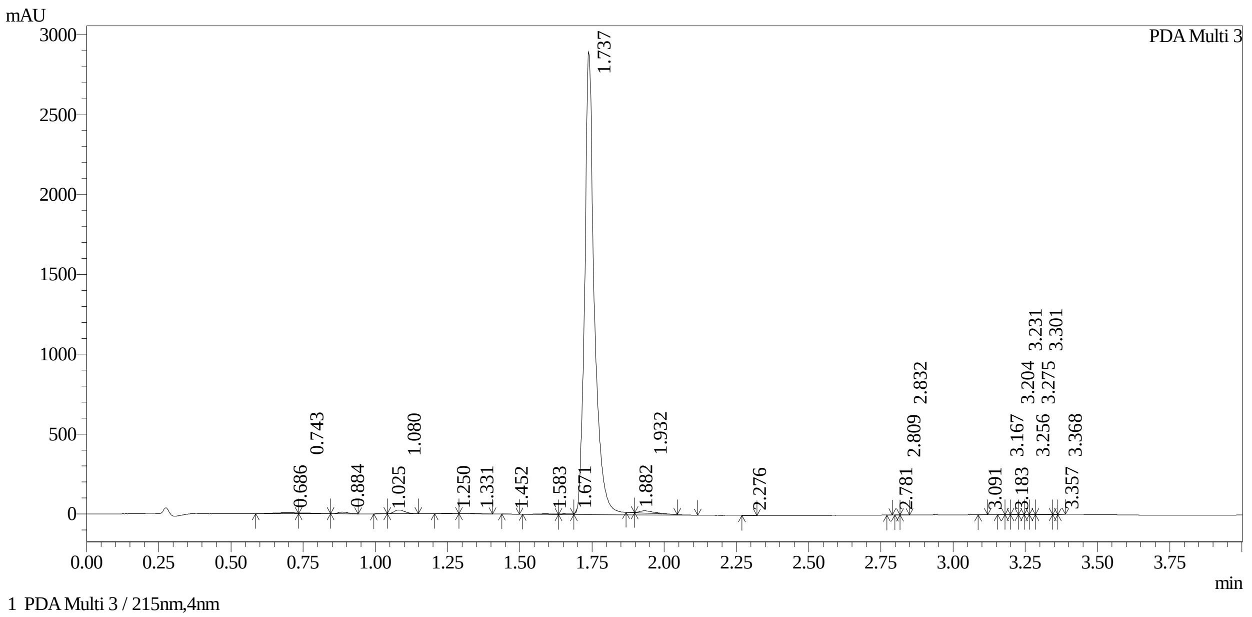

1 PDA Multi 3 / 215nm,4nm

Integration result

PeakTable

PDA Ch3 215nm

Peak#	Ret. Time	USP Width	Resolution	Height	Area	Area %
1	0.686	0.295	0.000	6000	32660	0.454
2	0.743	0.276	0.200	4926	16460	0.229
3	0.884	0.069	0.819	9964	24837	0.346
4	1.025	0.055	2.269	1307	2463	0.034
5	1.080	0.070	0.873	23677	61379	0.854
6	1.250	0.066	2.507	2025	5052	0.070
7	1.331	0.085	1.076	2417	7674	0.107
8	1.452	0.039	1.949	1044	1693	0.024
9	1.583	0.218	1.025	4405	20077	0.279
10	1.671	0.144	0.484	8566	20501	0.285
11	1.737	0.052	0.677	2898622	6910151	96.140
12	1.882	0.000	0.000	1477	2372	0.033
13	1.932	0.102	0.000	15345	56288	0.783
14	2.276	0.092	3.540	837	1954	0.027
15	2.781	0.068	6.333	1078	1105	0.015
16	2.809	0.044	0.504	1324	1225	0.017
17	2.832	0.055	0.455	666	1066	0.015
18	3.091	0.082	3.772	578	1081	0.015
19	3.167	0.101	0.831	1115	1600	0.022
20	3.183	0.040	0.227	1233	1369	0.019

PDF created with pdfFactory Pro trial version www.pdffactory.com

Peak#	Ret. Time	USP Width	Resolution	Height	Area	Area %
21	3.204	0.084	0.345	1214	2030	0.028
22	3.231	0.000	0.000	1461	1649	0.023
23	3.256	0.169	0.000	1652	1662	0.023
24	3.275	0.150	0.117	1828	2283	0.032
25	3.301	0.883	0.051	1976	6370	0.089
26	3.357	0.342	0.092	1182	1252	0.017
27	3.368	0.073	0.051	1045	1365	0.019
Total				2996964	7187616	100.000

Operater : ________________

Date : ________________

PDF created with pdfFactory Pro trial version www.pdffactory.com

Confidential. For research only, Not for regulatory filing.

[redacted]

Compound ID: GK-D3

EW13998-13-P1B CDCl3 Bruker_D_400MHz

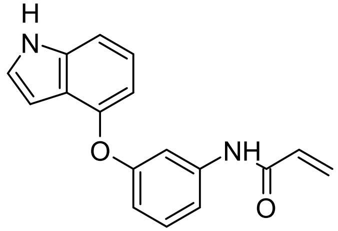

Supervisor:Rongfeng Zhao

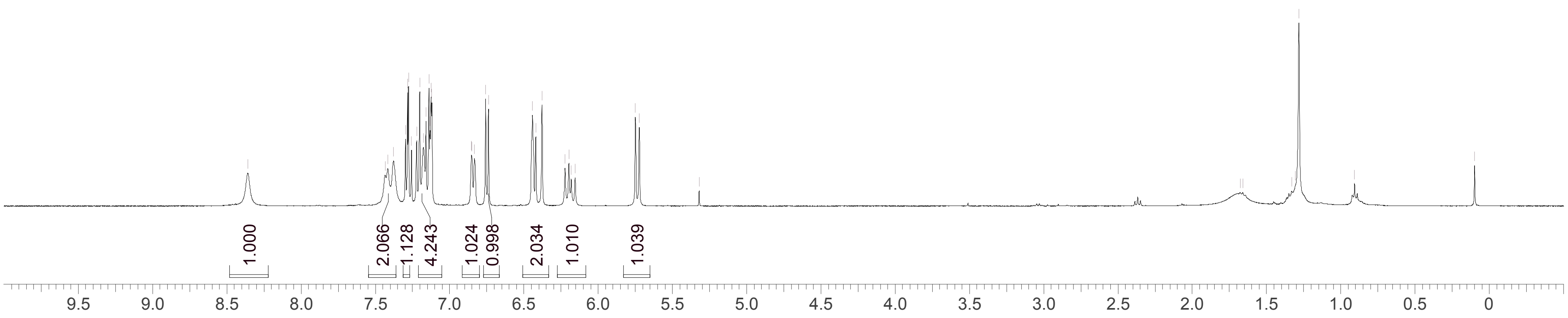

Operator:

Date:

[redacted]

EW13998-13-P1B1 CDCl3 HSQC

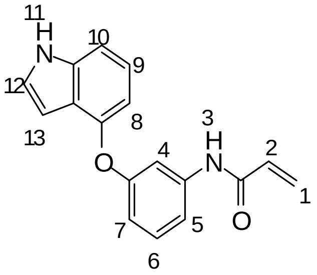

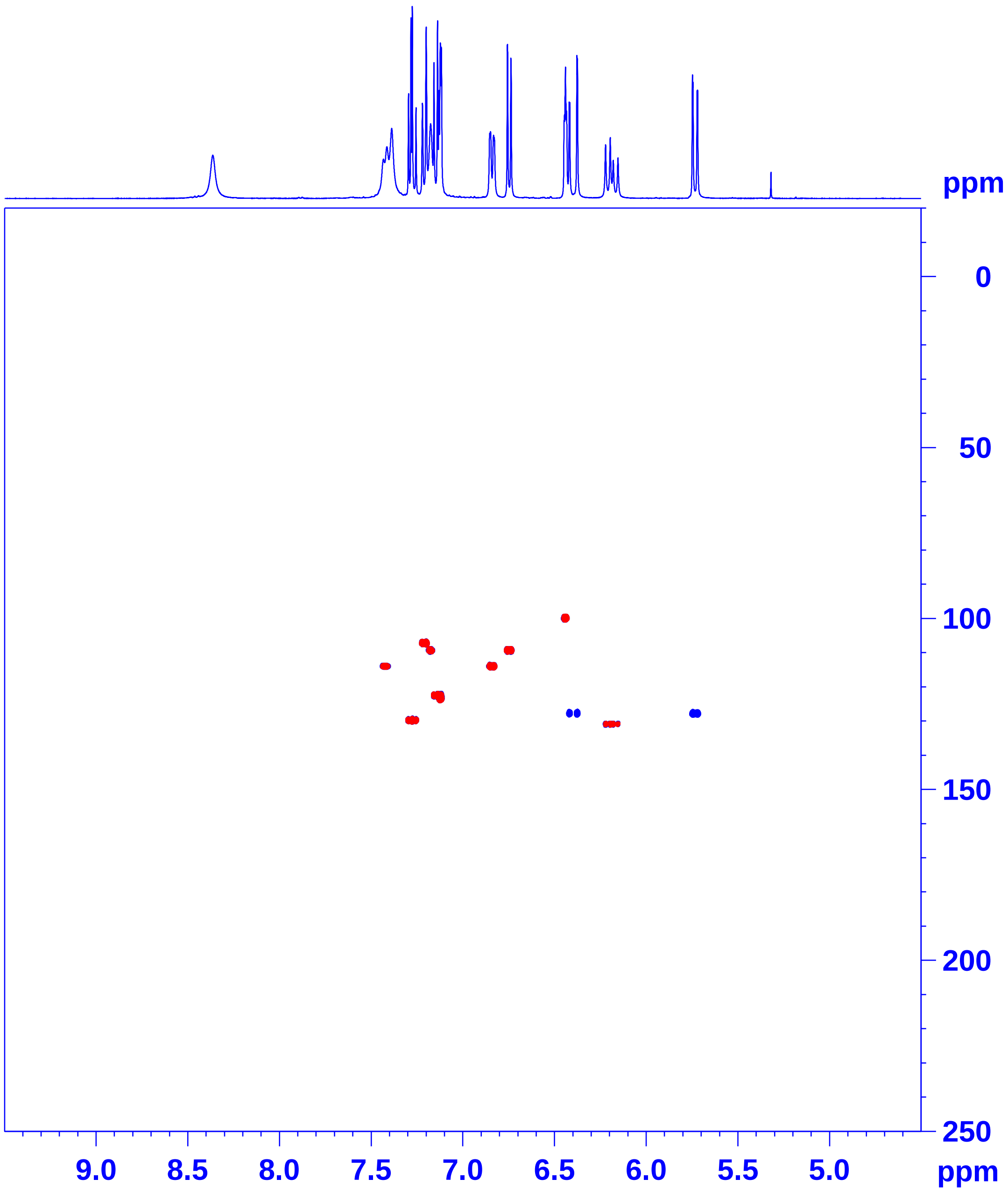

[redacted]

```
NAME        EW13998-13-P1B1
EXPNO                     2
PROCNO                    1
Date_              20180914
Time                   2.02
INSTRUM               spect
PROBHD      5 mm PABBO BB/
PULPROG          hsqcedetgp
TD                     1024
SOLVENT               CDCl3
NS                        2
DS                       16
SWH                2000.000 H
FIDRES             1.953125 H
AQ                0.2560500 s
RG                   211.47
DW                  250.000 u
DE                     6.50 u
TE                    298.2 K
CNST2           145.0000000
D0               0.00000300 s
D1               1.50000000 s
D4               0.00172414 s
D11              0.03000000 s
D13              0.00000400 s
D16              0.00020000 s
D21              0.00345000 s
IN0              0.00001840 s
ZGOPTNS

======== CHANNEL f1 =====
SFO1            400.1328009 M
NUC1                     1H
P1                     9.45 u
P2                    18.90 u
P28                 1000.00 u
ND0                       2
TD                      256
SFO1               100.6243 M
FIDRES           106.148094 H
SW                  270.053 p
FnMODE         Echo-Antiecho
SI                     1024
SF              400.1300000 M
WDW                   QSINE
SSB                       2
LB                     0.00 H
GB                        0
PC                     1.40
SI                     1024
MC2             echo-antiecho
SF              100.6127685 M
WDW                   QSINE
SSB                       2
LB                     0.00 H
GB                        0
```

Confidential, for research only not for regulatory filing

EW13998-13-P1B1 CDCl3 HSQC

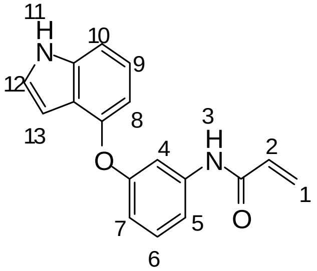

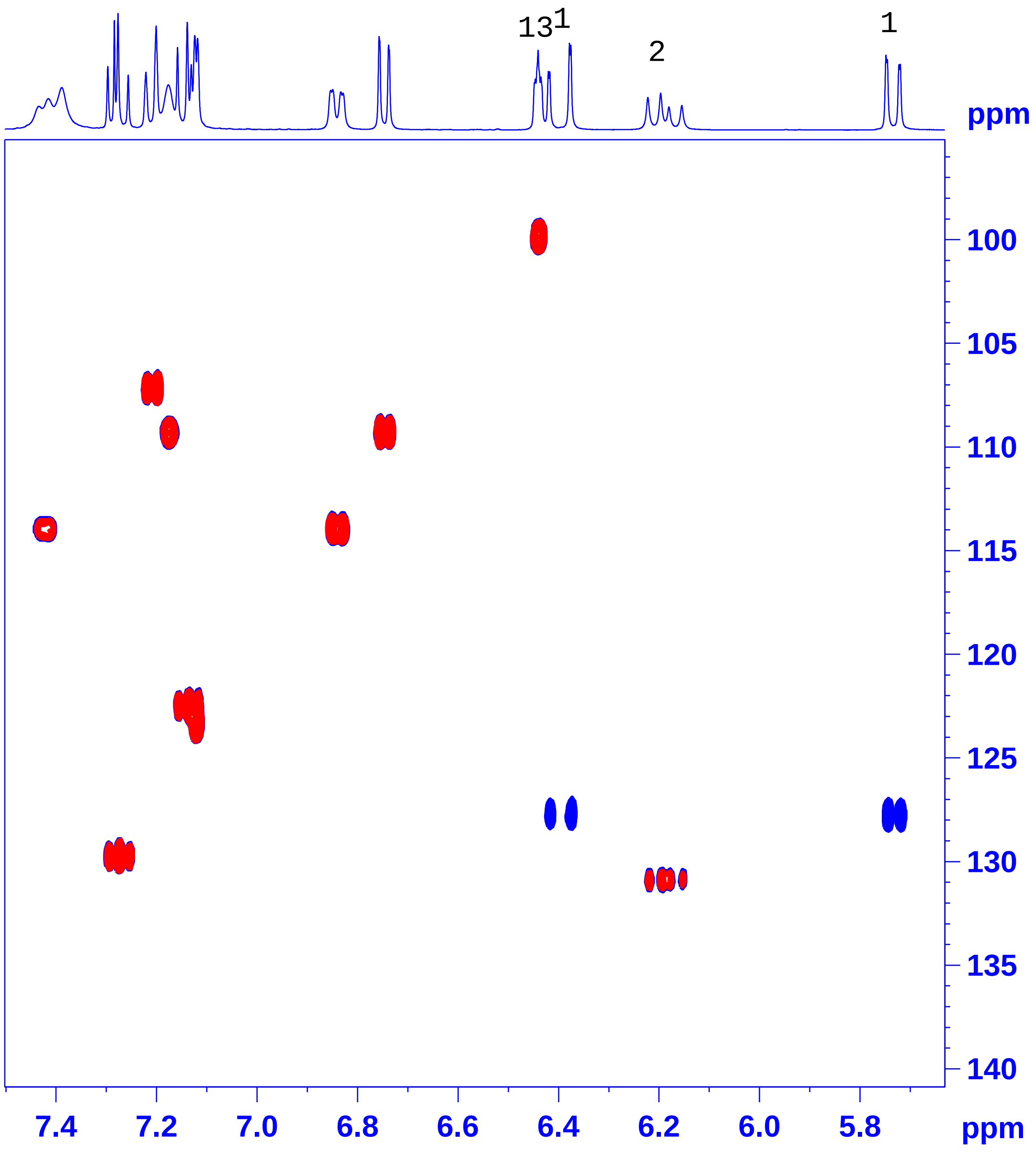

[redacted]

NAME	EW13998-13-P1B1
EXPNO	2
PROCNO	1
Date_	20180914
Time	2.02
INSTRUM	spect
PROBHD	5 mm PABBO BB/
PULPROG	hsqcedetgp
TD	1024
SOLVENT	CDCl3
NS	2
DS	16
SWH	2000.000 H
FIDRES	1.953125 H
AQ	0.2560500 s
RG	211.47
DW	250.000 u
DE	6.50 u
TE	298.2 K
CNST2	145.0000000
D0	0.00000300 s
D1	1.50000000 s
D4	0.00172414 s
D11	0.03000000 s
D13	0.00000400 s
D16	0.00020000 s
D21	0.00345000 s
IN0	0.00001840 s
ZGOPTNS	
======== CHANNEL f1 =====	
SFO1	400.1328009 M
NUC1	1H
P1	9.45 u
P2	18.90 u
P28	1000.00 u
ND0	2
TD	256
SFO1	100.6243 M
FIDRES	106.148094 H
SW	270.053 p
FnMODE	Echo-Antiecho
SI	1024
SF	400.1300000 M
WDW	QSINE
SSB	2
LB	0.00 H
GB	0
PC	1.40
SI	1024
MC2	echo-antiecho
SF	100.6127685 M
WDW	QSINE
SSB	2
LB	0.00 H
GB	0

Confidential, for research only not for regulatory filing

EW13998-13-P1B1 CDCl3 HSQC

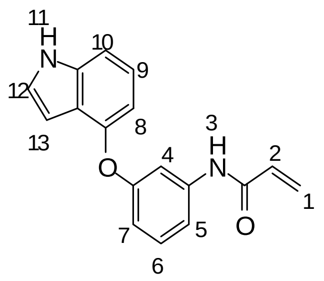

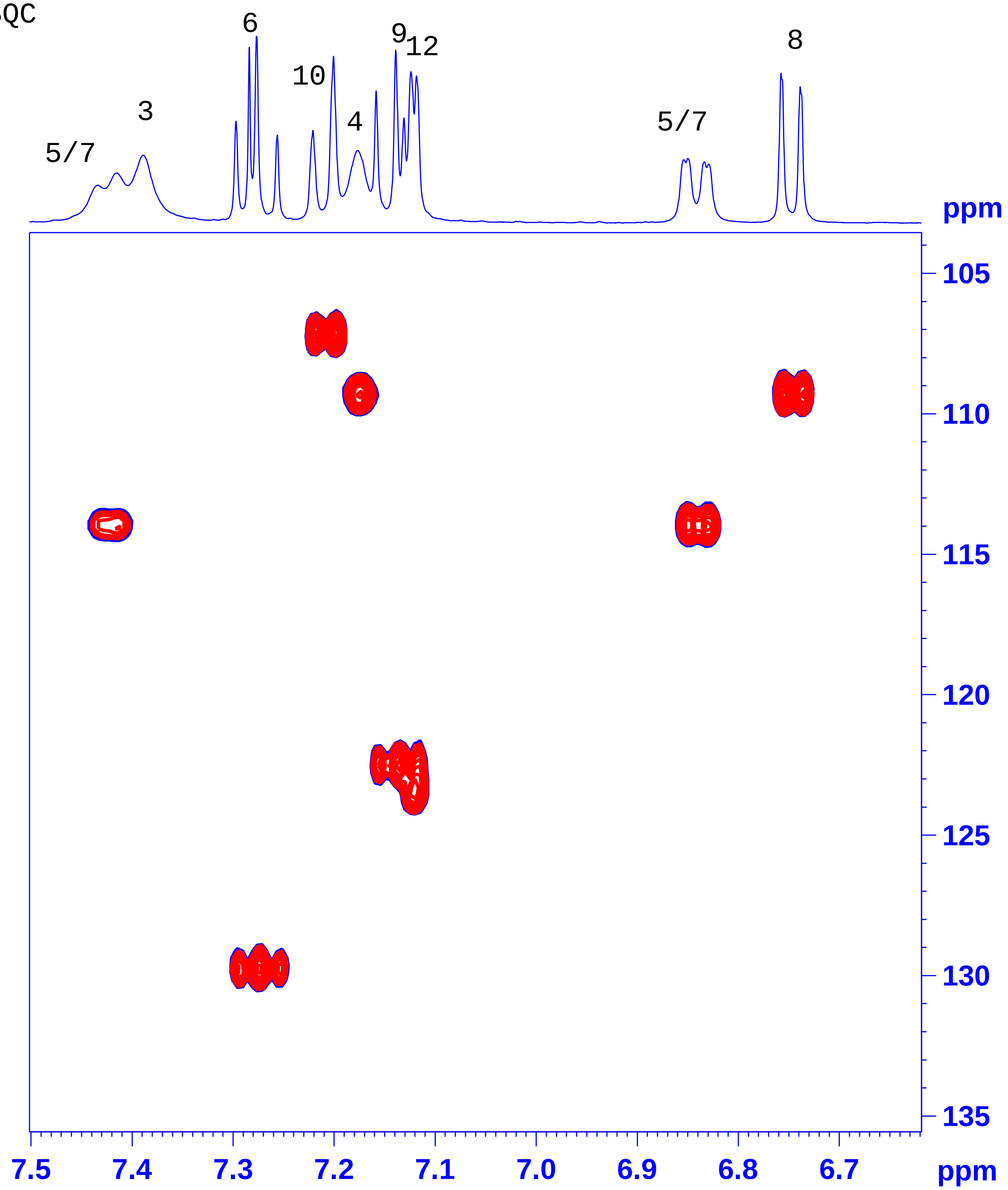

[redacted]

NAME	EW13998-13-P1B1
EXPNO	2
PROCNO	1
Date_	20180914
Time	2.02
INSTRUM	spect
PROBHD	5 mm PABBO BB/
PULPROG	hsqcedetgp
TD	1024
SOLVENT	CDCl3
NS	2
DS	16
SWH	2000.000 H
FIDRES	1.953125 H
AQ	0.2560500 s
RG	211.47
DW	250.000 u
DE	6.50 u
TE	298.2 K
CNST2	145.0000000
D0	0.00000300 s
D1	1.50000000 s
D4	0.00172414 s
D11	0.03000000 s
D13	0.00000400 s
D16	0.00020000 s
D21	0.00345000 s
IN0	0.00001840 s
ZGOPTNS	

======== CHANNEL f1 =====

SFO1	400.1328009 M
NUC1	1H
P1	9.45 u
P2	18.90 u
P28	1000.00 u
ND0	2
TD	256
SFO1	100.6243 M
FIDRES	106.148094 H
SW	270.053 p
FnMODE	Echo-Antiecho
SI	1024
SF	400.1300000 M
WDW	QSINE
SSB	2
LB	0.00 H
GB	0
PC	1.40
SI	1024
MC2	echo-antiecho
SF	100.6127685 M
WDW	QSINE
SSB	2
LB	0.00 H
GB	0

Confidential, for research only not for regulatory filing

EW13998-13-P1B1 CDCl3 NOE

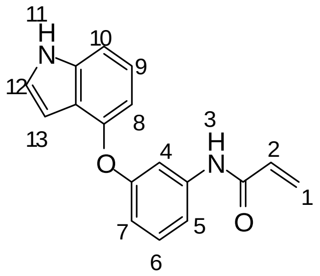

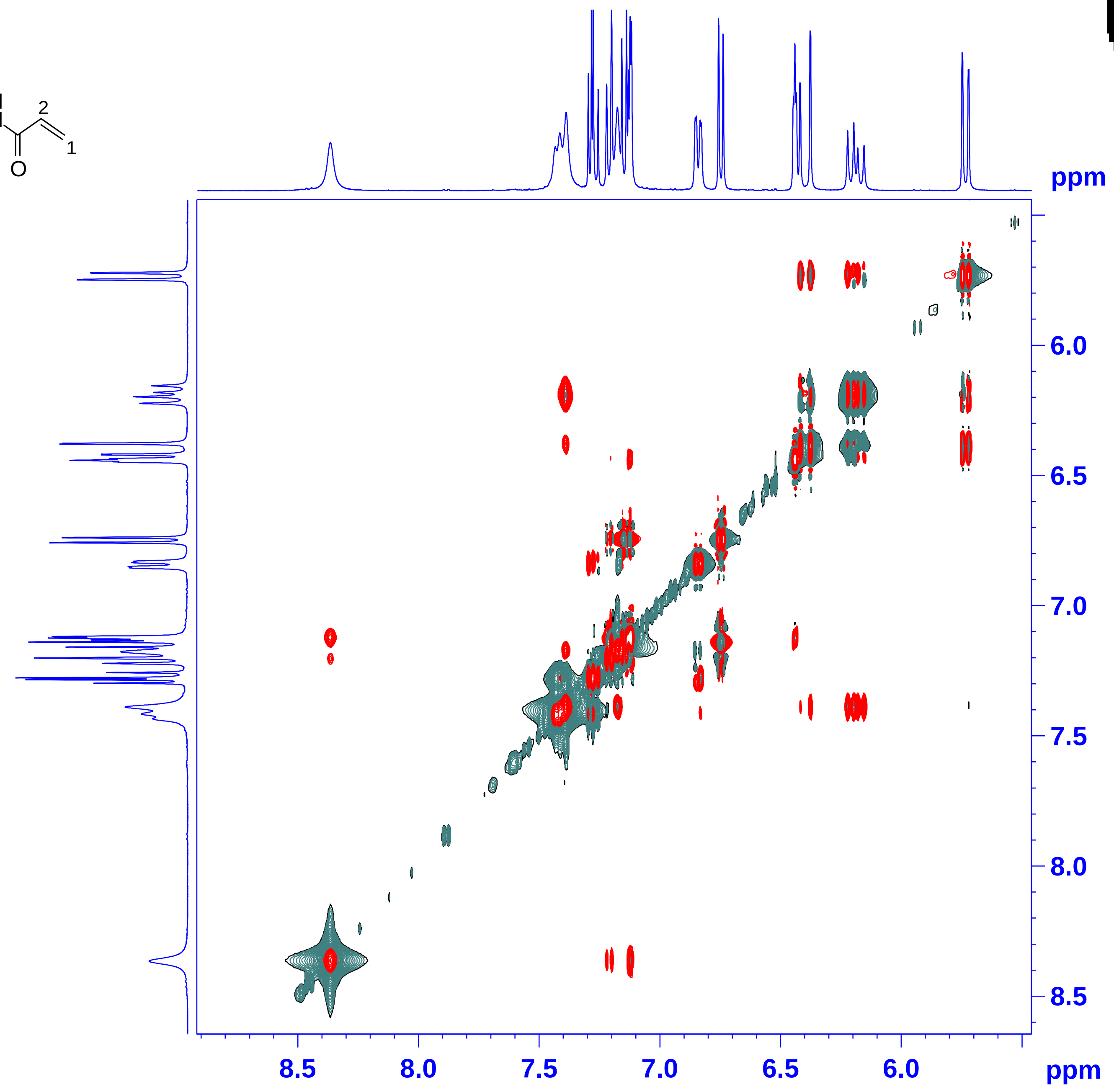

[redacted]

NAME	EW13998-13-P1B1
EXPNO	3
PROCNO	1
Date_	20180914
Time	2.18
INSTRUM	spect
PROBHD	5 mm PABBO BB/
PULPROG	noesygpph
TD	2048
SOLVENT	CDCl3
NS	8
DS	8
SWH	2000.000 Hz
FIDRES	0.976563 Hz
AQ	0.5120500 se
RG	117.79
DW	250.000 us
DE	6.50 us
TE	298.2 K
D0	0.00023787 se
D1	1.79999995 se
D8	0.80000001 se
D16	0.00020000 se
IN0	0.00049980 se
======== CHANNEL f1 =======	
SFO1	400.1328009 MH
NUC1	1H
P1	9.45 us
P2	18.90 us
ND0	1
TD	200
SFO1	400.1328 MH
FIDRES	10.004002 Hz
SW	5.000 pp
FnMODE	States-TPPI
SI	1024
SF	400.1300000 MH
WDW	QSINE
SSB	2
LB	0.00 Hz
GB	0
PC	1.40
SI	1024
MC2	States-TPPI
SF	400.1300000 MH
WDW	QSINE
SSB	2
LB	0.00 Hz
GB	0

Confidential, for research only not for regulatory filing

EW13998-13-P1B1 CDCl3 NOE

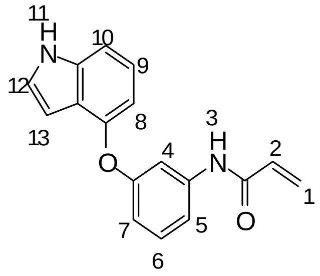

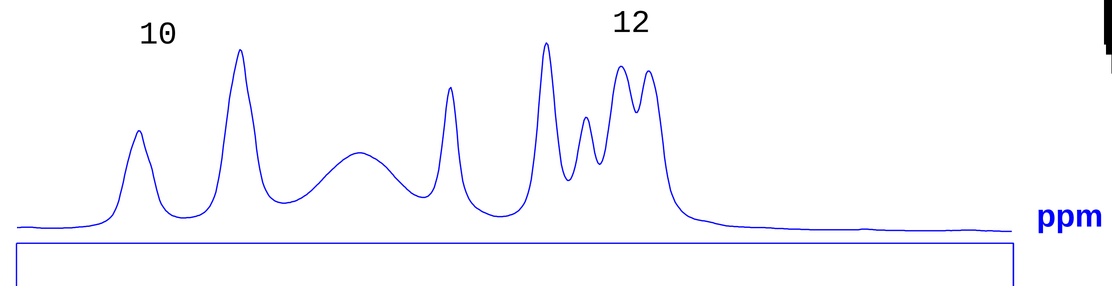

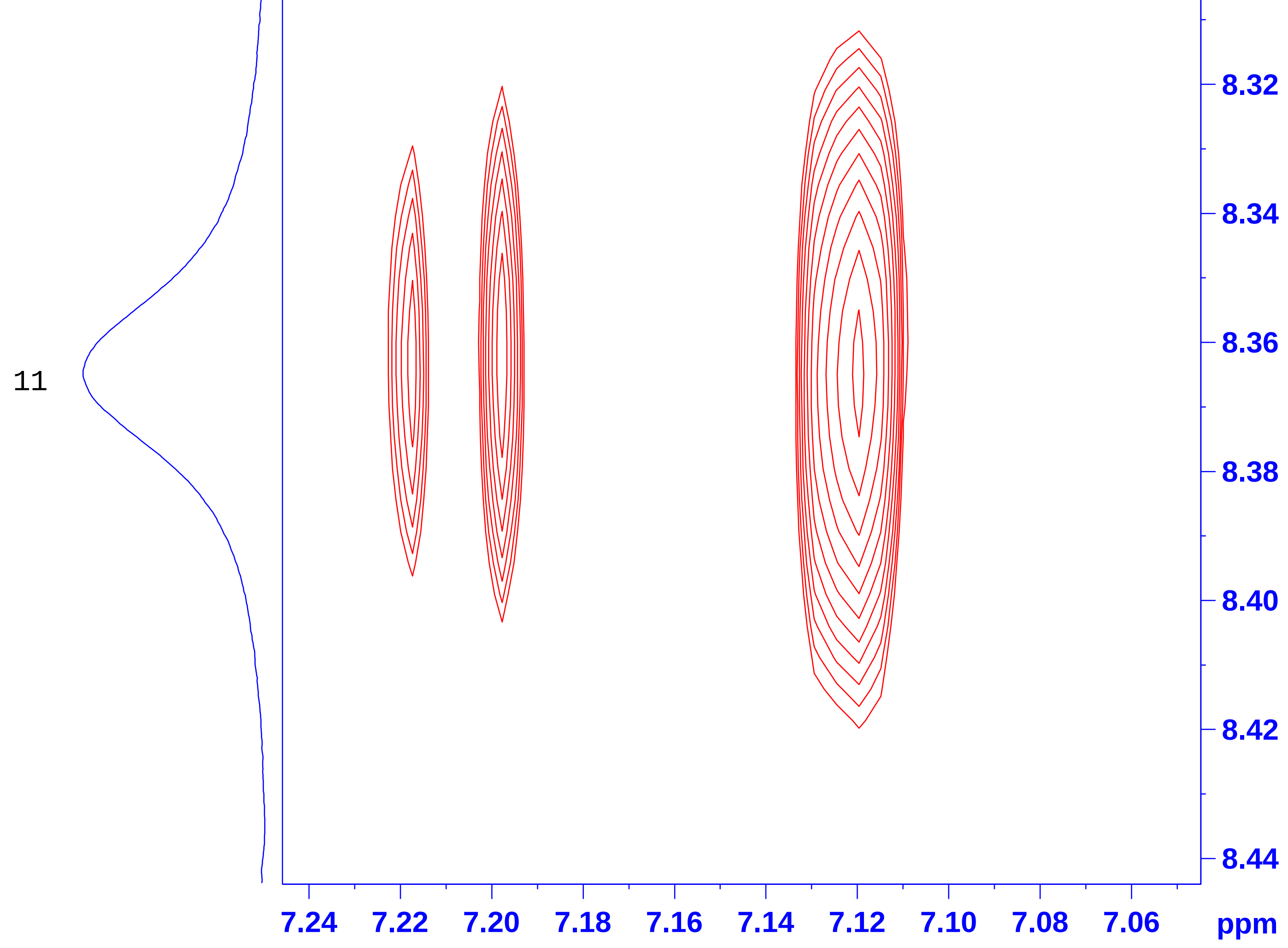

[redacted]

```
NAME        EW13998-13-P1B1
EXPNO                     3
PROCNO                    1
Date_              20180914
Time                   2.18
INSTRUM               spect
PROBHD       5 mm PABBO BB/
PULPROG            noesygpph
TD                     2048
SOLVENT               CDCl3
NS                        8
DS                        8
SWH                2000.000 Hz
FIDRES             0.976563 Hz
AQ                0.5120500 se
RG                   117.79
DW                  250.000 us
DE                     6.50 us
TE                    298.2 K
D0               0.00023787 se
D1               1.79999995 se
D8               0.80000001 se
D16              0.00020000 se
IN0              0.00049980 se

======== CHANNEL f1 =======
SFO1            400.1328009 MH
NUC1                     1H
P1                     9.45 us
P2                    18.90 us
ND0                       1
TD                      200
SFO1               400.1328 MH
FIDRES            10.004002 Hz
SW                    5.000 pp
FnMODE          States-TPPI
SI                     1024
SF              400.1300000 MH
WDW                   QSINE
SSB                       2
LB                     0.00 Hz
GB                        0
PC                     1.40
SI                     1024
MC2             States-TPPI
SF              400.1300000 MH
WDW                   QSINE
SSB                       2
LB                     0.00 Hz
GB                        0
```

Confidential, for research only not for regulatory filing

EW13998-13-P1B1 CDCl3 NOE

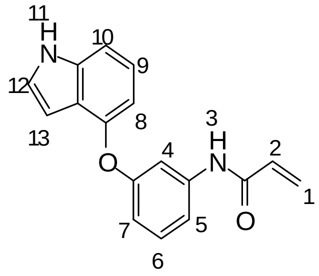

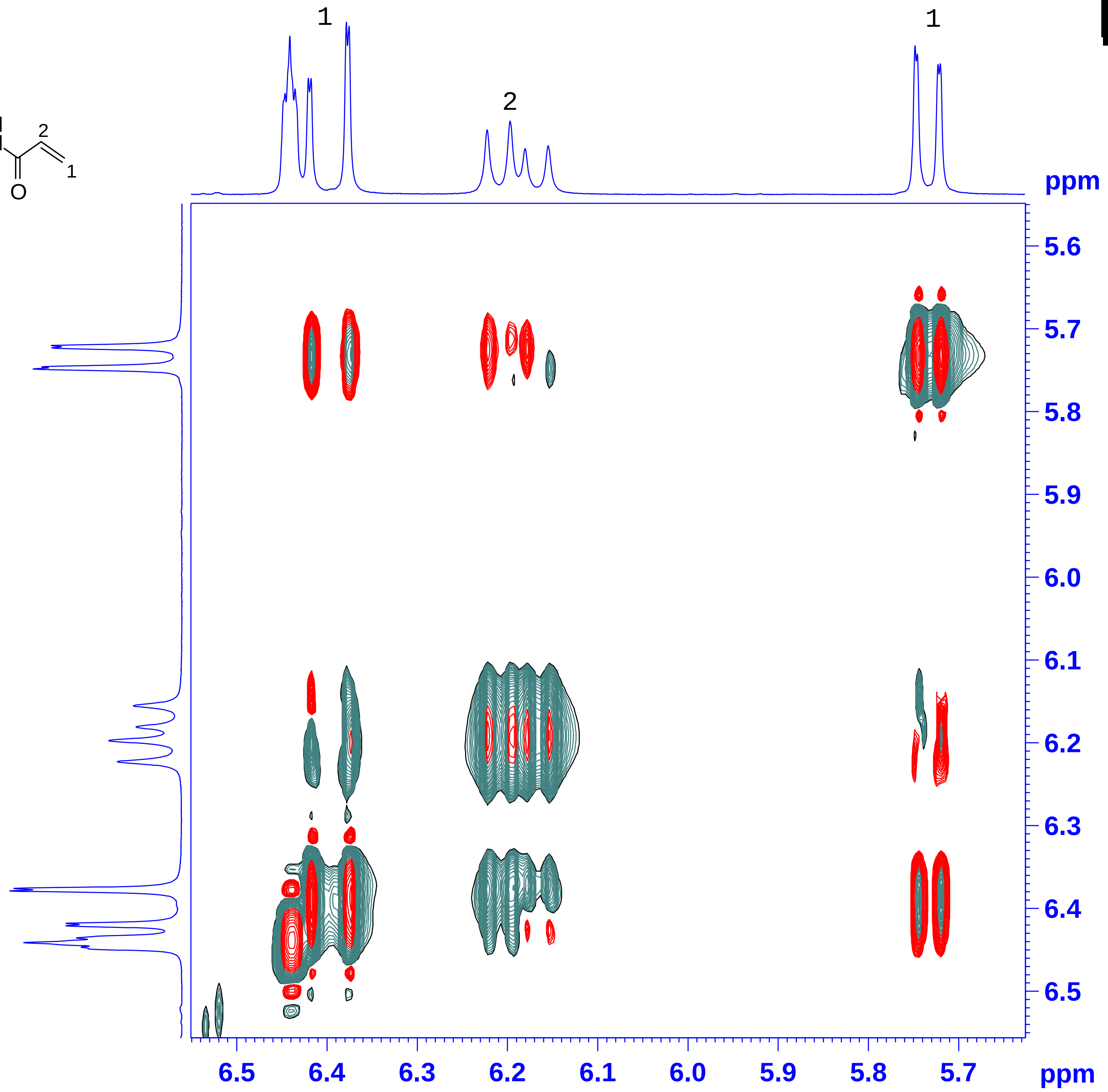

[redacted]

```
NAME      EW13998-13-P1B1
EXPNO                   3
PROCNO                  1
Date_            20180914
Time                 2.18
INSTRUM             spect
PROBHD   5 mm PABBO BB/
PULPROG          noesygpph
TD                   2048
SOLVENT             CDCl3
NS                      8
DS                      8
SWH              2000.000 Hz
FIDRES           0.976563 Hz
AQ              0.5120500 sec
RG                 117.79
DW                250.000 usec
DE                   6.50 usec
TE                  298.2 K
D0             0.00023787 sec
D1             1.79999995 sec
D8             0.80000001 sec
D16            0.00020000 sec
IN0            0.00049980 sec

======== CHANNEL f1 ========
SFO1          400.1328009 MHz
NUC1                   1H
P1                   9.45 usec
P2                  18.90 usec
ND0                     1
TD                    200
SFO1             400.1328 MHz
FIDRES          10.004002 Hz
SW                  5.000 ppm
FnMODE        States-TPPI
SI                   1024
SF            400.1300000 MHz
WDW                 QSINE
SSB                     2
LB                   0.00 Hz
GB                      0
PC                   1.40
SI                   1024
MC2           States-TPPI
SF            400.1300000 MHz
WDW                 QSINE
SSB                     2
LB                   0.00 Hz
GB                      0
```

Confidential, for research only not for regulatory filing

EW13998-13-P1B1 CDCl3 NOE

[redacted]

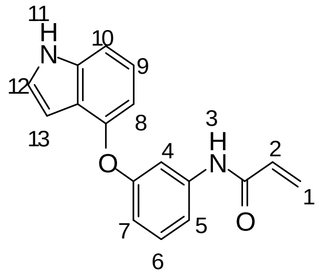

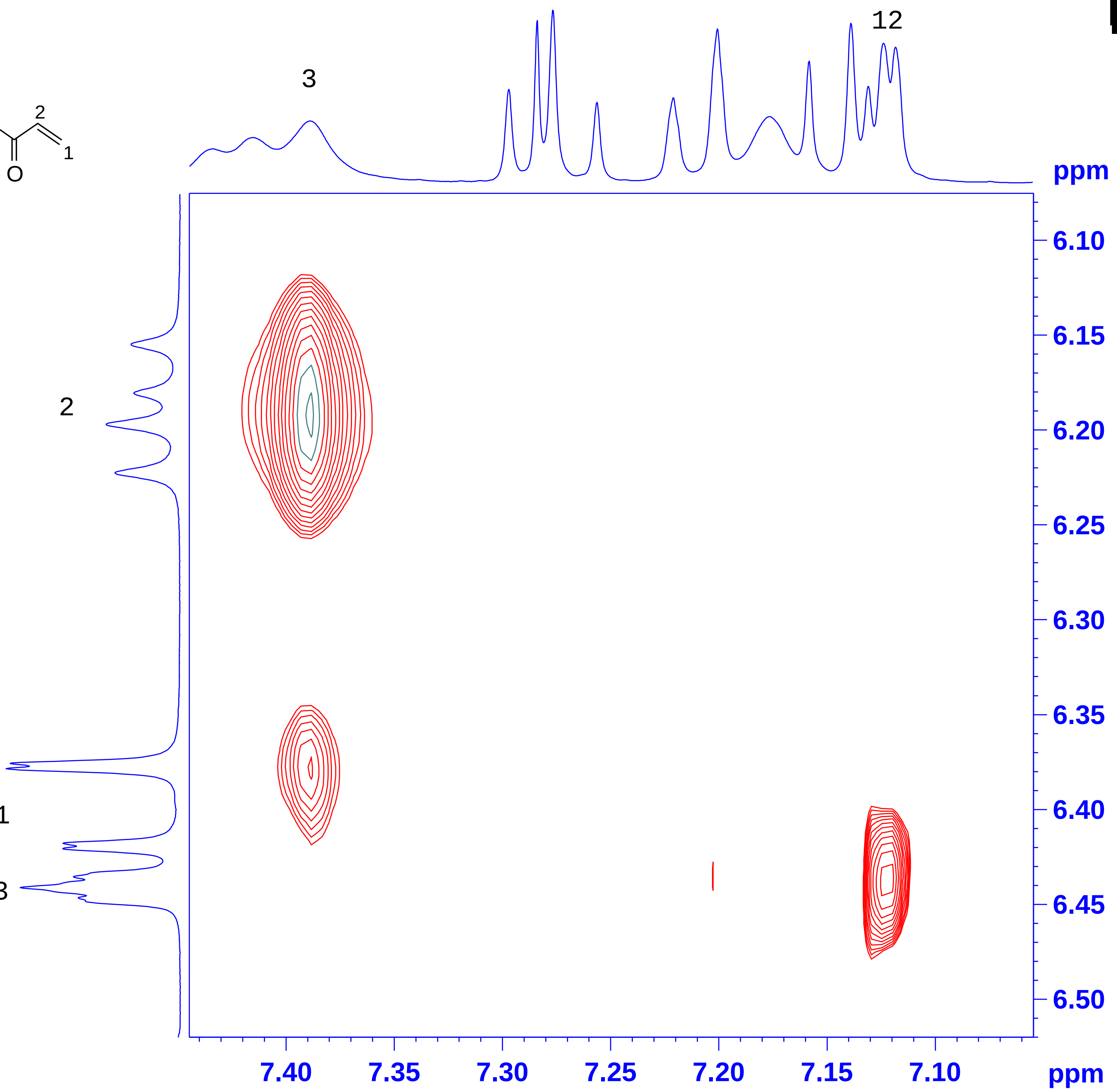

NAME	EW13998-13-P1B1	
EXPNO	3	
PROCNO	1	
Date_	20180914	
Time	2.18	
INSTRUM	spect	
PROBHD	5 mm PABBO BB/	
PULPROG	noesygpph	
TD	2048	
SOLVENT	CDCl3	
NS	8	
DS	8	
SWH	2000.000	Hz
FIDRES	0.976563	Hz
AQ	0.5120500	sec
RG	117.79	
DW	250.000	usec
DE	6.50	usec
TE	298.2	K
D0	0.00023787	sec
D1	1.79999995	sec
D8	0.80000001	sec
D16	0.00020000	sec
IN0	0.00049980	sec
======== CHANNEL f1 ========		
SFO1	400.1328009	MHz
NUC1	1H	
P1	9.45	usec
P2	18.90	usec
ND0	1	
TD	200	
SFO1	400.1328	MHz
FIDRES	10.004002	Hz
SW	5.000	ppm
FnMODE	States-TPPI	
SI	1024	
SF	400.1300000	MHz
WDW	QSINE	
SSB	2	
LB	0.00	Hz
GB	0	
PC	1.40	
SI	1024	
MC2	States-TPPI	
SF	400.1300000	MHz
WDW	QSINE	
SSB	2	
LB	0.00	Hz
GB	0	

Confidential, for research only not for regulatory filing

EW13998-13-P1B1 CDCl3 NOE

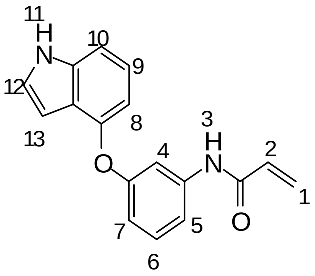

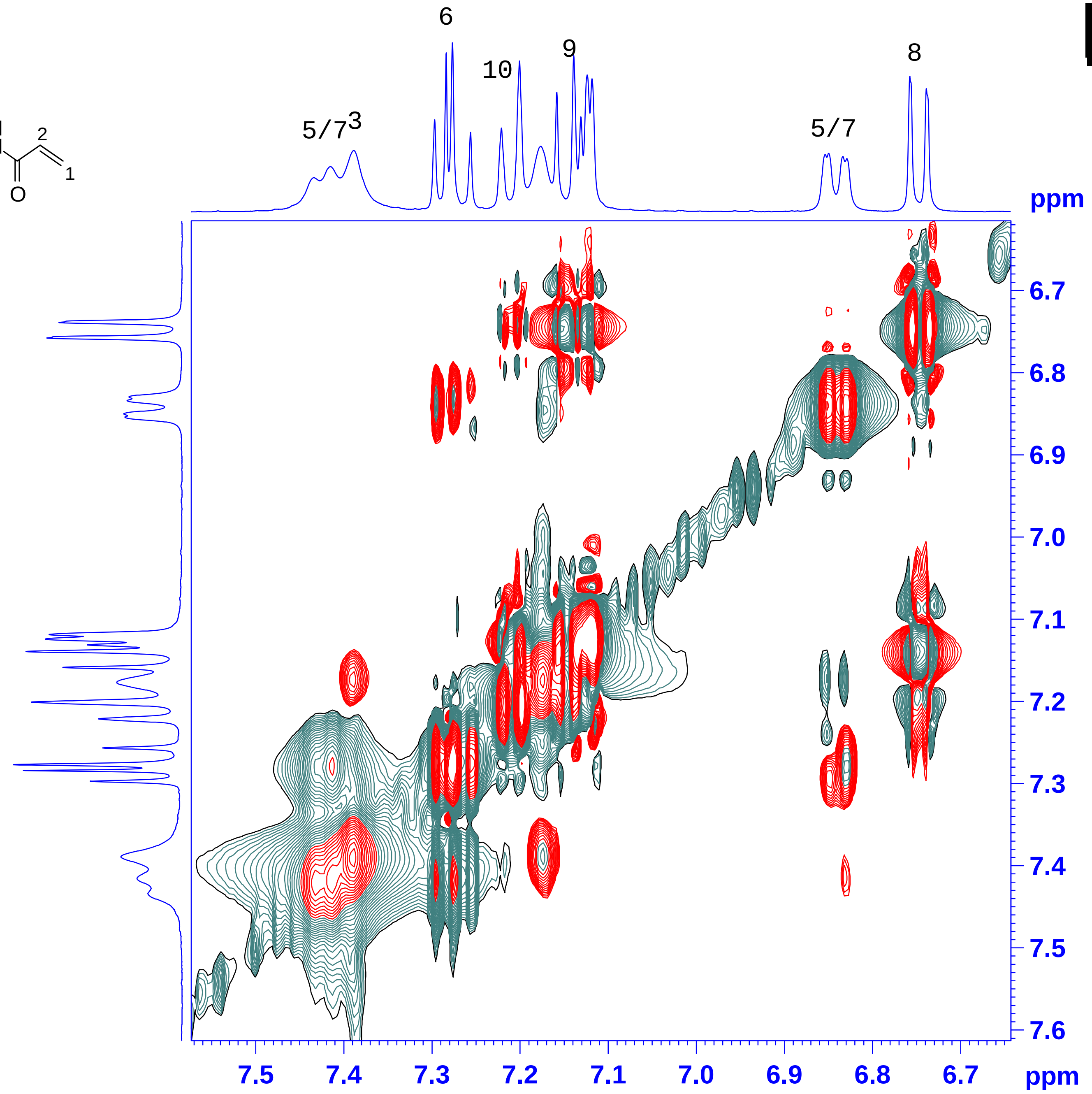

[redacted]

NAME	EW13998-13-P1B1
EXPNO	3
PROCNO	1
Date_	20180914
Time	2.18
INSTRUM	spect
PROBHD	5 mm PABBO BB/
PULPROG	noesygpph
TD	2048
SOLVENT	CDCl3
NS	8
DS	8
SWH	2000.000 Hz
FIDRES	0.976563 Hz
AQ	0.5120500 se
RG	117.79
DW	250.000 us
DE	6.50 us
TE	298.2 K
D0	0.00023787 se
D1	1.79999995 se
D8	0.80000001 se
D16	0.00020000 se
IN0	0.00049980 se
======== CHANNEL f1 ======	
SFO1	400.1328009 MH
NUC1	1H
P1	9.45 us
P2	18.90 us
ND0	1
TD	200
SFO1	400.1328 MH
FIDRES	10.004002 Hz
SW	5.000 pp
FnMODE	States-TPPI
SI	1024
SF	400.1300000 MH
WDW	QSINE
SSB	2
LB	0.00 Hz
GB	0
PC	1.40
SI	1024
MC2	States-TPPI
SF	400.1300000 MH
WDW	QSINE
SSB	2
LB	0.00 Hz
GB	0

Confidential, for research only not for regulatory filing

Confidential, for research only not for regulatory filing

LCMS REPORT

Compound ID : GK-D3
Sample ID : EW13998-13-P1B2
Injection Vol : 10ul
Location : vial9
Acq Method : d:\method\5-95CD_R_220&254_POS.lcm
Org DataFile : D:\DATA\1809\180911\EW13998-13-P1B2.lcd
Injection Date : 9/11/2018 5:12:14 PM
Instrument :LCMS-W 17-102

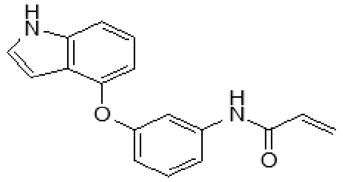

Chromatogram

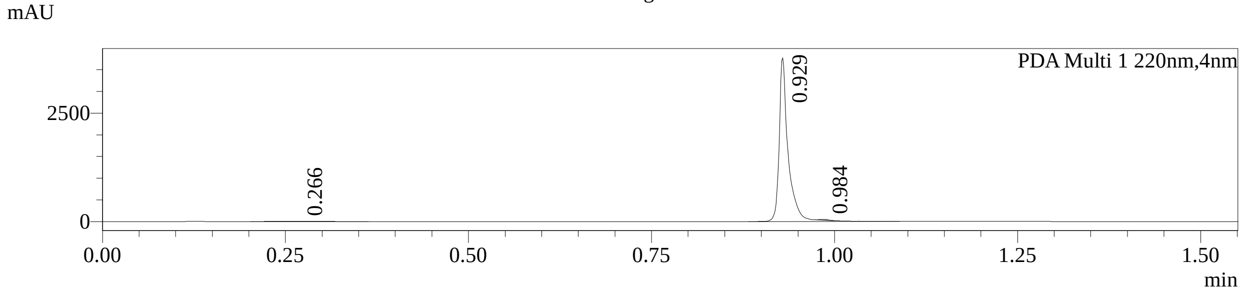

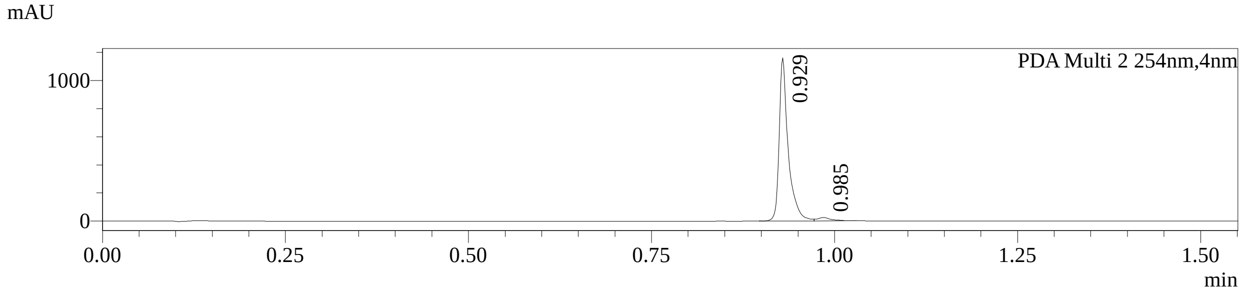

1 PDA Multi 1 / 220nm,4nm
2 PDA Multi 2 / 254nm,4nm

MS Chromatogram

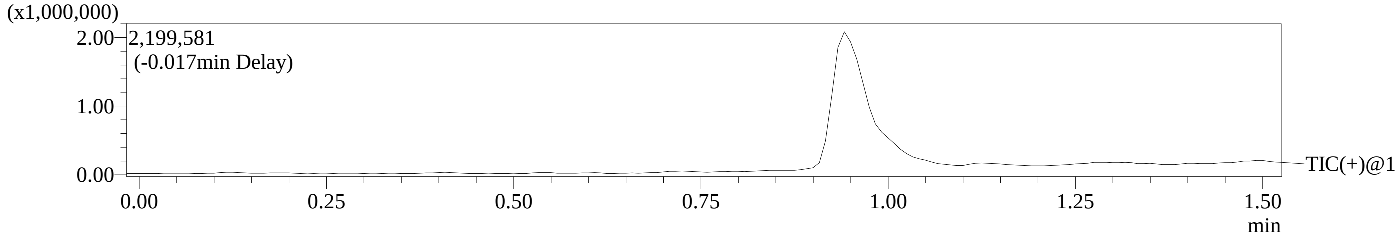

Integration Result

Peak Table

PDA Ch1 220nm

Peak#	Ret. Time	Height	Height%	USP Width	Area	Area%
1	0.266	6494	0.171	0.136	35274	1.069
2	0.929	3773628	99.362	0.021	3250436	98.539
3	0.984	17741	0.467	0.023	12934	0.392

Peak Table

PDA Ch2 254nm

Peak#	Ret. Time	Height	Height%	USP Width	Area	Area%
1	0.929	1160745	98.018	0.023	996298	97.072
2	0.985	23471	1.982	0.038	30055	2.928

Confidential, for research only not for regulatory filing

Operator:_______________

Date:___________________

Mass Spectrum

RetTime: 0.266 Datafile: D:\DATA\1809\180911\EW13998-13-P1B2.lcd

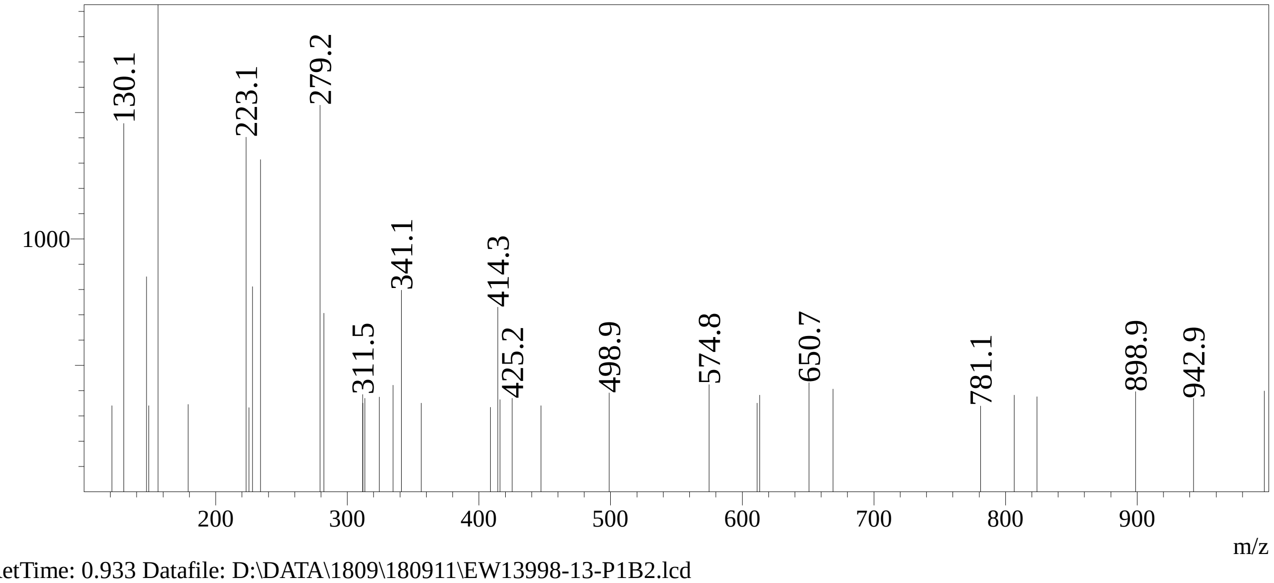

RetTime: 0.933 Datafile: D:\DATA\1809\180911\EW13998-13-P1B2.lcd

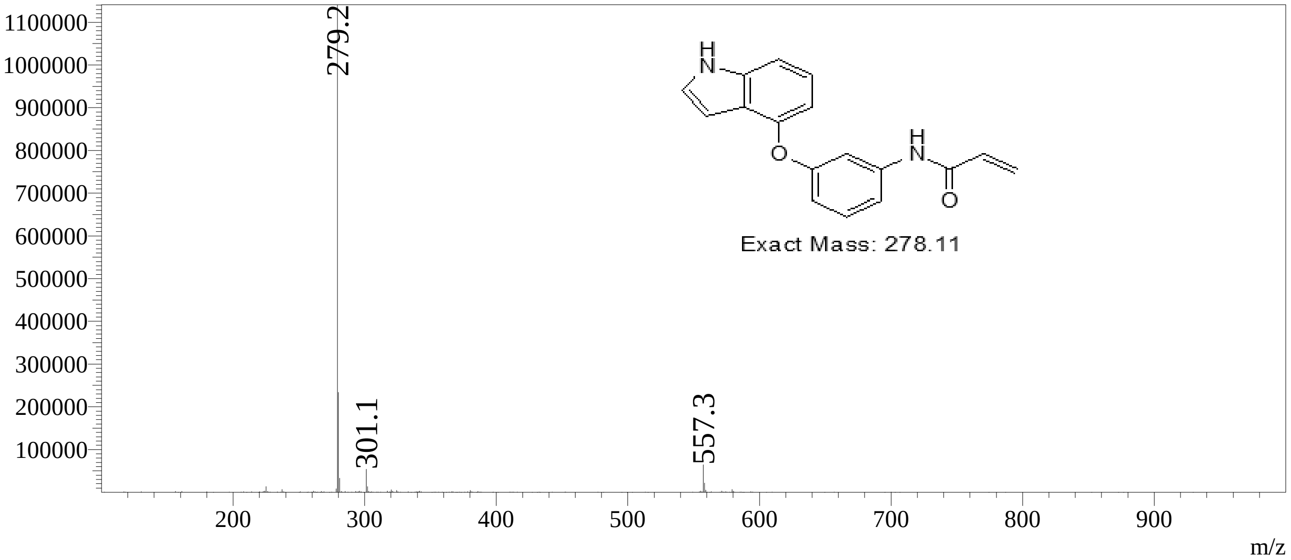

RetTime: 0.983 Datafile: D:\DATA\1809\180911\EW13998-13-P1B2.lcd

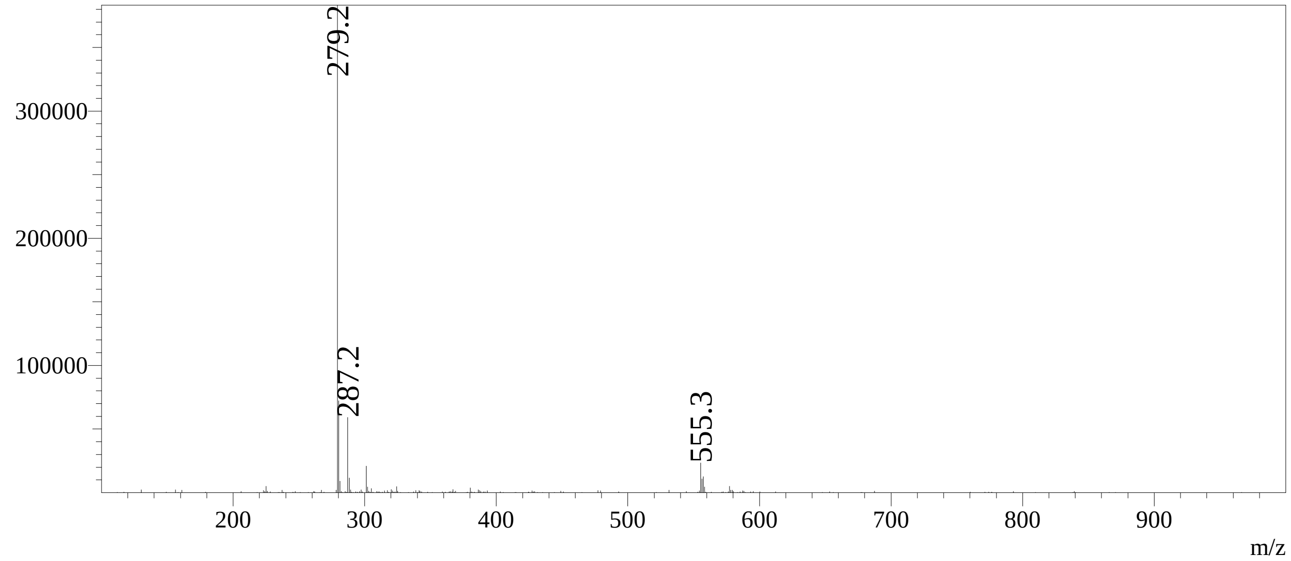

Confidential, for research only not for regulatory filing

HPLC REPORT

Compound ID : GK-D3
Sample ID : EW13998-13-P1B
Vial# : 44
Injection Volumn : 5
Filename : D:\DATA\2018\1809\180912\EW13998-13-P1B.lcd
Method Name : D:\METHOD\10-80CD_4min.lcm
Instrument : HPLC-J
Run time : 9/12/2018 11:24:47

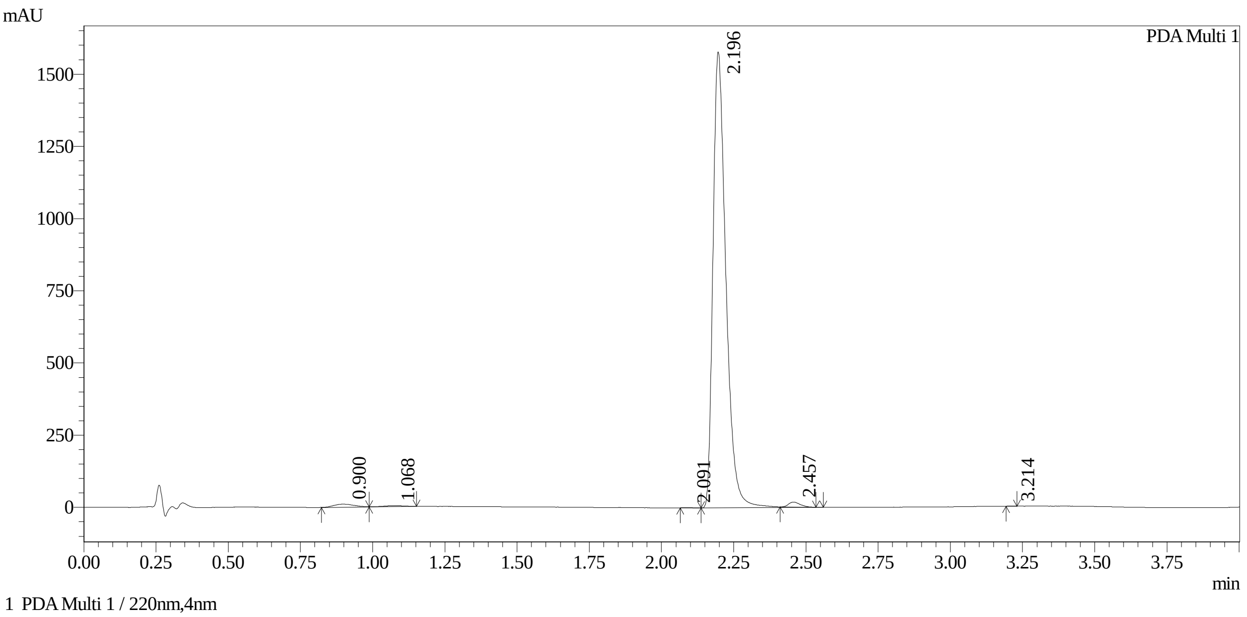

1 PDA Multi 1 / 220nm,4nm

Integration result

PeakTable

PDA Ch1 220nm

Peak#	Ret. Time	USP Width	Resolution	Height	Area	Area %
1	0.900	0.129	0.000	10579	52631	1.045
2	1.068	0.132	1.288	3750	19752	0.392
3	2.091	0.065	10.403	1238	2965	0.059
4	2.196	0.081	1.438	1578797	4909439	97.497
5	2.457	0.075	3.340	17373	49122	0.976
6	3.214	0.054	11.703	910	1580	0.031
Total				1612647	5035488	100.000

Operater : ________________

Date : ________________

PDF created with pdfFactory Pro trial version www.pdffactory.com

HPLC REPORT

Compound ID : GK-D3
Sample ID : EW13998-13-P1B
Filename : D:\DATA\2018\1809\180912\EW13998-13-P1B.lcd
Method Name : D:\METHOD\10-80CD_4min.lcm
Instrument &Column : HPLC-J
Run time : 9/12/2018 11:24:47

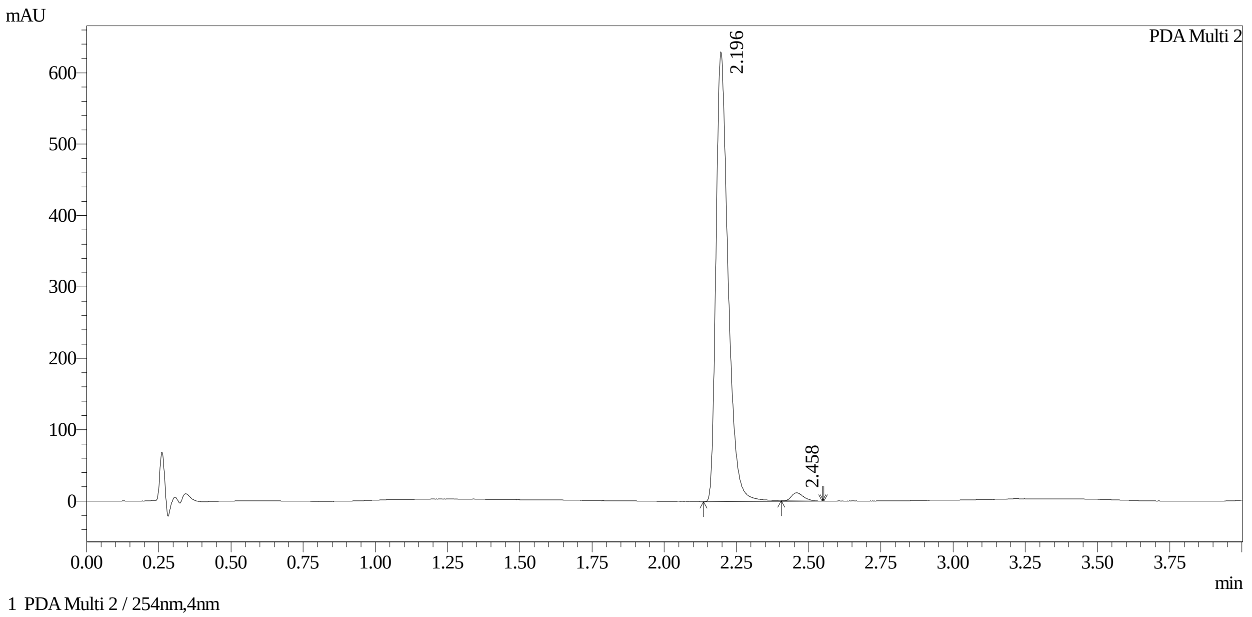

1 PDA Multi 2 / 254nm,4nm

Integration result

PeakTable

PDA Ch2 254nm

Peak#	Ret. Time	USP Width	Resolution	Height	Area	Area %
1	2.196	0.075	0.000	630030	1841575	98.232
2	2.458	0.076	3.473	11460	33140	1.768
Total				641491	1874715	100.000

Operater : ________________

Date : ________________

PDF created with pdfFactory Pro trial version www.pdffactory.com

Confidential, for research only not for regulatory filing

HPLC REPORT

Compound ID : GK-D3
Sample ID : EW13998-13-P1B
Filename : D:\DATA\2018\1809\180912\EW13998-13-P1B.lcd
Method Name : D:\METHOD\10-80CD_4min.lcm
Instrument &Column : HPLC-J
Run time : 9/12/2018 11:24:47

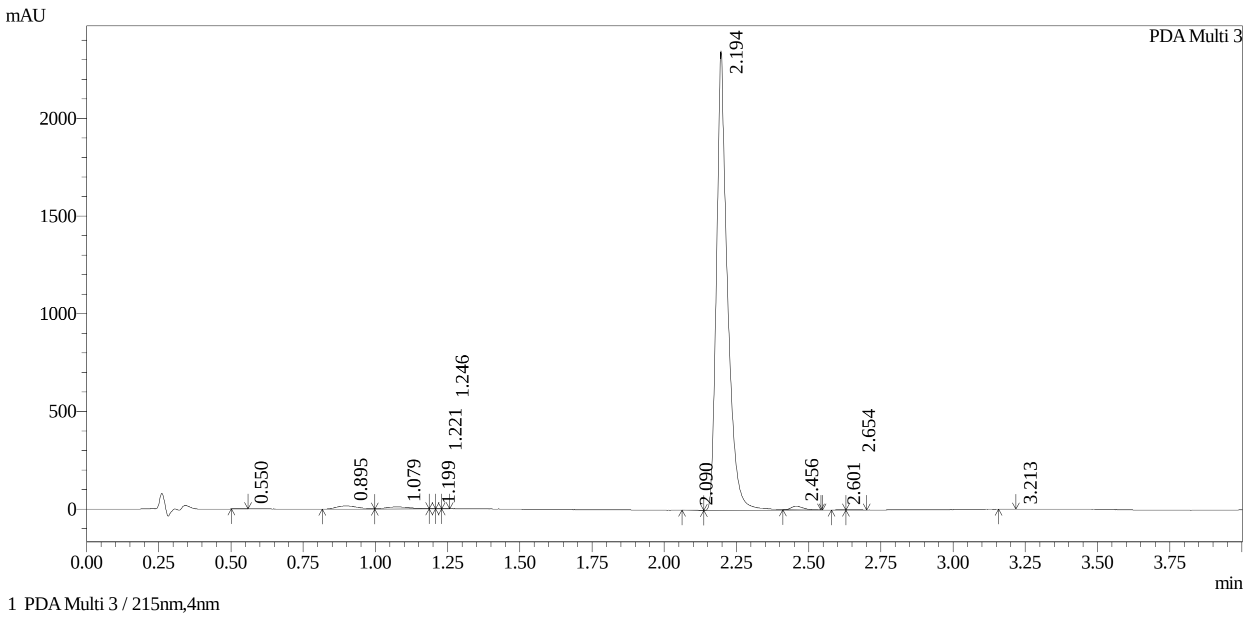

1 PDA Multi 3 / 215nm,4nm

Integration result

PeakTable

PDA Ch3 215nm

Peak#	Ret. Time	USP Width	Resolution	Height	Area	Area %
1	0.550	0.051	0.000	374	1072	0.017
2	0.895	0.136	3.680	16239	85439	1.328
3	1.079	0.159	1.247	10201	63483	0.986
4	1.199	0.085	0.986	1627	1935	0.030
5	1.221	0.160	0.185	1085	1329	0.021
6	1.246	0.100	0.193	814	1222	0.019
7	2.090	0.063	10.340	1204	2596	0.040
8	2.194	0.069	1.577	2346838	6222010	96.677
9	2.456	0.075	3.645	17936	50353	0.782
10	2.601	0.272	0.836	556	1458	0.023
11	2.654	0.092	0.289	1357	3231	0.050
12	3.213	0.073	6.778	895	1719	0.027
Total				2399127	6435848	100.000

Operater : ________________

Date : ________________

PDF created with pdfFactory Pro trial version www.pdffactory.com

Confidential. For research only, Not for regulatory filing.

[redacted]

Compound ID: GK-D4

EW13860-31-P1A DMSO Bruker_D_400MHz

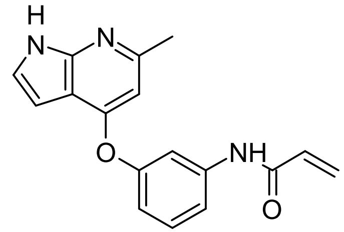

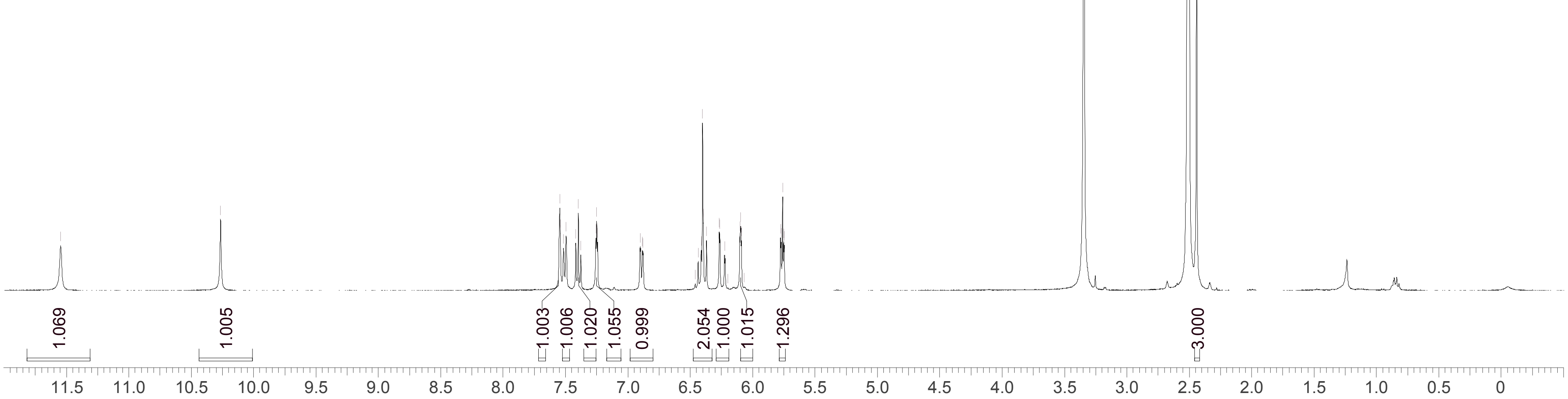

Supervisor:Rongfeng Zhao

Operator:

Date:

[redacted]

Confidential, for research only not for regulatory filing

LCMS REPORT

Compound ID : GK-D4
Sample ID : EW13860-31-P1B
Injection Vol : 2ul
Location : vial56
Acq Method : d:\method\5-95CD_R_220&254_POS.lcm
Org DataFile : D:\DATA\1808\180831\EW13860-31-P1B.lcd
Injection Date : 8/31/2018 3:17:45 PM
Instrument :LCMS-W 17-102

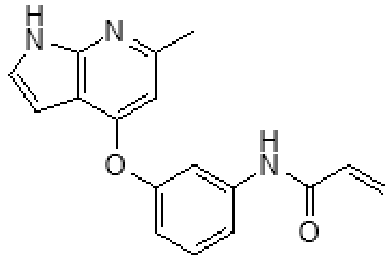

Exact Mass: 293.12

Chromatogram

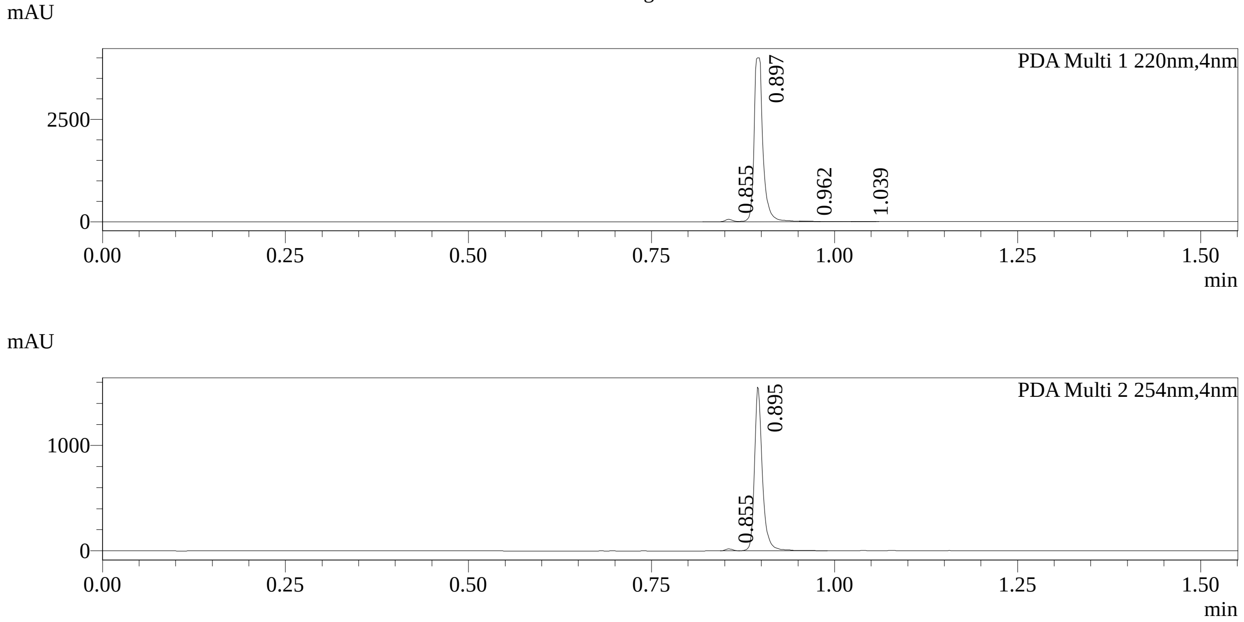

1 PDA Multi 1 / 220nm,4nm
2 PDA Multi 2 / 254nm,4nm

MS Chromatogram

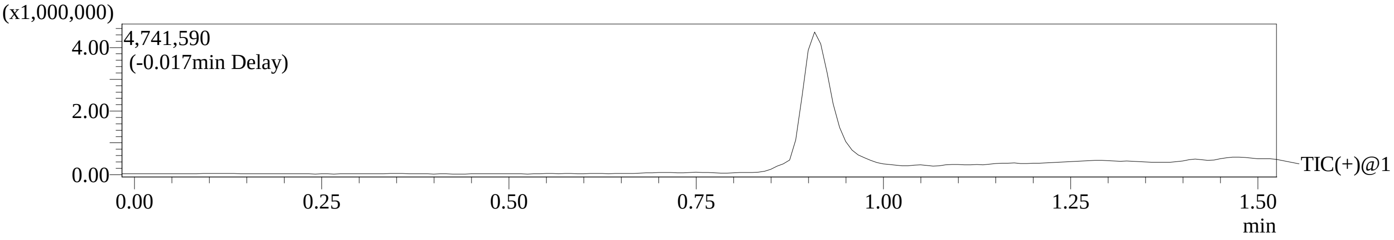

Integration Result

Peak Table

PDA Ch1 220nm

Peak#	Ret. Time	Height	Height%	USP Width	Area	Area%
1	0.855	58693	1.441	0.021	42541	1.214
2	0.897	4000534	98.239	0.021	3451856	98.494
3	0.962	6744	0.166	0.023	5078	0.145
4	1.039	6273	0.154	0.024	5154	0.147

Peak Table

PDA Ch2 254nm

Peak#	Ret. Time	Height	Height%	USP Width	Area	Area%
1	0.855	19890	1.263	0.020	13855	1.155
2	0.895	1555179	98.737	0.021	1185208	98.845

Operator:_______________

Date:___________________

Mass Spectrum

RetTime: 0.858 Datafile: D:\DATA\1808\180831\EW13860-31-P1B.lcd

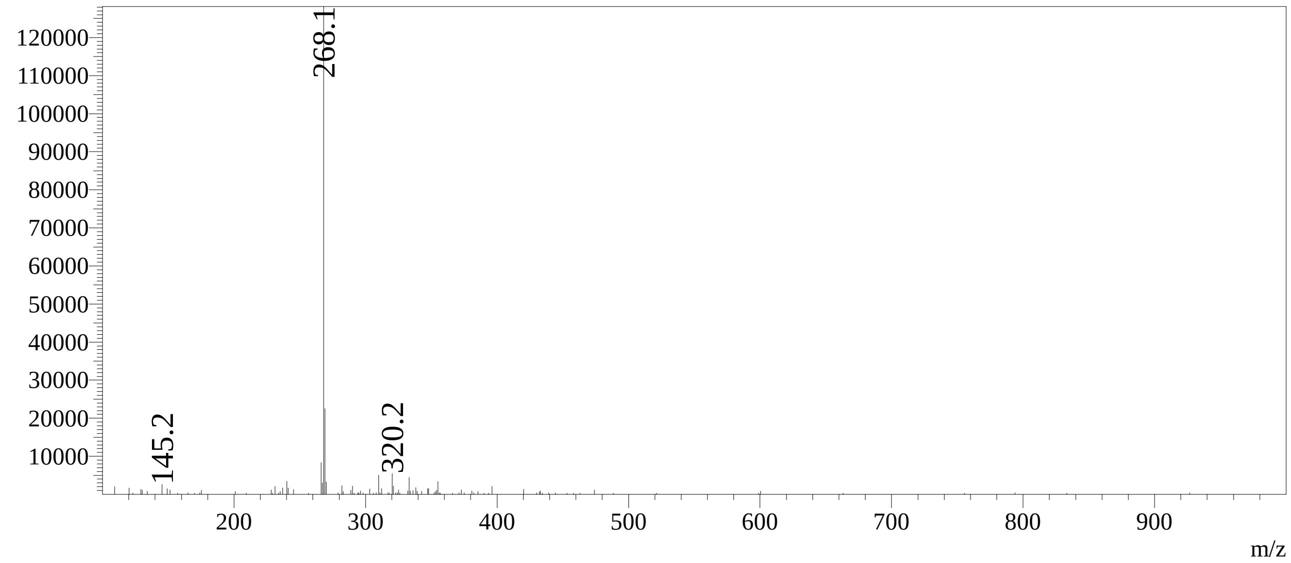

RetTime: 0.900 Datafile: D:\DATA\1808\180831\EW13860-31-P1B.lcd

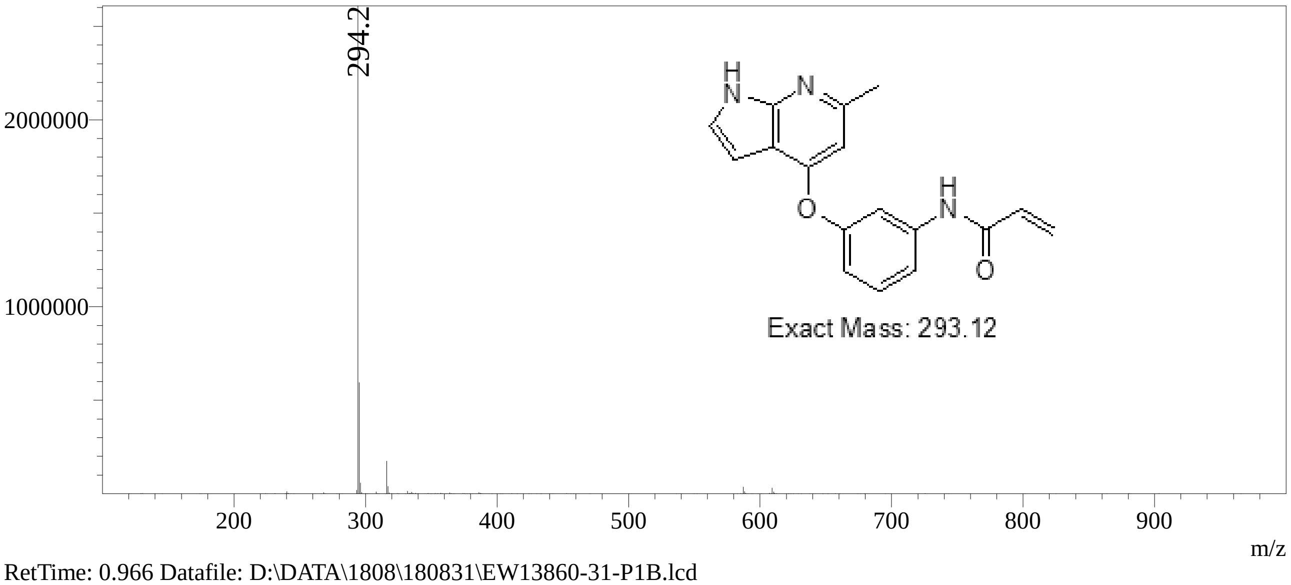

RetTime: 0.966 Datafile: D:\DATA\1808\180831\EW13860-31-P1B.lcd

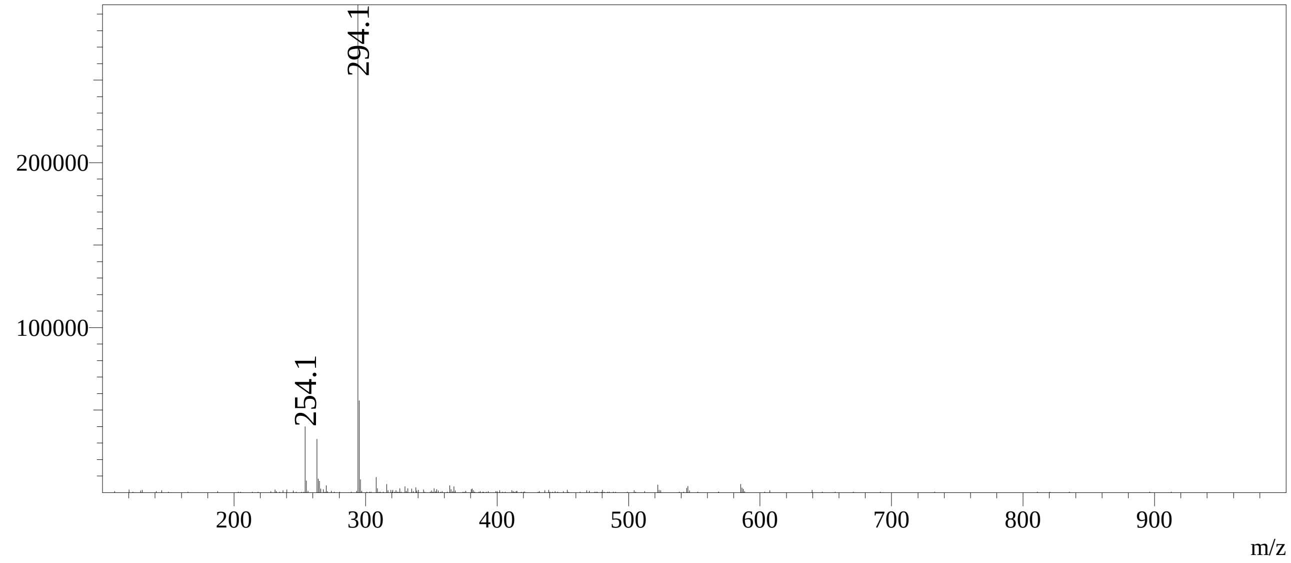

Confidential, for research only not for regulatory filing

RetTime: 1.041 Datafile: D:\DATA\1808\180831\EW13860-31-P1B.lcd

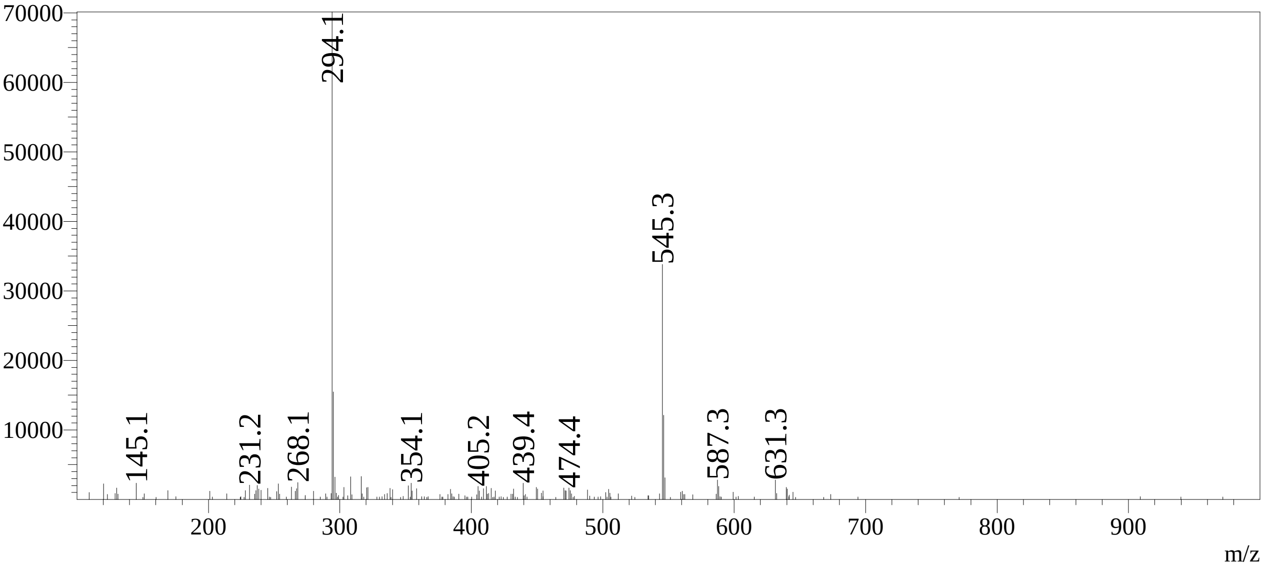

Confidential, for research only not for regulatory filing

HPLC REPORT

Compound ID : GK-D4
Sample ID : EW13860-31-P1B
Vial# : 60
Injection Volumn : 1
Filename : D:\DATA\2018\1809\180904\EW13860-31-P1B.lcd
Method Name : D:\Method\10-80CD_4min.lcm
Instrument : HPLC-J
Run time : 9/4/2018 11:15:53

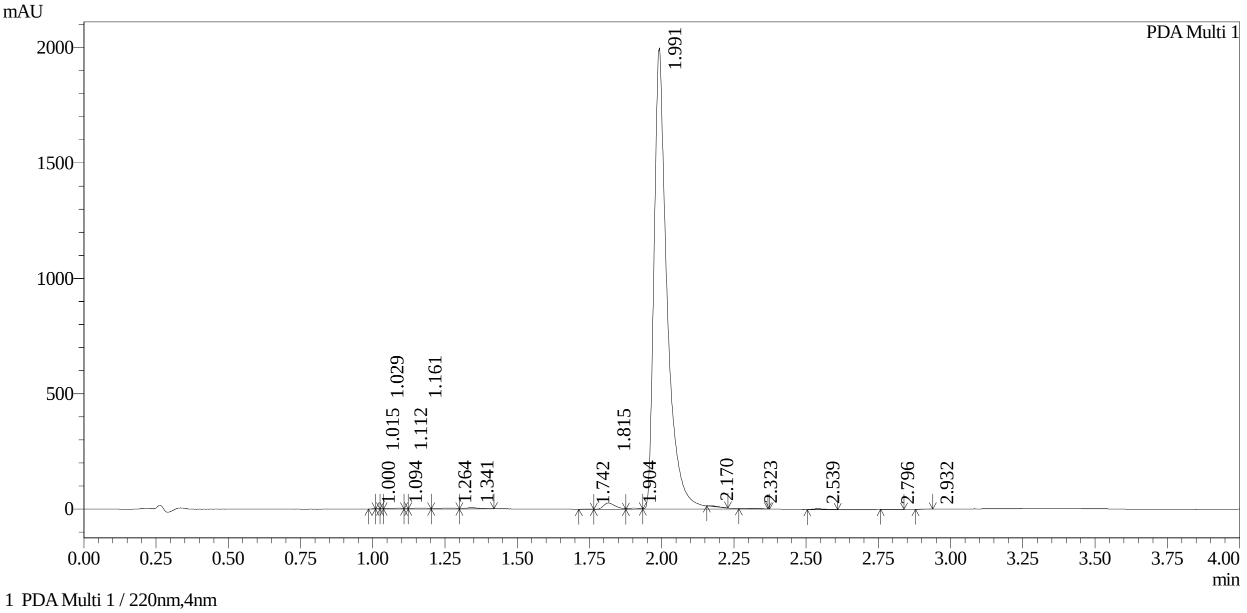

1 PDA Multi 1 / 220nm,4nm

Integration result

PeakTable

PDA Ch1 220nm

Peak#	Ret. Time	USP Width	Resolution	Height	Area	Area %
1	1.000	0.000	0.000	882	1038	0.016
2	1.015	0.046	0.000	1132	1042	0.016
3	1.029	0.105	0.195	1402	1002	0.015
4	1.094	0.238	0.374	3368	11549	0.177
5	1.112	1.158	0.026	3009	2511	0.039
6	1.161	0.329	0.066	4308	16408	0.252
7	1.264	0.191	0.396	3941	17767	0.273
8	1.341	0.099	0.532	4918	18467	0.284
9	1.742	0.050	5.352	690	1302	0.020
10	1.815	0.075	1.166	26785	75588	1.161
11	1.904	0.091	1.067	4586	11550	0.177
12	1.991	0.081	1.019	1999156	6322383	97.140
13	2.170	0.061	2.512	2154	6264	0.096
14	2.323	0.070	2.331	2742	7068	0.109
15	2.539	0.086	2.778	3273	10222	0.157
16	2.796	0.088	2.960	1040	3145	0.048
17	2.932	0.058	1.875	335	1204	0.019
Total				2063721	6508509	100.000

PDF created with pdfFactory Pro trial version www.pdffactory.com

Operater : ________________

Date : ________________

PDF created with pdfFactory Pro trial version www.pdffactory.com

Confidential, for research only not for regulatory filing

HPLC REPORT

Compound ID : GK-D4
Sample ID : EW13860-31-P1B
Filename : D:\DATA\2018\1809\180904\EW13860-31-P1B.lcd
Method Name : D:\Method\10-80CD_4min.lcm
Instrument &Column : HPLC-J
Run time : 9/4/2018 11:15:53

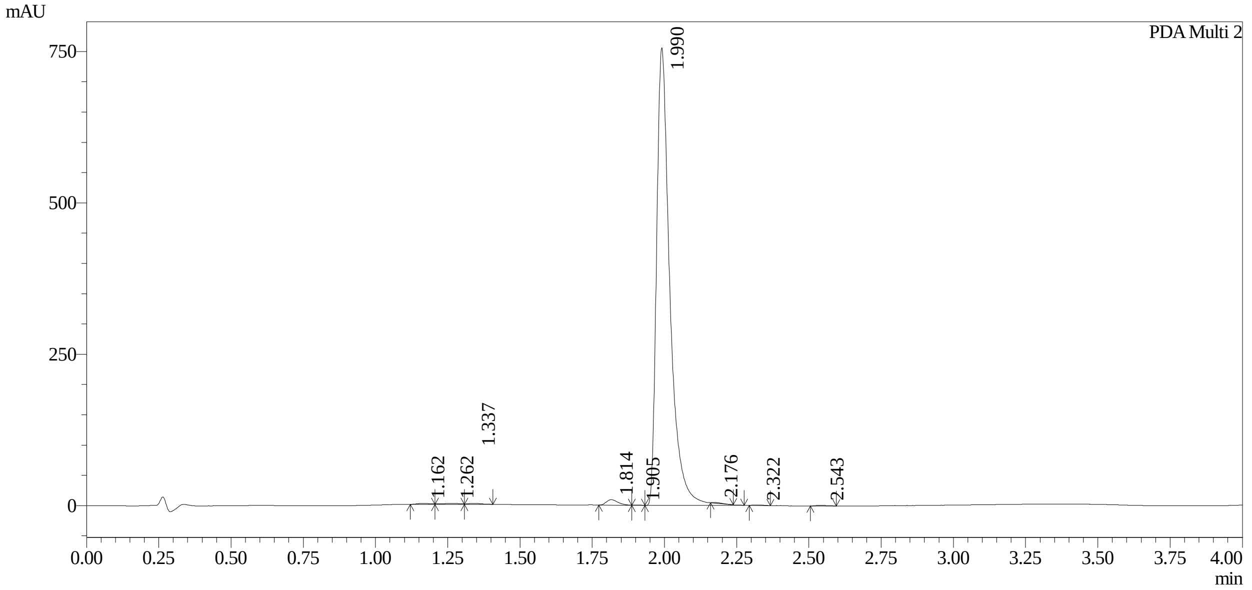

1 PDA Multi 2 / 254nm,4nm

Integration result

PeakTable

PDA Ch2 254nm

Peak#	Ret. Time	USP Width	Resolution	Height	Area	Area %
1	1.162	0.102	0.000	1539	4894	0.198
2	1.262	0.114	0.931	1683	6803	0.275
3	1.337	0.093	0.721	1617	5131	0.207
4	1.814	0.074	5.732	8948	25072	1.013
5	1.905	0.058	1.376	685	1278	0.052
6	1.990	0.080	1.230	755714	2424499	97.977
7	2.176	0.058	2.694	875	2115	0.085
8	2.322	0.065	2.386	841	1908	0.077
9	2.543	0.081	3.015	1043	2846	0.115
Total				772945	2474548	100.000

Operater : ________________

Date : ________________

PDF created with pdfFactory Pro trial version www.pdffactory.com

Confidential, for research only not for regulatory filing

HPLC REPORT

Compound ID : GK-D4
Sample ID : EW13860-31-P1B
Filename : D:\DATA\2018\1809\180904\EW13860-31-P1B.lcd
Method Name : D:\Method\10-80CD_4min.lcm
Instrument &Column : HPLC-J
Run time : 9/4/2018 11:15:53

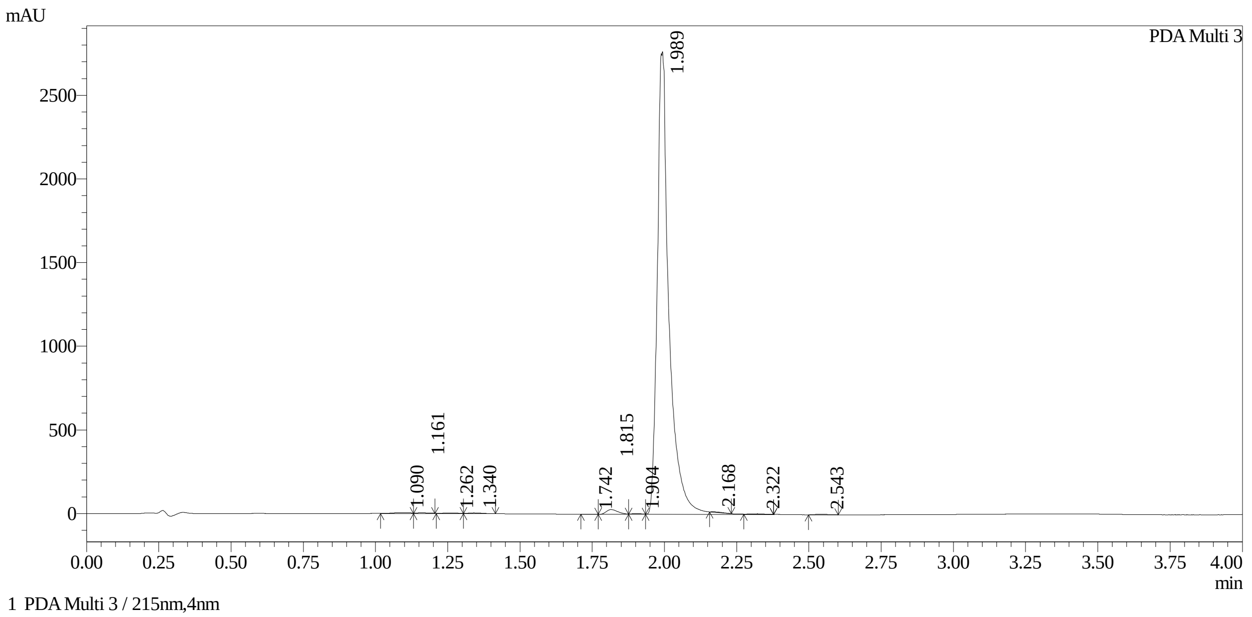

1 PDA Multi 3 / 215nm,4nm

Integration result

PeakTable

PDA Ch3 215nm

Peak#	Ret. Time	USP Width	Resolution	Height	Area	Area %
1	1.090	0.177	0.000	6264	29318	0.364
2	1.161	0.197	0.380	5170	18836	0.234
3	1.262	0.275	0.426	4134	17776	0.220
4	1.340	0.090	0.432	4982	16549	0.205
5	1.742	0.053	5.606	944	1817	0.023
6	1.815	0.076	1.133	28531	80828	1.003
7	1.904	0.081	1.135	4815	12228	0.152
8	1.989	0.059	1.222	2753307	7859495	97.493
9	2.168	0.064	2.910	2052	6811	0.084
10	2.322	0.067	2.354	2924	7318	0.091
11	2.543	0.087	2.875	3551	10609	0.132
Total				2816674	8061586	100.000

Operater : ________________

Date : ________________

PDF created with pdfFactory Pro trial version www.pdffactory.com

EW13860-31-P1B DMSO NOE

[redacted]

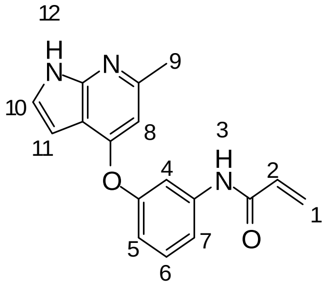

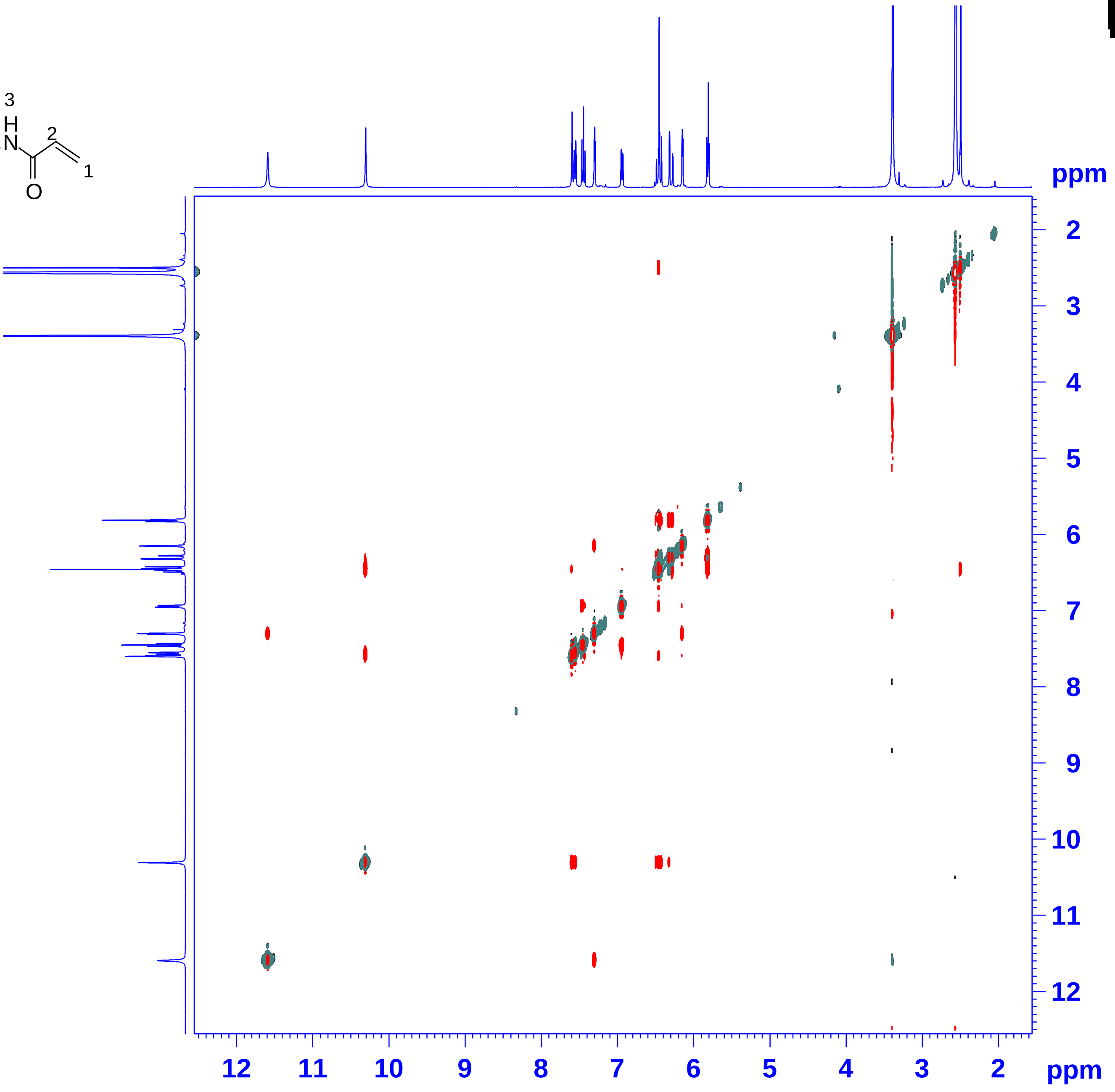

NAME	EW13860-31-P1B
EXPNO	2
PROCNO	1
Date_	20180903
Time	21.14 h
INSTRUM	spect
PROBHD	Z116098_0689 (
PULPROG	noesygpph
TD	2048
SOLVENT	DMSO
NS	8
DS	8
SWH	4401.409 Hz
FIDRES	4.298251 Hz
AQ	0.2327028 se
RG	112.48
DW	113.600 us
DE	6.50 us
TE	300.1 K
D0	0.00010087 se
D1	2.00000000 se
D8	0.80000001 se
D16	0.00020000 se
IN0	0.00022720 se
ND0	1
TD	200
SFO1	400.1728 MH
FIDRES	22.007042 Hz
SW	10.999 pp
FnMODE	TPPI
SI	1024
SF	400.1699771 MH
WDW	QSINE
SSB	2
LB	0.00 Hz
GB	0
PC	1.00
SI	1024
MC2	TPPI
SF	400.1699772 MH
WDW	QSINE
SSB	2
LB	0.00 Hz
GB	0

Confidential, for research only not for regulatory filing

EW13860-31-P1B DMSO NOE

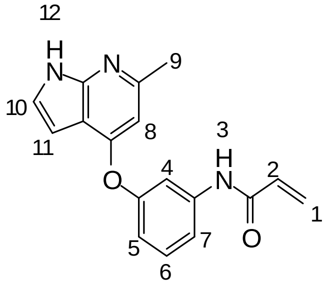

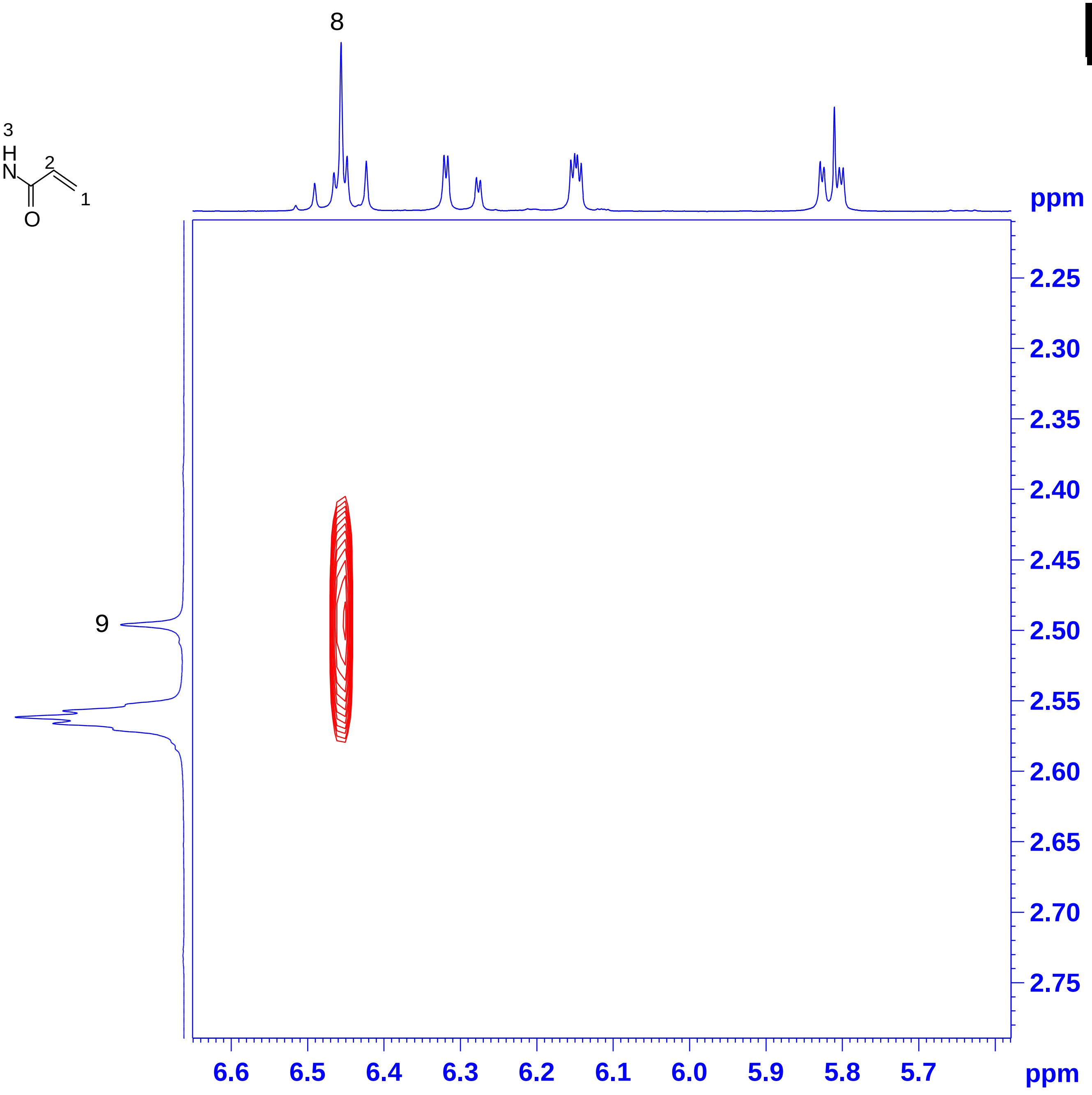

[redacted]

NAME	EW13860-31-P1B	
EXPNO	2	
PROCNO	1	
Date_	20180903	
Time	21.14	h
INSTRUM	spect	
PROBHD	Z116098_0689 (	
PULPROG	noesygpph	
TD	2048	
SOLVENT	DMSO	
NS	8	
DS	8	
SWH	4401.409	Hz
FIDRES	4.298251	Hz
AQ	0.2327028	se
RG	112.48	
DW	113.600	us
DE	6.50	us
TE	300.1	K
D0	0.00010087	se
D1	2.00000000	se
D8	0.80000001	se
D16	0.00020000	se
IN0	0.00022720	se
ND0	1	
TD	200	
SFO1	400.1728	MH
FIDRES	22.007042	Hz
SW	10.999	pp
FnMODE	TPPI	
SI	1024	
SF	400.1699771	MH
WDW	QSINE	
SSB	2	
LB	0.00	Hz
GB	0	
PC	1.00	
SI	1024	
MC2	TPPI	
SF	400.1699772	MH
WDW	QSINE	
SSB	2	
LB	0.00	Hz
GB	0	

Confidential, for research only not for regulatory filing

EW13860-31-P1B DMSO NOE

[redacted]

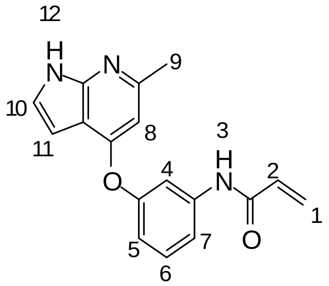

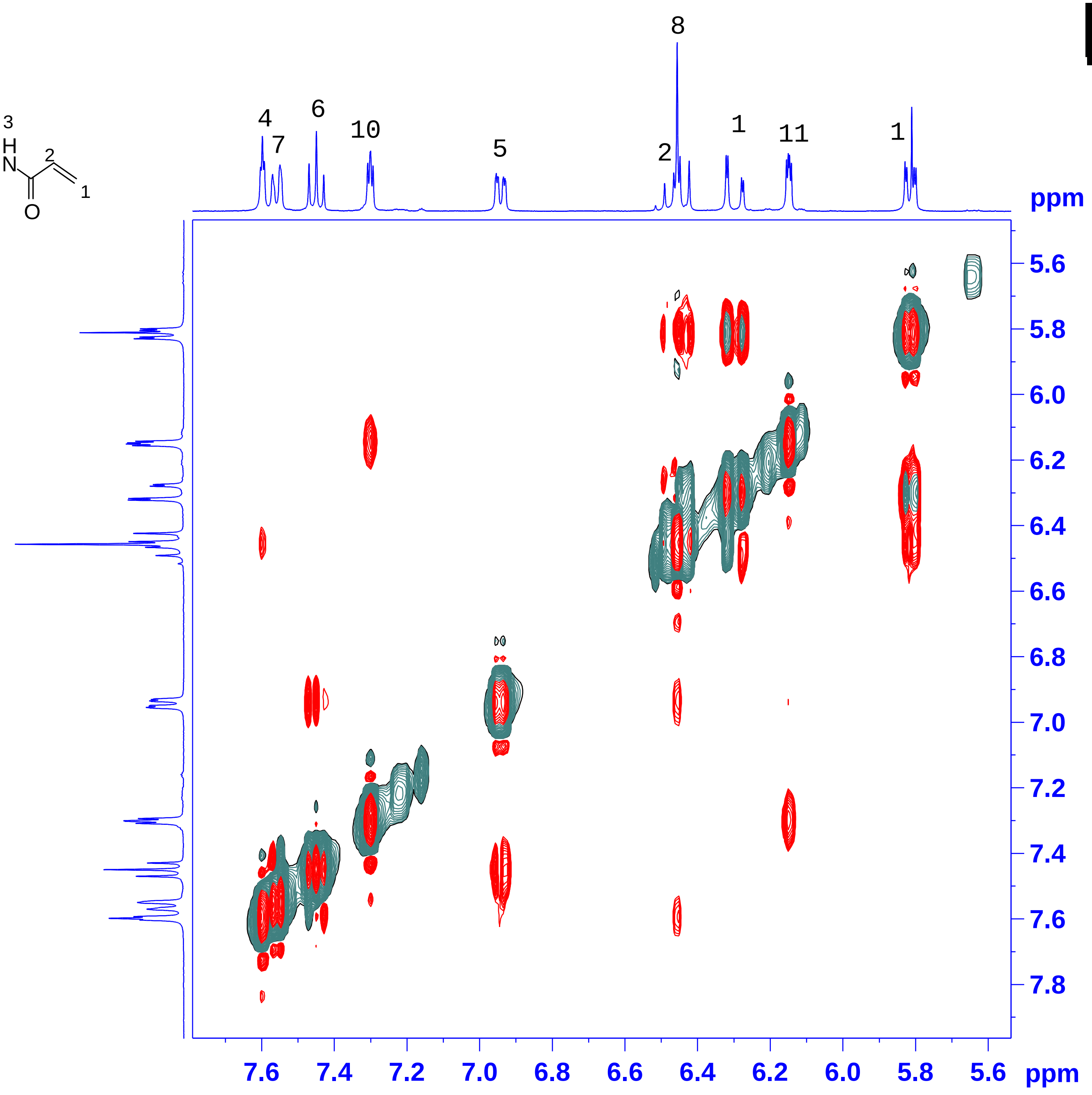

NAME	EW13860-31-P1B	
EXPNO	2	
PROCNO	1	
Date_	20180903	
Time	21.14	h
INSTRUM	spect	
PROBHD	Z116098_0689 (	
PULPROG	noesygpph	
TD	2048	
SOLVENT	DMSO	
NS	8	
DS	8	
SWH	4401.409	Hz
FIDRES	4.298251	Hz
AQ	0.2327028	sec
RG	112.48	
DW	113.600	usec
DE	6.50	usec
TE	300.1	K
D0	0.00010087	sec
D1	2.00000000	sec
D8	0.80000001	sec
D16	0.00020000	sec
IN0	0.00022720	sec
ND0	1	
TD	200	
SFO1	400.1728	MHz
FIDRES	22.007042	Hz
SW	10.999	ppm
FnMODE	TPPI	
SI	1024	
SF	400.1699771	MHz
WDW	QSINE	
SSB	2	
LB	0.00	Hz
GB	0	
PC	1.00	
SI	1024	
MC2	TPPI	
SF	400.1699772	MHz
WDW	QSINE	
SSB	2	
LB	0.00	Hz
GB	0	

Confidential, for research only not for regulatory filing

EW13860-31-P1B DMSO NOE

[redacted]

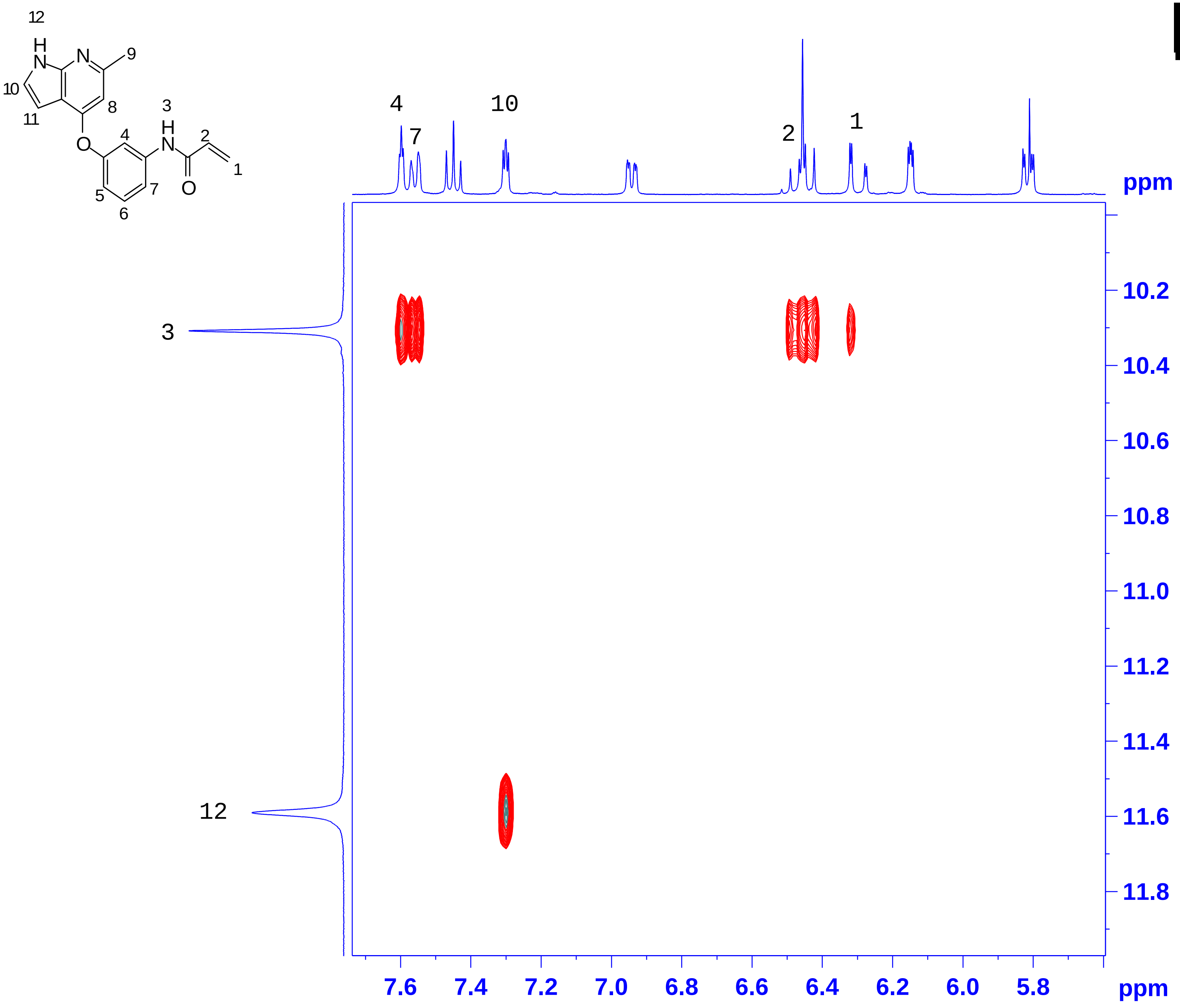

NAME	EW13860-31-P1B	
EXPNO	2	
PROCNO	1	
Date_	20180903	
Time	21.14	h
INSTRUM	spect	
PROBHD	Z116098_0689 (	
PULPROG	noesygpph	
TD	2048	
SOLVENT	DMSO	
NS	8	
DS	8	
SWH	4401.409	Hz
FIDRES	4.298251	Hz
AQ	0.2327028	se
RG	112.48	
DW	113.600	use
DE	6.50	use
TE	300.1	K
D0	0.00010087	se
D1	2.00000000	se
D8	0.80000001	se
D16	0.00020000	se
IN0	0.00022720	se
ND0	1	
TD	200	
SFO1	400.1728	MH
FIDRES	22.007042	Hz
SW	10.999	pp
FnMODE	TPPI	
SI	1024	
SF	400.1699771	MH
WDW	QSINE	
SSB	2	
LB	0.00	Hz
GB	0	
PC	1.00	
SI	1024	
MC2	TPPI	
SF	400.1699772	MH
WDW	QSINE	
SSB	2	
LB	0.00	Hz
GB	0	

Confidential, for research only not for regulatory filing

Confidential. For research only, Not for regulatory filing.

[redacted]

Compound ID: GK-D5

EW13860-33-P1A DMSO Bruker_F_400MHz

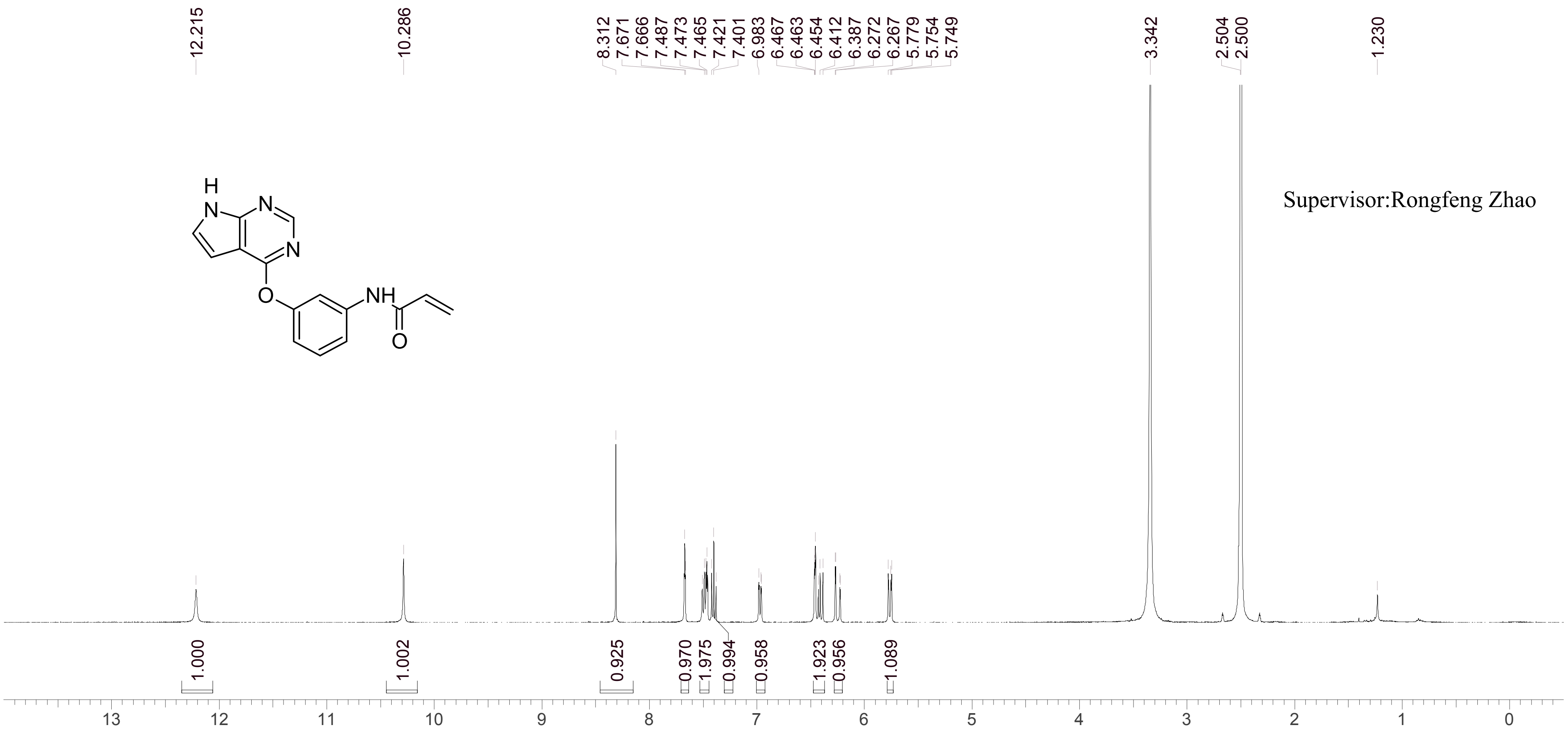

Supervisor:Rongfeng Zhao

Operator: Date:

[redacted]

Confidential, for research only not for regulatory filing

LCMS REPORT

Compound ID : GK-D5
Sample ID : EW13860-33-P1B
Injection Vol : 5ul
Location : vial9
Acq Method : d:\method\5-95CD_R_220&254_POS.lcm
Org DataFile : D:\DATA\1809\180903\EW13860-33-P1B.lcd
Injection Date : 9/3/2018 8:00:36 PM
Instrument :LCMS-W 17-102

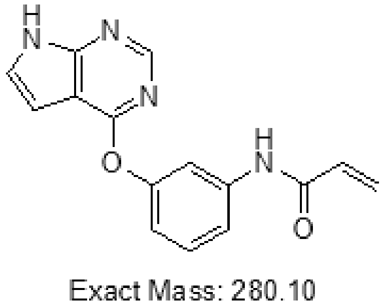

Chromatogram

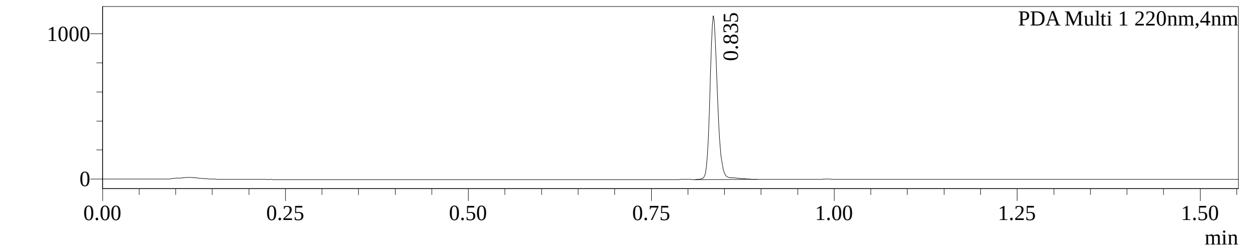

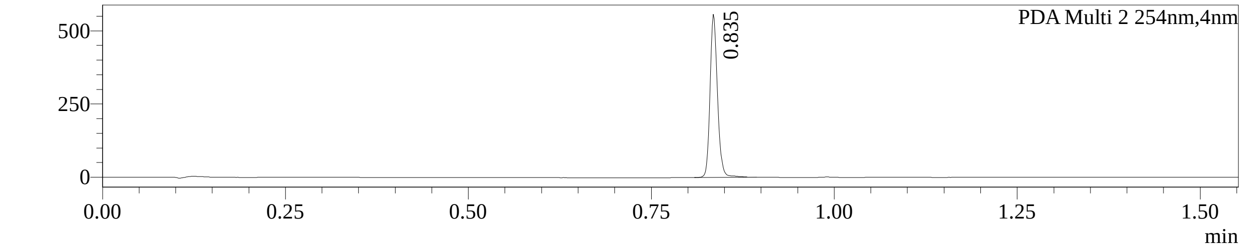

1 PDA Multi 1 / 220nm,4nm
2 PDA Multi 2 / 254nm,4nm

MS Chromatogram

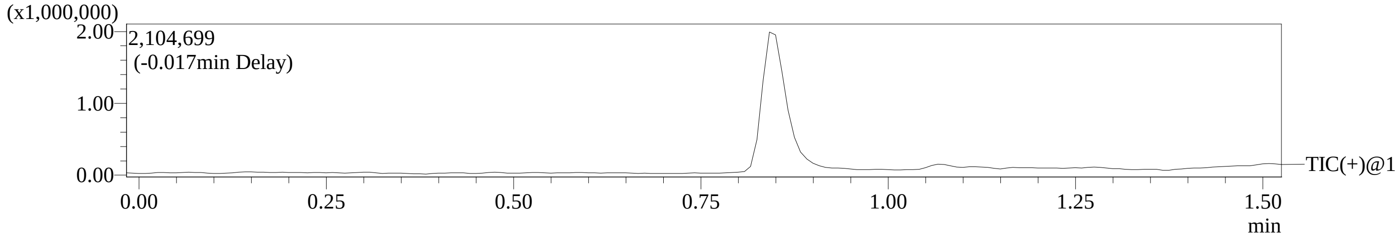

Integration Result

Peak Table

PDA Ch1 220nm

Peak#	Ret. Time	Height	Height%	USP Width	Area	Area%
1	0.835	1126303	100.000	0.021	815742	100.000

Peak Table

PDA Ch2 254nm

Peak#	Ret. Time	Height	Height%	USP Width	Area	Area%
1	0.835	557590	100.000	0.021	399685	100.000

Confidential, for research only not for regulatory filing

Operator:______________

Date:__________________

Confidential, for research only not for regulatory filing

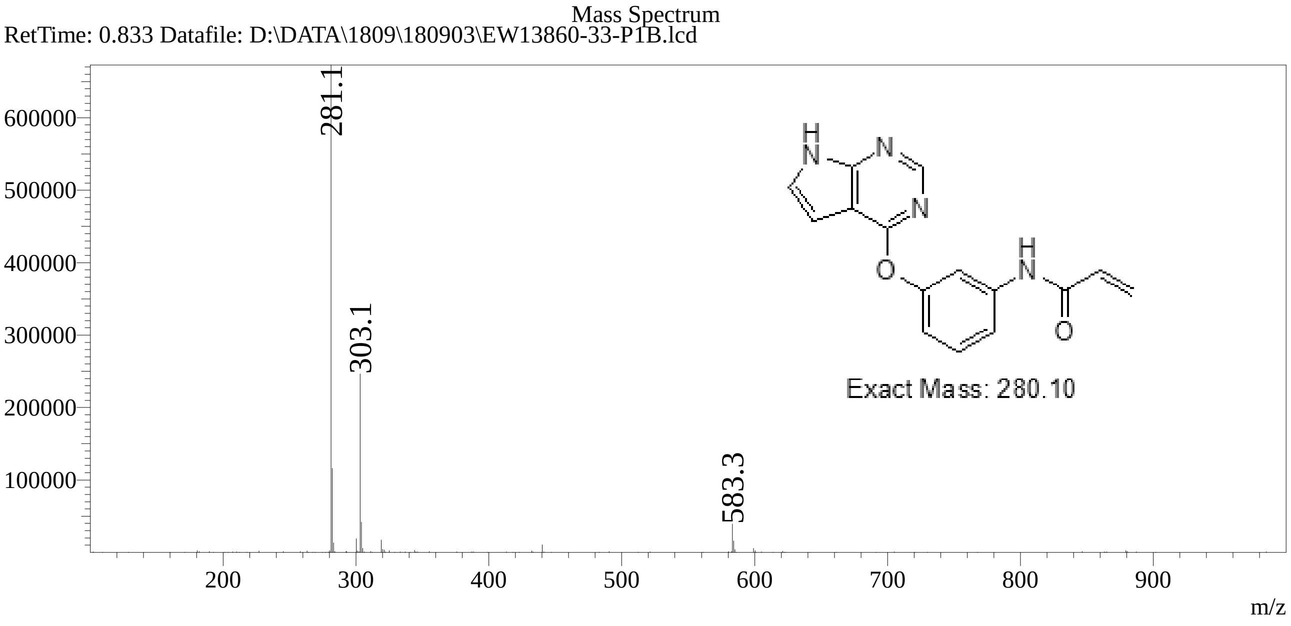

Confidential, for research only not for regulatory filing

HPLC REPORT

Compound ID : GK-D5
Sample ID : EW13860-33-P1B
Vial# : 56
Injection Volumn : 4
Filename : D:\DATA\2018\1809\180904\EW13860-33-P1B.lcd
Method Name : D:\Method\10-80CD_4min.lcm
Instrument : HPLC-J
Run time : 9/4/2018 10:56:15

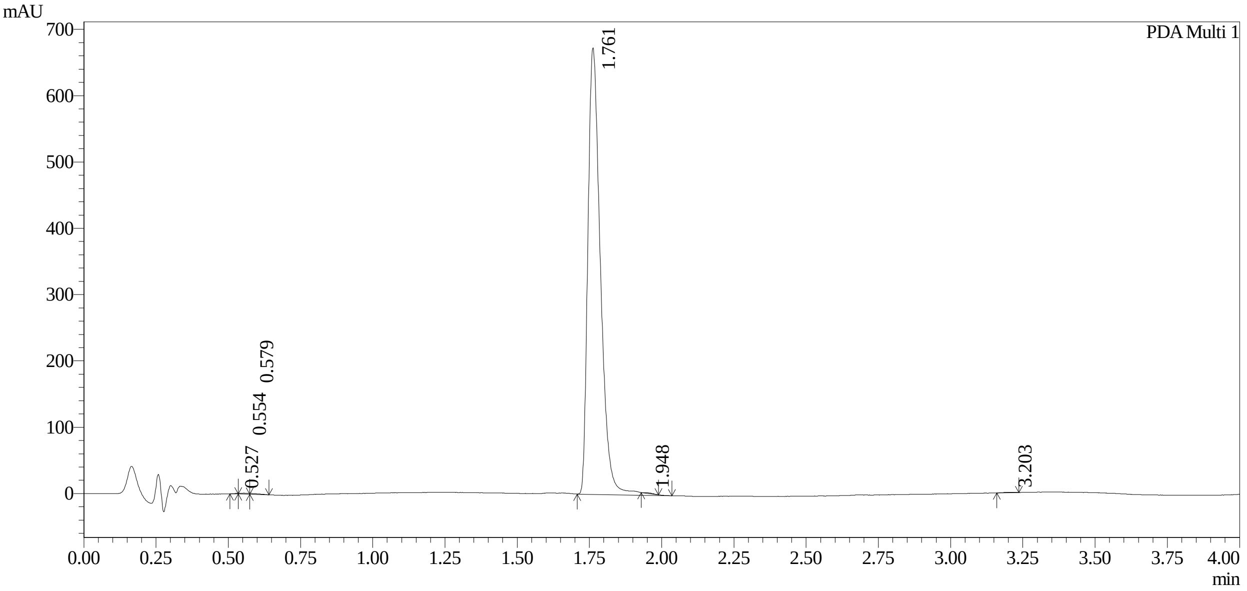

1 PDA Multi 1 / 220nm,4nm

Integration result

PeakTable

PDA Ch1 220nm

Peak#	Ret. Time	USP Width	Resolution	Height	Area	Area %
1	0.527	0.062	0.000	998	1100	0.054
2	0.554	0.169	0.233	1642	3480	0.170
3	0.579	0.119	0.174	1589	3290	0.160
4	1.761	0.079	11.956	673428	2038816	99.441
5	1.948	0.053	2.825	1000	1951	0.095
6	3.203	0.069	20.512	686	1635	0.080
Total				679342	2050272	100.000

Operater : ________________

Date : ________________

PDF created with pdfFactory Pro trial version www.pdffactory.com

Confidential, for research only not for regulatory filing

HPLC REPORT

Compound ID : GK-D5
Sample ID : EW13860-33-P1B
Filename : D:\DATA\2018\1809\180904\EW13860-33-P1B.lcd
Method Name : D:\Method\10-80CD_4min.lcm
Instrument &Column : HPLC-J
Run time : 9/4/2018 10:56:15

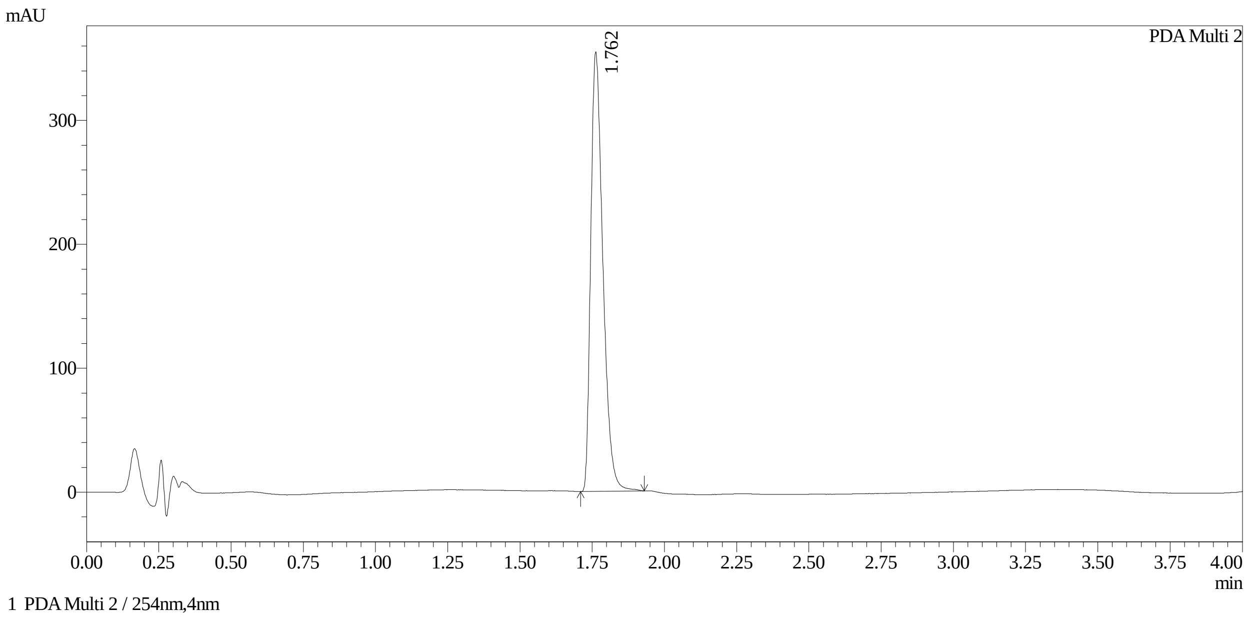

1 PDA Multi 2 / 254nm,4nm

Integration result

PeakTable

PDA Ch2 254nm

Peak#	Ret. Time	USP Width	Resolution	Height	Area	Area %
1	1.762	0.077	0.000	354845	1037414	100.000
Total				354845	1037414	100.000

Operater : ________________

Date : ________________

PDF created with pdfFactory Pro trial version www.pdffactory.com

Confidential, for research only not for regulatory filing

HPLC REPORT

Compound ID : GK-D5
Sample ID : EW13860-33-P1B
Filename : D:\DATA\2018\1809\180904\EW13860-33-P1B.lcd
Method Name : D:\Method\10-80CD_4min.lcm
Instrument &Column : HPLC-J
Run time : 9/4/2018 10:56:15

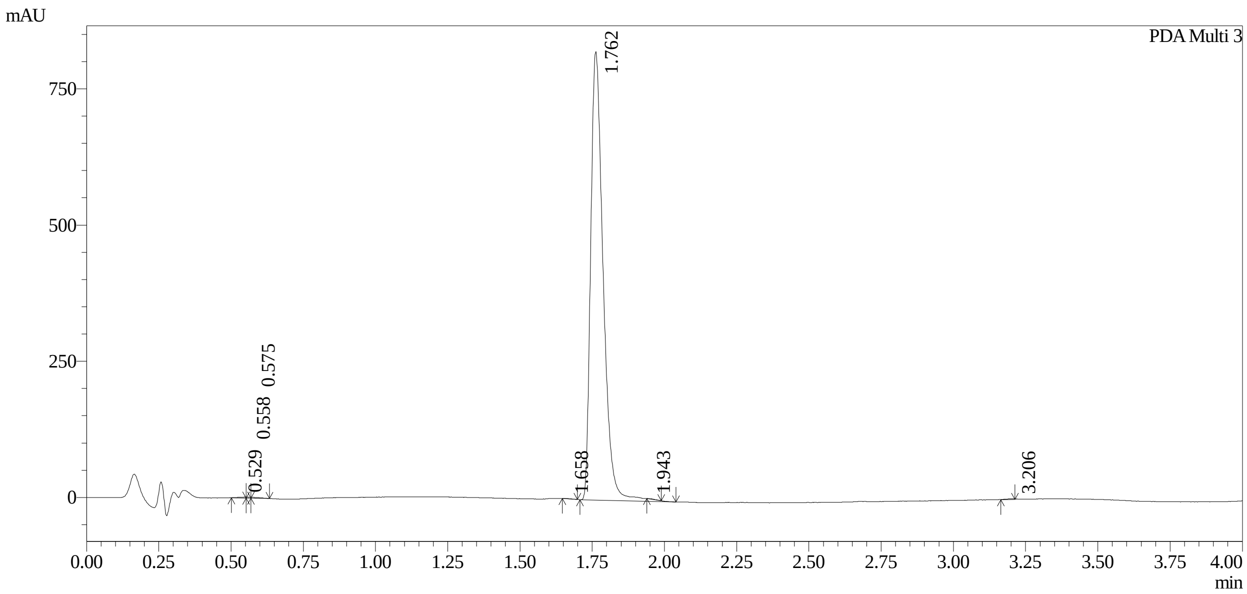

1 PDA Multi 3 / 215nm,4nm

Integration result

PeakTable

PDA Ch3 215nm

Peak#	Ret. Time	USP Width	Resolution	Height	Area	Area %
1	0.529	0.074	0.000	1370	3106	0.125
2	0.558	0.087	0.357	1855	1795	0.072
3	0.575	0.096	0.182	1755	3661	0.148
4	1.658	0.042	15.675	656	1285	0.052
5	1.762	0.078	1.719	822771	2466076	99.455
6	1.943	0.045	2.945	478	1380	0.056
7	3.206	0.057	24.885	946	2287	0.092
Total				829832	2479591	100.000

Operater : ________________

Date : ________________

PDF created with pdfFactory Pro trial version www.pdffactory.com

EW13860-33-P1B DMSO NOE

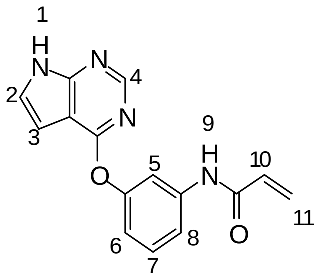

[redacted]

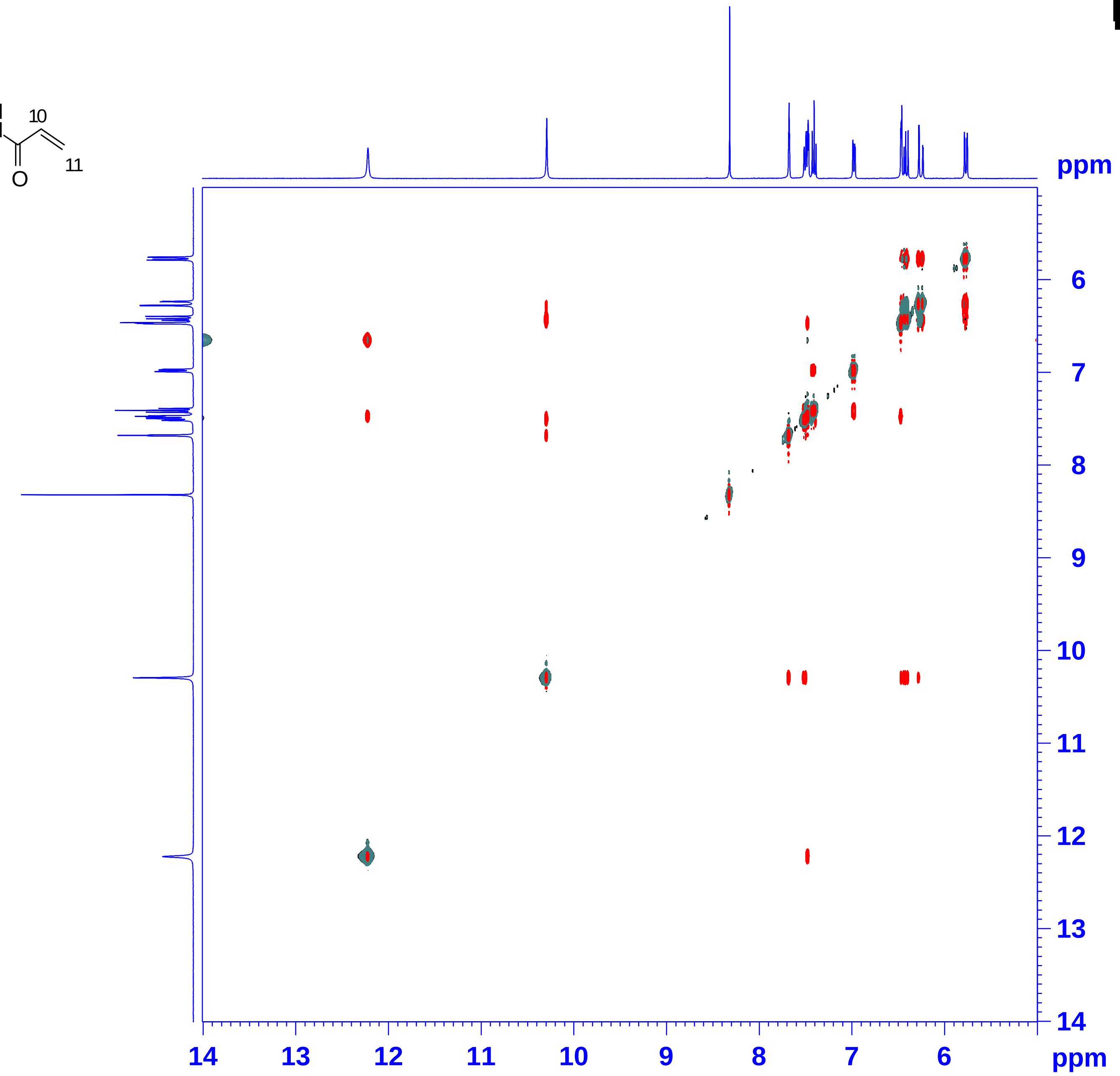

NAME	EW13860-33-P1B	
EXPNO	2	
PROCNO	1	
Date_	20180904	
Time	22.57	h
INSTRUM	spect	
PROBHD	Z116098_0689 (	
PULPROG	noesygpph	
TD	2048	
SOLVENT	DMSO	
NS	12	
DS	8	
SWH	3605.769	Hz
FIDRES	3.521259	Hz
AQ	0.2840393	se
RG	80.69	
DW	138.667	use
DE	6.50	use
TE	300.2	K
D0	0.00012607	se
D1	2.00000000	se
D8	0.80000001	se
D16	0.00020000	se
IN0	0.00027760	se
ND0	1	
TD	200	
SFO1	400.1738	MH
FIDRES	18.011528	Hz
SW	9.002	pp
FnMODE	TPPI	
SI	1024	
SF	400.1699986	MH
WDW	QSINE	
SSB	2	
LB	0.00	Hz
GB	0	
PC	1.00	
SI	1024	
MC2	TPPI	
SF	400.1699975	MH
WDW	QSINE	
SSB	2	
LB	0.00	Hz
GB	0	

Confidential, for research only not for regulatory filing

EW13860-33-P1B DMSO NOE

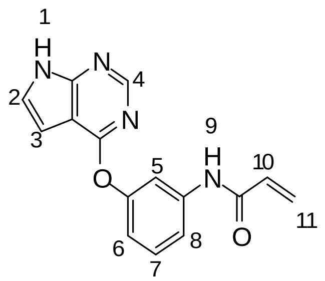

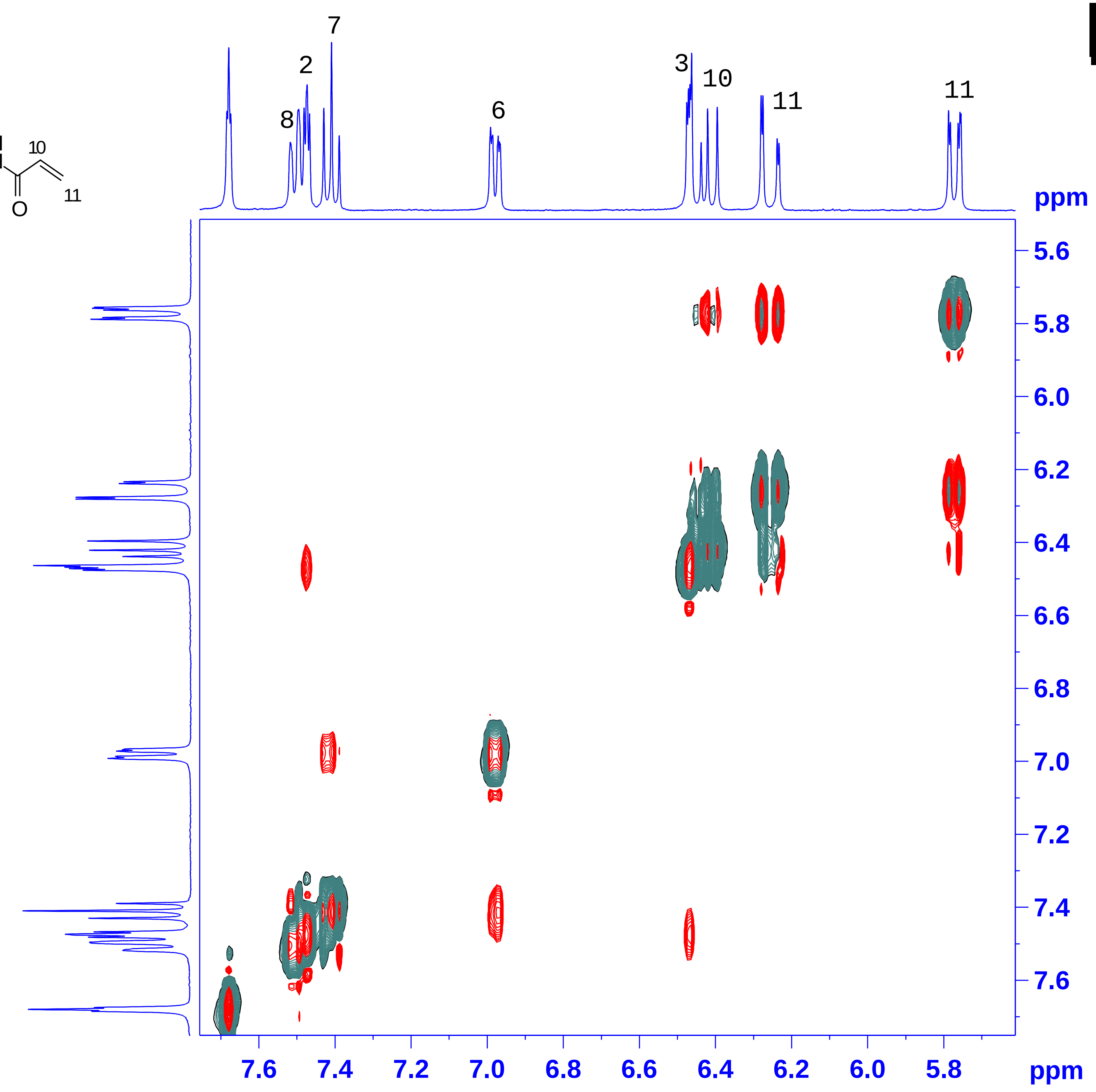

[redacted]

NAME	EW13860-33-P1B	
EXPNO	2	
PROCNO	1	
Date_	20180904	
Time	22.57	h
INSTRUM	spect	
PROBHD	Z116098_0689 (	
PULPROG	noesygpph	
TD	2048	
SOLVENT	DMSO	
NS	12	
DS	8	
SWH	3605.769	Hz
FIDRES	3.521259	Hz
AQ	0.2840393	sec
RG	80.69	
DW	138.667	use
DE	6.50	use
TE	300.2	K
D0	0.00012607	sec
D1	2.00000000	sec
D8	0.80000001	sec
D16	0.00020000	sec
IN0	0.00027760	sec
ND0	1	
TD	200	
SFO1	400.1738	MHz
FIDRES	18.011528	Hz
SW	9.002	ppm
FnMODE	TPPI	
SI	1024	
SF	400.1699986	MHz
WDW	QSINE	
SSB	2	
LB	0.00	Hz
GB	0	
PC	1.00	
SI	1024	
MC2	TPPI	
SF	400.1699975	MHz
WDW	QSINE	
SSB	2	
LB	0.00	Hz
GB	0	

Confidential, for research only not for regulatory filing

EW13860-33-P1B DMSO NOE

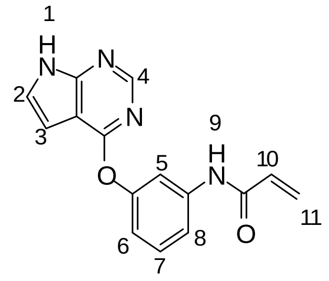

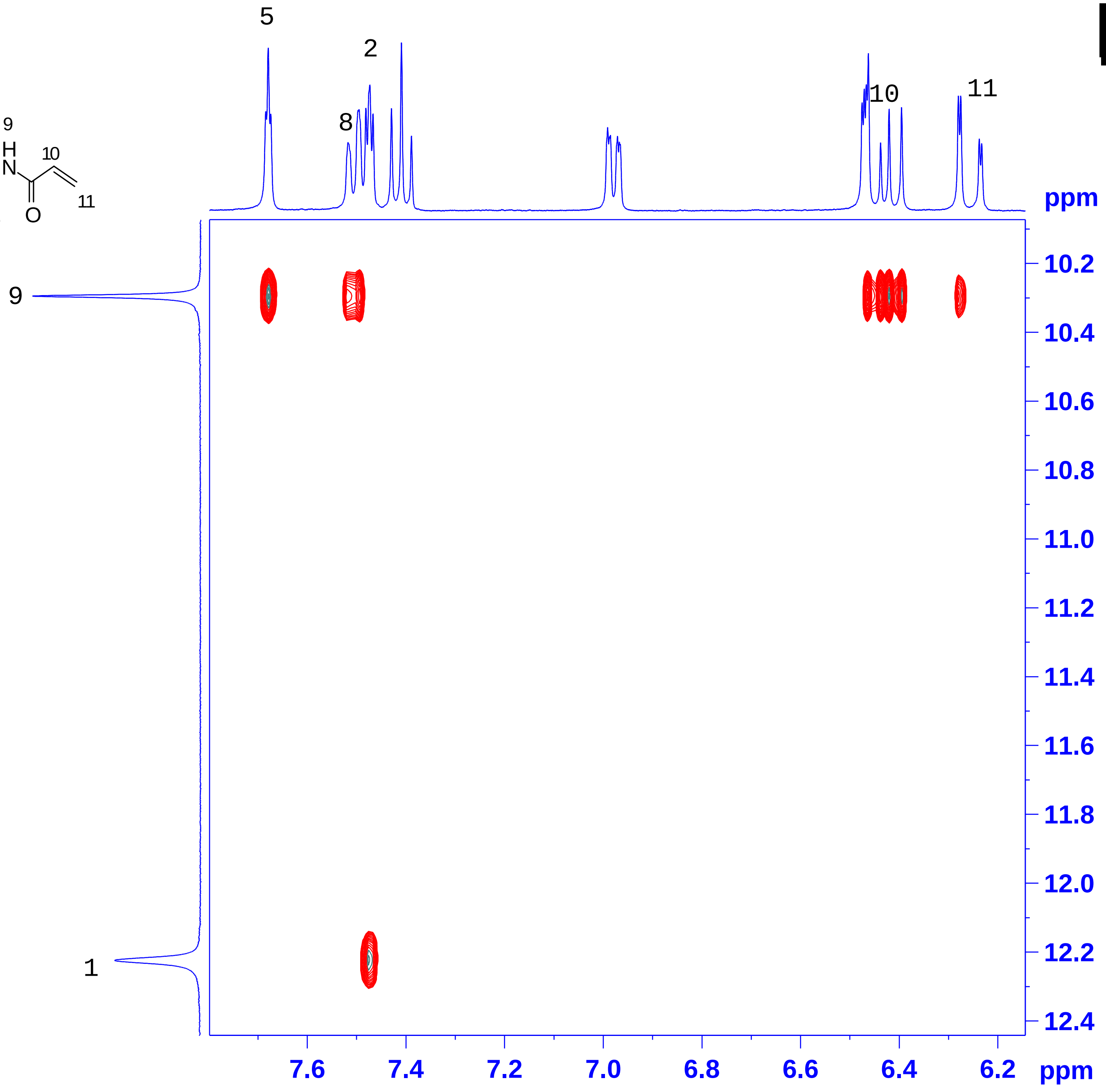

[redacted]

NAME	EW13860-33-P1B	
EXPNO	2	
PROCNO	1	
Date_	20180904	
Time	22.57	h
INSTRUM	spect	
PROBHD	Z116098_0689 (	
PULPROG	noesygpph	
TD	2048	
SOLVENT	DMSO	
NS	12	
DS	8	
SWH	3605.769	Hz
FIDRES	3.521259	Hz
AQ	0.2840393	se
RG	80.69	
DW	138.667	use
DE	6.50	use
TE	300.2	K
D0	0.00012607	se
D1	2.00000000	se
D8	0.80000001	se
D16	0.00020000	se
IN0	0.00027760	se
ND0	1	
TD	200	
SFO1	400.1738	MH
FIDRES	18.011528	Hz
SW	9.002	pp
FnMODE	TPPI	
SI	1024	
SF	400.1699986	MH
WDW	QSINE	
SSB	2	
LB	0.00	Hz
GB	0	
PC	1.00	
SI	1024	
MC2	TPPI	
SF	400.1699975	MH
WDW	QSINE	
SSB	2	
LB	0.00	Hz
GB	0	

Confidential, for research only not for regulatory filing

EW13860-33-P1B DMSO NOE

[redacted]

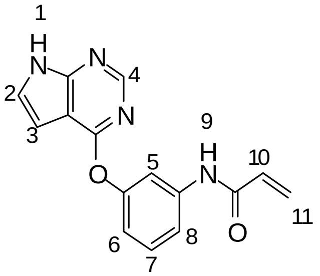

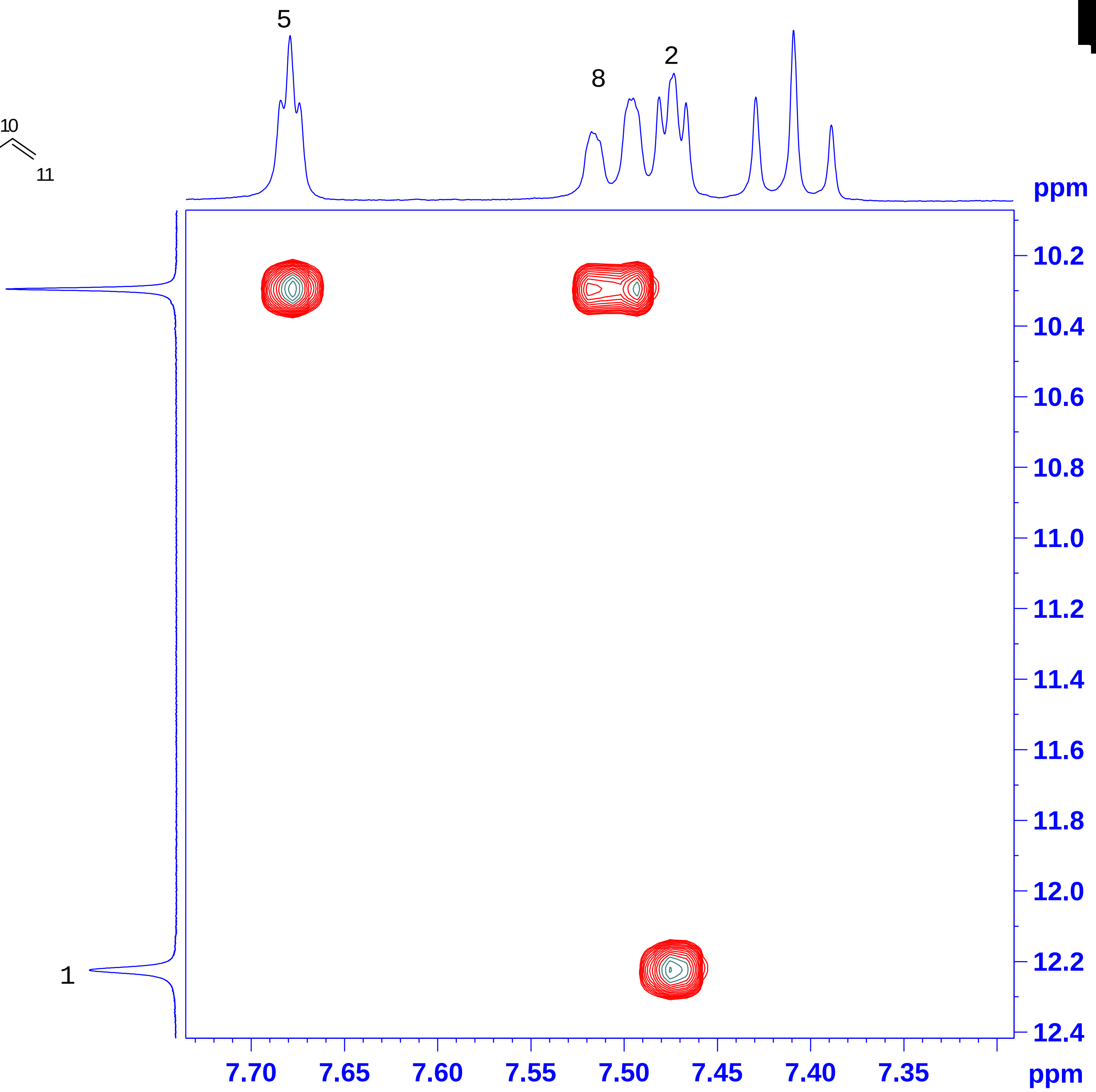

NAME	EW13860-33-P1B	
EXPNO	2	
PROCNO	1	
Date_	20180904	
Time	22.57	h
INSTRUM	spect	
PROBHD	Z116098_0689 (	
PULPROG	noesygpph	
TD	2048	
SOLVENT	DMSO	
NS	12	
DS	8	
SWH	3605.769	Hz
FIDRES	3.521259	Hz
AQ	0.2840393	se
RG	80.69	
DW	138.667	use
DE	6.50	use
TE	300.2	K
D0	0.00012607	se
D1	2.00000000	se
D8	0.80000001	se
D16	0.00020000	se
IN0	0.00027760	se
ND0	1	
TD	200	
SFO1	400.1738	MH
FIDRES	18.011528	Hz
SW	9.002	pp
FnMODE	TPPI	
SI	1024	
SF	400.1699986	MH
WDW	QSINE	
SSB	2	
LB	0.00	Hz
GB	0	
PC	1.00	
SI	1024	
MC2	TPPI	
SF	400.1699975	MH
WDW	QSINE	
SSB	2	
LB	0.00	Hz
GB	0	

Confidential, for research only not for regulatory filing

Confidential. For research only, Not for regulatory filing.

[redacted]

Compound ID: GK-D6

EW13860-48-P1A DMSO Bruker_D_400MHz

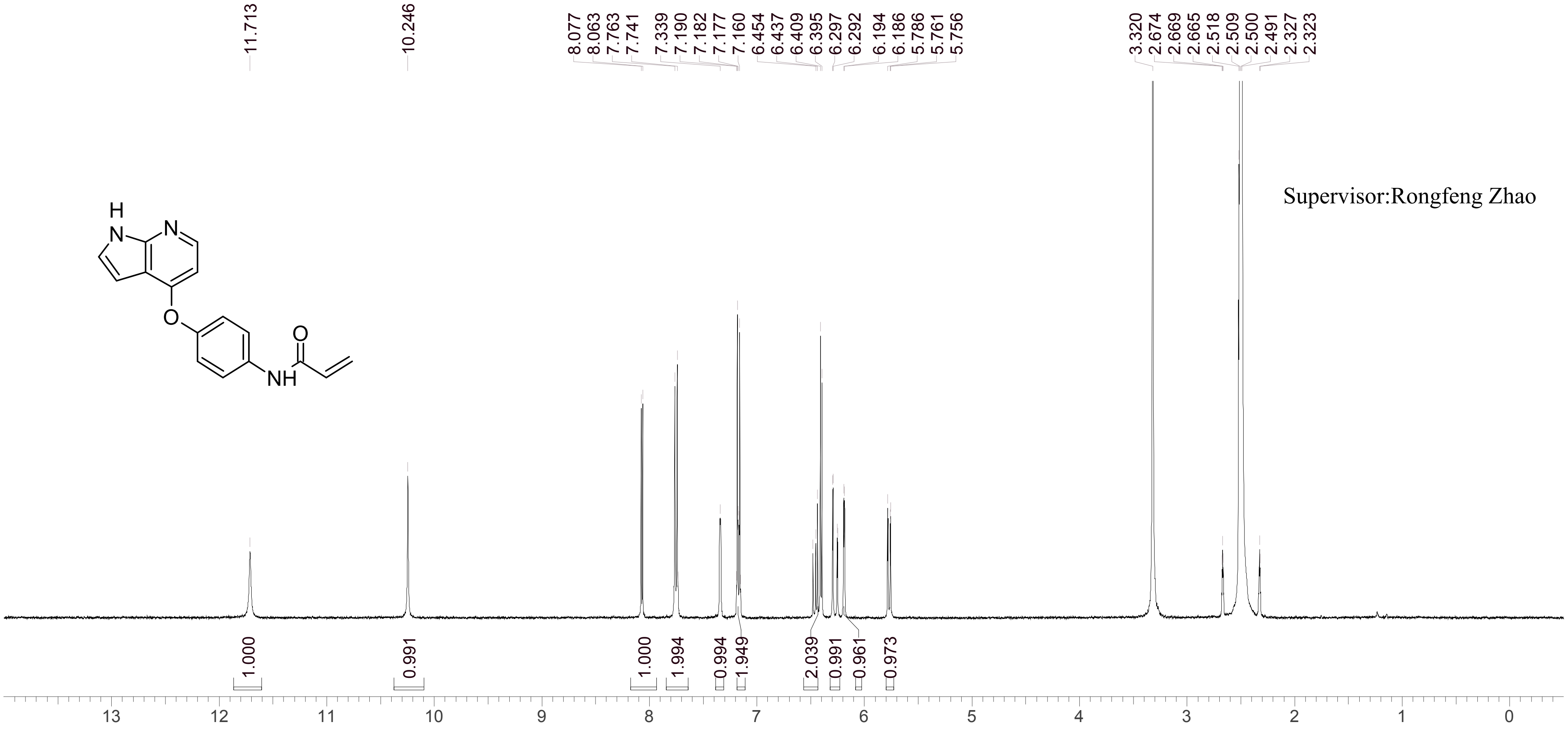

Operator:

Date:

[redacted]

Confidential, for research only not for regulatory filing

LCMS REPORT

Compound ID : GK-D6
Sample ID : EW13860-48-P1G
Injection Vol : 4ul
Location : vial62
Acq Method : d:\method\5-95CD_R_220&254_POS.lcm
Org DataFile : D:\DATA\1809\180914\EW13860-48-P1G.lcd
Injection Date : 9/14/2018 9:57:45 AM
Instrument :LCMS-W 17-102

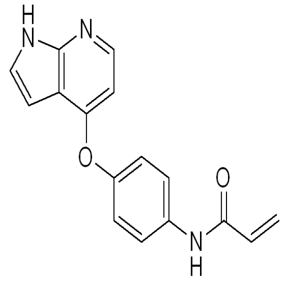

Exact Mass: 279.10

Chromatogram

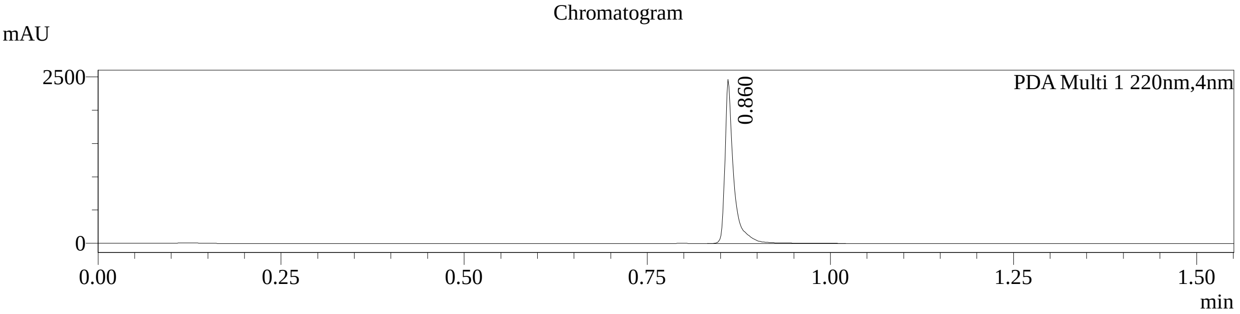

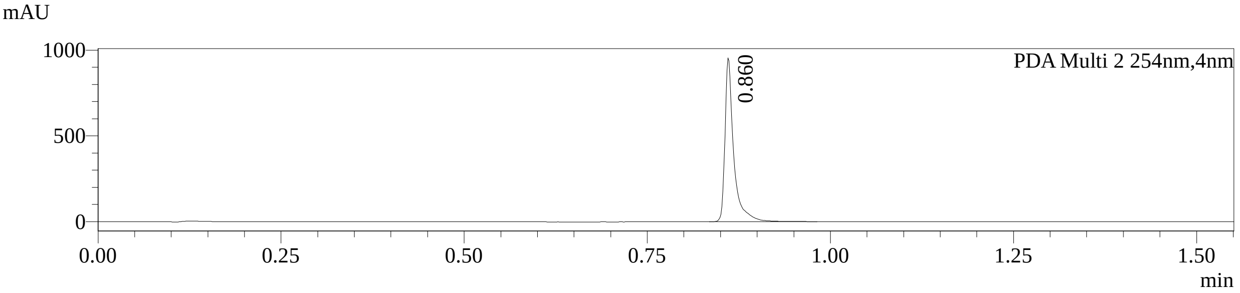

1 PDA Multi 1 / 220nm,4nm
2 PDA Multi 2 / 254nm,4nm

MS Chromatogram

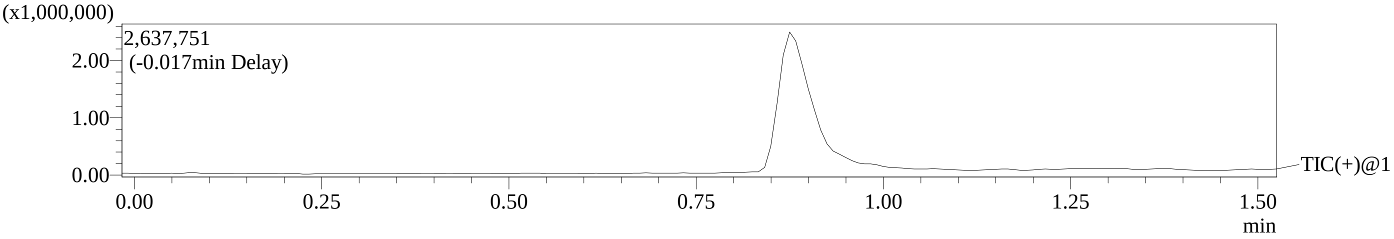

Integration Result

Peak Table

PDA Ch1 220nm

Peak#	Ret. Time	Height	Height%	USP Width	Area	Area%
1	0.860	2462819	100.000	0.022	2044073	100.000

Peak Table

PDA Ch2 254nm

Peak#	Ret. Time	Height	Height%	USP Width	Area	Area%
1	0.860	955200	100.000	0.022	797770	100.000

Operator:_______________

Date:___________________

Confidential, for research only not for regulatory filing

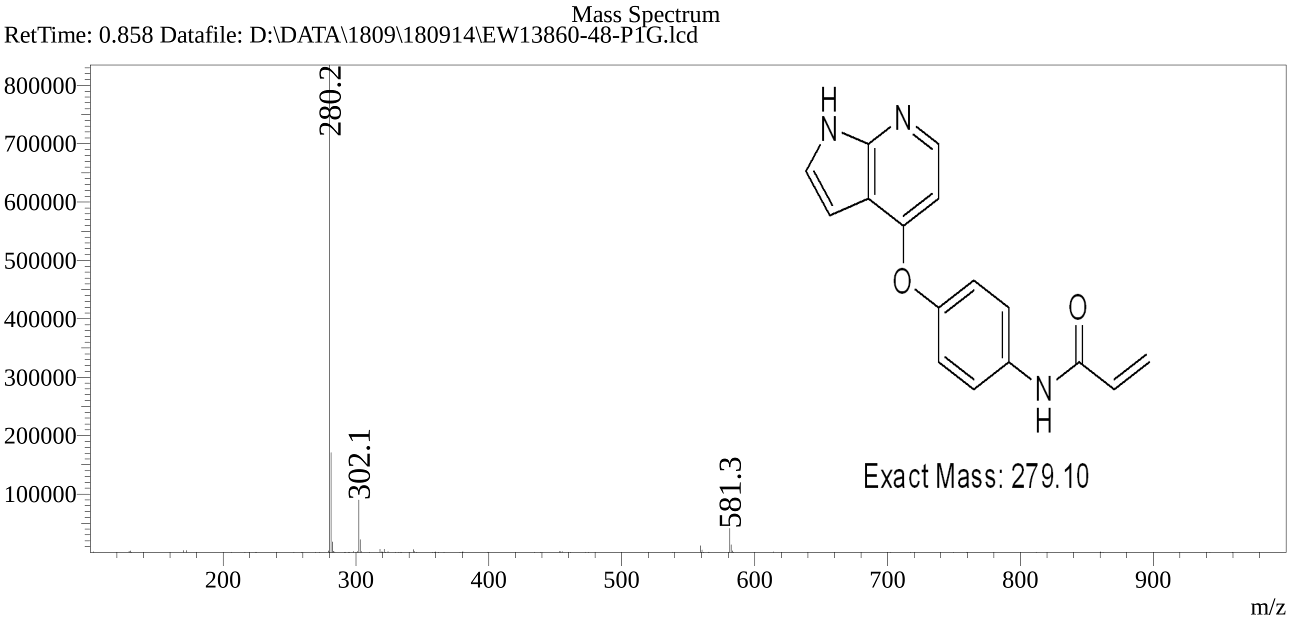

Confidential, for research only not for regulatory filing

HPLC REPORT

Compound ID : GK-D6
Sample ID : EW13860-48-P1C
Vial# : 30
Injection Volumn : 2
Filename : D:\DATA\2018\1809\180914\EW13860-48-P1C.lcd
Method Name : D:\Method\10-80CD_4min.lcm
Instrument : HPLC-J
Run time : 9/14/2018 10:58:03

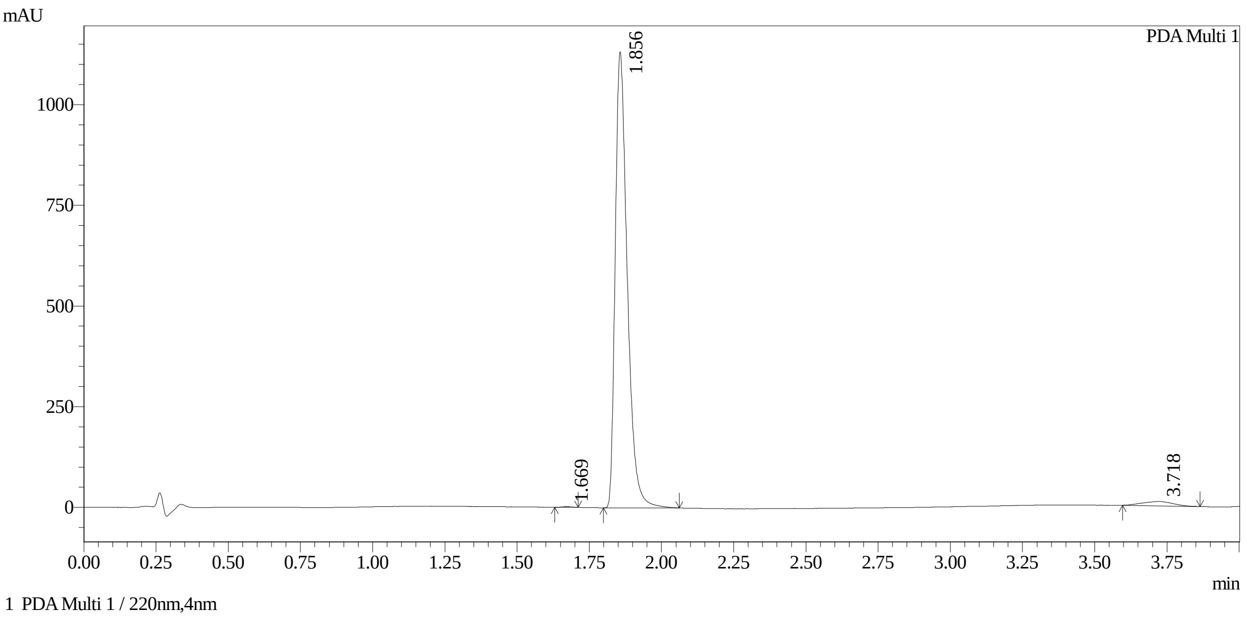

1 PDA Multi 1 / 220nm,4nm

Integration result

PeakTable

PDA Ch1 220nm

Peak#	Ret. Time	USP Width	Resolution	Height	Area	Area %
1	1.669	0.065	0.000	1717	4144	0.123
2	1.856	0.076	2.673	1132252	3279379	97.438
3	3.718	0.206	13.244	10933	82092	2.439
Total				1144902	3365614	100.000

Operater : ________________

Date : ________________

PDF created with pdfFactory Pro trial version www.pdffactory.com

Confidential, for research only not for regulatory filing

HPLC REPORT

Compound ID : GK-D6
Sample ID : EW13860-48-P1C
Filename : D:\DATA\2018\1809\180914\EW13860-48-P1C.lcd
Method Name : D:\Method\10-80CD_4min.lcm
Instrument &Column : HPLC-J
Run time : 9/14/2018 10:58:03

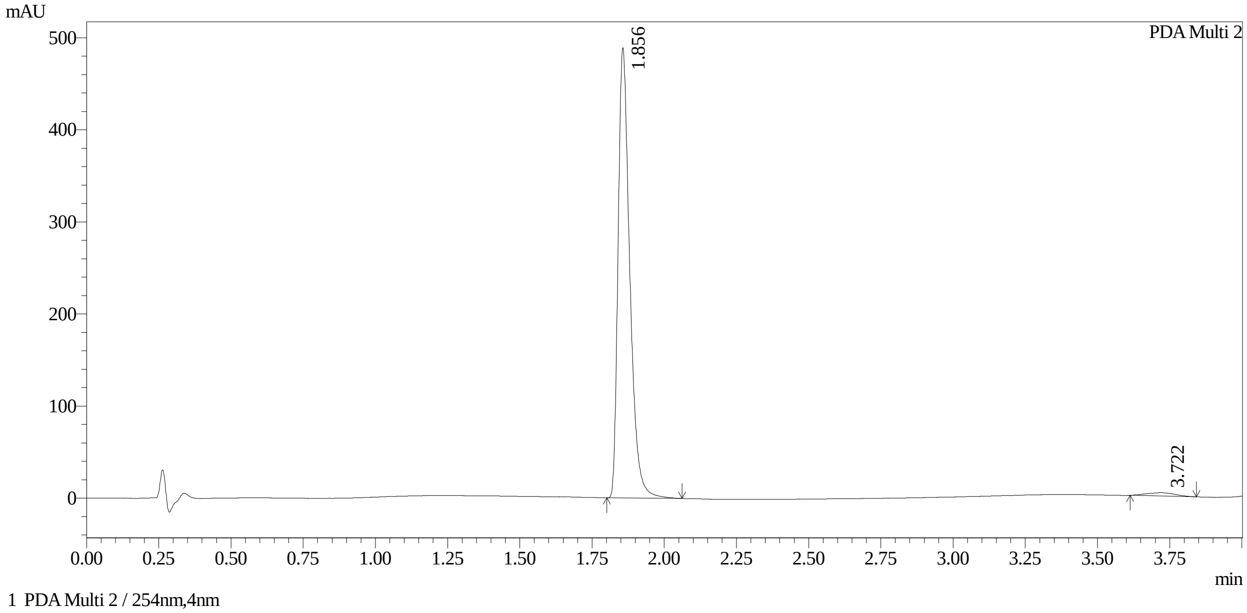

1 PDA Multi 2 / 254nm,4nm

Integration result

PeakTable

PDA Ch2 254nm

Peak#	Ret. Time	USP Width	Resolution	Height	Area	Area %
1	1.856	0.073	0.000	488951	1377702	98.261
2	3.722	0.187	14.328	3622	24378	1.739
Total				492573	1402080	100.000

Operater : ________________

Date : ________________

PDF created with pdfFactory Pro trial version www.pdffactory.com

Confidential, for research only not for regulatory filing

HPLC REPORT

Compound ID : GK-D6
Sample ID : EW13860-48-P1C
Filename : D:\DATA\2018\1809\180914\EW13860-48-P1C.lcd
Method Name : D:\Method\10-80CD_4min.lcm
Instrument &Column : HPLC-J
Run time : 9/14/2018 10:58:03

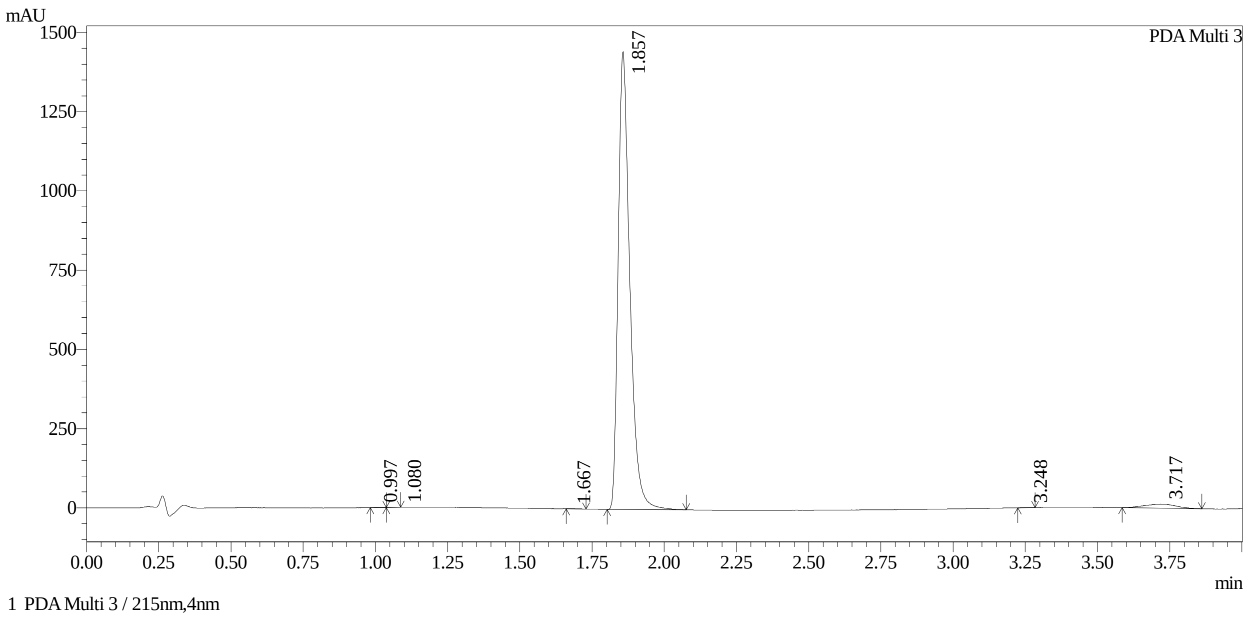

1 PDA Multi 3 / 215nm,4nm

Integration result

PeakTable

PDA Ch3 215nm

Peak#	Ret. Time	USP Width	Resolution	Height	Area	Area %
1	0.997	0.063	0.000	606	1532	0.037
2	1.080	0.084	1.132	620	1441	0.035
3	1.667	0.067	7.789	1675	3526	0.085
4	1.857	0.074	2.690	1444189	4046426	97.517
5	3.248	0.047	23.025	431	1143	0.028
6	3.717	0.203	3.745	12546	95397	2.299
Total				1460066	4149465	100.000

Operater : ________________

Date : ________________

PDF created with pdfFactory Pro trial version www.pdffactory.com

Confidential. For research only, Not for regulatory filing.

[redacted]

Compound ID: GK-D7

EW17394-6-P1B DMSO Bruker_H_400MHz

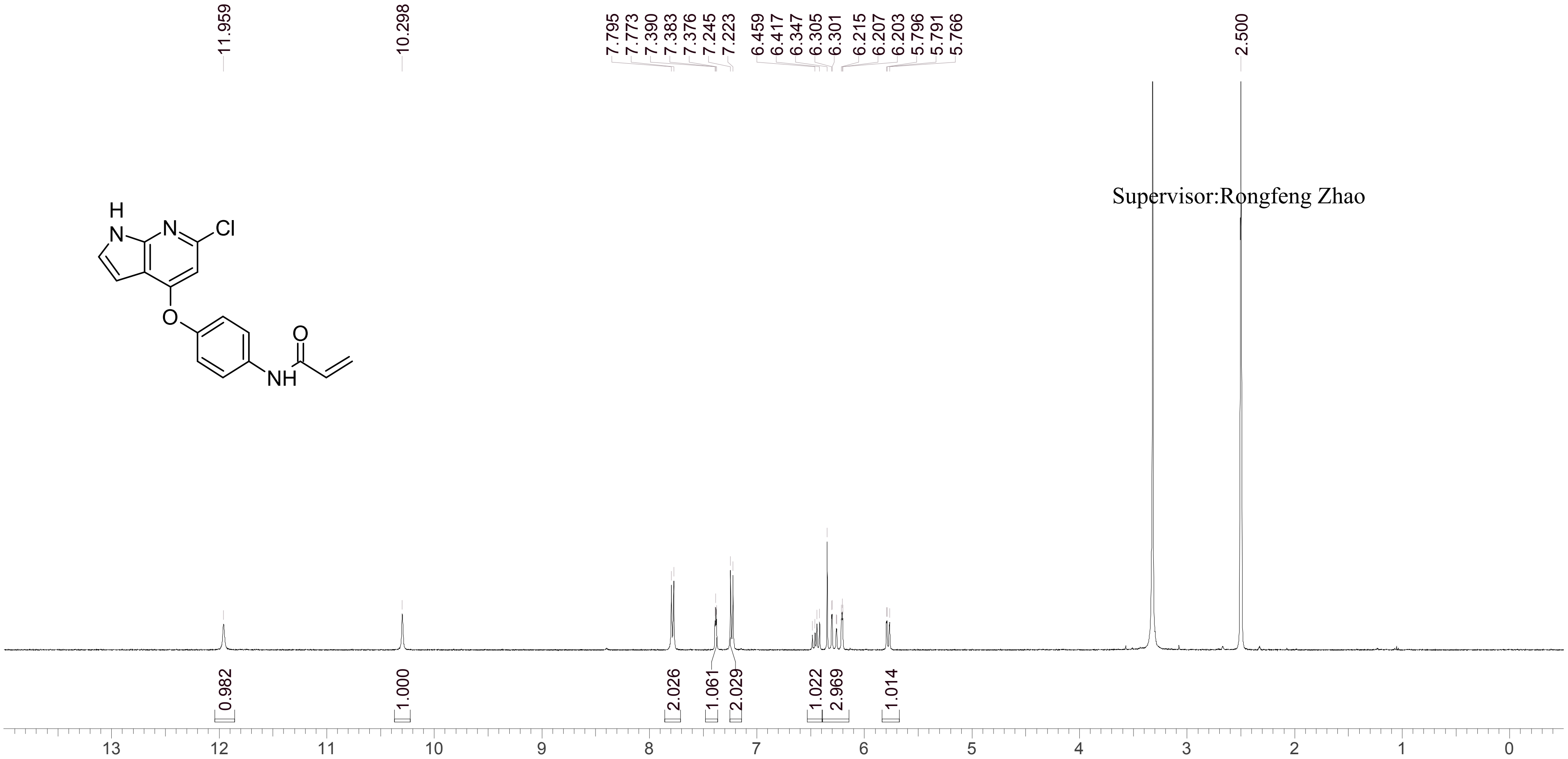

Supervisor:Rongfeng Zhao

Operator: Date:

If y[redacted]lease click below link to download

Confidential. For research information only

LCMS REPORT

Compound ID : GK-D7
Sample ID : EW17394-6-P1B
Injection Vol : 2ul
Location : vial33
Acq Method : d:\method\5-95AB_4min_220&254.lcm
Org DataFile : D:\DATA\1903\190319\EW17394-6-P1B.lcd
Injection Date : 2019-03-19 20:30:12
Instrument : LCMS-I

Chromatogram

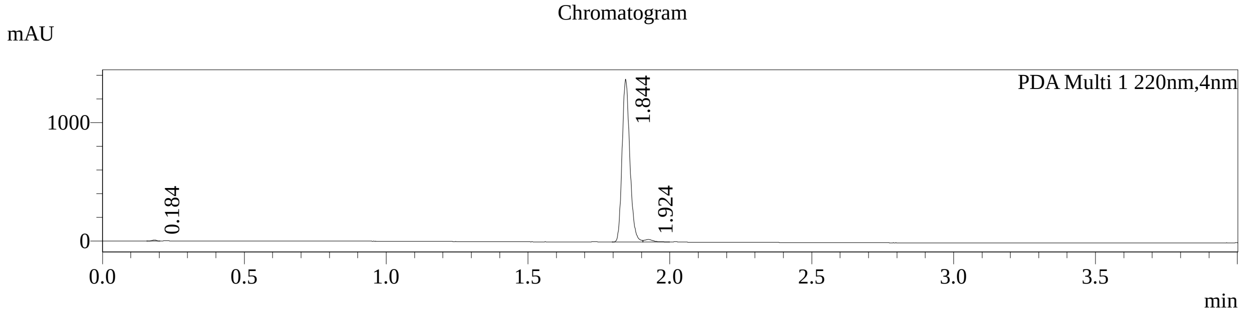

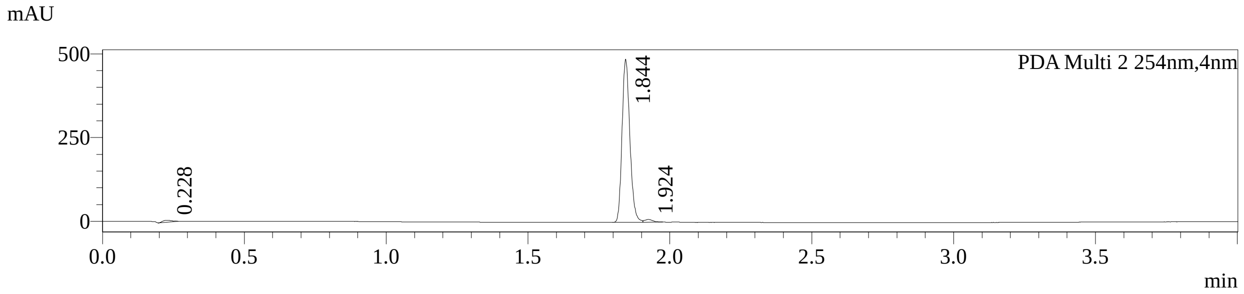

1 PDA Multi 1 / 220nm,4nm
2 PDA Multi 2 / 254nm,4nm

MS Chromatogram

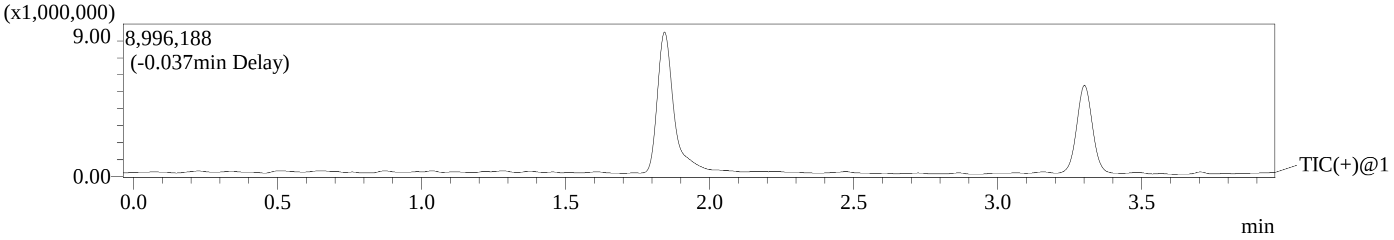

Integration Result

Peak Table

PDA Ch1 220nm

Peak#	Ret. Time	Height	Height%	USP Width	Area	Area%
1	0.184	9699	0.690	0.032	10678	0.403
2	1.844	1375569	97.835	0.051	2597828	97.933
3	1.924	20735	1.475	0.070	44150	1.664

Peak Table

PDA Ch2 254nm

Peak#	Ret. Time	Height	Height%	USP Width	Area	Area%
1	0.228	5550	1.109	0.061	12693	1.323
2	1.844	487147	97.318	0.051	930916	97.064
3	1.924	7877	1.574	0.066	15464	1.612

Confidential. For research information only

Operator:_______________

Date:___________________

Confidential. For research information only

Mass Spectrum

RetTime: 1.843 DateFile: D:\DATA\1903\190319\EW17394-6-P1B.lcd

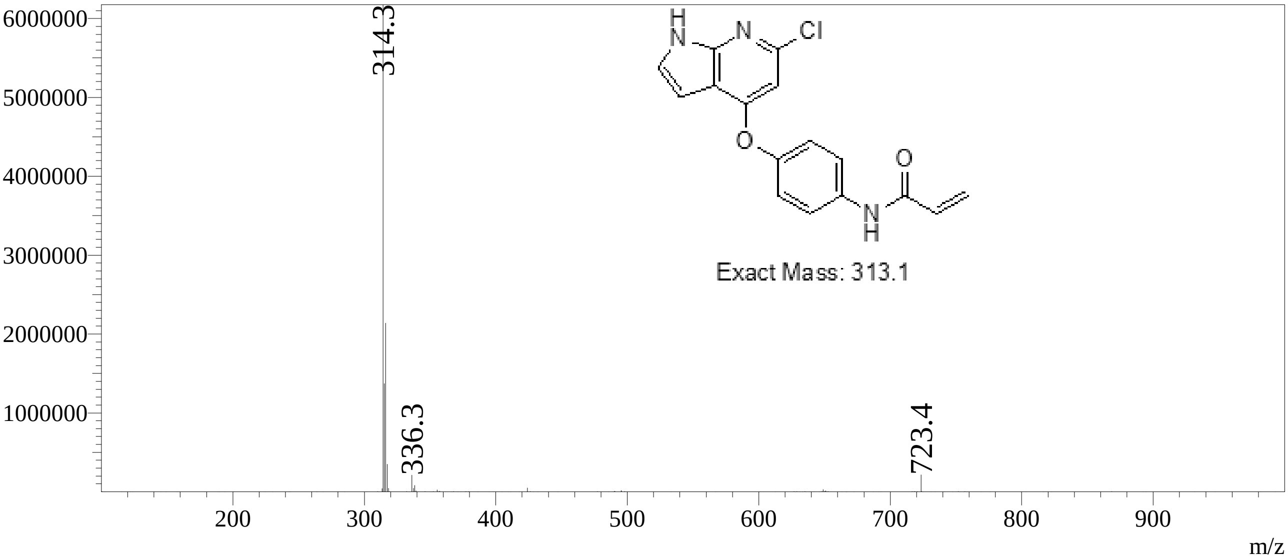

RetTime: 1.923 DateFile: D:\DATA\1903\190319\EW17394-6-P1B.lcd

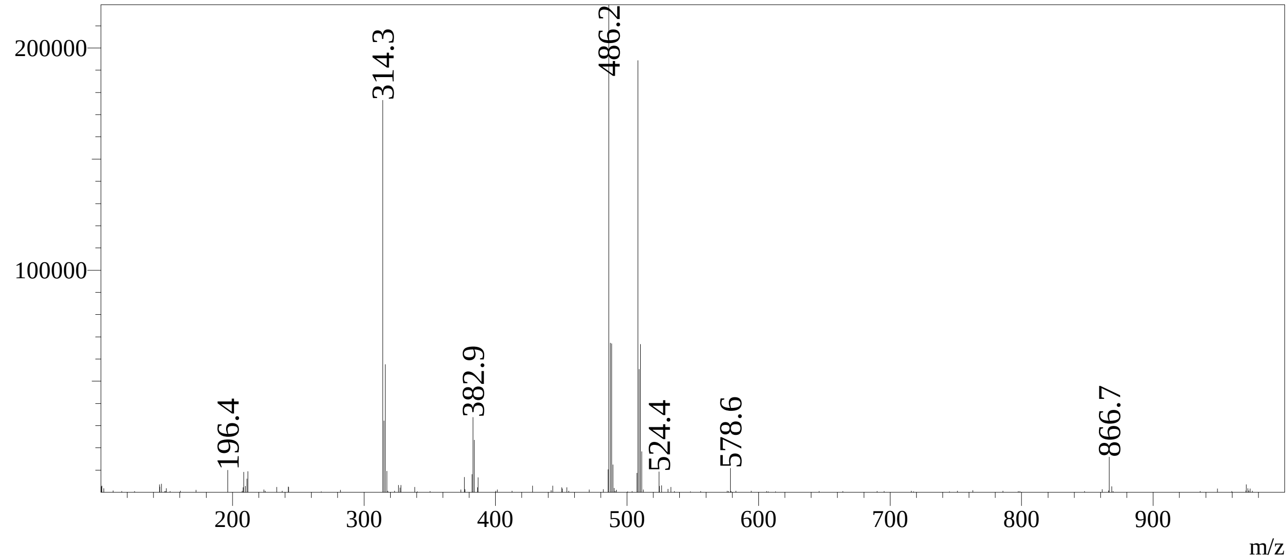

Confidential. For research only, Not for regulatory filing.

[redacted]

Compound ID: GK-D8

EW17394-7-P1C DMSO Bruker_H_400MHz

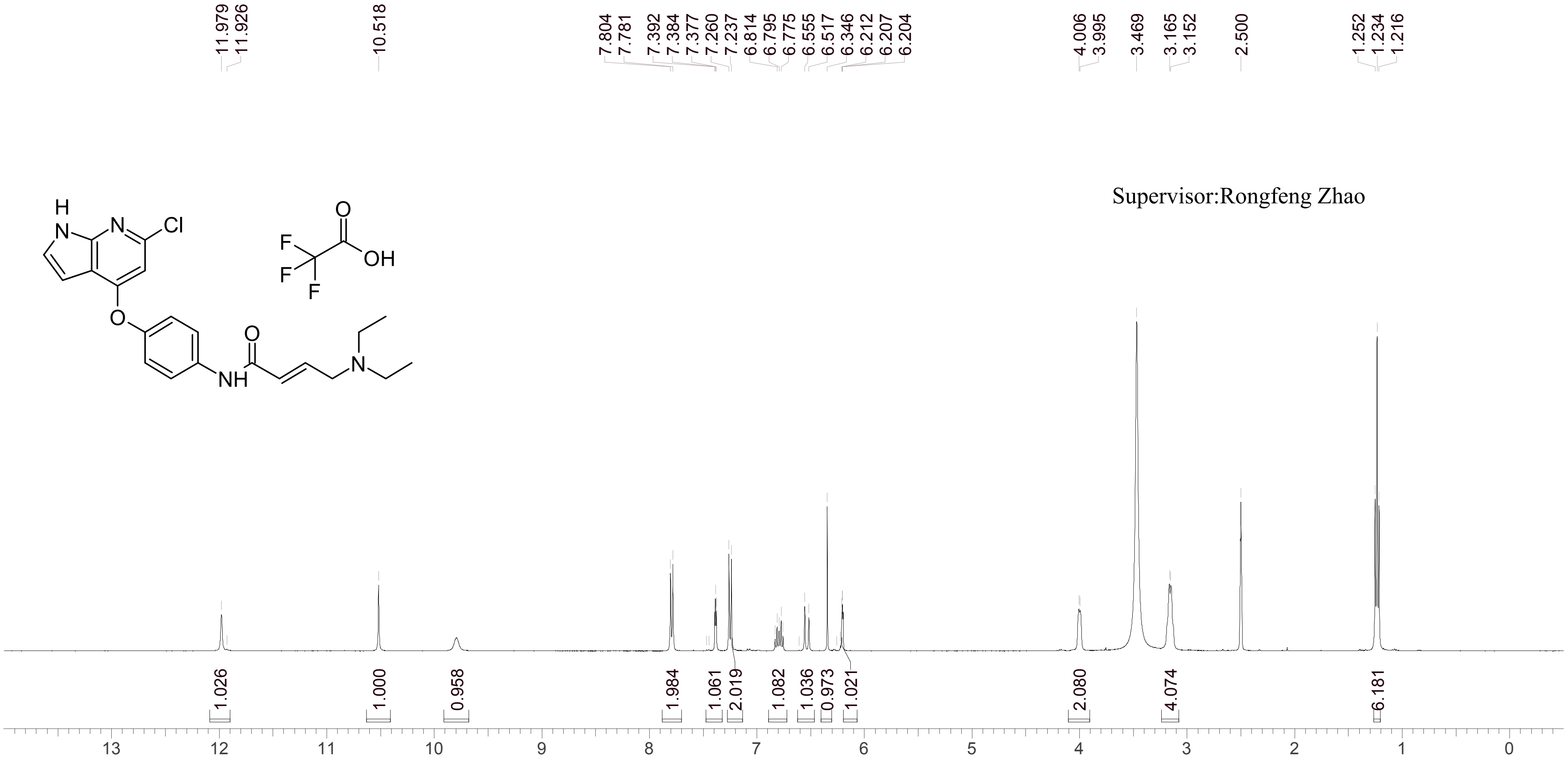

Operator: Date:

[redacted]

Confidential. For research information only

LCMS REPORT

Compound ID : GK-D8
Sample ID : EW17394-7-P1C
Injection Vol : 0.5ul
Location : vial35
Acq Method : d:\method\5-95AB_4min_220&254.lcm
Org DataFile : D:\DATA\1903\190319\EW17394-7-P1C.lcd
Injection Date : 2019-03-19 20:55:22
Instrument : LCMS-I

Chromatogram

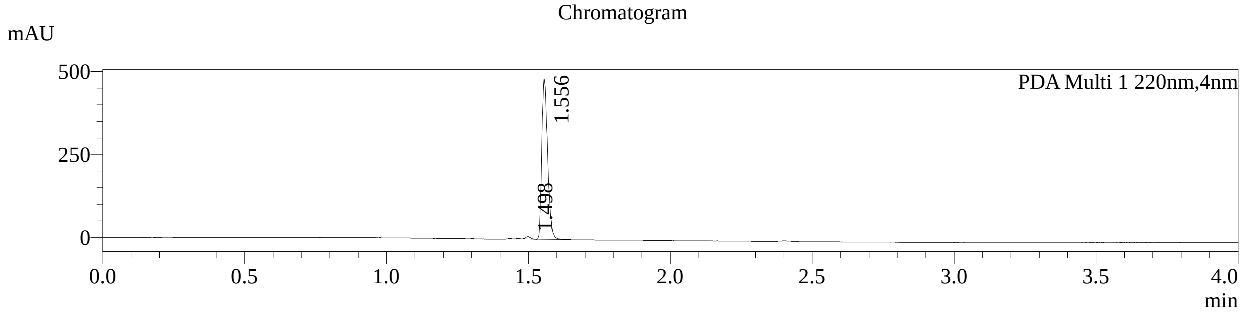

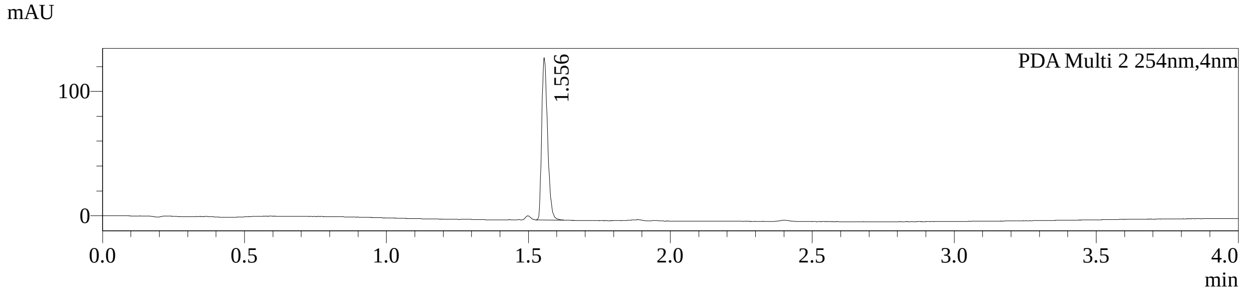

1 PDA Multi 1 / 220nm,4nm
2 PDA Multi 2 / 254nm,4nm

MS Chromatogram

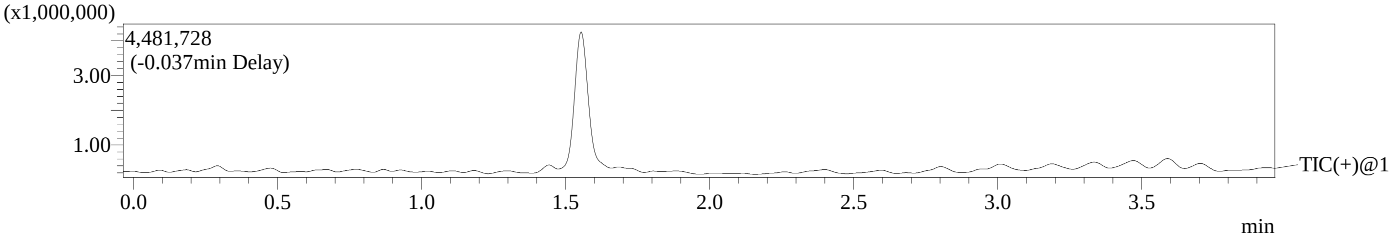

Integration Result

Peak Table

PDA Ch1 220nm

Peak#	Ret. Time	Height	Height%	USP Width	Area	Area%
1	1.498	6973	1.420	0.034	8261	1.177
2	1.556	484123	98.580	0.039	693731	98.823

Peak Table

PDA Ch2 254nm

Peak#	Ret. Time	Height	Height%	USP Width	Area	Area%
1	1.556	130342	100.000	0.039	186824	100.000

Confidential. For research information only

Operator:_______________

Date:___________________

Confidential. For research information only

Mass Spectrum

RetTime: 1.498 DateFile: D:\DATA\1903\190319\EW17394-7-P1C.lcd

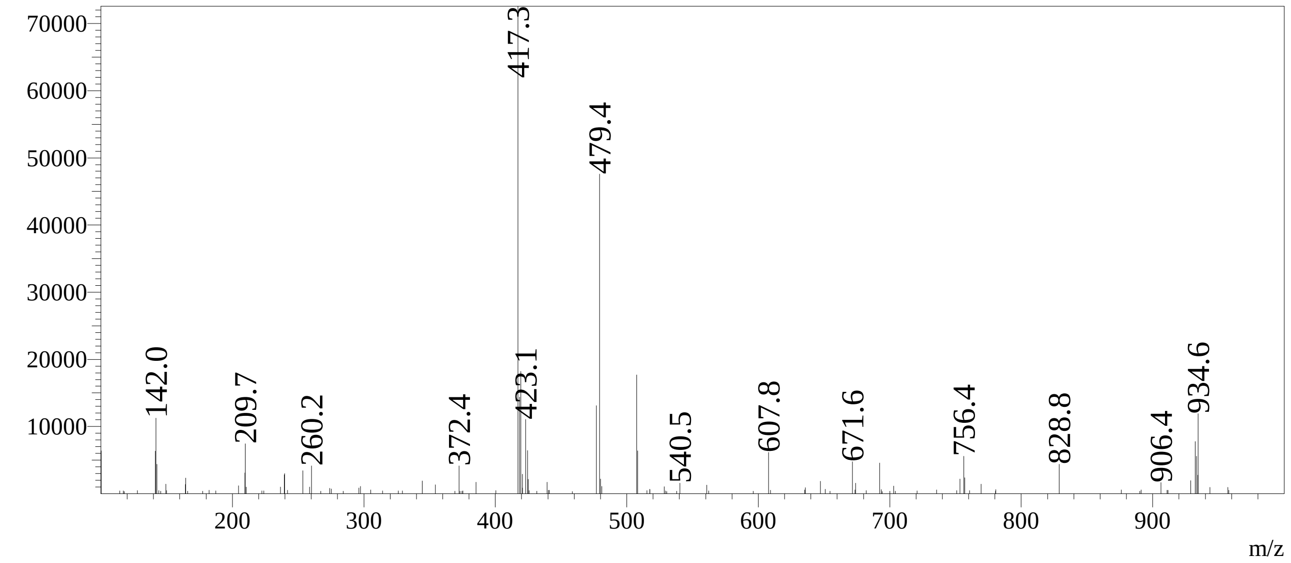

RetTime: 1.555 DateFile: D:\DATA\1903\190319\EW17394-7-P1C.lcd

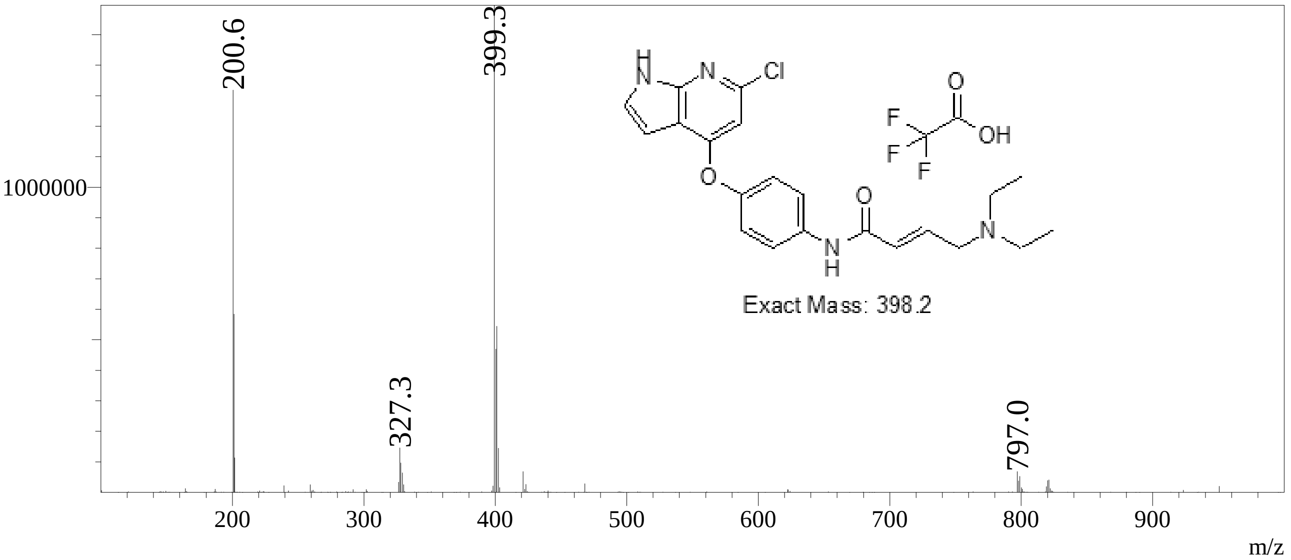

Confidential. For research only, Not for regulatory filing.

[redacted]

Compound ID: GK-D9

EW17394-26-P1A DMSO Bruker_B_400MHz

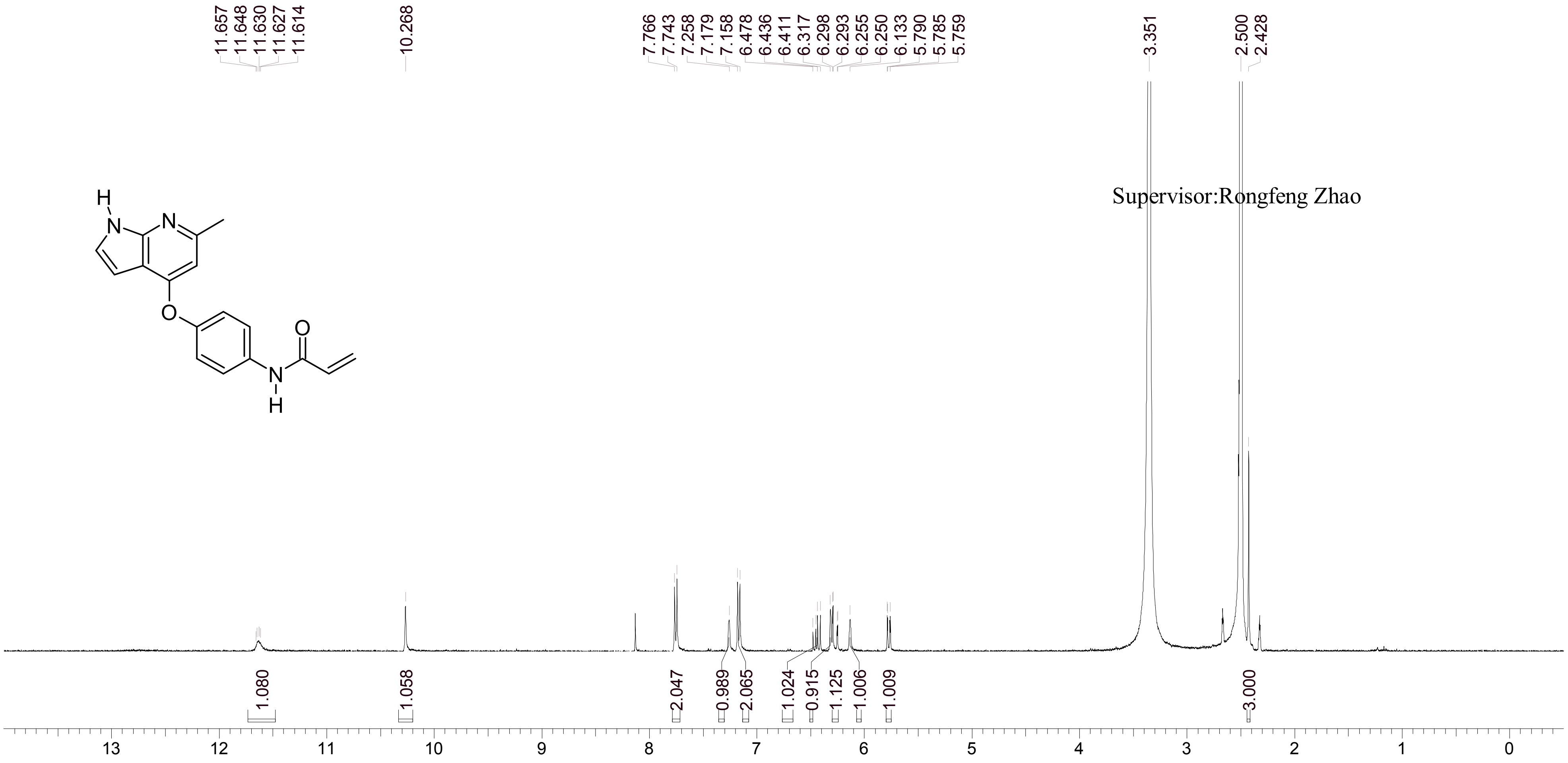

Supervisor:Rongfeng Zhao

Date:

[redacted]

Confidential. For research information only

LCMS REPORT

Compound ID : GK-D9
Sample ID : EW17394-26-P1A2
Injection Vol : 0.2ul
Location : vial8
Acq Method : d:\method\5-95AB_4min_220&254.lcm
Org DataFile : D:\DATA\1904\190403\EW17394-26-P1A2.lcd
Injection Date : 4/3/2019 7:12:48 PM
Instrument : LCMS-I

Chromatogram

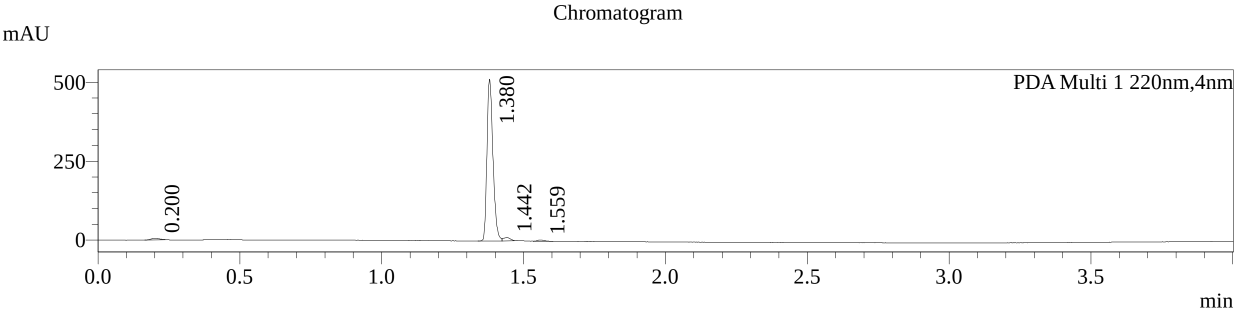

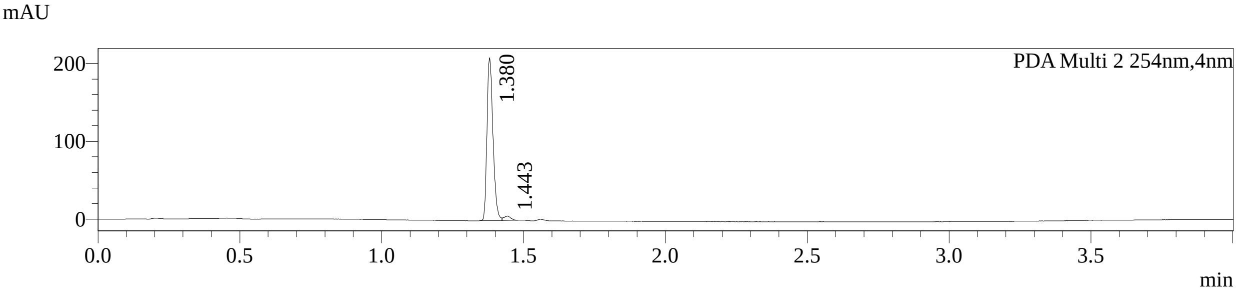

1 PDA Multi 1 / 220nm,4nm
2 PDA Multi 2 / 254nm,4nm

MS Chromatogram

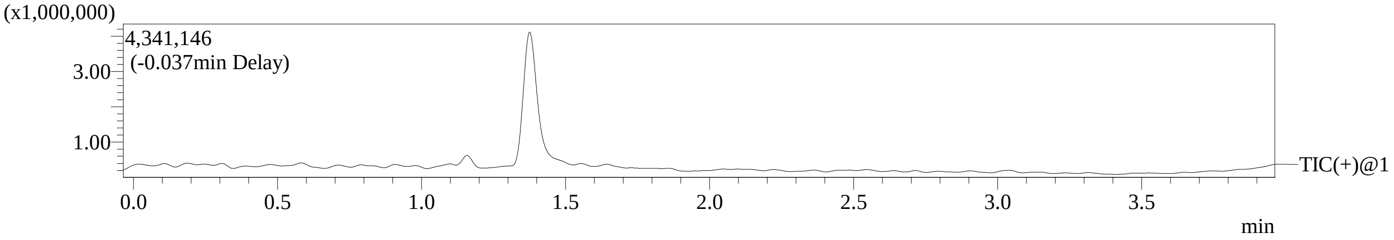

Integration Result

Peak Table

PDA Ch1 220nm

Peak#	Ret. Time	Height	Height%	USP Width	Area	Area%
1	0.200	4942	0.929	0.068	11887	1.501
2	1.380	512379	96.341	0.040	754588	95.292
3	1.442	10378	1.951	0.078	18578	2.346
4	1.559	4141	0.779	0.046	6817	0.861

Peak Table

PDA Ch2 254nm

Peak#	Ret. Time	Height	Height%	USP Width	Area	Area%
1	1.380	209188	97.487	0.040	308592	97.068
2	1.443	5392	2.513	0.059	9321	2.932

Confidential. For research information only

Operator:_______________

Date:___________________

Confidential. For research information only

Mass Spectrum

RetTime: 0.200 DateFile: D:\DATA\1904\190403\EW17394-26-P1A2.lcd

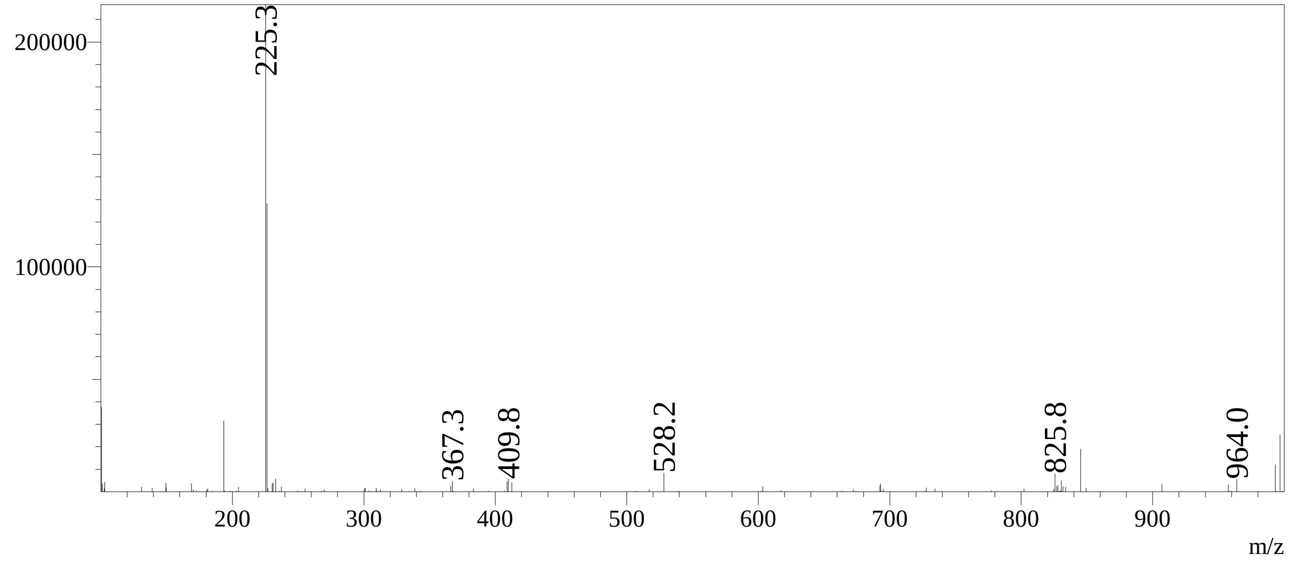

RetTime: 1.380 DateFile: D:\DATA\1904\190403\EW17394-26-P1A2.lcd

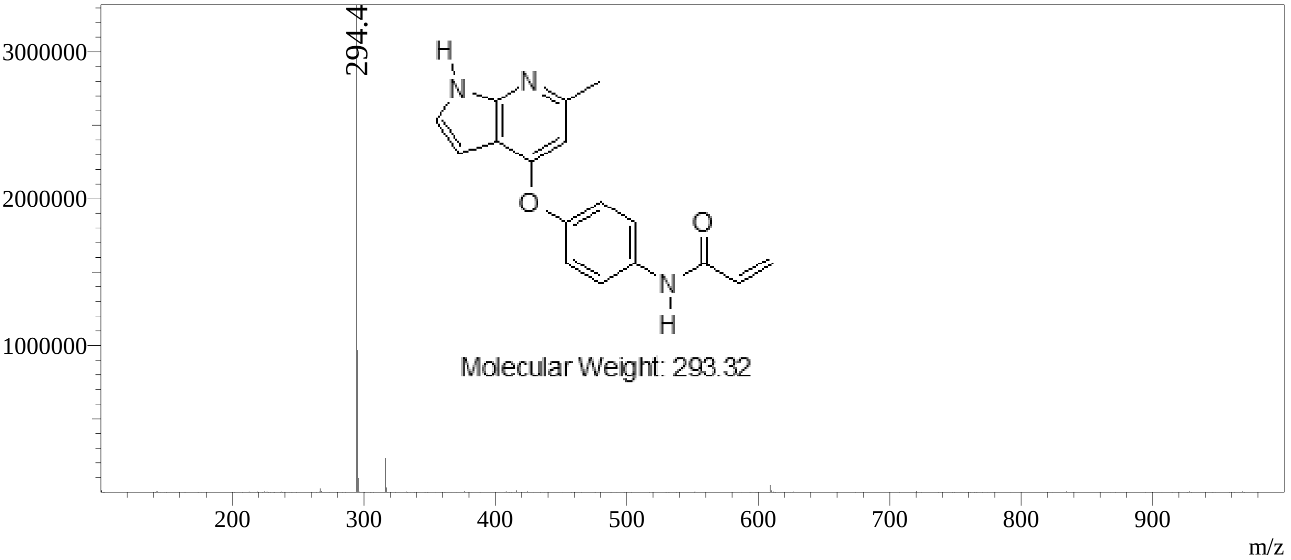

RetTime: 1.442 DateFile: D:\DATA\1904\190403\EW17394-26-P1A2.lcd

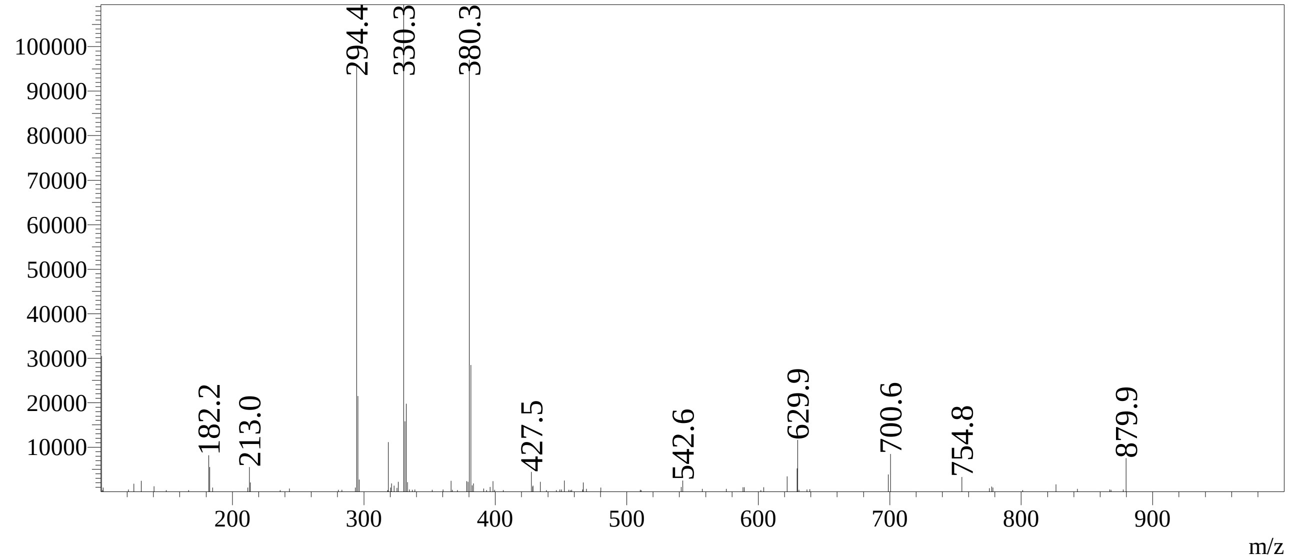

Confidential. For research only, Not for regulatory filing.

[redacted]

Compound ID: GK-D10

EW17394-27-P1A DMSO Bruker_F_400MHz

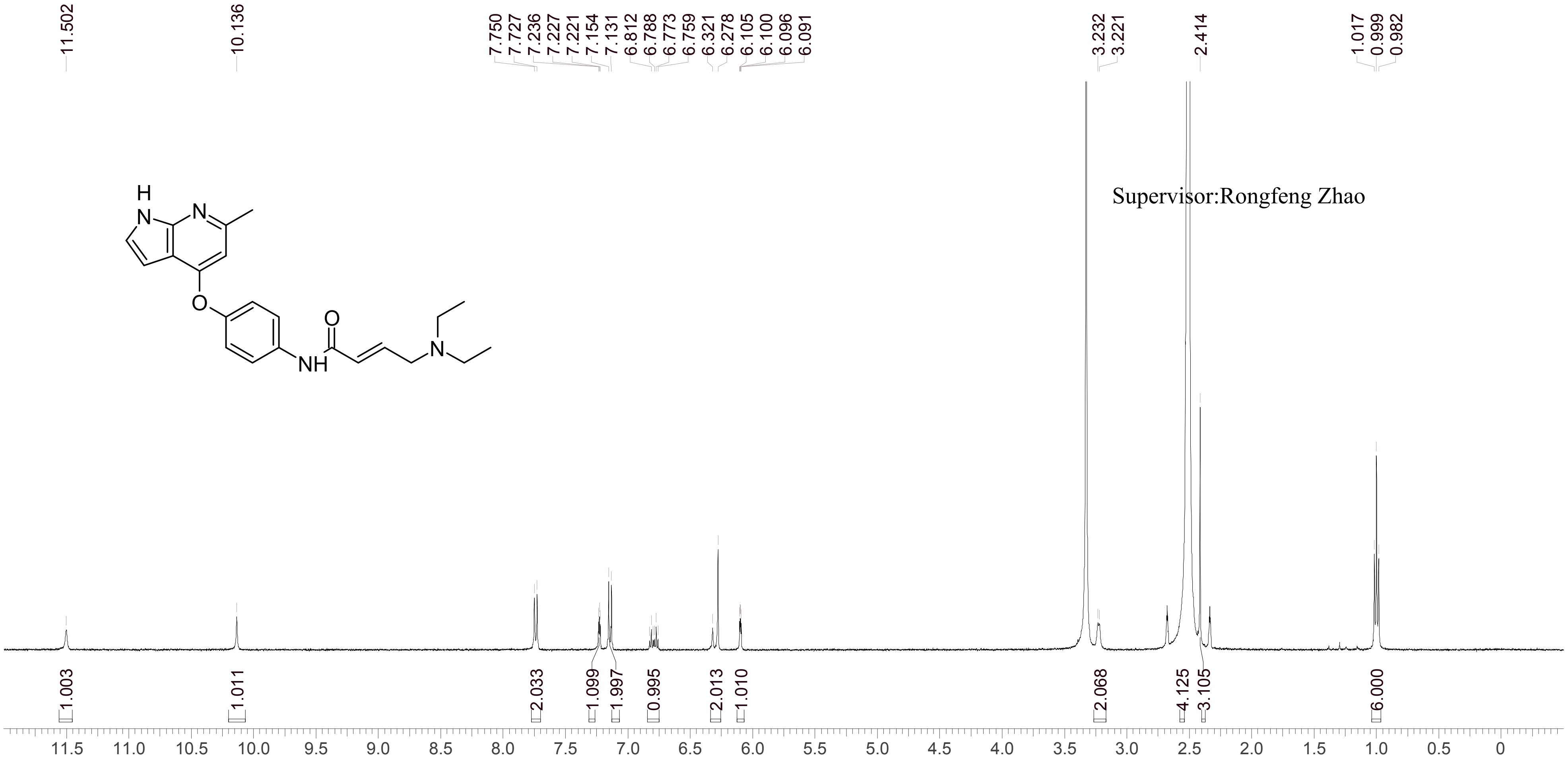

If y [redacted]

Confidential. For research information only

LCMS REPORT

Compound ID : GK-D10
Sample ID : EW17394-27-P1F1
Injection Vol : 1ul
Location : vial29
Acq Method : d:\method\5-95AB_R_220&254.lcm
Org DataFile : D:\DATA\1904\190404\EW17394-27-P1F1.lcd
Injection Date : 4/4/2019 12:17:34 PM
Instrument : LCMS-I

Chromatogram

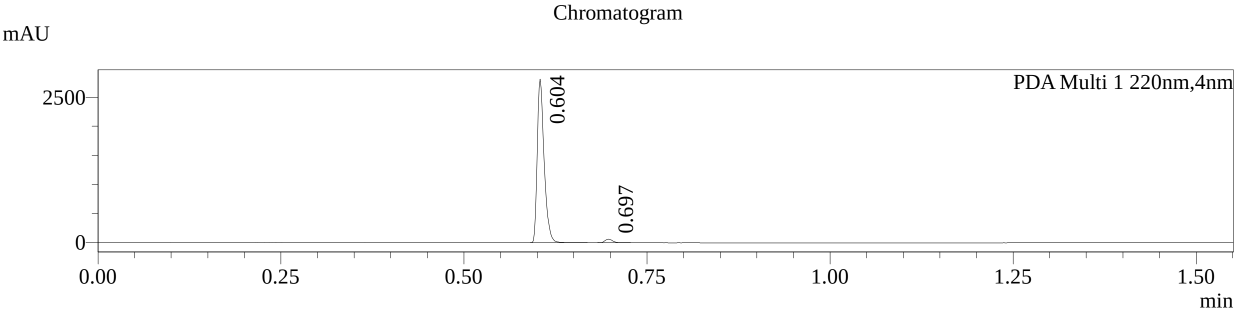

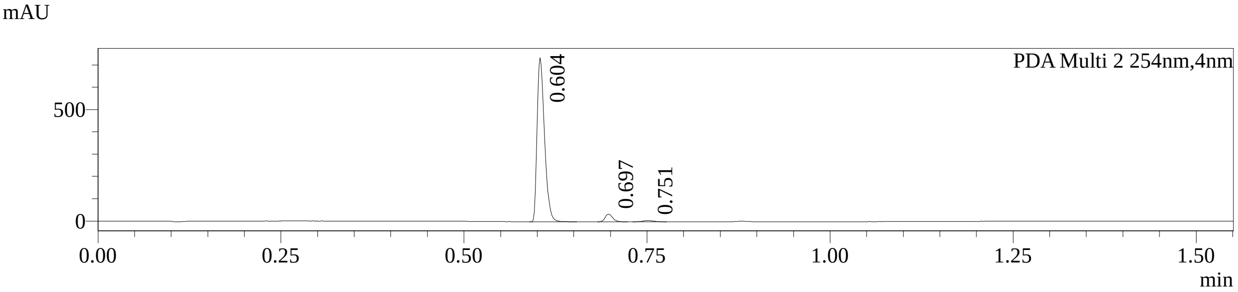

1 PDA Multi 1 / 220nm,4nm
2 PDA Multi 2 / 254nm,4nm

MS Chromatogram

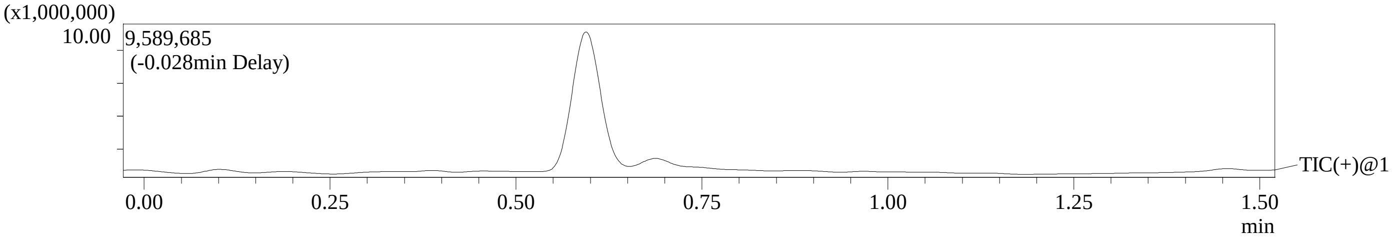

Integration Result

Peak Table

PDA Ch1 220nm

Peak#	Ret. Time	Height	Height%	USP Width	Area	Area%
1	0.604	2815408	97.850	0.020	1859966	97.739
2	0.697	61854	2.150	0.021	43033	2.261

Peak Table

PDA Ch2 254nm

Peak#	Ret. Time	Height	Height%	USP Width	Area	Area%
1	0.604	735720	94.851	0.021	524278	94.620
2	0.697	34499	4.448	0.021	24017	4.334
3	0.751	5440	0.701	0.030	5793	1.046

Operator:_______________

Date:___________________

Confidential. For research information only

Mass Spectrum

RetTime: 0.603 DateFile: D:\DATA\1904\190404\EW17394-27-P1F1.lcd

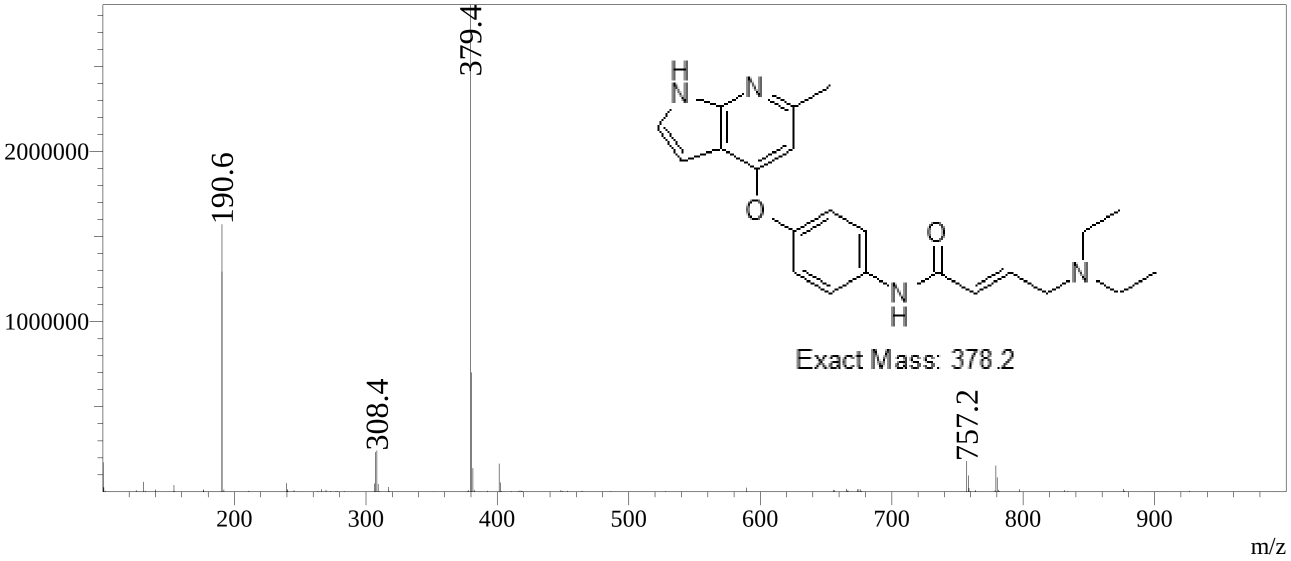

RetTime: 0.697 DateFile: D:\DATA\1904\190404\EW17394-27-P1F1.lcd

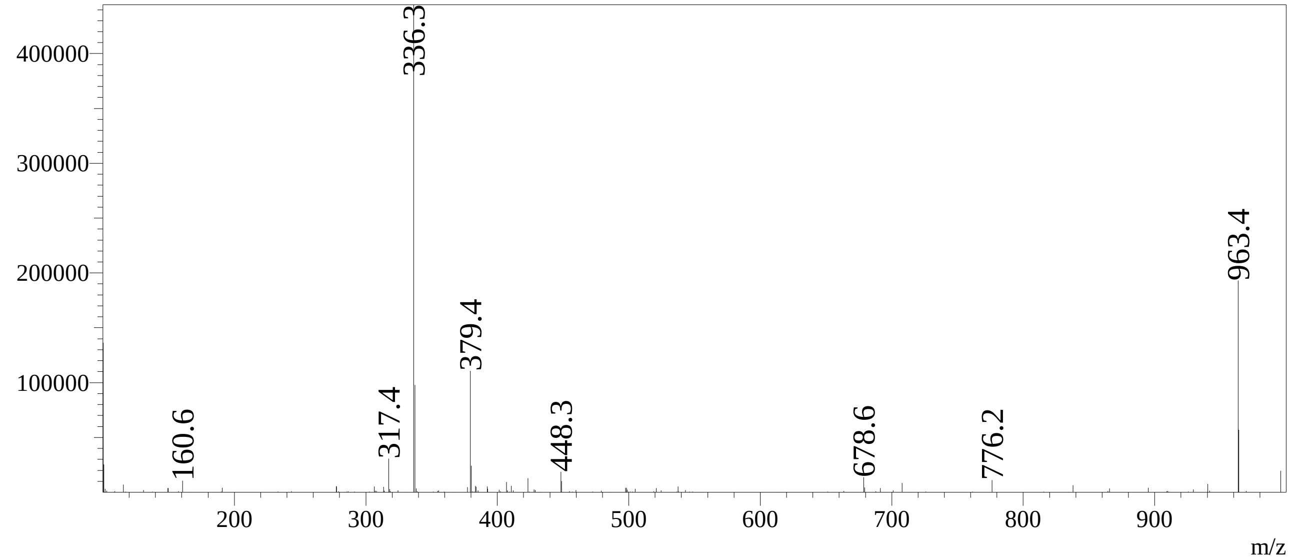

Confidential. For research information only

HPLC REPORT

Compound ID	: GK-D10
Sample ID	: EW17394-27-P1F2
Vial#	: 58
Injection Volumn	: 0.5
Filename	: D:\DATA\2019\1904\190404\EW17394-27-P1F2.lcd
Method Name	: D:\Method\10-80AB_4min.lcm
Instrument	: HPLC-Q
Run time	: 4/4/2019 2:51:53 PM

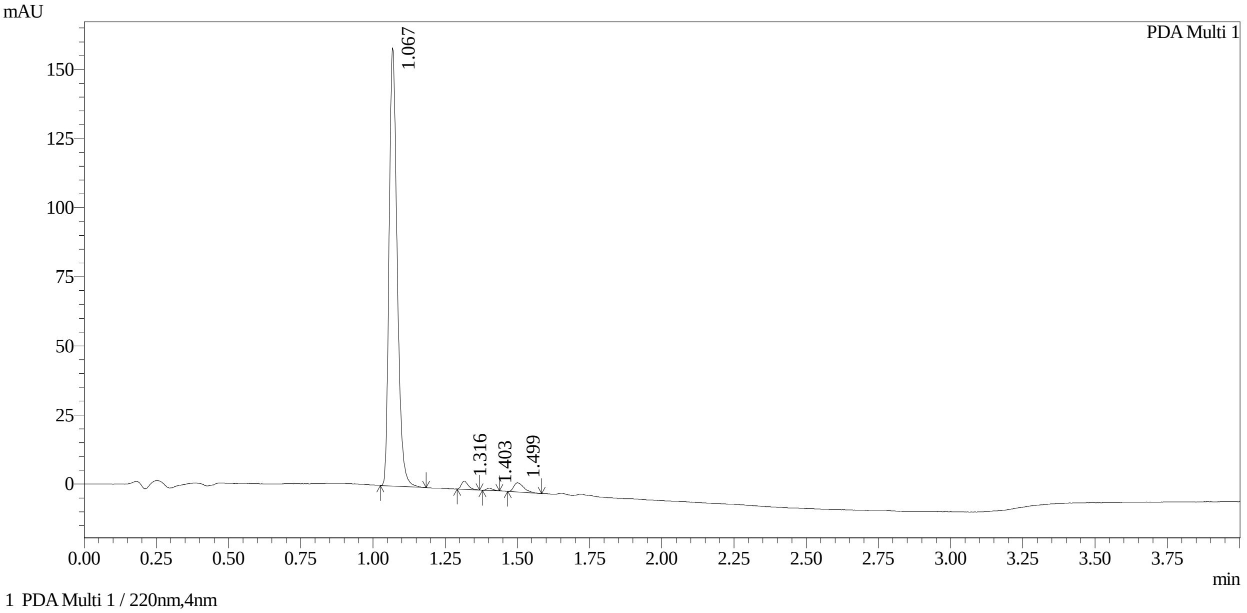

1 PDA Multi 1 / 220nm,4nm

Integration result

PeakTable

PDA Ch1 220nm

Peak#	Ret. Time	USP Width	Resolution	Height	Area	Area %
1	1.067	0.051	0.000	158562	306942	95.554
2	1.316	0.044	5.221	2966	4960	1.544
3	1.403	0.044	1.972	830	1233	0.384
4	1.499	0.063	1.802	3321	8089	2.518
Total				165678	321225	100.000

Operater : ________________

Date : ________________

Confidential. For research information only

HPLC REPORT

Compound ID : GK-D10
Sample ID : EW17394-27-P1F2
Filename : D:\DATA\2019\1904\190404\EW17394-27-P1F2.lcd
Method Name : D:\Method\10-80AB_4min.lcm
Instrument : HPLC-Q
Run time : 4/4/2019 2:51:53 PM

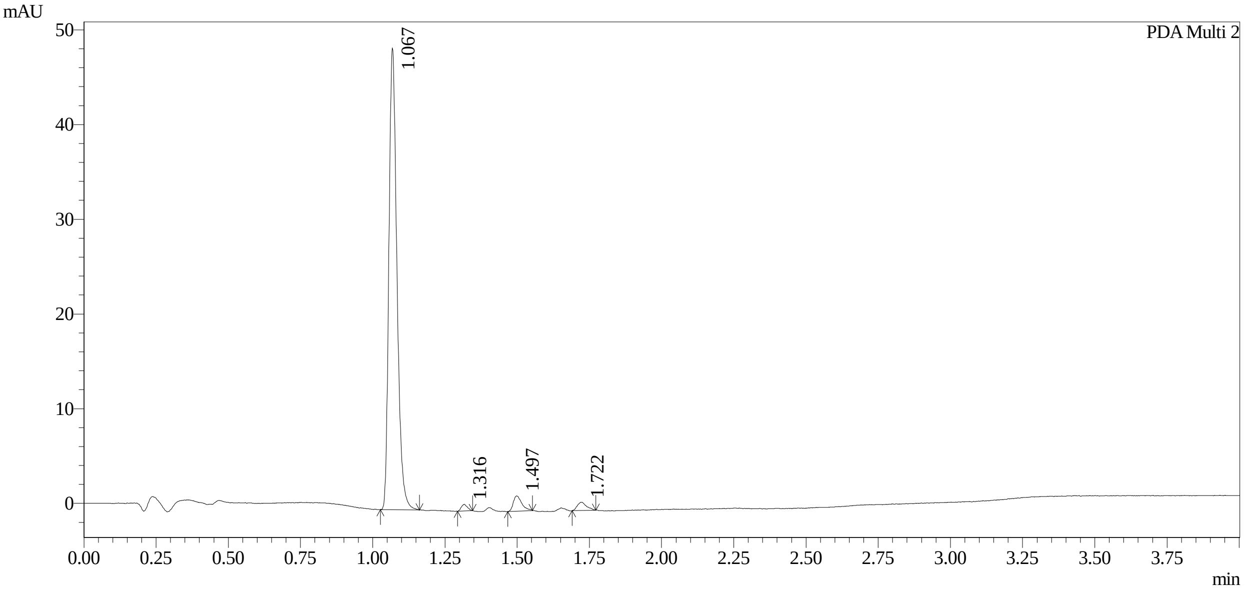

1 PDA Multi 2 / 254nm,4nm

Integration result

PeakTable

PDA Ch2 254nm

Peak#	Ret. Time	USP Width	Resolution	Height	Area	Area %
1	1.067	0.051	0.000	48755	94150	93.823
2	1.316	0.042	5.332	704	1021	1.017
3	1.497	0.054	3.775	1608	3317	3.306
4	1.722	0.057	4.061	865	1861	1.854
Total				51932	100349	100.000

Operater : ________________

Date : ________________

Confidential. For research information only

HPLC REPORT

Compound ID : GK-D10
Sample ID : EW17394-27-P1F2
Filename : D:\DATA\2019\1904\190404\EW17394-27-P1F2.lcd
Method Name : D:\Method\10-80AB_4min.lcm
Instrument &Column : HPLC-Q
Run time : 4/4/2019 2:51:53 PM

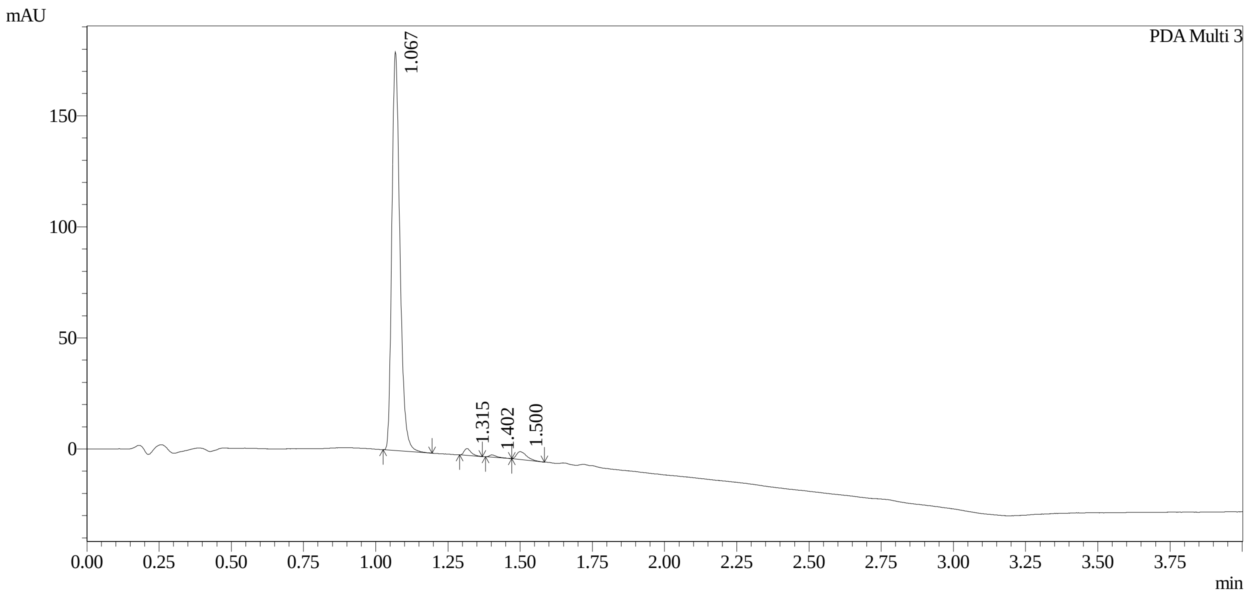

1 PDA Multi 3 / 215nm,4nm

Integration result

PeakTable

PDA Ch3 215nm

Peak#	Ret. Time	USP Width	Resolution	Height	Area	Area %
1	1.067	0.051	0.000	179506	347287	95.691
2	1.315	0.045	5.157	3063	5158	1.421
3	1.402	0.046	1.906	1026	1731	0.477
4	1.500	0.064	1.781	3563	8750	2.411
Total				187158	362926	100.000

Operater : ________________

Date : ________________

Confidential. For research only, Not for regulatory filing.

[redacted]

Compound ID: GK-PD1b

EW20854-35-P1A DMSO Bruker_E_400MHz

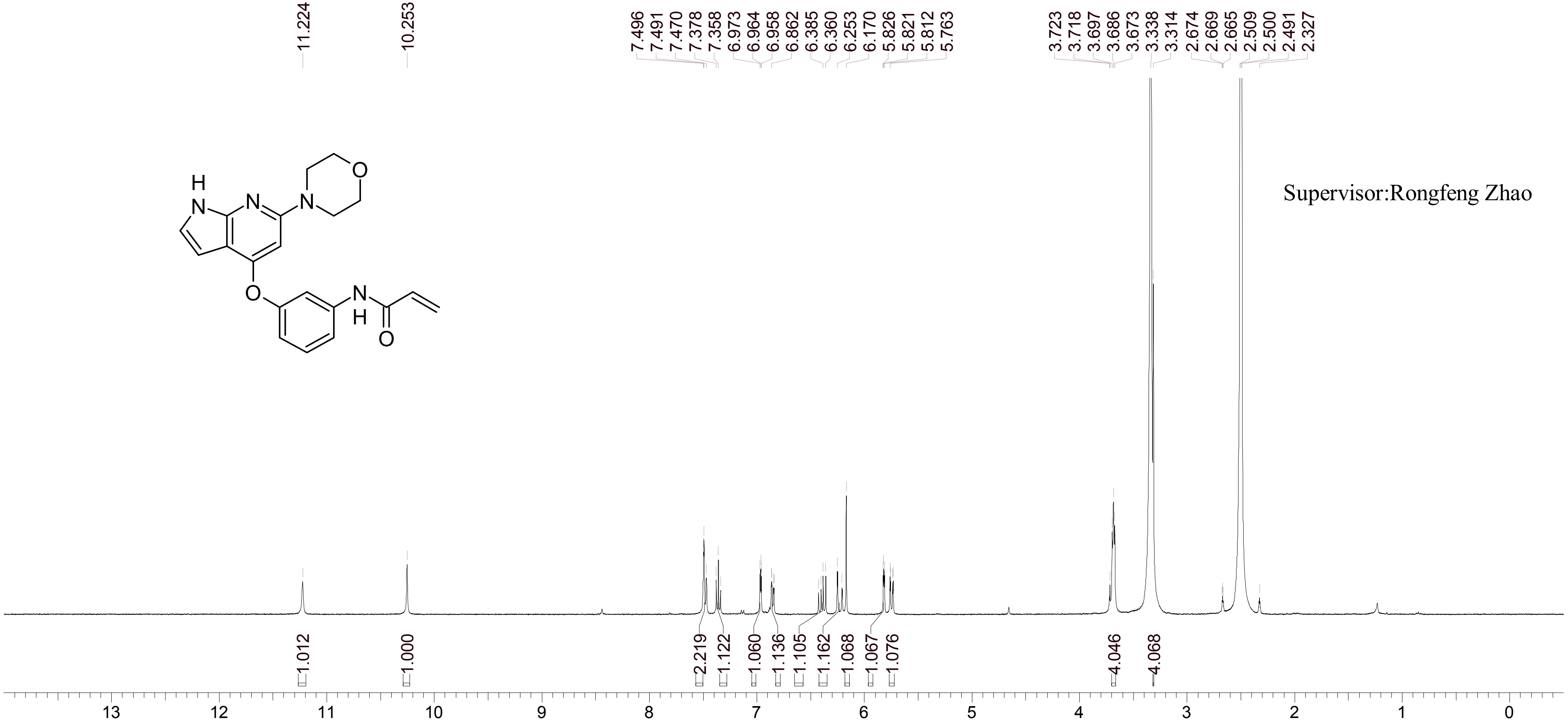

Supervisor:Rongfeng Zhao

Operator:

Date:

[redacted]

LCMS REPORT

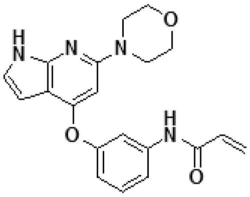

->

Exact Mass: 364.1535

```
Compound ID      : GK-PD1b
Sample ID        : EW20854-35-P1E
Injection Date   : Thu, 31. Oct. 2019        15:20:11
Location         : P2-F-07
Inj. Vol.        : 4.00        uL
Acq   Method     : D:\METHODS\WUXIAB10.M
Data Filename    : D:\2019\201910\191031LCMS-F1\EW20854-35-P1E.D
Instrument       : CAS-WH-LCMS-F
```

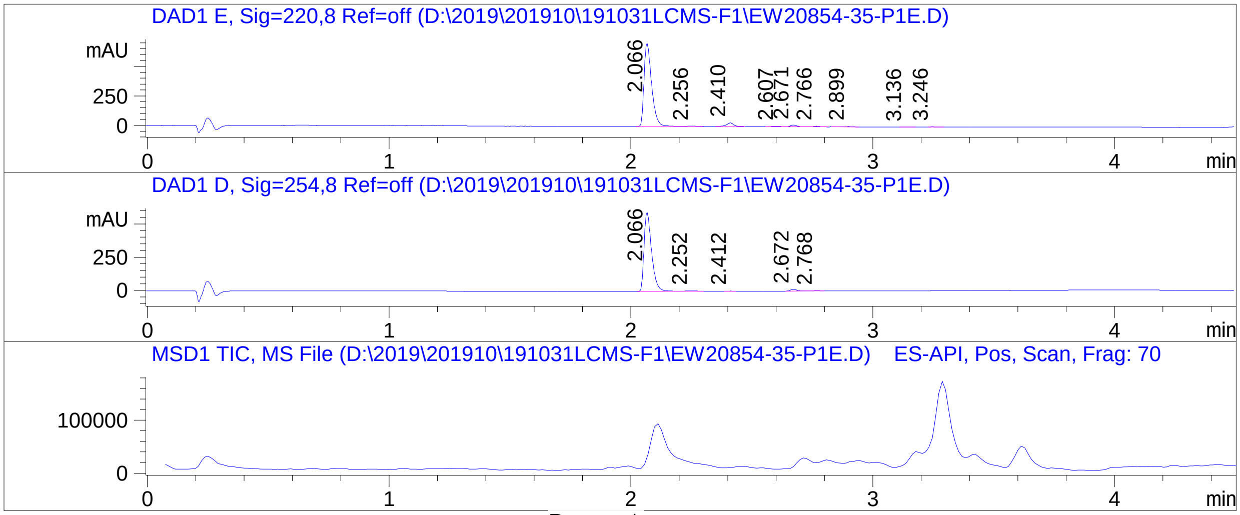

Report

Signal 1 : DAD1 E, Sig=220,8 Ref=off

#	Meas. Ret.	Height	Width	Area	Area %
1	2.066	705.127	0.036	1530.8291	91.86
2	2.256	4.331	0.048	12.4430	0.75
3	2.410	31.301	0.035	66.2521	3.98
4	2.607	3.239	0.039	7.5003	0.45
5	2.671	15.111	0.029	26.1020	1.57
6	2.766	5.076	0.029	8.7604	0.53
7	2.899	3.270	0.036	7.0901	0.43
8	3.136	1.453	0.041	3.5342	0.21
9	3.246	2.422	0.027	3.9693	0.24

Signal 2 : DAD1 D, Sig=254,8 Ref=off

#	Meas. Ret.	Height	Width	Area	Area %
1	2.066	594.990	0.036	1288.8213	96.64
2	2.252	3.686	0.053	11.7700	0.88
3	2.412	0.973	0.024	1.4118	0.11
4	2.672	14.440	0.030	25.8134	1.94
5	2.768	3.248	0.030	5.7657	0.43

Operator:____________________

Date:____________________

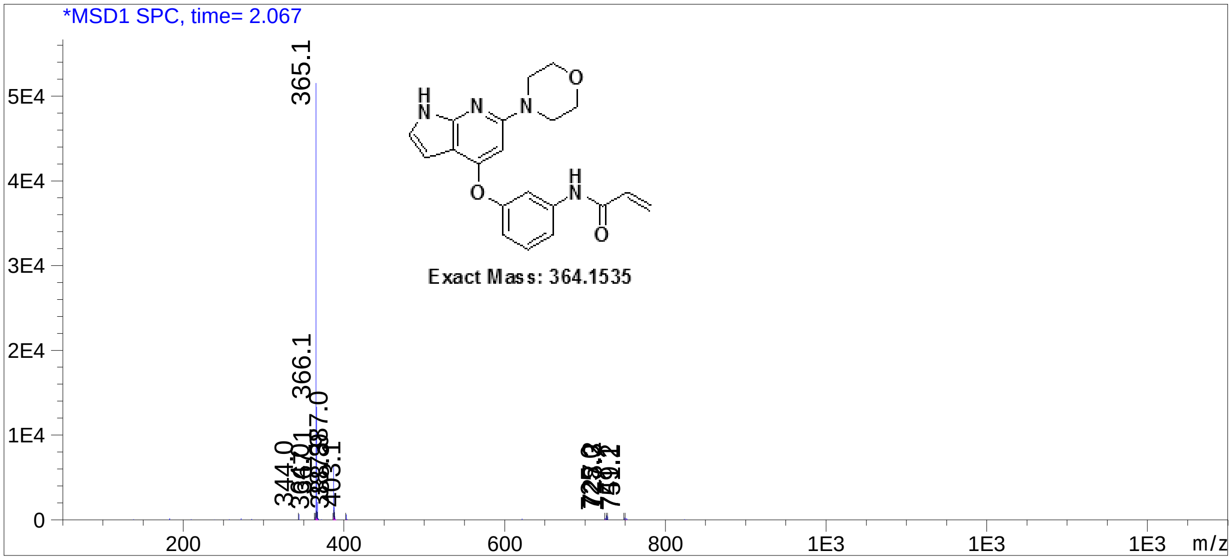
*MSD1 SPC, time= 2.067
365.1
366.1
344.0
403.1
Exact Mass: 364.1535
5E4
4E4
3E4
2E4
1E4
0
200
400
600
800
1E3
m/z

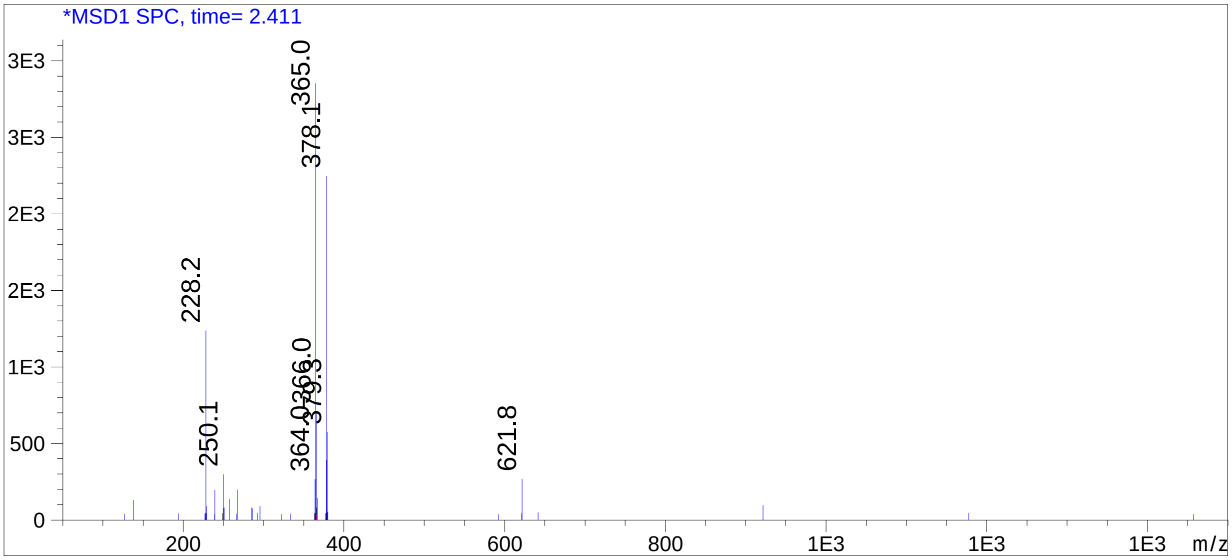
*MSD1 SPC, time= 2.411
365.0
378.1
228.2
250.1
366.0
379.3
364.0
621.8
3E3
2E3
1E3
500
0
200
400
600
800
1E3
m/z

Confidential. For research only, Not for regulatory filing.

[redacted]

Compound ID: GK-PD1b1

EW20854-57-P1B DMSO Bruker_E_400MHz

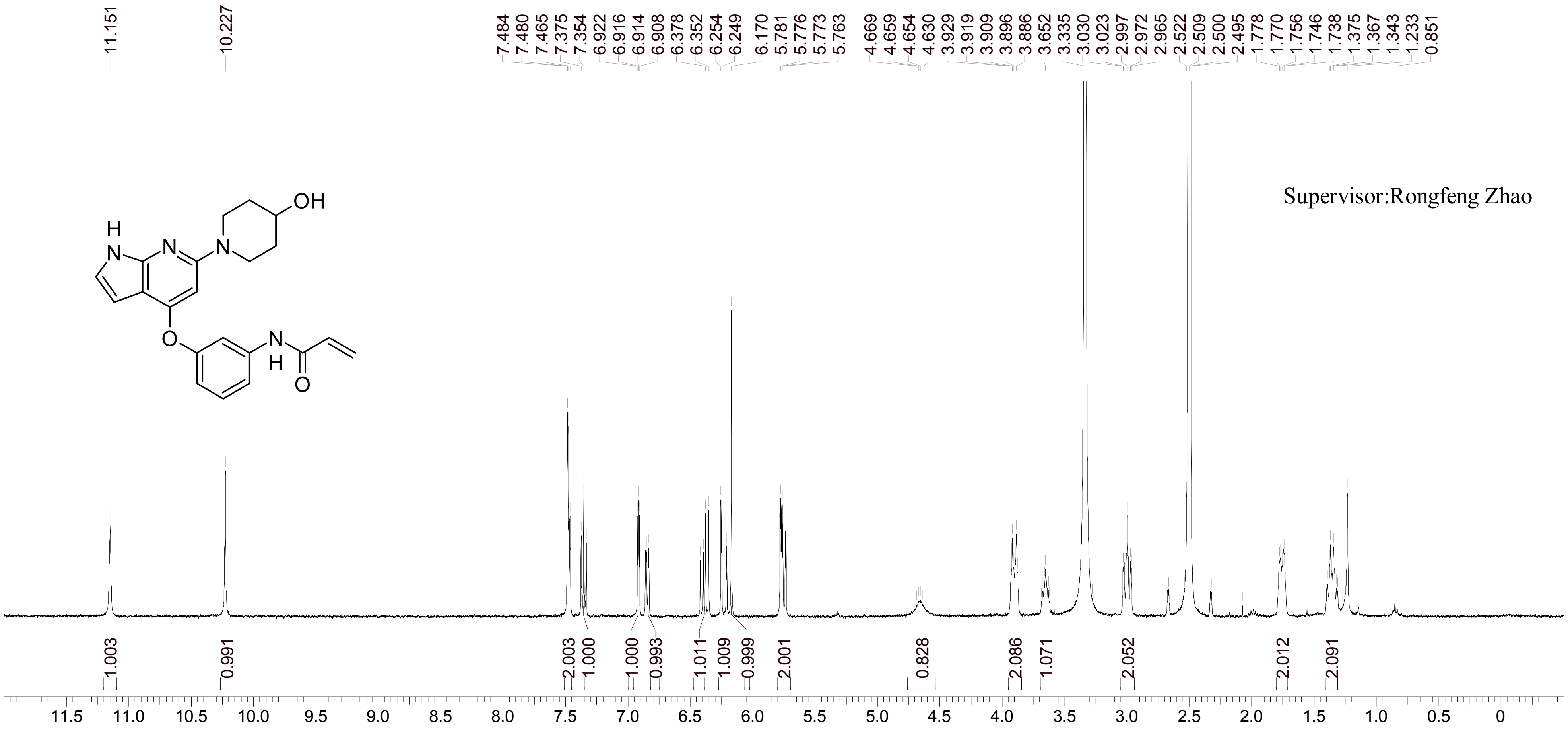

Operator: Date:

[redacted]

Confidential. For research information only

LCMS REPORT

Compound ID : GK-PD1b1
Sample ID : EW20854-57-P1G
Injection Vol : 7ul
Location : vial24
Acq Method : D:\method\5-95CD_R_220&254_POS.lcm
Org DataFile : D:\DATA\2019\1912\191212\EW20854-57-P1G.lcd
Injection Date : 2019-12-12 11:52:55
Instrument :LCMS-M 17-102

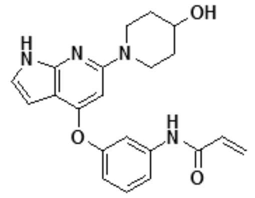

Exact Mass: 378.1692

Chromatogram

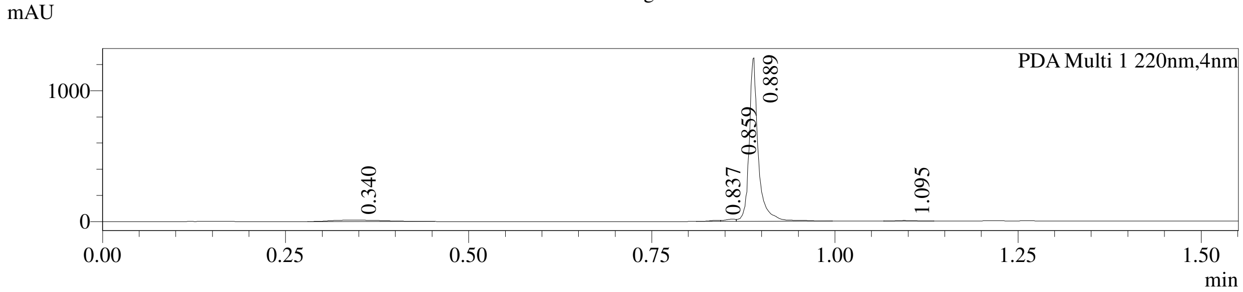

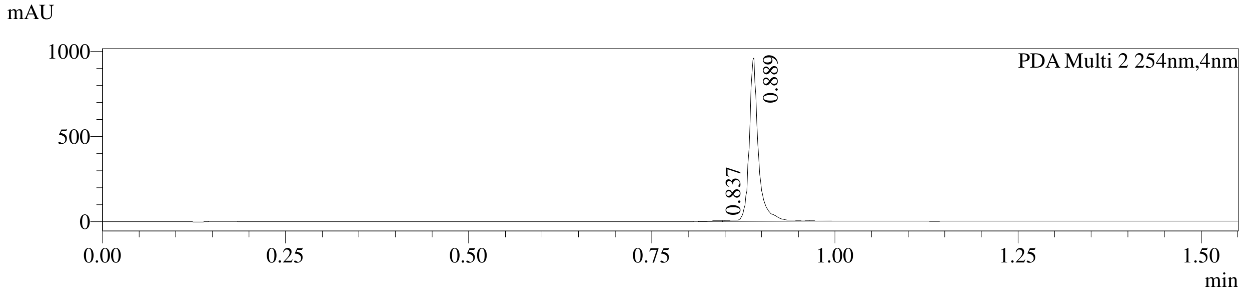

1 PDA Multi 1 / 220nm,4nm
2 PDA Multi 2 / 254nm,4nm

MS Chromatogram

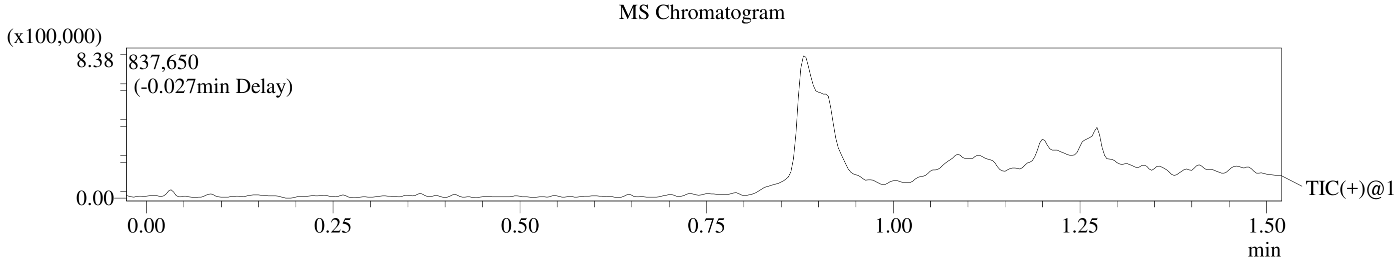

Integration Result

Peak Table

PDA Ch1 220nm

Peak#	Ret. Time	Height	Height%	USP Width	Area	Area%
1	0.340	10996	0.853	0.131	55314	4.753
2	0.837	7685	0.596	0.000	8010	0.688
3	0.859	16586	1.286	0.105	17018	1.462
4	0.889	1249605	96.912	0.054	1075761	92.437
5	1.095	4553	0.353	0.047	7673	0.659

Peak Table

PDA Ch2 254nm

Peak#	Ret. Time	Height	Height%	USP Width	Area	Area%
1	0.837	4891	0.506	0.084	5342	0.623
2	0.889	960947	99.494	0.022	852660	99.377

Operator:______________

Date:_________________

Confidential. For research information only

Mass Spectrum

RetTime: 0.340 Datafile: D:\DATA\2019\1912\191212\EW20854-57-P1G.lcd

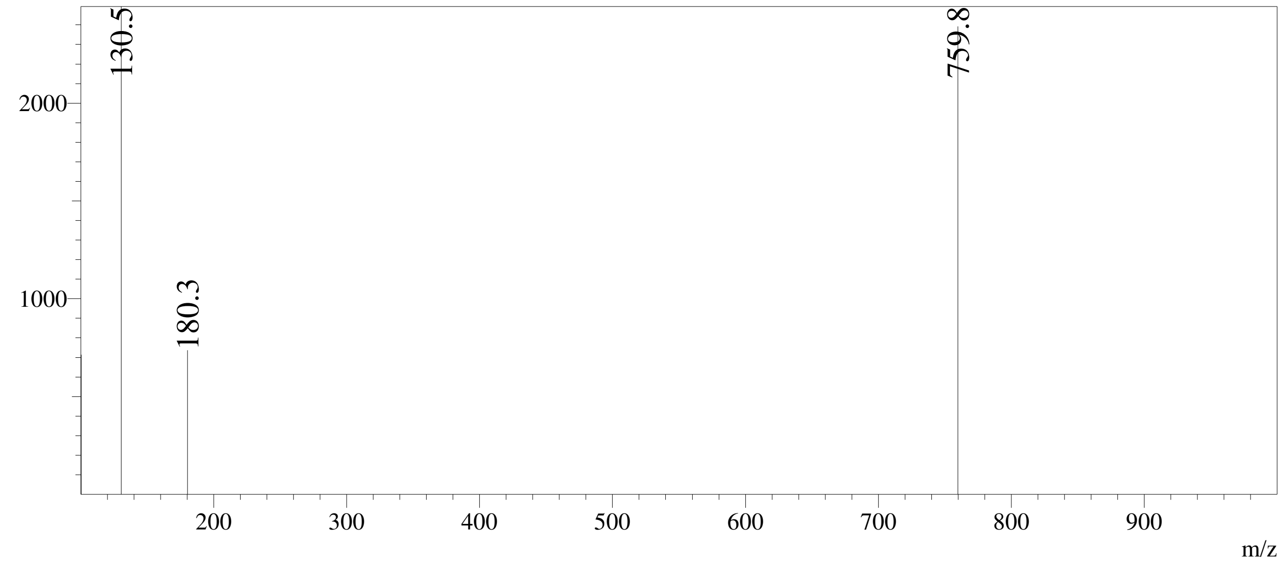

RetTime: 0.836 Datafile: D:\DATA\2019\1912\191212\EW20854-57-P1G.lcd

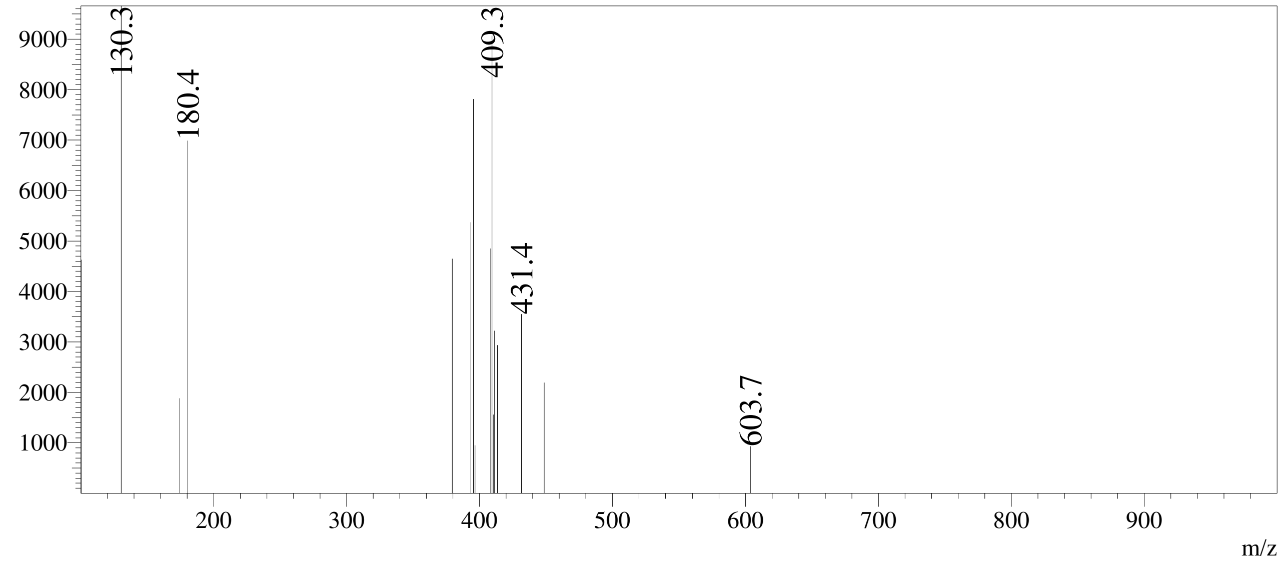

RetTime: 0.860 Datafile: D:\DATA\2019\1912\191212\EW20854-57-P1G.lcd

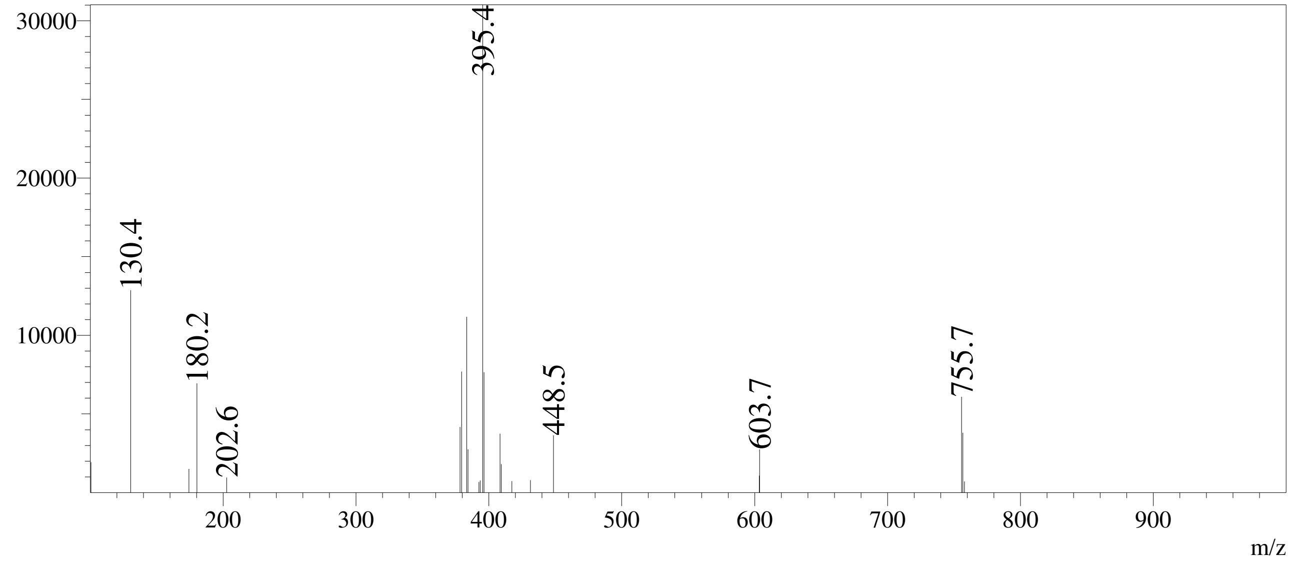

Confidential. For research information only

RetTime: 0.890 Datafile: D:\DATA\2019\1912\191212\EW20854-57-P1G.lcd

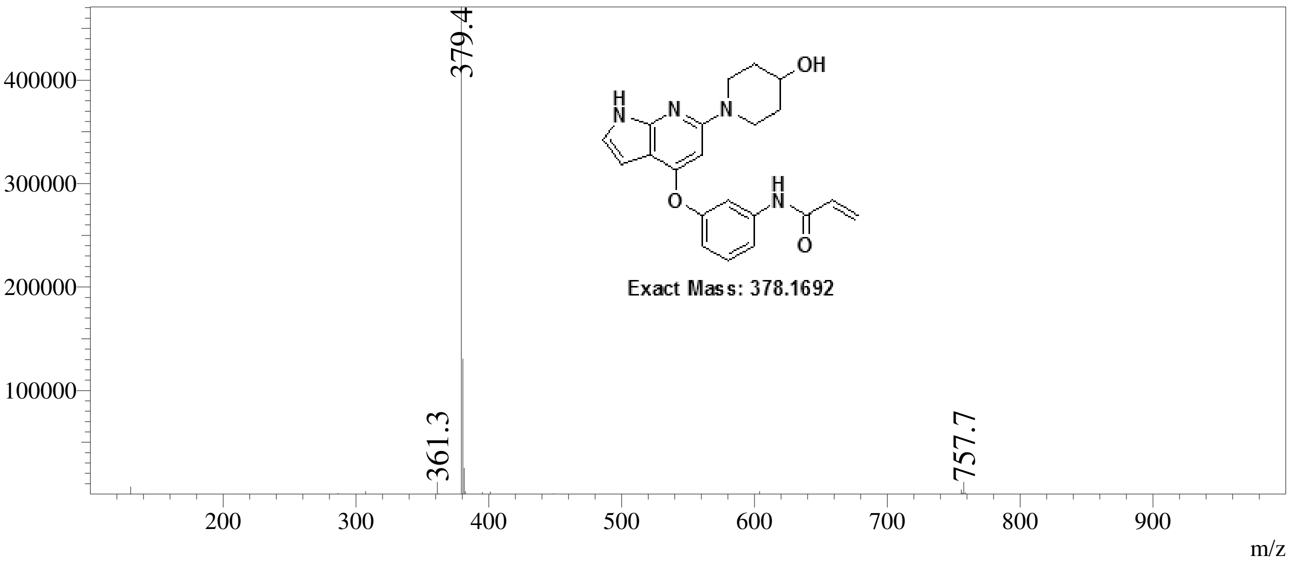

RetTime: 1.096 Datafile: D:\DATA\2019\1912\191212\EW20854-57-P1G.lcd

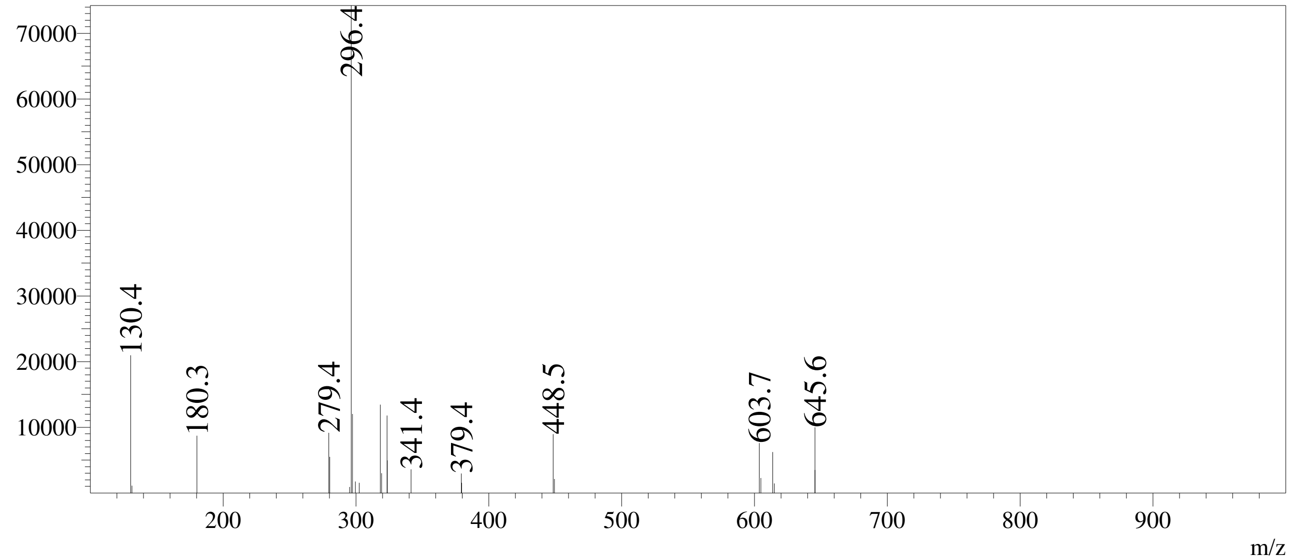

Confidential. For research information only

HPLC REPORT

Compound ID : GK-PD1b1
Sample ID : EW20854-57-P1I
Vial# : 86
Injection Volumn : 7
Filename : D:\DATA\2019\1912\191212\EW20854-57-P1I.lcd
Method Name : D:\method\10-80AB_4min.lcm
Instrument : HPLC-R
Run time : 2019-12-12 17:30:21

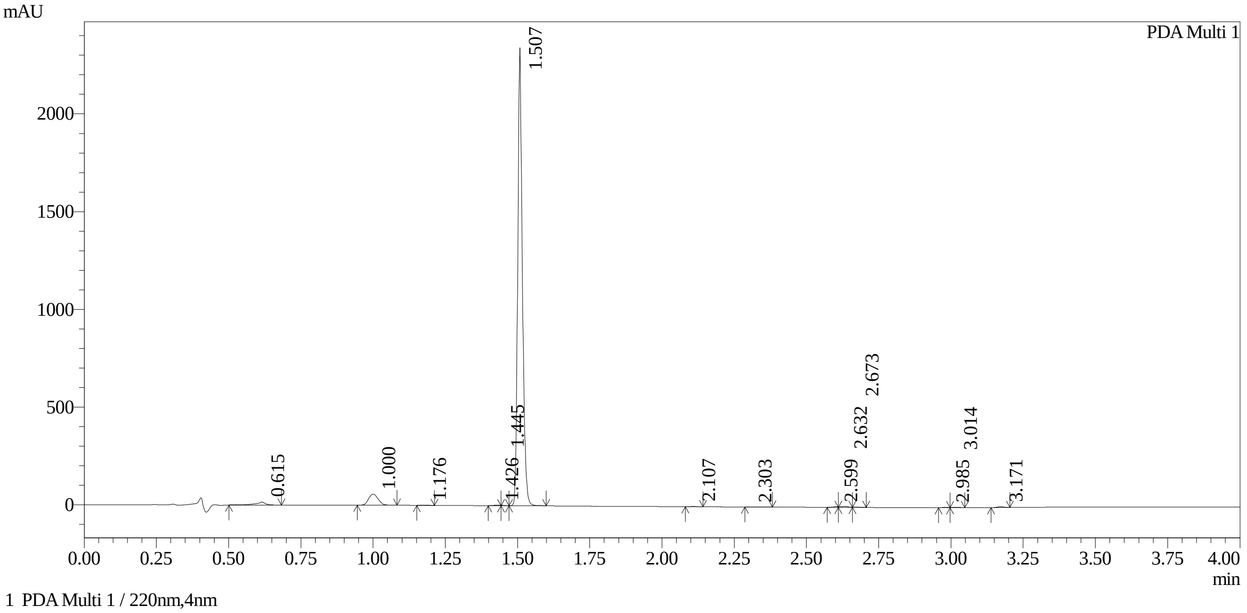

1 PDA Multi 1 / 220nm,4nm

Integration result

PeakTable

PDA Ch1 220nm

Peak#	Ret. Time	USP Width	Resolution	Height	Area	Area %
1	0.615	0.047	0.000	15547	39312	1.386
2	1.000	0.062	7.093	56262	131324	4.631
3	1.176	0.043	3.354	1192	1853	0.065
4	1.426	0.048	5.488	3209	4408	0.155
5	1.445	0.164	0.186	2171	1736	0.061
6	1.507	0.032	0.633	2342783	2625204	92.571
7	2.107	0.041	16.477	1570	2309	0.081
8	2.303	0.074	3.400	446	1556	0.055
9	2.599	0.068	4.179	2787	3702	0.131
10	2.632	0.054	0.531	5179	9449	0.333
11	2.673	0.046	0.809	2057	2921	0.103
12	2.985	0.049	6.613	1159	1592	0.056
13	3.014	0.048	0.580	1633	2589	0.091
14	3.171	0.043	3.435	5066	7930	0.280
Total				2441063	2835885	100.000

Operater : ________________

Date : ________________

Confidential. For research information only

HPLC REPORT

Compound ID : GK-PD1b1
Sample ID : EW20854-57-P1I
Filename : D:\DATA\2019\1912\191212\EW20854-57-P1I.lcd
Method Name : D:\method\10-80AB_4min.lcm
Instrument &Column : HPLC-R
Run time : 2019-12-12 17:30:21

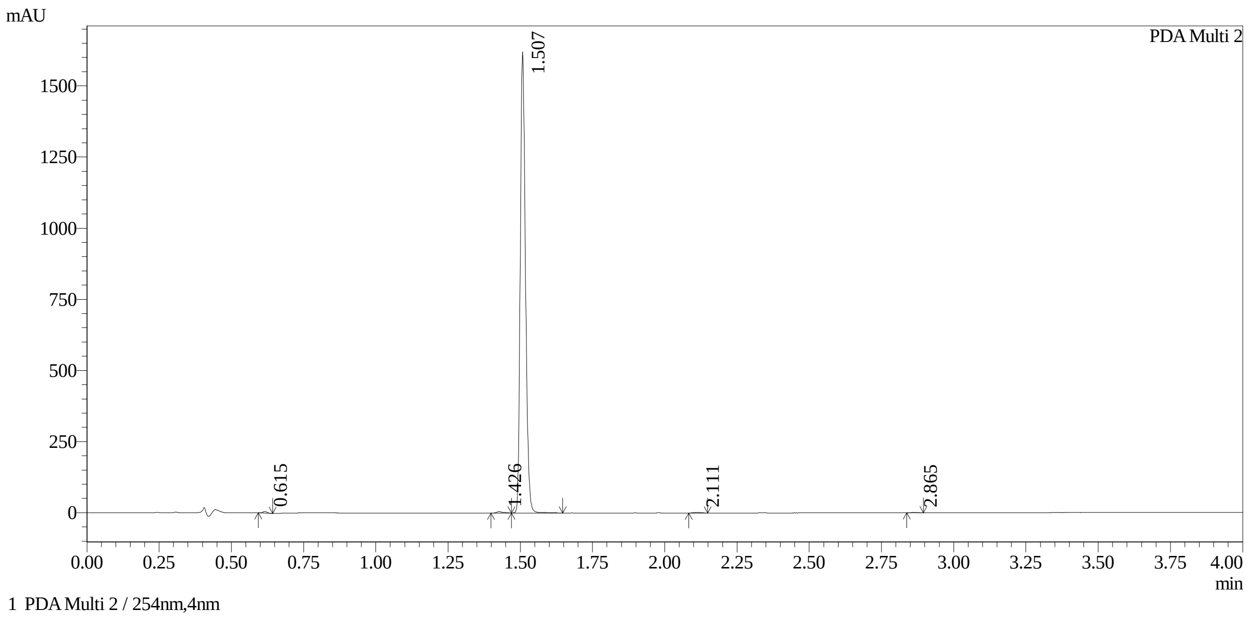

1 PDA Multi 2 / 254nm,4nm

Integration result

PeakTable

PDA Ch2 254nm

Peak#	Ret. Time	USP Width	Resolution	Height	Area	Area %
1	0.615	0.028	0.000	5763	5654	0.275
2	1.426	0.039	24.324	5018	7755	0.377
3	1.507	0.035	2.214	1620614	2036801	99.068
4	2.111	0.040	16.221	2219	3154	0.153
5	2.865	0.041	18.754	1773	2606	0.127
Total				1635386	2055970	100.000

Operater : ________________

Date : ________________

Confidential. For research information only

HPLC REPORT

Compound ID : GK-PD1b1
Sample ID : EW20854-57-P1I
Filename : D:\DATA\2019\1912\191212\EW20854-57-P1I.lcd
Method Name : D:\method\10-80AB_4min.lcm
Instrument &Column : HPLC-R
Run time : 2019-12-12 17:30:21

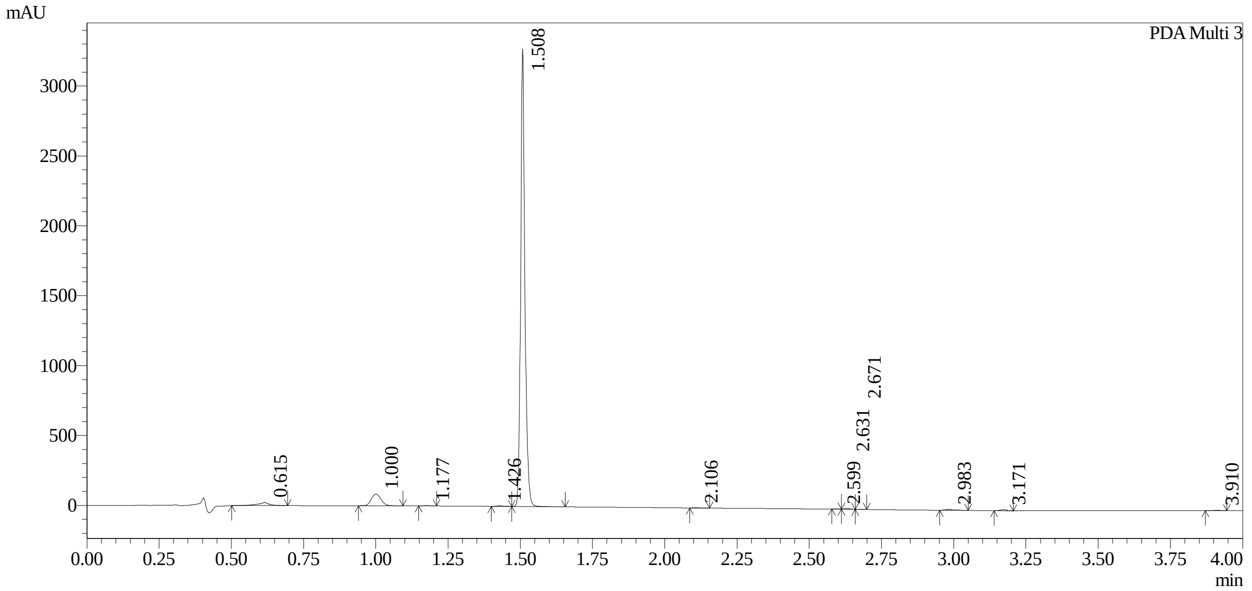

1 PDA Multi 3 / 215nm,4nm

Integration result

PeakTable

PDA Ch3 215nm

Peak#	Ret. Time	USP Width	Resolution	Height	Area	Area %
1	0.615	0.056	0.000	22423	65190	1.780
2	1.000	0.062	6.542	83070	194355	5.306
3	1.177	0.045	3.321	2337	3781	0.103
4	1.426	0.054	5.055	3909	7364	0.201
5	1.508	0.027	2.026	3274616	3354014	91.561
6	2.106	0.042	17.306	1514	2252	0.061
7	2.599	0.056	10.027	2060	2722	0.074
8	2.631	0.049	0.610	3630	5801	0.158
9	2.671	0.032	0.986	1035	1213	0.033
10	2.983	0.043	8.347	6069	11298	0.308
11	3.171	0.043	4.366	8718	13677	0.373
12	3.910	0.064	13.734	581	1490	0.041
Total				3409962	3663158	100.000

Operater : ________________

Date : ________________

Confidential. For research only, Not for regulatory filing.

[redacted]

Compound ID: GK-PD1b2

EW20854-45-P1A DMSO Bruker_G_400MHz

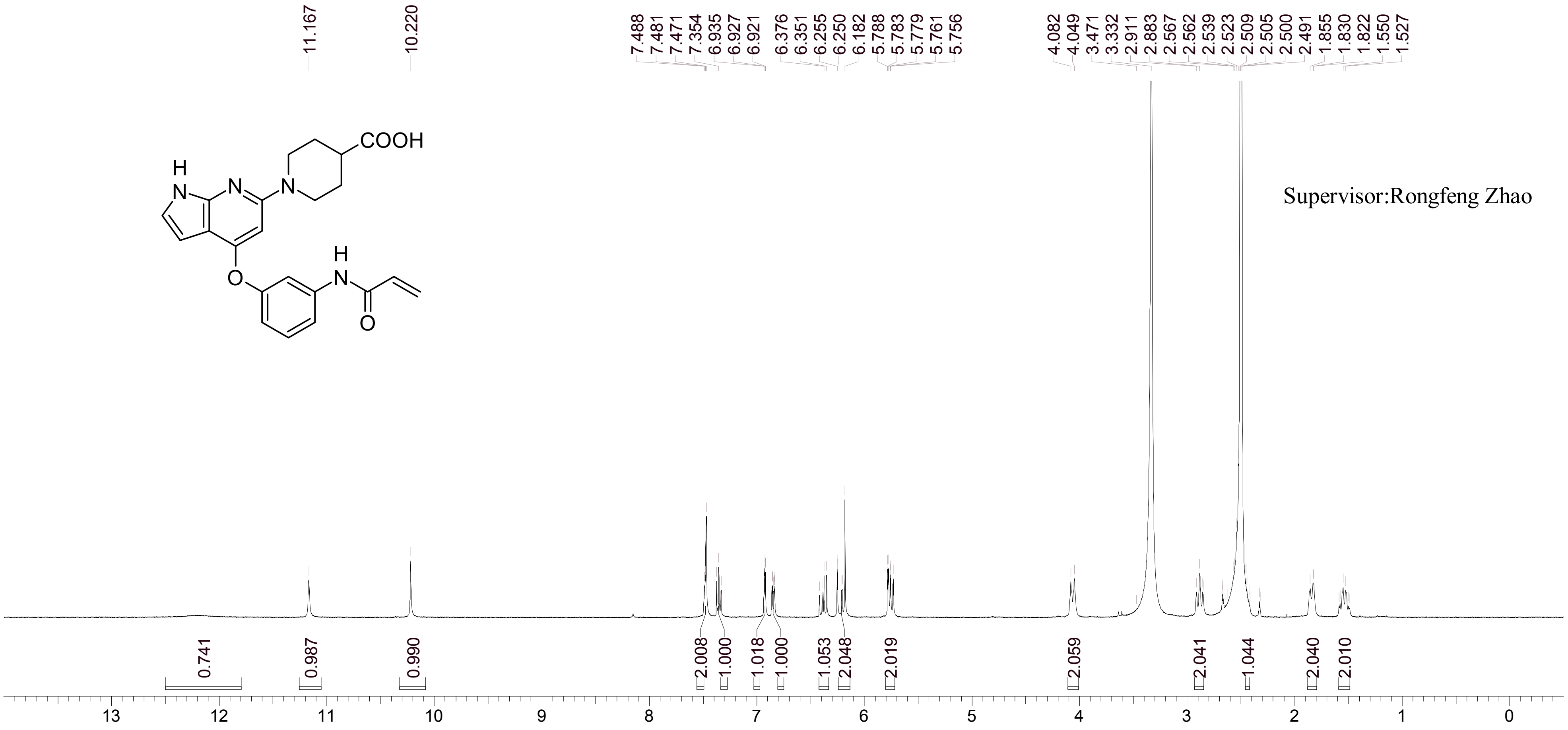

Operator: Date:

[redacted]

Confidential. For research information only

LCMS REPORT

Compound ID : GK-PD1b2
Sample ID : EW20854-45-P1F
Injection Vol : 5ul
Location : vial101
Acq Method : D:\method\0-60CD_R_220&254_POS.lcm
Org DataFile : D:\DATA\2019\1911\191118\EW20854-45-P1F.lcd
Injection Date : 2019-11-18 11:52:46
Instrument :LCMS-M 17-102

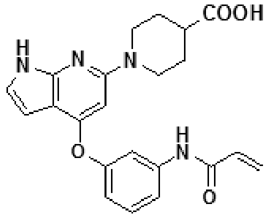

Exact Mass: 406.1641

Chromatogram

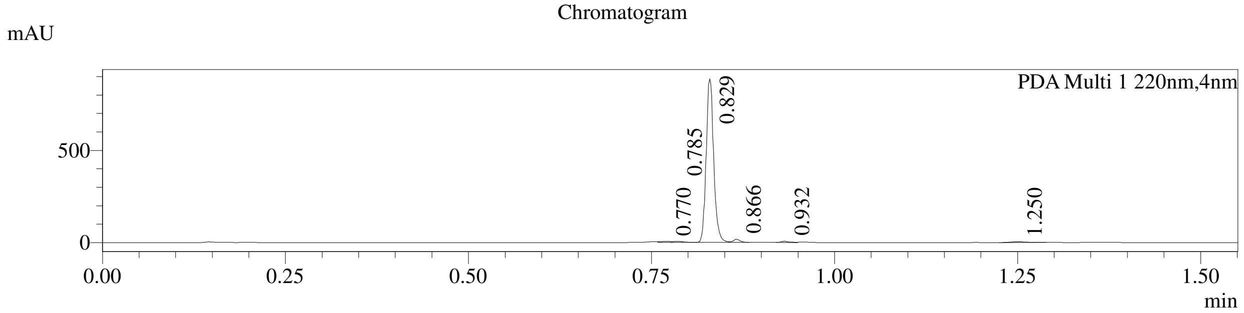

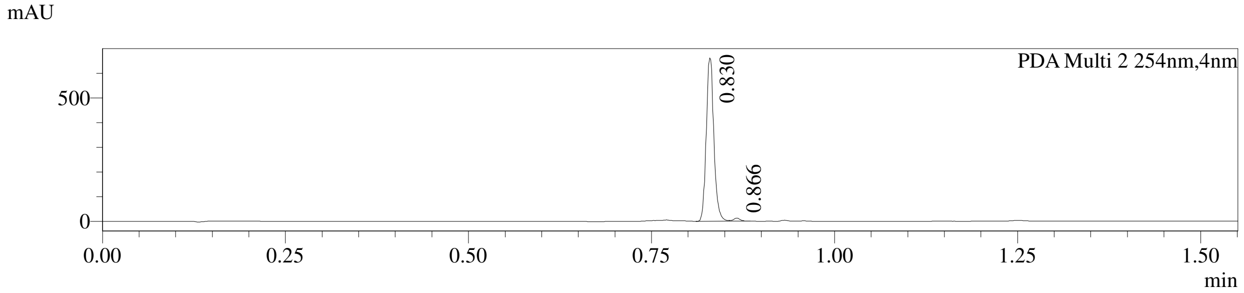

1 PDA Multi 1 / 220nm,4nm
2 PDA Multi 2 / 254nm,4nm

MS Chromatogram

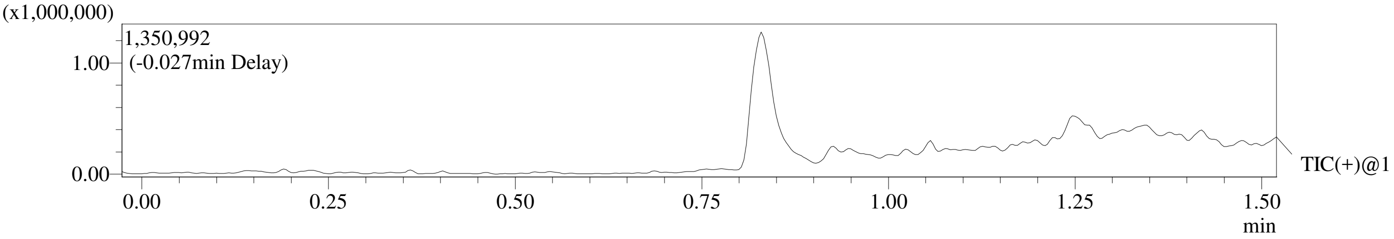

Integration Result

Peak Table

PDA Ch1 220nm

Peak#	Ret. Time	Height	Height%	USP Width	Area	Area%
1	0.770	6327	0.682	0.038	5427	0.830
2	0.785	5528	0.596	0.042	5383	0.823
3	0.829	886663	95.598	0.021	619768	94.789
4	0.866	16938	1.826	0.020	10890	1.665
5	0.932	7027	0.758	0.021	5459	0.835
6	1.250	5008	0.540	0.037	6915	1.058

Peak Table

PDA Ch2 254nm

Peak#	Ret. Time	Height	Height%	USP Width	Area	Area%
1	0.830	661284	98.099	0.022	469569	98.228
2	0.866	12816	1.901	0.019	8473	1.772

Confidential. For research information only

Operator:_______________

Date:___________________

Confidential. For research information only

Mass Spectrum

RetTime: 0.770 Datafile: D:\DATA\2019\1911\191118\EW20854-45-P1F.lcd

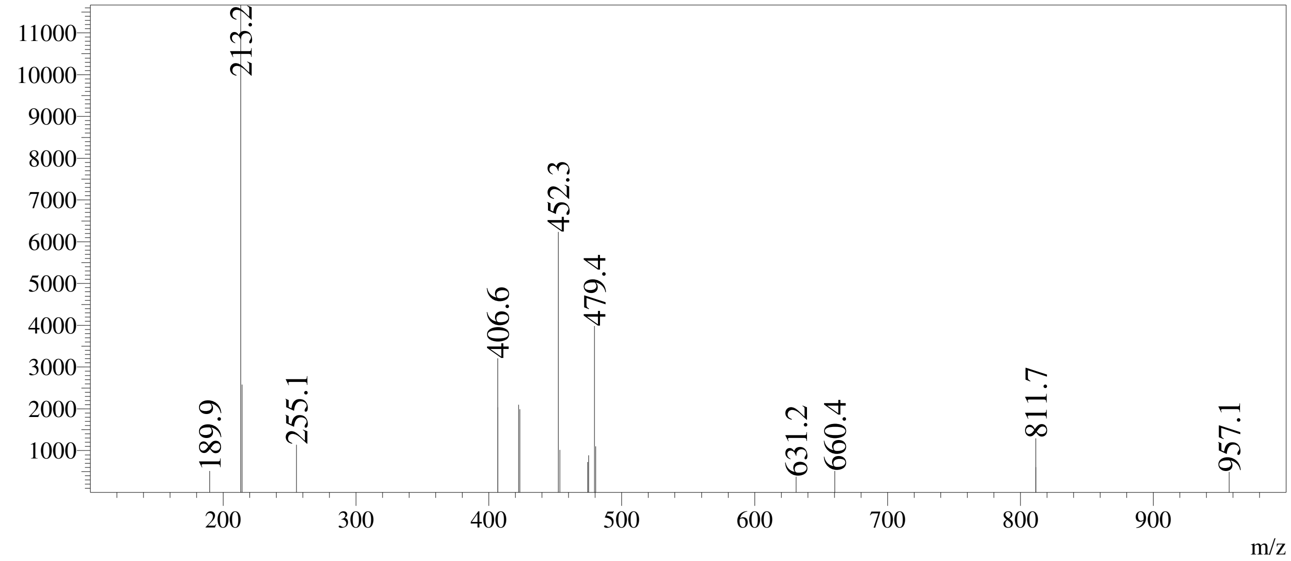

RetTime: 0.786 Datafile: D:\DATA\2019\1911\191118\EW20854-45-P1F.lcd

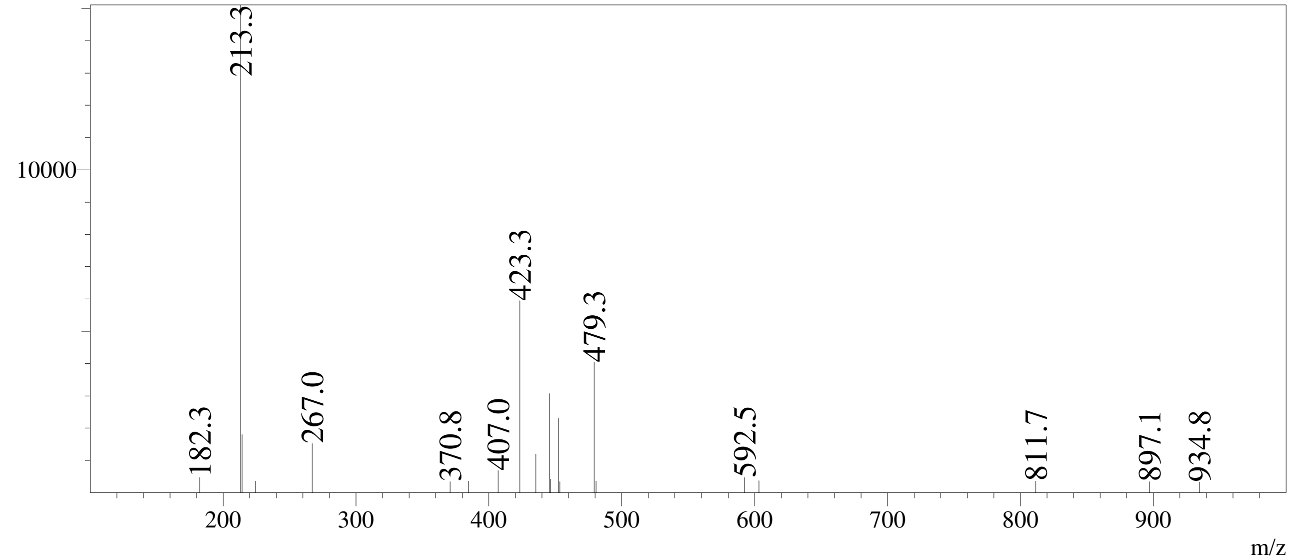

RetTime: 0.830 Datafile: D:\DATA\2019\1911\191118\EW20854-45-P1F.lcd

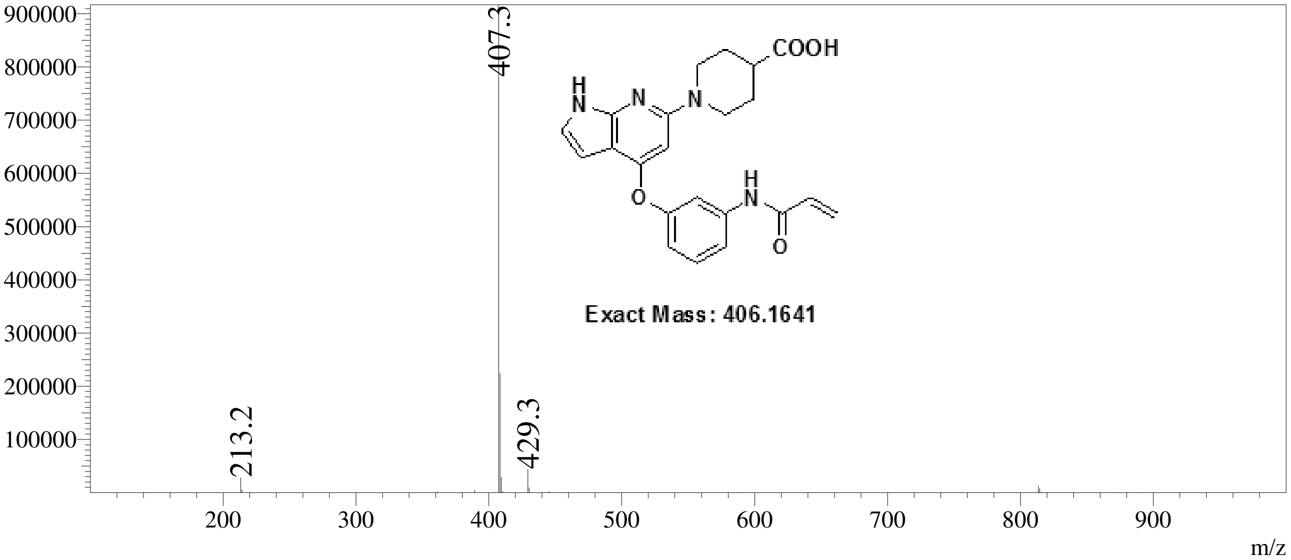

Confidential. For research information only

RetTime: 0.866 Datafile: D:\DATA\2019\1911\191118\EW20854-45-P1F.lcd

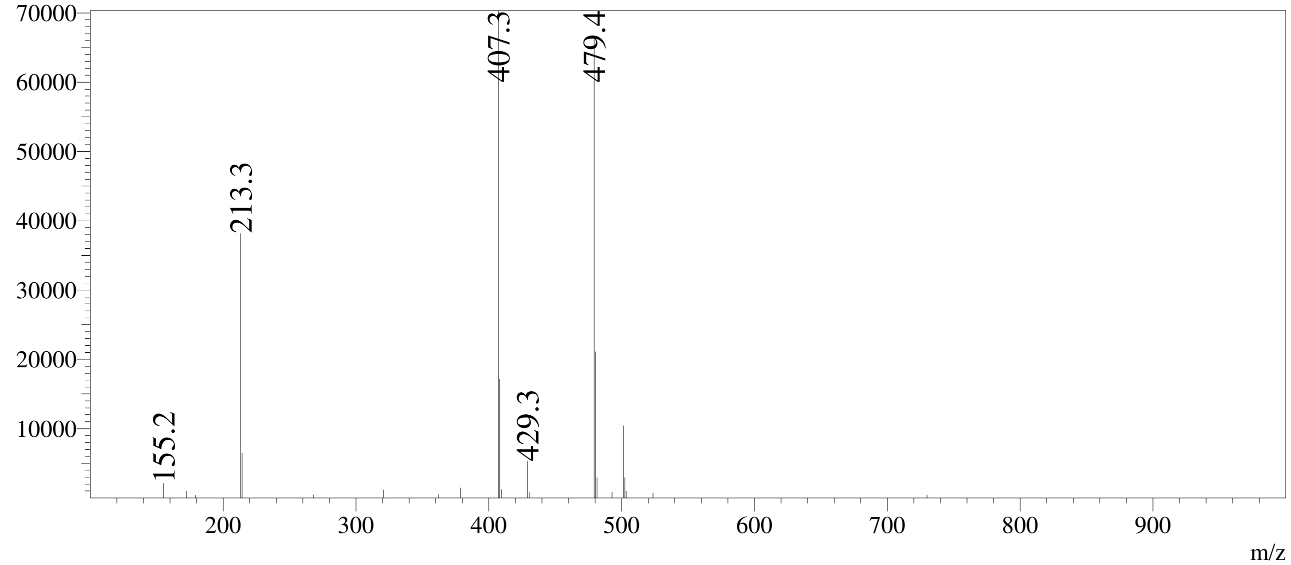

RetTime: 0.933 Datafile: D:\DATA\2019\1911\191118\EW20854-45-P1F.lcd

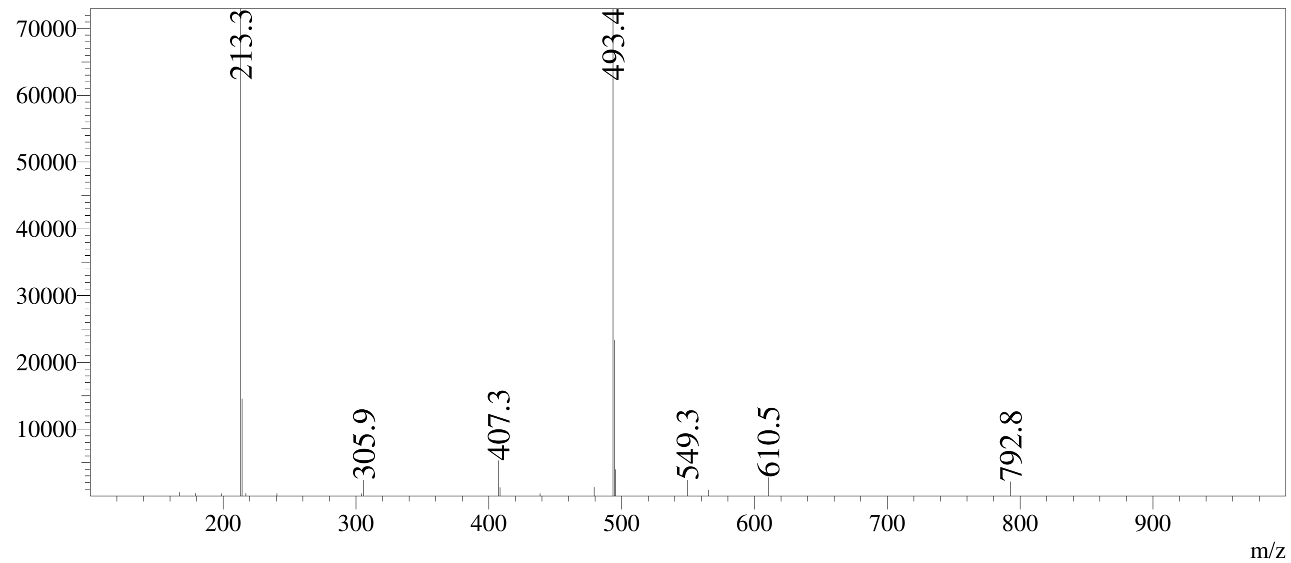

RetTime: 1.250 Datafile: D:\DATA\2019\1911\191118\EW20854-45-P1F.lcd

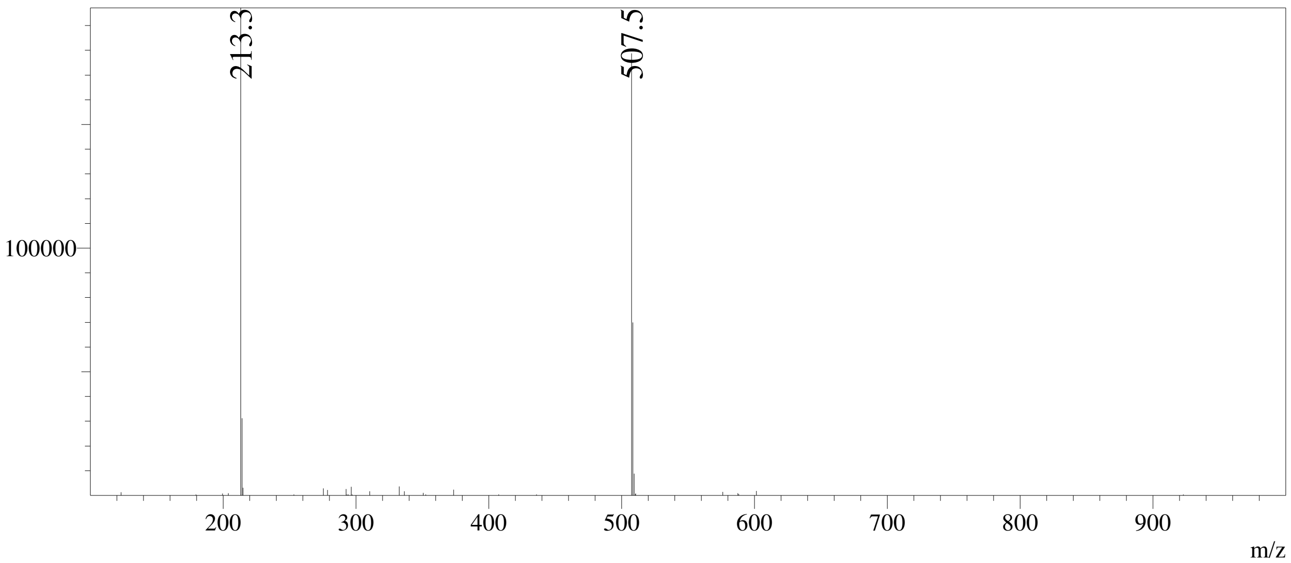

Confidential. For research information only

HPLC REPORT

Compound ID : GK-PD1b2
Sample ID : EW20854-45-P1E
Vial# : 105
Injection Volumn : 5
Filename : D:\DATA\2019\1911\191118\EW20854-45-P1E.lcd
Method Name : D:\method\0-60CD_4min.lcm
Instrument : HPLC-AG
Run time : 2019-11-18 11:49:23

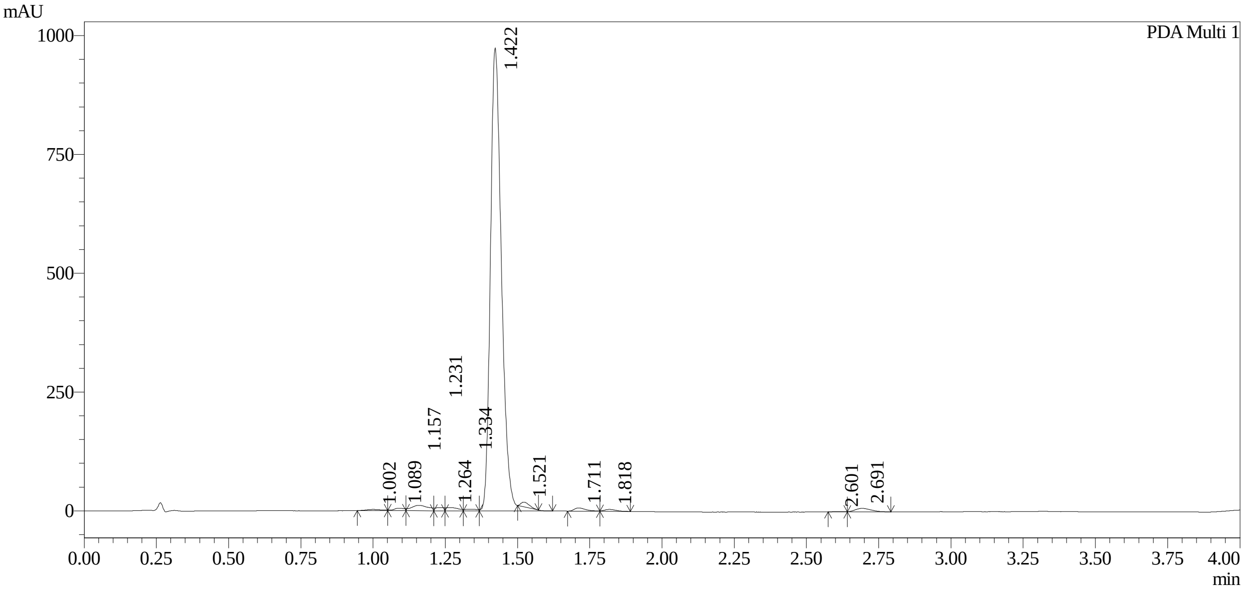

1 PDA Multi 1 / 220nm,4nm

Integration result

PeakTable

PDA Ch1 220nm

Peak#	Ret. Time	USP Width	Resolution	Height	Area	Area %
1	1.002	0.112	0.000	2539	9591	0.342
2	1.089	0.110	0.788	5201	13264	0.473
3	1.157	0.121	0.580	11356	46565	1.661
4	1.231	0.443	0.266	6486	14418	0.514
5	1.264	0.260	0.092	6714	19821	0.707
6	1.334	0.176	0.323	3481	10262	0.366
7	1.422	0.070	0.711	974478	2608006	93.023
8	1.521	0.056	1.580	9959	20677	0.738
9	1.711	0.073	2.934	6724	19437	0.693
10	1.818	0.077	1.423	4060	11683	0.417
11	2.601	0.071	10.629	659	1824	0.065
12	2.691	0.103	1.026	7295	28077	1.001
Total				1038951	2803625	100.000

Operater : ________________

Date : ________________

Confidential. For research information only

HPLC REPORT

Compound ID : GK-PD1b2
Sample ID : EW20854-45-P1E
Filename : D:\DATA\2019\1911\191118\EW20854-45-P1E.lcd
Method Name : D:\method\0-60CD_4min.lcm
Instrument : HPLC-AG
Run time : 2019-11-18 11:49:23

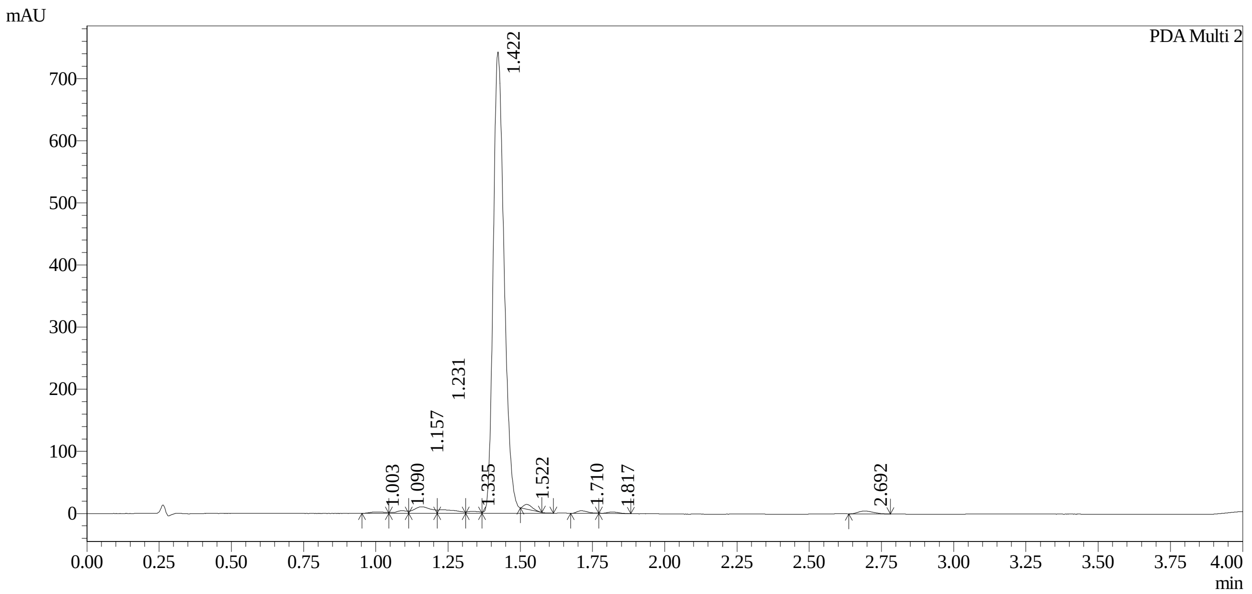

1 PDA Multi 2 / 254nm,4nm

Integration result

PeakTable

PDA Ch2 254nm

Peak#	Ret. Time	USP Width	Resolution	Height	Area	Area %
1	1.003	0.144	0.000	2321	8503	0.398
2	1.090	0.314	0.378	4154	11872	0.555
3	1.157	0.123	0.306	10522	43580	2.037
4	1.231	0.259	0.391	5811	25610	1.197
5	1.335	0.129	0.533	3084	8824	0.413
6	1.422	0.070	0.879	742634	1989604	93.015
7	1.522	0.056	1.575	7619	15893	0.743
8	1.710	0.070	2.987	4092	11036	0.516
9	1.817	0.074	1.481	2560	7324	0.342
10	2.692	0.098	10.176	4578	16774	0.784
Total				787374	2139019	100.000

Operater : ________________

Date : ________________

Confidential. For research information only

HPLC REPORT

Compound ID : GK-PD1b2
Sample ID : EW20854-45-P1E
Filename : D:\DATA\2019\1911\191118\EW20854-45-P1E.lcd
Method Name : D:\method\0-60CD_4min.lcm
Instrument : HPLC-AG
Run time : 2019-11-18 11:49:23

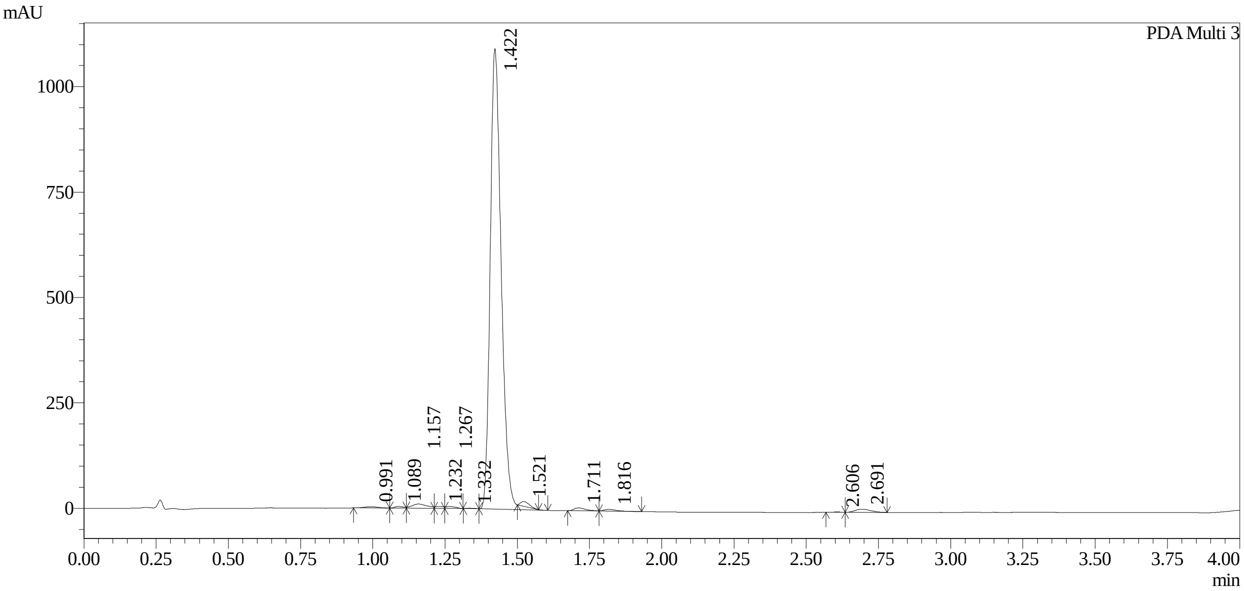

1 PDA Multi 3 / 215nm,4nm

Integration result

PeakTable

PDA Ch3 215nm

Peak#	Ret. Time	USP Width	Resolution	Height	Area	Area %
1	0.991	0.096	0.000	3084	11088	0.361
2	1.089	0.073	1.165	3904	8695	0.283
3	1.157	0.102	0.775	10082	37378	1.216
4	1.232	0.230	0.451	4453	9371	0.305
5	1.267	0.177	0.171	4429	10679	0.348
6	1.332	0.048	0.578	904	1548	0.050
7	1.422	0.070	1.532	1091644	2905647	94.558
8	1.521	0.056	1.575	11113	23185	0.755
9	1.711	0.073	2.943	6972	20652	0.672
10	1.816	0.082	1.351	4364	13969	0.455
11	2.606	0.070	10.427	814	2064	0.067
12	2.691	0.102	0.987	7533	28591	0.930
Total				1149297	3072866	100.000

Operater : ________________

Date : ________________
